# Two Copper-Carbenes from One Diazo Compound

**DOI:** 10.1021/jacs.1c01483

**Published:** 2021-03-18

**Authors:** María Álvarez, Maria Besora, Francisco Molina, Feliu Maseras, Tomás R. Belderrain, Pedro J. Pérez

**Affiliations:** † Laboratorio de Catálisis Homogénea, Unidad Asociada al CSIC, CIQSO-Centro de Investigación en Química Sostenible and Departamento de Química, Universidad de Huelva, 21007 Huelva, Spain; ‡ Institute of Chemical Research of Catalonia, ICIQ, Av. Països Catalans, 16, Barcelona Institut of Science and Technology, 43007 Tarragona, Spain; § Departament de Química Física i Inorgànica, Universitat Rovira i Virgili, 43007 Tarragona, Spain; ∥ Departament de Química, Universitat Autònoma de Barcelona, 08193 Bellaterra, Spain

## Abstract

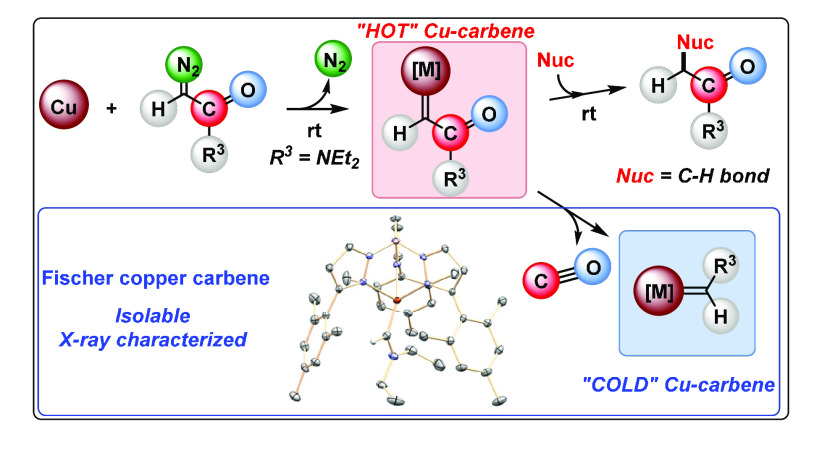

Many transition-metal complexes ML_n_ decompose diazo compounds N_2_=CR^1^R^2^ generating metal-carbenes L_n_M=CR^1^R^2^ which transfer the carbene group to other substrates, constituting an important tool in organic synthesis. All previous reports have shown that the CR^1^R^2^ fragment at the metal-carbene remains intact from the parent diazo compound. Herein we report the detection and isolation of a monosubstituted copper carbene where the CR^1^R^2^ ligand has undergone a modification from the initial diazo reagent. When Tp^Ms^Cu(THF) (Tp^Ms^ = hydrotris(3-mesityl)pyrazolylborate ligand) was reacted with *N,N*-diethyl diazoacetamide [N_2_=C(H)(CONEt_2_)], the stable copper carbene Tp^Ms^Cu=C(H)(NEt_2_) was isolated, resulting from a decarbonylation process, with carbon monoxide being trapped as Tp^Ms^Cu(CO). The simultaneous observation of products derived from the intramolecular carbene insertion reaction into C–H bonds demonstrates that the expected Tp^Ms^Cu=C(H)(CONEt_2_) complex is also formed. Experimental data, DFT calculations, and microkinetic models allow us to propose that the latter undergoes CO loss en route to the former.

## Introduction

**Scheme 1:**
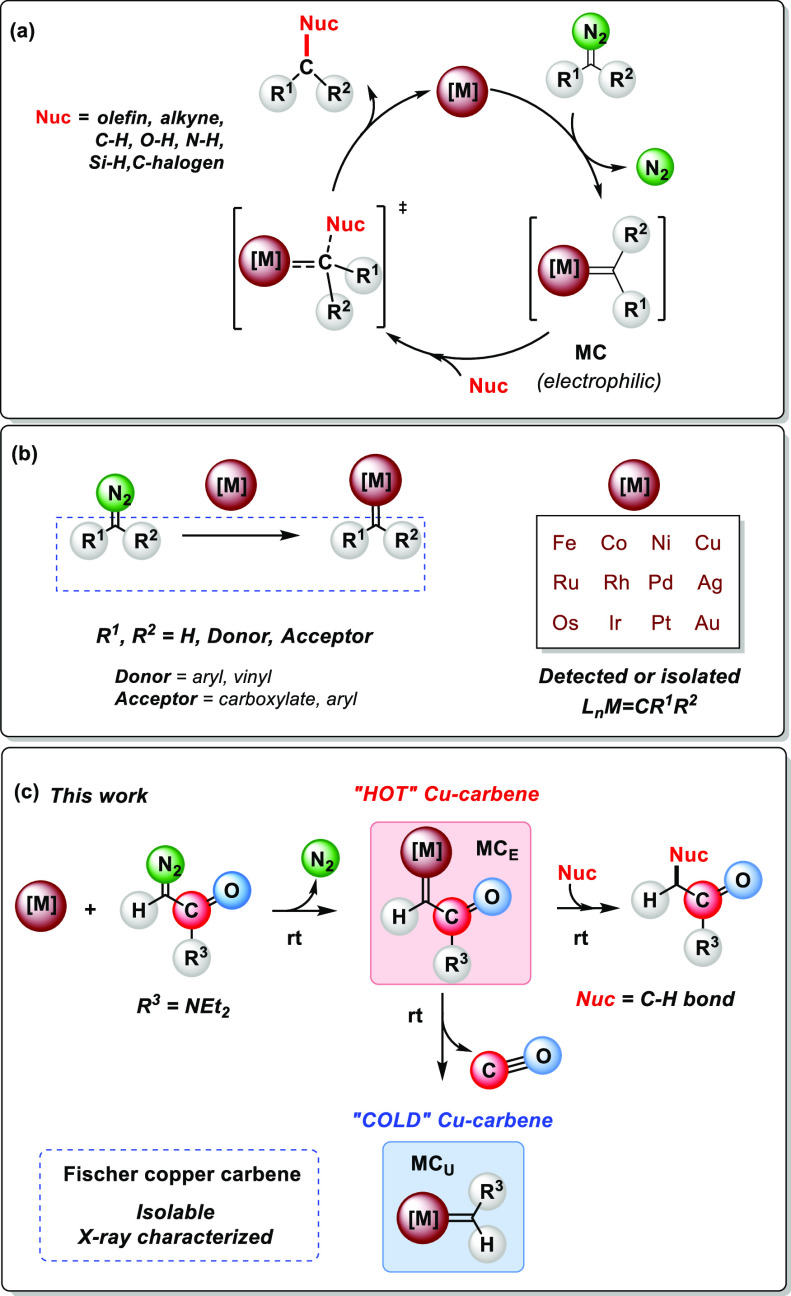
Metal-Catalyzed Carbene Transfer from Diazo Compounds and the Formation of Metalocarbene Intermediates

More than a century after Buchner postulated the existence of carbene CR_2_ groups during the thermal decomposition of ethyl diazoacetate,^1^ the catalytic transfer of such moiety from diazo compounds yet constitutes an area of continuous growth.^2,3^ Such process consists of the metal-induced decomposition of the diazo reagent in a process in which molecular N_2_ is extruded and a metallocarbene intermediate **MC** is formed (Scheme 1a).^4^ This species is electrophilic^5^ in nature and reacts with available nucleophiles transferring the carbene group, thus liberating the metal to continue the catalytic cycle. This strategy has been successfully employed in the addition of carbene groups to unsaturated bonds or in its insertion into C–H or other C–X bonds, both intra- and intermolecularly.^6−8^

With the appropriate tuning of the carbene substituents and the metal precursor, several metallocarbene complexes have been detected, some of them being isolated and structurally characterized. All metals from groups 8 to 11 are known to catalyze the carbene transfer from diazo compounds (Scheme 1b), and at least one detected/isolated example of a metal-carbene intermediate formed from a diazo compound has been reported for each of them.^8−18^ In all cases, the CR^1^R^2^ moiety in the initial diazo compound appears unmodified in the subsequent metal-carbene intermediate (Scheme 1b), from where it is further transferred to the nucleophile. From here, it is assumed that the carbene ligand is always transferred without modification. One of the most popular diazo reagents are diazocarbonyl compounds,^4^ with a CO group directly bonded to the diazo functionality: the acceptor nature of the −COR group favors the transfer of the carbene group toward the nucleophile.

Herein we report the observation of the unprecedented modification of the carbene unit during the course of a copper-catalyzed transformation. The use of a diazoacetamide compound (Scheme 1c) bearing a CONR_2_ substituent leads to the formation of the expected, undetected, *hot*, highly reactive copper carbene **MC**_**E**_ (Scheme 1c) which promotes the intramolecular C–H bond functionalization of the ethyl groups of the amide fragment. In a parallel manner, **MC**_**E**_ undergoes the loss of CO en route to the formation of the unexpected, stable and isolable, *cold*, Fischer carbene complex **MC**_**U**_ (Scheme 1c), which has been structurally characterized.

## Results and Discussion

### Reaction of Tp^Ms^Cu(THF) and N_2_=C(H)(CONEt_2_)

**Scheme 2:**
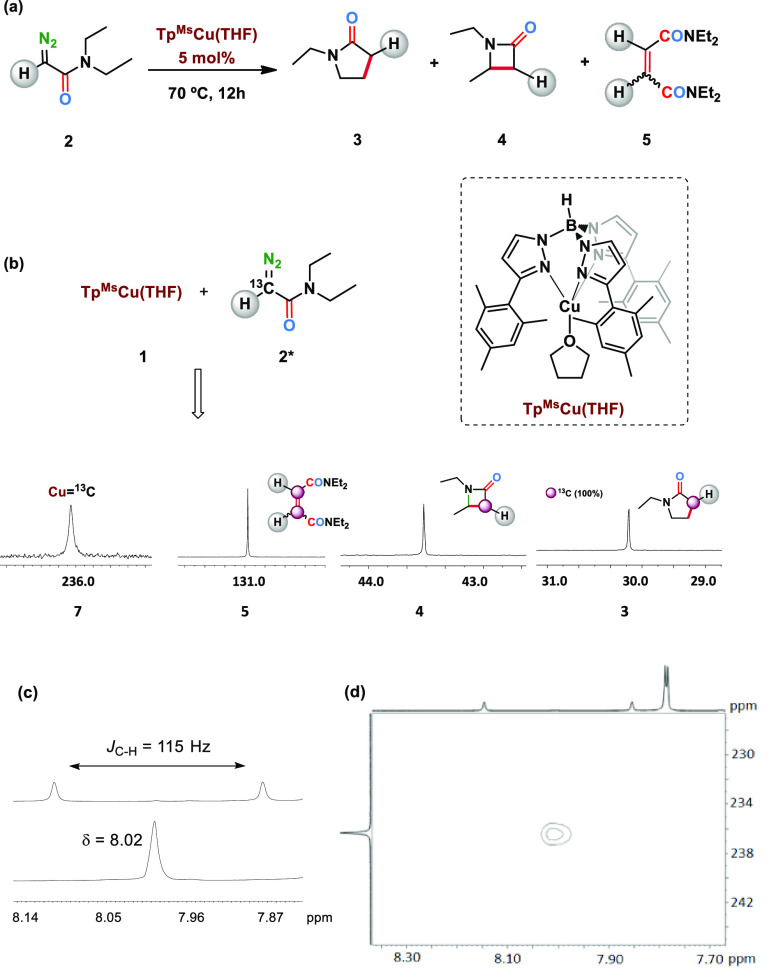
(a) Copper-Catalyzed Intramolecular C–H Bond Functionalization of *N*–*N*-Diethyldiazoacetamide; (b) ^13^C{^1^H} NMR Spectrum of the Reaction of Copper Complex **1** and ^13^C-Labeled Diazoacetamide (2*)

Our group has been involved in the area of carbene transfer from diazo compounds, with emphasis on its application to the functionalization of C–H bonds of unmodified alkanes,^6,19^ for which the design of very active catalysts precluded the observation of intermediates. However, we recently detected copper-carbene species^20^ in solution upon reacting Tp^Ms^Cu(THF) (**1**) and ethyl phenyldiazoacetate (PheDA), which were stable at temperatures below 10 °C. After those findings, we aimed to detect copper-carbene intermediates with monosubstituted diazo compounds, which yet remains a challenge, particularly with the most popular catalysts within this field, i.e., rhodium and copper, for which only disubstituted carbene species have been detected or isolated.^11,13,21^ Toward that end we chose Tp^Ms^Cu(THF)^22^ as the copper source, since the Tp^Ms^ ligand provides a high degree of steric protection to the metal center and, when formed, to the carbene ligand. Regarding the diazo reagent, we selected *N,N*-diethyl diazoacetamide (**2**), in view of the previously reported^23^ intramolecular carbene insertion into the C–H bonds of the ethyl N-substituents in the presence of **1** as the catalyst (Scheme 2a), leading to mixtures of lactams **3** and **4**. Olefin **5** was also observed from the catalytic reaction of two molecules of **2**.^24^ Since heating at 70 °C was needed to reach efficient ratios, we reasoned that perhaps the copper-carbene intermediate expected from the reaction of **1** and **2**, Tp^Ms^Cu=C(H)(CONEt_2_) (**6**), could be stable enough at room temperature to be detected. Based on this idea, we started this study carrying out the reaction of Tp^Ms^Cu(THF) (**1**) with 5 equiv of *N*,*N*-diethyl diazoacetamide (2*), 100% enriched in ^13^C at the ^13^C=N_2_ site, in toluene-d_8_ (Scheme 2b). Monitoring of the reaction by ^13^C{^1^H} NMR spectroscopy showed the appearance of a resonance centered at 236.6 ppm. This is within the typical region of Cu=*C* moieties reported by Hofmann^25^ for the complexes [^*t*^Bu_2_P(NSiMe_3_)_2_-κ_2_N]Cu=C(Ar)C(O)R) (235.8–219.0 ppm), by Warren^13^ for [β-diketiminate]Cu=CPh_2_ (253.1 ppm), or by our group for complexes Tp^*x*^Cu=C(Ph)(CO_2_Et) (Tp^x^ = Tp^iPr2^, Tp*, Tp^Ms^; 233.9, 236.8, and 248.5 ppm, respectively).^20^ Under these conditions, the resonances for **3**, **4**, and **5** were also observed, the latter being the major product. The reaction was immediate, and no free diazo compound 2* was observed after 5 min. Successive registration of ^13^C{^1^H} NMR spectra for hours showed no change in the composition of the mixture.

In view of the apparent stability of the species bearing the Cu=C unit, we repeated the reaction at a bench scale (see Supporting Information (SI) for optimization conditions). When 0.45 g of **1** were reacted with 5 equiv of diazo **2** in toluene at room temperature for 1 h, a yellowish solution was formed from which, after workup, the new compound **7** was isolated as crystalline material in 35% yield. The ^1^H NMR spectrum of **7** showed three equivalent pyrazolyl rings, two inequivalent ethyl groups, and a singlet at 8.02 ppm (Scheme 2c), which corresponds to one proton that correlates in the 2D-HSQC experiment with the aforementioned signal at 236.6 ppm (Scheme 2d). In the sample derived from the ^13^C-labeled diazo compound, the singlet at 8.02 in the ^1^H NMR spectrum split into a doublet with *J*_C–H_ = 115 Hz (Scheme 2c).

**Figure 1:**
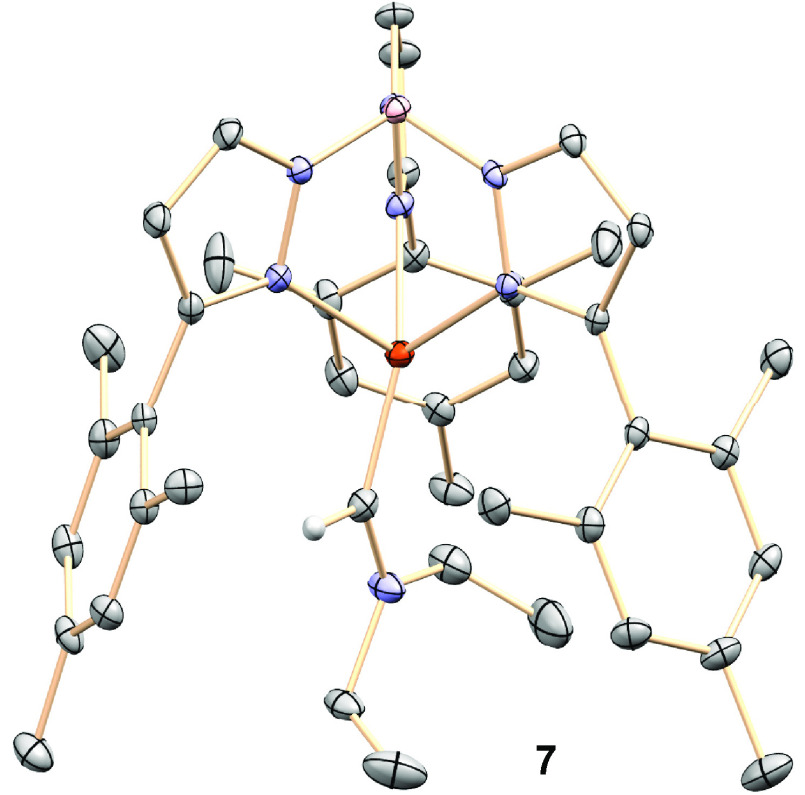
Molecular structure of the molecules for complex **7**. Hydrogens have been omitted for clarity.

Albeit the above data could support the assignment of complex **7** as the pursued Tp^Ms^Cu=C(H)(CONEt_2_), some other experimental data were not in agreement with that proposal: neither the FT-IR spectrum showed the expected absorption for the CO group nor the ^13^C{^1^H} NMR spectrum displayed any resonance within the carbonyl region. We suspected that a decarbonylation process could have occurred, an idea that was confirmed when single crystals of this complex were grown, and the molecular structure determined by X-ray studies showed the formulation Tp^Ms^Cu=C(H)(NEt_2_) (**7**).^26^ As shown in Figure 1, this complex contains the Tp^Ms^ ligand bonded to copper in a κ^3^-fashion, and a carbene ligand with two substituents: a hydrogen and a diethylamido group, the latter resulting from the loss of the CO present in the initial diazo compound. The distance Cu1–C1, 1858(5) Å, is similar to that reported by Warren for [β-diketiminate]Cu=CPh_2_ (1.834(3) Å).^13^ Complex **7** constitutes the first example of a metal-carbene complex formed from a diazo compound in which the CR^1^R^2^ moiety in the latter is different from that in the former. Additionally, it is also the first example of a monosubstituted copper carbene complex.^27^

**Scheme 3:**
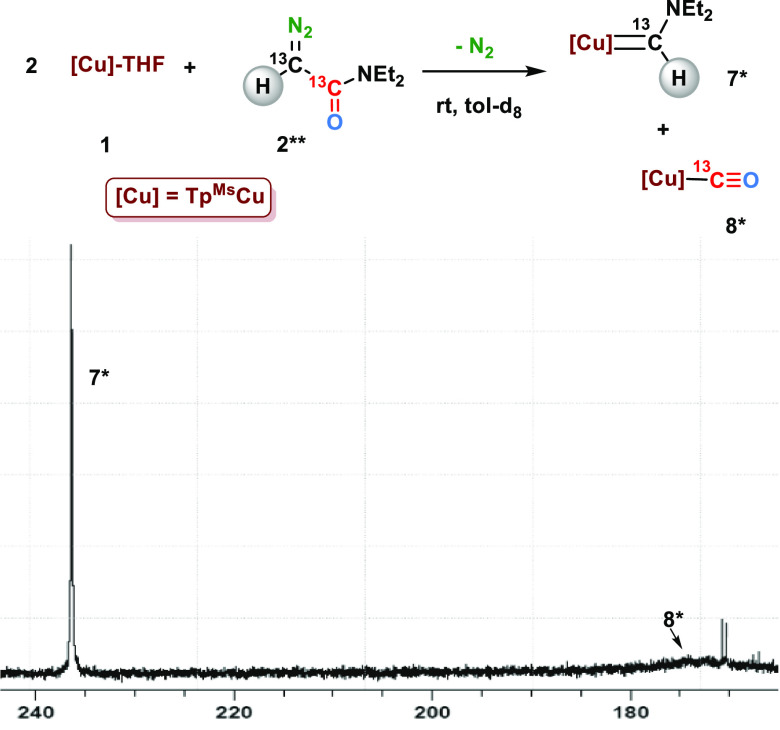
Reaction of Tp^Ms^Cu(THF) with Doubly Isotopically Labeled 2** and Region of the ^13^C{^1^H} NMR Spectrum Showing the Labeled Carbene and Carbonyl Groups of 7* and 8*

Further investigation of the ^1^H NMR spectrum of the experiment carried out with a 1:5 ratio (excess of diazo is employed since C–H bond insertion also occurs in a catalytic manner to some extent) of **1** and 2* showed that, in addition to **7**, another Tp^Ms^Cu-containing species was formed, both accounting for all detectable Tp^Ms^Cu cores. Such compound has been identified as Tp^Ms^Cu(CO) (**8**) (Scheme 3), as the result of the trapping of carbon monoxide by **1**.^22^ To gain further information, we have prepared the doubly isotopically enriched N_2_=^13^C(H)(^13^CONEt_2_) (2**) diazo compound and monitored its reaction with complex **1**, observing the resonances of carbene and carbonyl ligands of **7** and **8**, respectively (see Supporting Information also). Thus, this observation unambiguously demonstrates the existence of a decarbonylation process.

**Figure 2:**
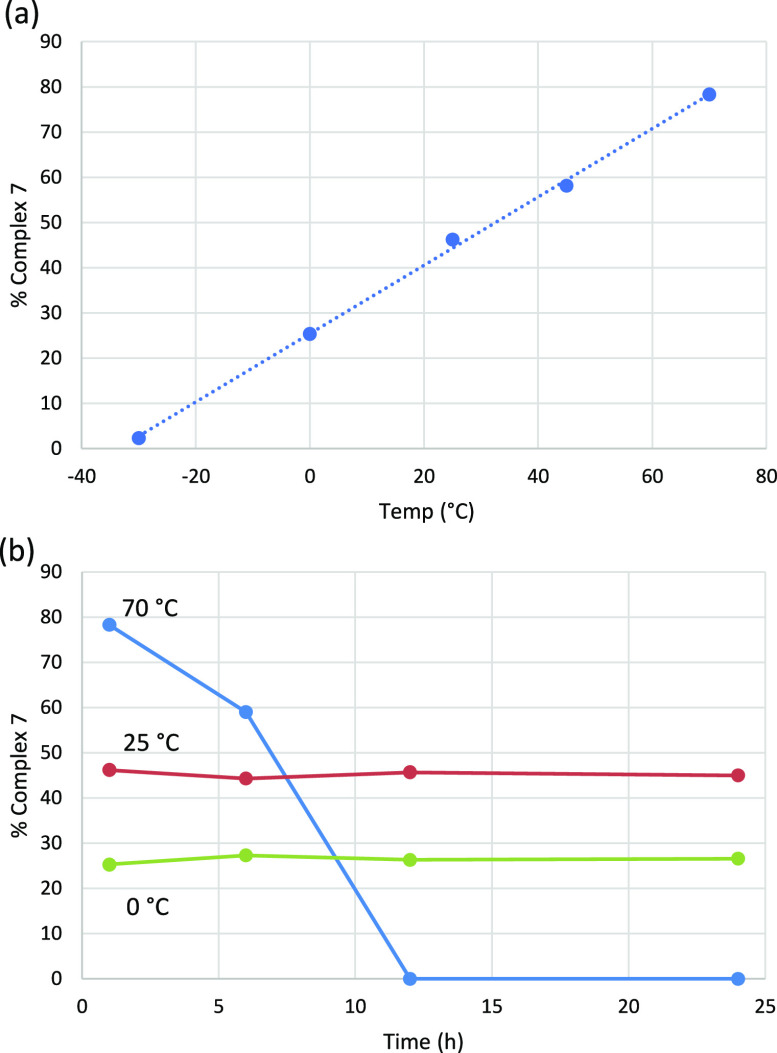
(a) Plot of the yields into complex **7** vs temperature (°C). (b) Variation of the yield of complex **7** with time at different temperatures.

The yield in complex **7** is dramatically affected by temperature, as shown in Figure 2a. The reaction of **1** and **2** (1:5 ratio) has been performed within the −30 to +70 °C range, showing an increase in the reaction yield from 2% to 78%, respectively, in 1 h time experiments. The high yield preparation and isolation of **7** is better performed at 70 °C (see Supporting Information). We have also monitored the concentration of complex **7** with time at three different temperatures (Figure 2b) and have found that at 0 or 25 °C such concentration remains constant after 24 h. However, at 70 °C, a process involving the disappearance of **7** takes place with time in such a way that after 12 h it cannot be detected in solution.

The reactivity of isolated copper carbene **7** has also been investigated. On one hand, no reaction was observed with styrene, *tert*-butanol, benzene, or cyclohexane from room temperature to 70 °C. It seems clear that the loss of the CO group modifies the electronic behavior of the carbene C(H)NEt_2_ ligand decreasing the electrophilicity from Tp^Ms^Cu=C(H)(CONEt_2_) (**6**) to Tp^Ms^Cu=C(H)NEt_2_ (**7**). Interestingly, heating toluene solutions of isolated **7** at 70 °C for hours/days did not lead to any transformation, whereas the same experiment under a CO atmosphere (4 bar) for 6 h induced a clean conversion of **7** into Tp^Ms^Cu(CO) (**8**). NMR studies have also shown that the olefin (Et_2_N)(H)C=C(H)(NEt_2_) (**9**) derived from the coupling of two of the carbene units in **7** is formed.This result agrees with the lack of observation of copper-carbene **7** in the reaction of **1** and **2** at 70 °C for several hours, because of the presence of CO from the decarbonylation process.

**Scheme 4:**
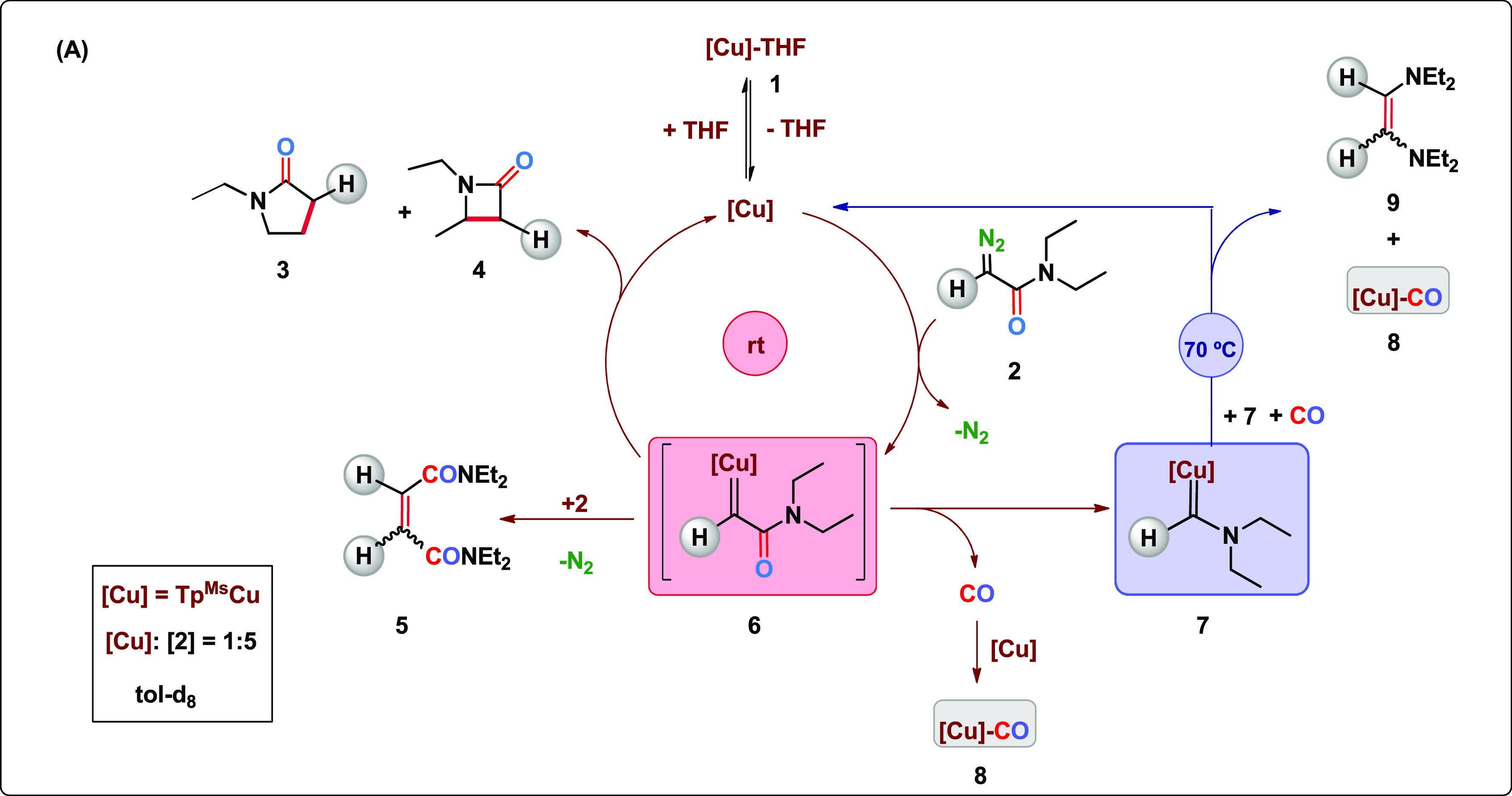
General Picture of the Reactivity Derived from the Interaction of Tp^Ms^Cu(THF) (**1**) and N_2_=C(H)CONEt_2_ (**2**)

The reactivity observed from the initial mixture of **1** and **2** (1:5 ratio) is rationalized in Scheme 4. The Tp^Ms^Cu core is generated upon decoordination of THF and reacts with the diazo compound **2** to generate the transient copper carbene **6**. This species undergoes either the intermolecular reaction with a second molecule of **2** or the intramolecular insertion of the carbene group into the secondary or primary C–H bonds of the ethyl groups of the NEt_2_ fragment, thus yielding lactams **3** and **4**, these catalytic cycles taking place at room temperature. Intermediate **6** can alternatively undergo a decarbonylation process which generates the isolable copper carbene **7**, a process which occurs, at least, within the −30 to +70 °C interval. Evolved CO can be trapped by Tp^Ms^Cu cores leading to the formation of Tp^Ms^Cu(CO) (**8**). At 70 °C, copper-carbene **7** originates olefin **9** and carbonyl **8** in a process requiring carbon monoxide. This proposal agrees with the initial observation of the need for heating at 70 °C to induce catalysis by **1**,^23^ since the copper–carbonyl **8** under the reaction conditions in the presence of **2** also generates Tp^Ms^Cu cores to catalyze the C–H bond functionalization reaction.

**Scheme 5:**
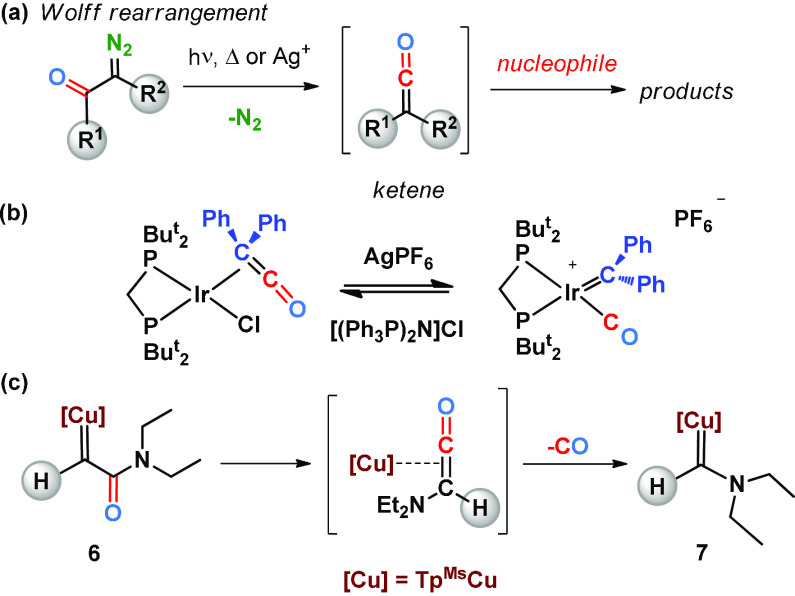
(a) Wolff Rearrangement of Diazo Compounds; (b) Reversible Formation of Ketenes from Carbene and CO Ligands Described by Grotjahn; (c) Plausible Intermediate for the Conversion of **6** into **7**

The transformation of **6** into **7** somehow resembles the Wolff rearrangement of diazo compounds (Scheme 5a), which originate short-lived ketenes^4,28^ which further react with nucleophiles, in a process triggered by light, heating, or silver(I) salts. Grotjahn and co-workers described a ketene iridium complex which undergoes the reversible conversion of the coordinated ketene into a metallocarbene which contains a CO ligand (Scheme 5b).^29^ Based on these precedents, it could be possible that copper-carbene **6** undergoes a related rearrangement in the coordination sphere of copper, followed by CO extrusion (Scheme 5c). As mentioned above, the reverse reaction is not observed in our case, at least apparently, as inferred from the experiment carried out with **7** and CO.

### DFT Studies and Microkinetic Model

**Figure 3:**
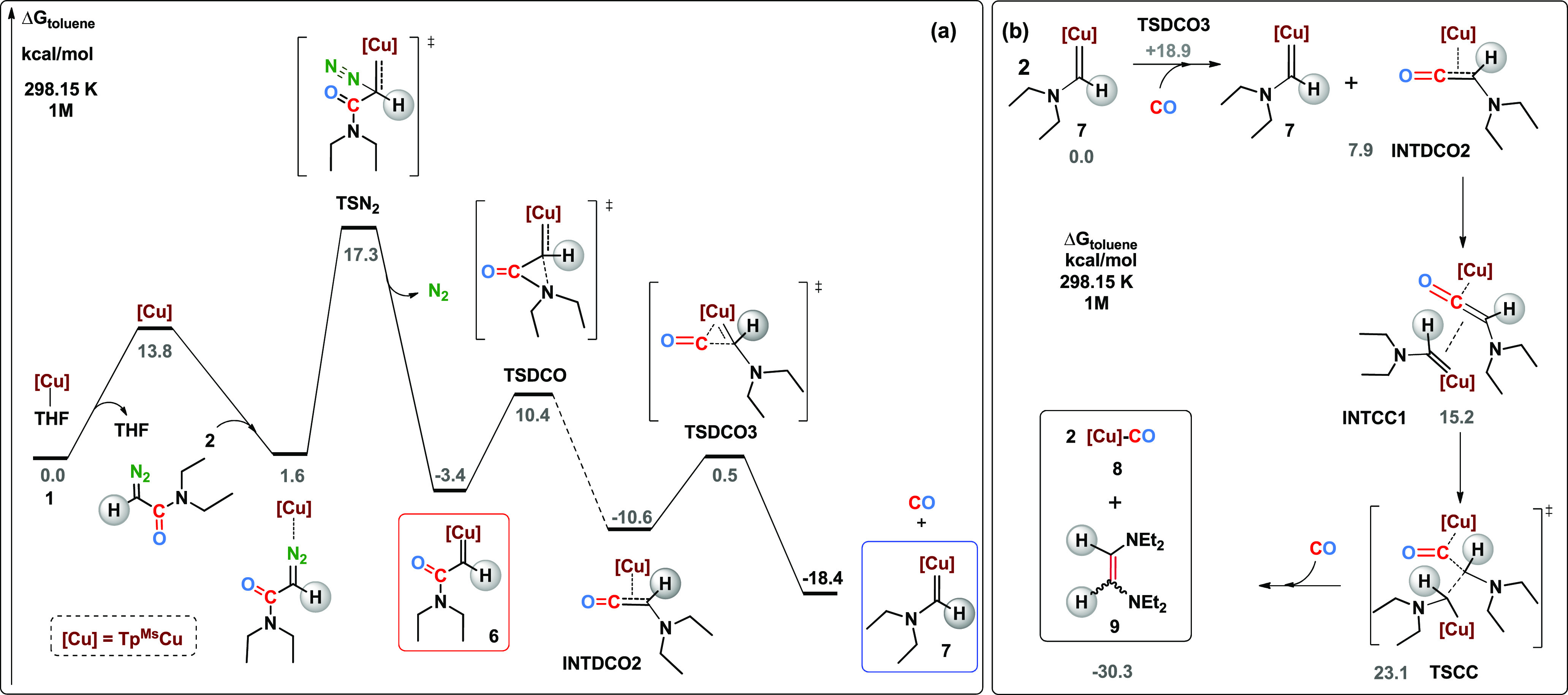
(a) Simplified reaction free energy profile from DFT calculations for the generation of copper carbenes **6** and **7**. (b) Simplified reaction mechanisms for the formation of olefin **9**.

Given the unexpected formation of **7**, we further explored the process with DFT studies (B3LYP-D3 optimizations in solution; see details in the Supporting Information) and microkinetic simulations. Computational results are summarized in Figure 3, showing the free energy profile for the conversion from the starting Tp^Ms^Cu(THF) complex (**1**) to compound **7**. The initial steps follow the usual mechanism up to the formation of metal-carbene **6**, with the highest barrier corresponding to nitrogen extrusion (17.3 kcal/mol^–1^). Species such as metal-carbene **6** have been previously shown to be very reactive toward homocoupling or C–H activation.^19,23,24^ The novelty in this system is the availability of an alternative low energy path via decarbonylation. The latter proceeds through a rather stable ketene intermediate **INTDCO2**, with a linear arrangement between the CO and the former carbene carbon (see Supporting Information for more details). The barrier for the decarbonylation process is low at 298.15 K (13.8 kcal/mol) while the reverse barrier is 28.0 kcal·mol^–1^, too high to take place significantly at ambient temperature. At 343.15 K the profile is similar, with a barrier of 13.9 kcal/mol for decarbonylation and a barrier of 30.3 kcal/mol for the reverse process. The lack of observation of the reverse pathway when reacting complex **7** and CO at 70 °C is due to the more favorable path of formation of olefin **9** and carbonyl adduct **8** (see below). Further calculations were carried out for the case where the toluene solvent is replaced by dichloromethane, also reproducing the experimental observation of pyrrolidinone (**3**) and azetidinone (**4**). The detailed computational results for these cases are given in the Supporting Information.

The formation of **9** has also been investigated. Several paths previously reported for such carbene coupling have been discarded based on their high energy barriers: (a) direct interaction of two molecules of **7**;^24^ (b) carbene C(H)NEt_2_ dissociation and reaction with **7**;^24^ and (c) coupling of two dissociated carbene units^30^ (see Supporting Information). At variance with that, we have found an accessible route which involves two molecules of **7** and one of CO. The simplified mechanism for the formation of olefin **9** is depicted in Figure 3b. The mechanism starts with one molecule of the metal-carbene **7** adding a molecule of CO to form the above-mentioned intermediate **INTDCO2** which further reacts with a second molecule of **7**. The formation of the olefin **9** must overcome a barrier of 23.1 kcal/mol. Thus, albeit olefin **9** is more stable than metal-carbene **7**, its formation involves a higher barrier (see Supporting Information for further details).

**Figure 4:**
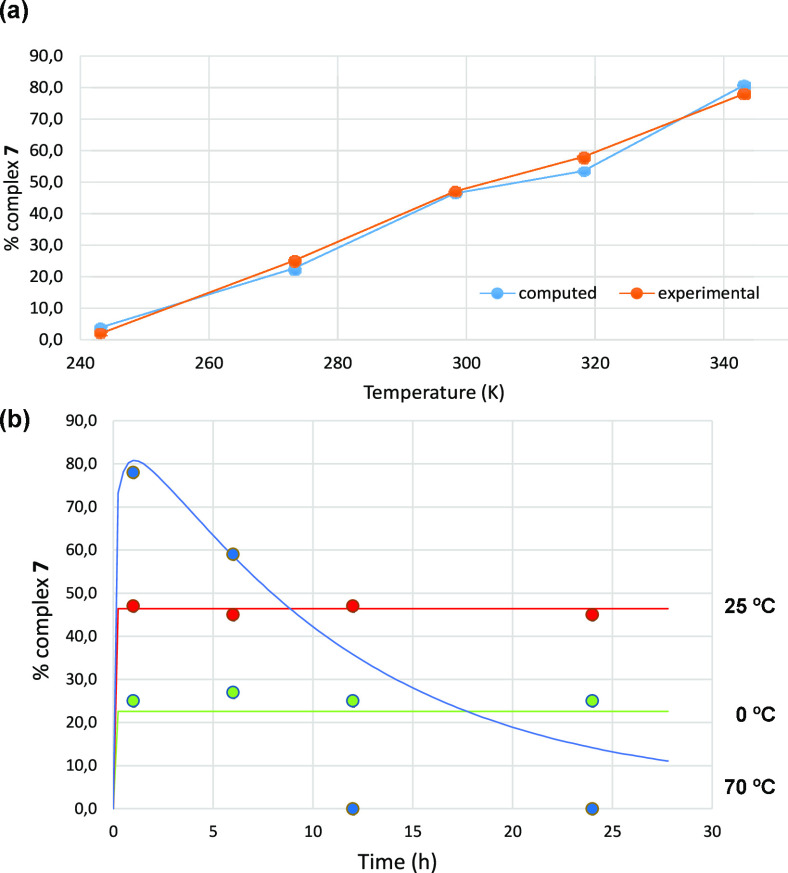
(a) Computed yield (%) of metallocarbene **7** at 1 h of reaction for different reaction temperatures (K). (b) Evolution over time (h) of the yield (%) of metallocarbene **7** at different temperatures (continuous line obtained from DFT and microkinetic simulations; dots obtained from experiment).

The third possible product from the interaction between the precursor **1** and *N,N*-diethyl diazoacetamide is olefin **5**, which is also experimentally observed. We also computed the mechanism of its formation from **6**, which is detailed in the Supporting Information. The key result is that the free energy barrier for C=C bond formation leading to **5** is 8.9 kcal/mol. This value is significantly lower than the 13.8 kcal/mol reported above for decarbonylation, which from a simplistic view would indicate that decarbonylation should not take place. However, for convoluted mechanisms such as the one present here, it is better to translate the computed free energy barriers to rate constants and use those to estimate reaction times through microkinetic modeling.^31^ We performed such microkinetic modeling with additional energy adjustment^32^ (see Supporting Information for details), and we obtained the results shown in Figure 4. The agreement between calculations and experimental data (displayed in Figure 2) is certainly remarkable, and this strongly supports the validity of our mechanistic proposal.

## Conclusion

We herein report the first example of the modification of a carbene group during its metal-catalyzed transfer from a diazo functionality, a discovery that should result in further consideration from the community involved in this area. From now on, the belief that the carbene moiety remains undisturbed in this type of processes must be reconsidered when analyzing reaction outcomes, particularly in those catalytic systems which either seem deactivated or require anomalous heating. Additionally, the chemistry of these isolated metal-carbenes is yet to be developed and could find important applications as surrogates of well-known midtransition metal Fischer carbene complexes

## Experimental Section

### Synthesis and Characterization of Complexes **7** and 7*

In a 250 mL Schlenk flask, Tp^Ms^Cu(THF) (**1**, 0.450 g, 0.64 mmol) was dissolved in 75 mL of toluene, and a solution of 2-diazo-*N,N*-diethylacetamide (**2**, 0.451 g, 3.2 mmol) in the same solvent (37 mL) was added via canula. The mixture was stirred at 70 °C for 1 h before the volatiles were removed under reduced pressure. The residue was washed with dry acetone (5 mL) and cold Et_2_O (3 × 5 mL) and dried under vacuum, to afford 0.35 g of a yellow solid corresponding, according to NMR studies, to a mixture of complexes **7** and **8** (yields of 47% and 10% referred to initial **1**). Crystallization from a mixture of toluene/hexane (4:4 mL) at −30 °C led to the isolation of complex **7** as yellow crystals in 35% yield (combined crops). Analytically calculated for C_41_H_51_BCuN_7_ (**1**): C, 68.75; H, 7.18; N, 13.69%. Found: C, 69.41; H, 7.12; N, 13.66%. Complex 7* was prepared following the same procedure, with the corresponding labeled diazo compound (see SI) with an isolated yield of 28%.

^1^H NMR (C_6_D_6_, 500 MHz): δ 0.28 (t, *J*_HH_ = 7.3 Hz, 3H, NCH_2_C*H*_*3*_), 0.44 (t, *J*_HH_ = 7.3 Hz, 3H, NCH_2_C*H*_*3*_), 2.12 (s, 27H, C*H*_*3*_), 2.23 (q, *J*_HH_ = 7.3 Hz, 2H, NC*H*_*2*_CH_3_), 2.41 (q, *J*_HH_ = 7.3 Hz, 2H, NC*H*_*2*_CH_3_), 6.01 (d, *J*_HH_ = 2.0 Hz, 3H, C*H*), 6.71 (s, 6H, C*H*), 7.80 (d, *J*_HH_ = 2.0 Hz, 3H, C*H*), 8.02 (s, 1H, Cu=C*H*). ^13^C{^1^H} NMR (C_6_D_6_, 125 MHz): δ 12.9 (*C*H_3_), 13.6 (*C*H_3_), 20.9 (*C*H_3,Ms_), 21.1 (*C*H_3,Ms_), 53.1 (N*C*H_2_), 54.9 (N*C*H_2_), 104.5 (*C*H_pz_), 127.8 (*C*H_Ms_), 133.6 (*C*_q,Ms_), 134.9 (*C*H_pz_), 136.2 (*C*_q,Ms_), 138.3 (*C*_q,Ms_), 150.9 (C_q,pz_), 236.6 (Cu=*C*).

## Supporting Information Available

All procedures and characterization data, computational data, and Cartesian coordinates of the optimized structures (PDF)

## Accession Codes

CCDC 2057177 contains the supplementary crystallographic data for this paper. These data can be obtained free of charge via www.ccdc.cam.ac.uk/data_request/cif, or by emailing data_request@ccdc.cam.ac.uk, or by contacting The Cambridge Crystallographic Data Centre, 12 Union Road, Cambridge CB2 1EZ, UK; fax: +44 1223 336033.
