# Supplementary material for: Two Copper-Carbenes from One Diazo Compound
Source: J Am Chem Soc. 2021 Mar 18;143(12):4837–43. doi: 10.1021/jacs.1c01483 (PMC8603358; doi:10.1021/jacs.1c01483)
Supplement: Supplementary file 1 — ja1c01483_si_001.pdf [file ja1c01483_si_001.pdf]

SUPPORTING INFORMATION for

Two Copper-Carbenes from One Diazo Compound

María Álvarez,[†] Maria Besora,[‡,§] Francisco Molina,[†] Feliu Maseras,[*,‡,‖] Tomás R. Belderrain,[*,†] Pedro J. Pérez[*,†]

[†]Laboratorio de Catálisis Homogénea, Unidad Asociada al CSIC, CIQSO-Centro de Investigación en Química Sostenible and Departamento de Química, Universidad de Huelva, 21007 Huelva, Spain.

[‡]Institute of Chemical Research of Catalonia, ICIQ, Av. Països Catalans, 16, Barcelona Institut of Science and Technology, 43007 Tarragona, Spain.

[§]Departament de Química Física i Inorgànica, Universitat Rovira i Virgili, 43007 Tarragona, Spain.

[‖]Departament de Química, Universitat Autònoma de Barcelona, 08193 Bellaterra, Spain

Table of Contents

1. General Considerations **S3**
2. Synthesis and characterization of labelled 2-diazo-*N,N*-diethyl acetamides (**2*** or **2****) **S3**
3. Synthesis and characterization of complexes **7** and **7*** **S6**
4. NMR studies of the reaction of **1** with diazo compounds **2***and **2****.** **S11**
5. Factors affecting the synthetic procedure of **7** **S14**
6. Computational Methods **S15**

1. General considerations

All air and moisture-sensitive manipulations were carried out using standard high vacuum lines and standard Schlenk techniques under inert atmosphere of a nitrogen or in a glovebox under an atmosphere of purified nitrogen. All reactants were purchased from Sigma-Aldrich or Across and used without further purification. Solvents were dried using a SPS-MBraun system, with the exception of toluene which was dried with sodium and separated by distillation under nitrogen.

The compounds $Tp^{Ms}Cu(THF)$, $Tp^{Ms}Cu(CO)$ and *N,N*-diethyl diazoacetamide were prepared according to literature procedures.[1,2] NMR spectra were recorded on Agilent 400 MR or Agilent 500 DD2 spectrometers, ^{1}H and ^{13}C NMR shifts are reported relative to tetramethylsilane. FT-IR spectra were collected on a Nicolet IR200 FTIR spectrometer. Elemental analyses were performed on a Perkin-Elmer Series II CHNS/O Analyzer 2400. Crystal structure determinations were carried out using a BRUKER D8 FIXED-CHI diffractometer equipped with an Oxford Cryosystems low-temperature device.

2. Synthesis and characterization of labelled *2*-diazo-*N,N*-diethyl acetamides (2* or 2**)

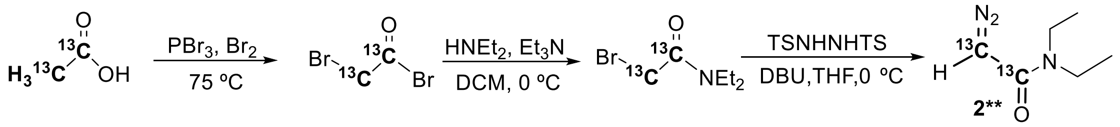

Both 2-diazo-*N,N*-diethyl acetamide **2*** and **2**** were prepared in the same manner, following the above reaction sequence. In a three-necked flask equipped with a reflux condenser, acetic acid-$^{13}C_1$-$^{13}C_2$ (1g, 16.12 mmol) and PBr_3 (1.96 mL, 16.12 mmol) were added under nitrogen atmosphere. Then, elemental bromine (2 mL, 40.1 mmol) was added dropwise to the stirred solution. The resulting mixture was heated at 75 ° C for 3 h (after 1.5 h, additional 2 mL of Br_2 were added). After this time, the mixture was cooled to room temperature and transferred into a distillation device. Heating at 70 °C under reduced pressure provided the desired compound, which was redissolved in 20 mL of dry dichloromethane and cooled to 0 °C before diethylamine (4.72 mL, 47.2 mmol) was added dropwise. After stirring for 10 min, triethylamine (6.3 mL, 47.2 mmol) was slowly. After 2 h the mixture was allowed to slowly reach the room temperature. The reaction mixture was then washed with distilled water, and the aqueous phase was extracted with dichloromethane (3 x 20 mL). The organic phases were combined and washed with a 20 % aqueous $Na_2S_2O_3$ solution, brine and finally with distilled water. The organic phase was dried over anhydrous $MgSO_4$ and concentrated under reduced pressure.

---

1. Schneider, J. L.; Carrier, S. M.; Ruggiero, Jr. C. E.; Young, V. G.; Tolman, W. B. *J. Am. Chem. Soc.* **1998,** *120***,** 11408-11418.
2. Pisella, G.; Gagnebin, A.; Waser, J. *Org. Lett.* **2020**, *22*, 3884-3889.

The resulting 2-bromo-*N*,*N*-diethyl acetamide (0.78 g, 4 mmol), was used in the next step without purification, and *N,N'*-ditosylhydrazine (1.25 g, 4 mmol) was dissolved in dry THF (20 mL) and cooled down to 0 ˚C, before DBU (3 mL, 20 mmol) was added dropwise. The mixture was stirred at room temperature for 1 h and then quenched with a saturated solution of $NaHCO_3$ (2 x 50 mL) and extracted with diethyl ether (3 x 50 mL). The combined organic layers were dried over anhydrous $MgSO_4$. The solvent was removed under reduced pressure and purified by basic alumina column chromatography using diethyl ether as eluent, the corresponding 2-bromo-*N*,*N*-diethyl acetamides being obtained as yellow oils: **2***, 0.544 g, 3.8 mmol, 24 %; **2****, 0.370 g, 2.59 mmol, 16 %.

2*: ^{1}H NMR (400 MHz, $CDCl_3$): δ 1.15 (t, J_{HH} = 7.1 Hz, 6H, $2CH_2CH_3$), 3.26 (br, 4H, 2 CH_2CH_3), 4.93 (d, J_{CH} = 194 Hz, 1H, N_2CH). $^{13}C\{^1H\}$ NMR (100 MHz, $CDCl_3$): δ 46.4 ($N_2{}^{13}CH$).

2**: ^{1}H NMR (400 MHz, $CDCl_3$): δ 1.08 (t, J_{HH} = 7.1 Hz, 6H, $2CH_2CH_3$), 3.21 (br s, 4H, 2 CH_2CH_3), 4.8 (dd, J_{CH} = 3.7 Hz, 1H, N_2CH). $^{13}C\{^1H\}$ NMR (100 MHz, $CDCl_3$): 46.3 (d, J_{CC} = 82 Hz, $^{13}CH^{13}CO$) 164.7 (d, J_{CC} = 82 Hz, $^{13}CH^{13}CO$).

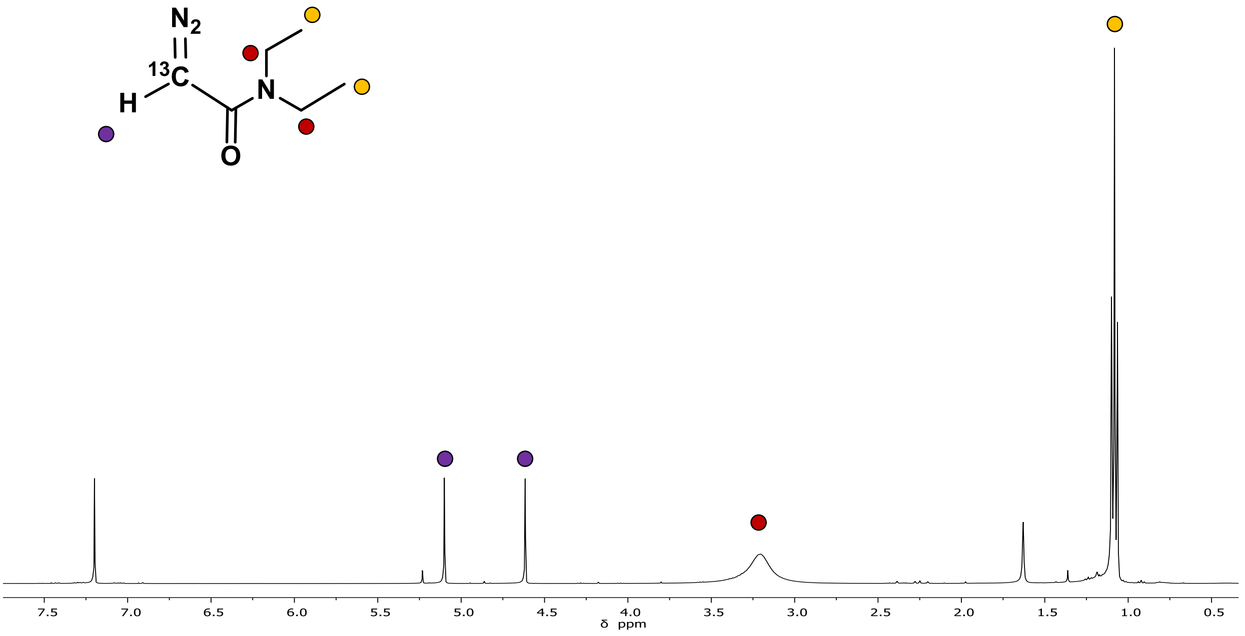

Figure S1**: ^{1}H NMR spectrum of **2 (400 MHz, $CDCl_3$).*

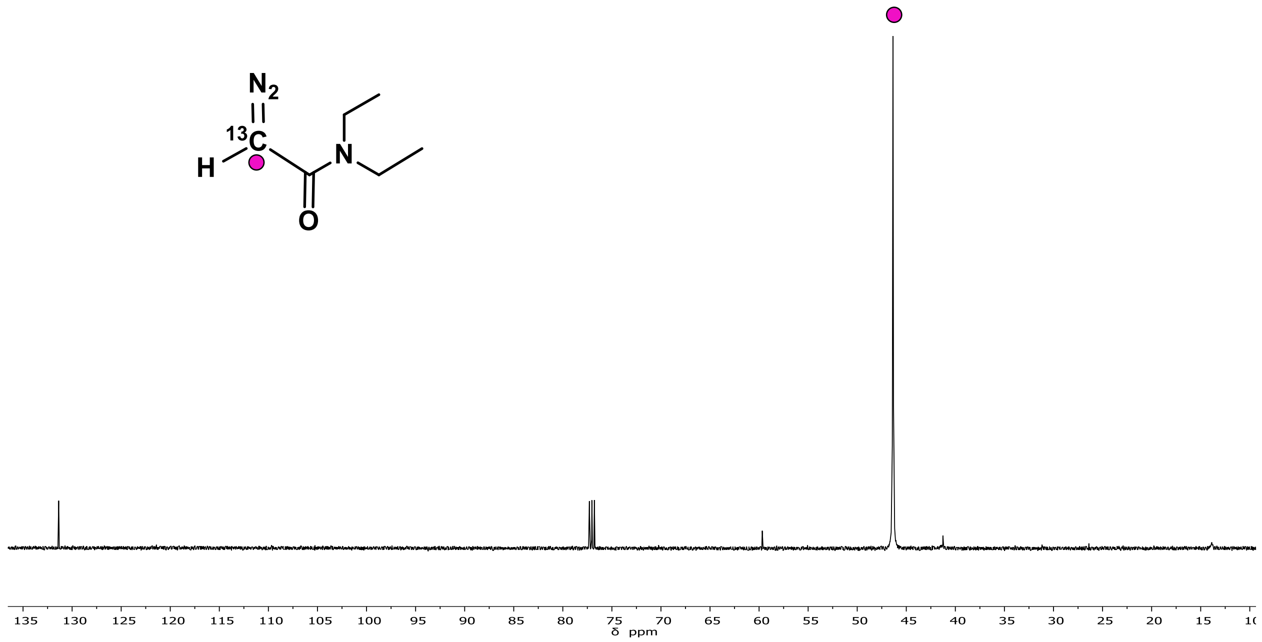

Figure S2: *$^{13}C\{^{1}H\}$ NMR spectrum of **2*** (100 MHz, $CDCl_3$).*

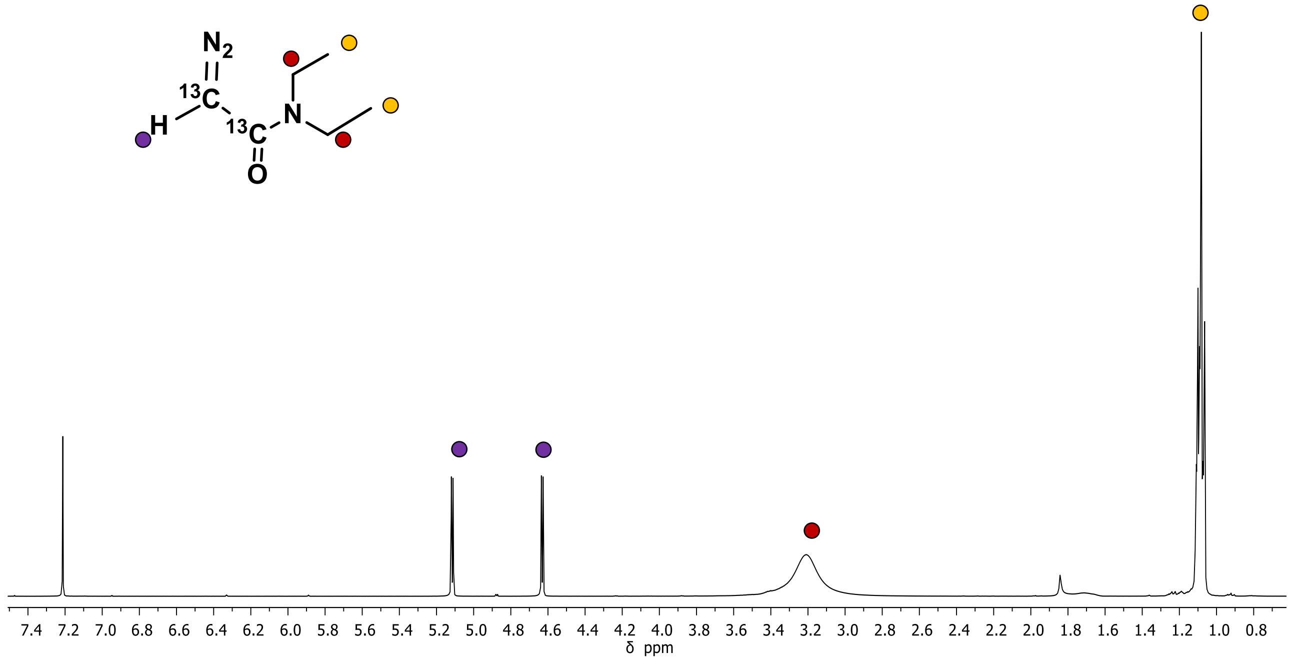

Figure S3: *^{1}H NMR spectrum of **2**** (400 MHz, $CDCl_3$).*

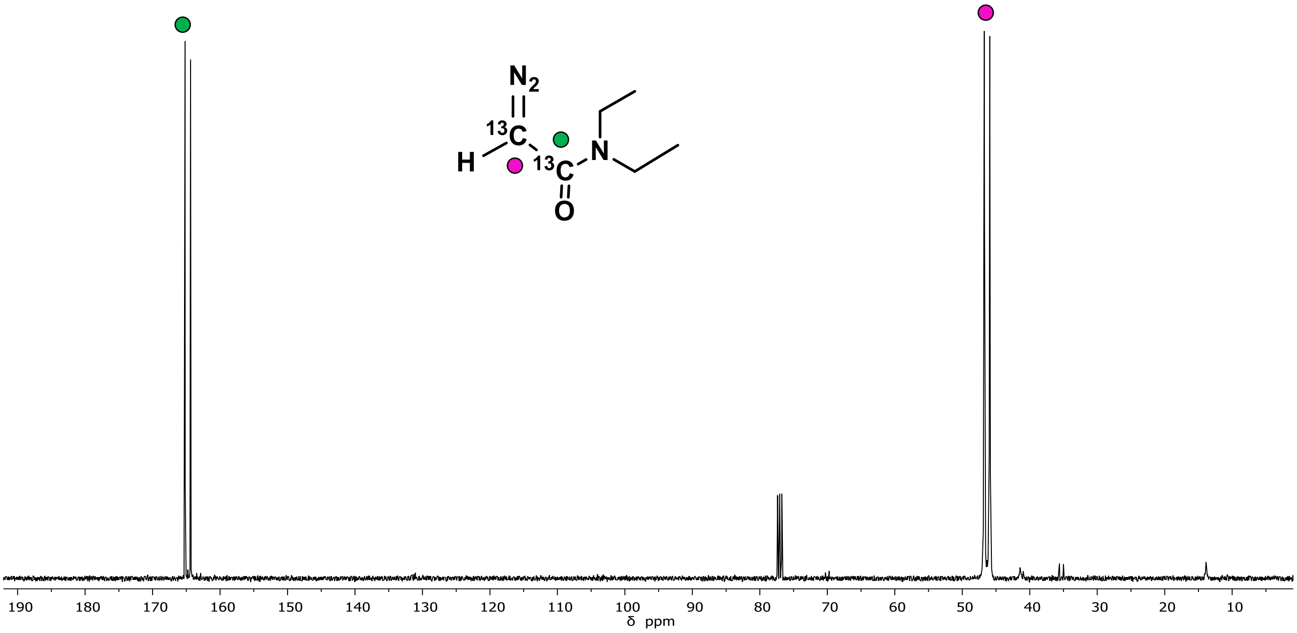

Figure S4**: $^{13}C\{^1H\}$ NMR spectrum of **2* (100 MHz, $CDCl_3$).*

3. Synthesis and characterization of complexes 7 and 7*

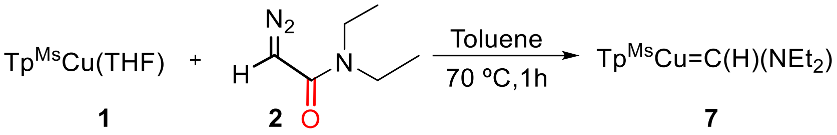

In a 250mL Schlenk flask, $Tp^{Ms}Cu(THF)$ (**1**, 0.450 g, 0.64 mmol) was dissolved in 75 mL of toluene, and a solution of 2-diazo-*N*,*N*-diethylacetamide (**2**, 0.451 g, 3.2 mmol) in the same solvent (37 mL) was added via canula. The mixture was stirred at 70 ºC for 1 h before the volatiles were removed under reduced pressure. The residue was washed with dry acetone (5 mL) and cold Et_2O (3 x 5 mL), and dried under vacuum, to afford 0.35 g of a yellow solid corresponding, according to NMR studies, to a mixture of complexes **7** and **8** (yields of 47% and 10% referred to initial **1**). Crystallization from a mixture of toluene:hexane (4:4 mL) at -30 ºC led to the isolation of complex **7** as yellow crystals in 35 % yield (combined crops). Analysis calculated of $C_{41}H_{51}BCuN_7$ (**1**): C, 68.75; H, 7.18; N, 13.69 %. Found: C, 69.41; H, 7.12; N, 13.66 %. Complex **7*** was prepared following the same procedure, with an isolated yield of 28 %.

Tp^{Ms}Cu=C(H)(NEt$_2$) (7): ^{1}H NMR (C_6D_6, 500 MHz): δ 0.28 (t, J_{HH} = 7.3 Hz, 3H, NCH$_2$C*H*$_3$), 0.44 (t, J_{HH} = 7.3 Hz, 3H, NCH$_2$C*H*$_3$), 2.12 (s, 27H, C*H*$_3$), 2.23 (q, J_{HH} = 7.3 Hz, 2H, NC*H*$_2$CH$_3$), 2.41 (q, J_{HH} = 7.3 Hz, 2H, NC*H*$_2$CH$_3$), 6.01 (d, J_{HH} = 2.0 Hz, 3H, C*H*), 6.71 (s, 6H, C*H*), 7.80 (d, J_{HH} = 2.0 Hz, 3H, C*H*), 8.02 (s, 1H, Cu=C*H*). ^{13}C{^{1}H} NMR (C_6D_6, 125 MHz): δ 12.9 (*C*H$_3$), 13.6 (*C*H$_3$), 20.9 (*C*H$_{3,Ms}$), 21.1 (*C*H$_{3,Ms}$), 53.1 (N*C*H$_2$), 54.9 (N*C*H$_2$), 104.5 (*C*H$_{pz}$), 127.8 (*C*H$_{Ms}$), 133.6 (*C*$_{q,Ms}$), 134.9 (*C*H$_{pz}$), 136.2 (*C*$_{q,Ms}$), 138.3 (*C*$_{q,Ms}$) , 150.9 (C$_{q,pz}$), 236.6 (Cu=*C*).

For **7***, the resonance at 8.02 ppm in the ^{1}H NMR spectrum split into a doublet with J_{H-C} = 115 Hz.

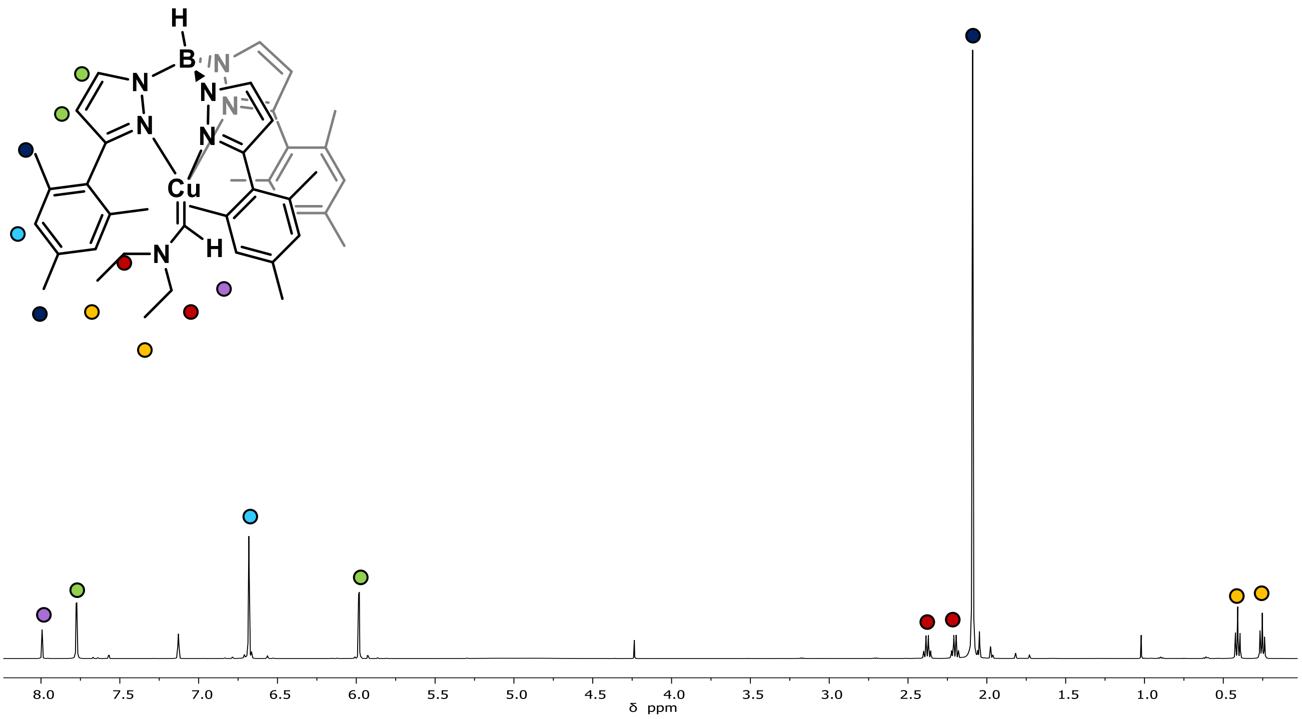

***Figure S5**: ^{1}H NMR spectrum of **7** (500 MHz, C_6D_6).*

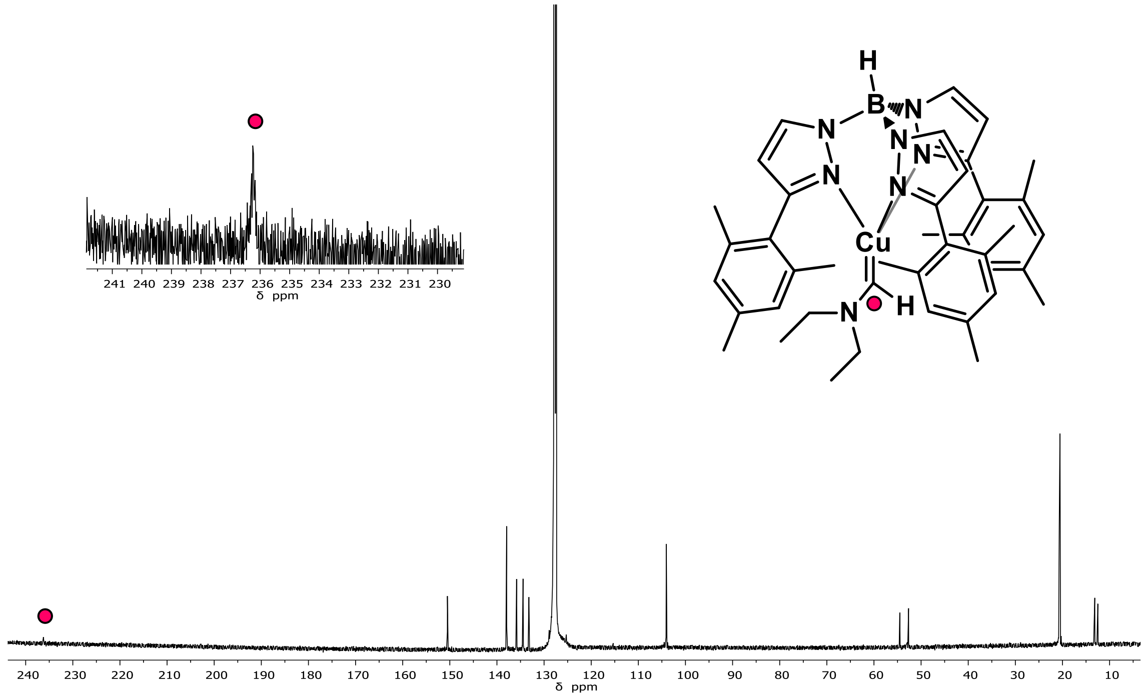

Figure S6: *$^{13}C\{^{1}H\}$ NMR spectrum of **7** (125 MHz, C_6D_6).*

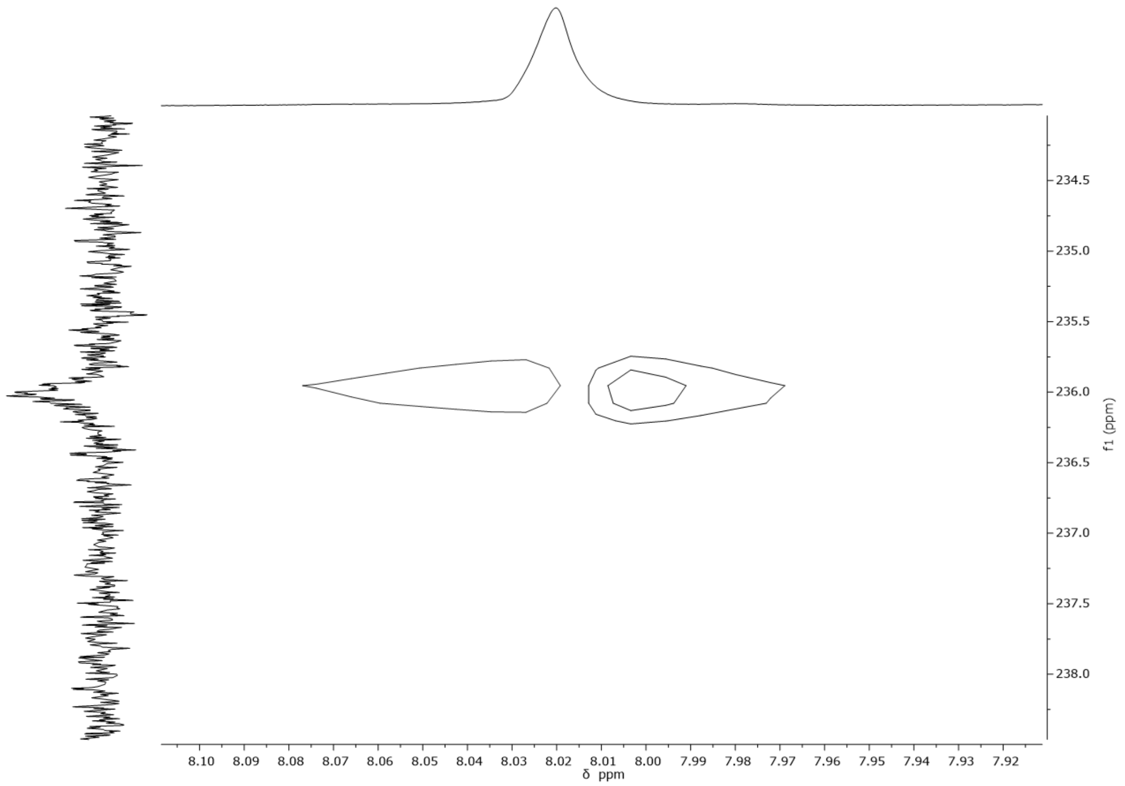

Figure S7: *HSQC NMR spectrum of **7** (500 MHz, C_6D_6).*

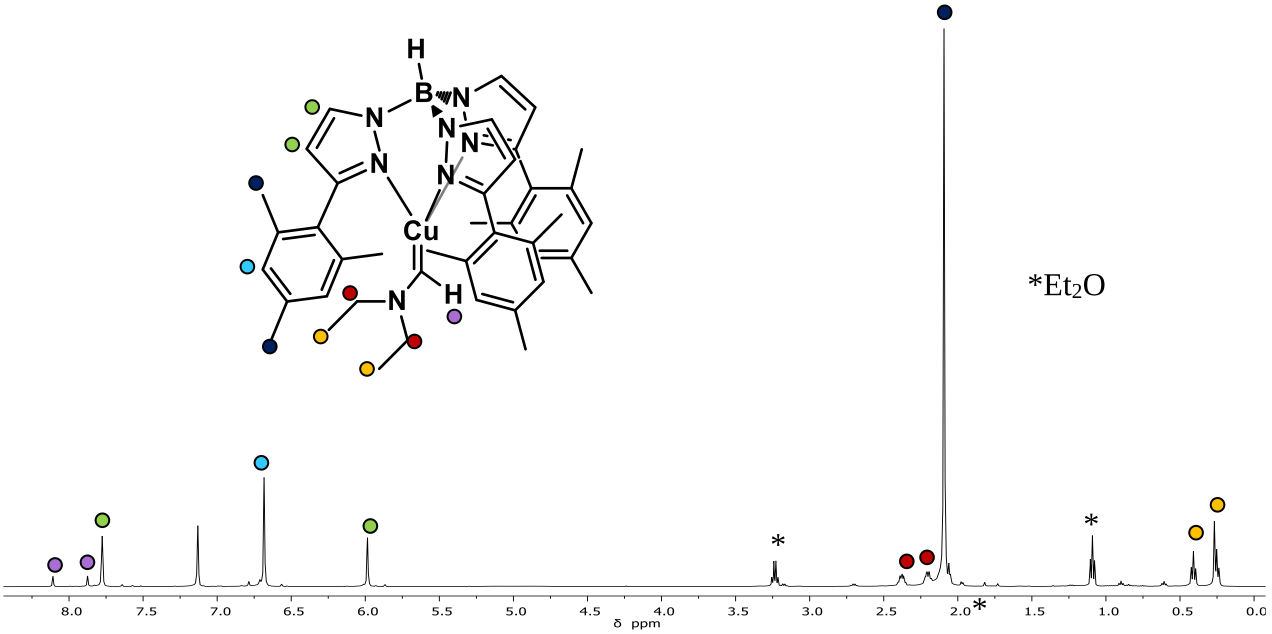

Figure S8: ^{1}H NMR spectrum of ***7**** (500 MHz, C_6D_6).

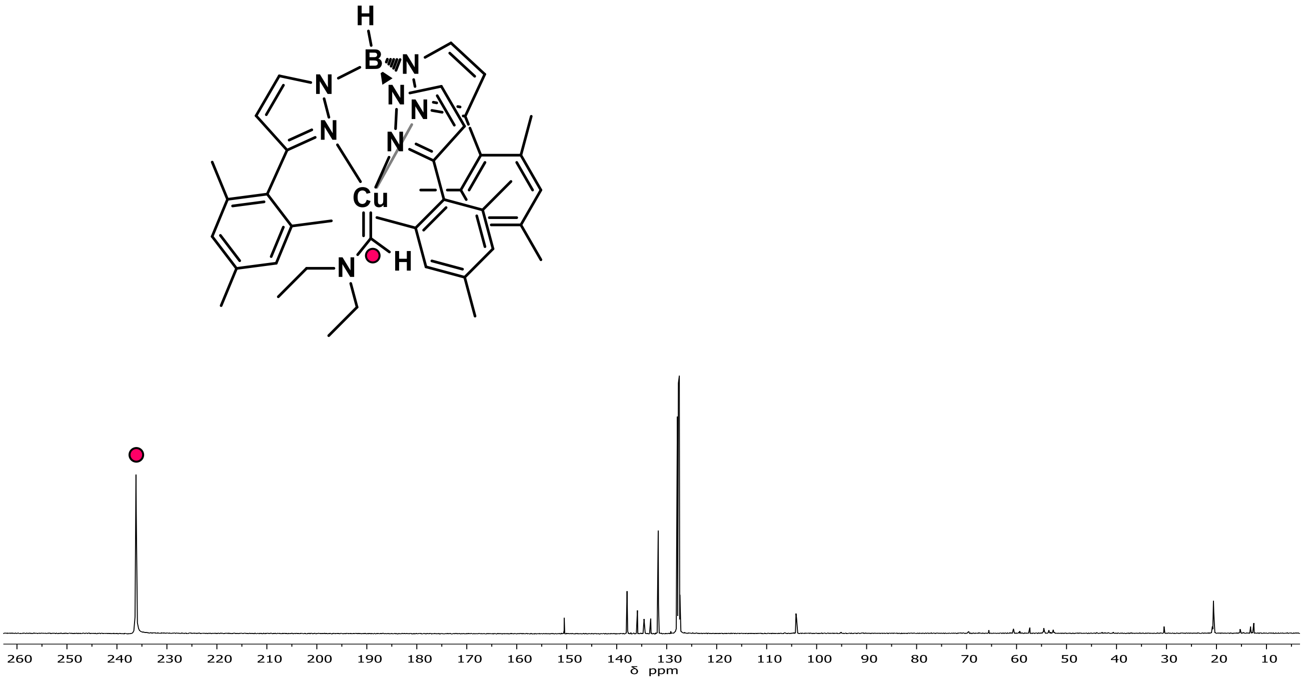

Figure S9: ^{13}C{^{1}H} NMR spectrum of ***7**** (125 MHz, C_6D_6).

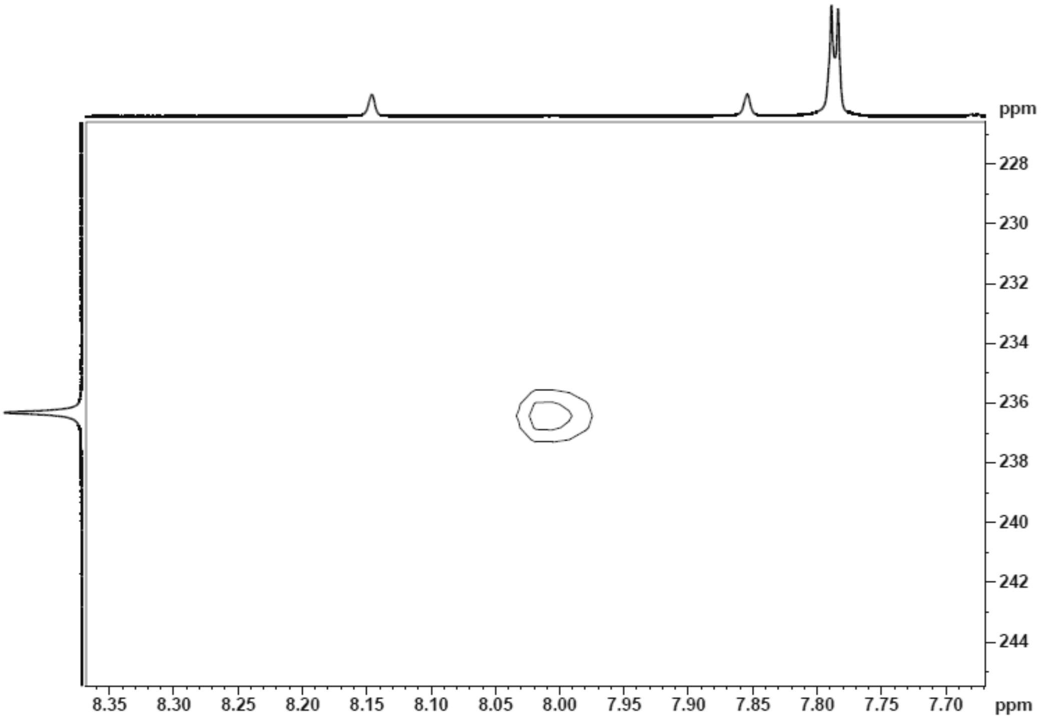

Figure S10: *HSQC NMR spectrum of complex* ***7****. (500 MHz, C_6D_6)

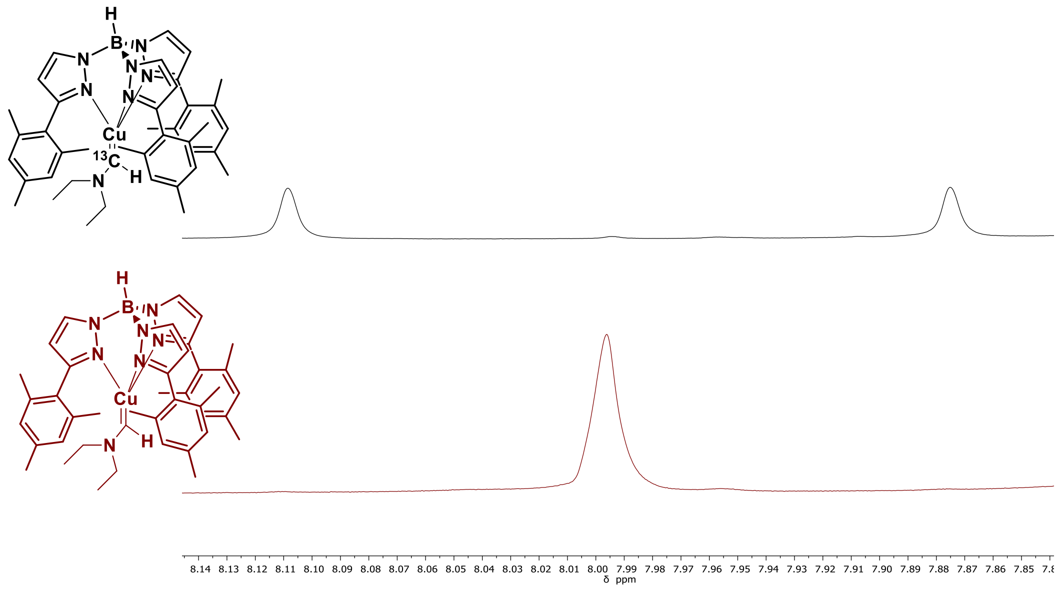

Figure S11: *Regions of the* ^{1}*H NMR spectra of* ***7*** *(bottom) and* ***7**** *(top) showing the Cu=C(H) resonances (500 MHz,* C_6D_6*).*

4. NMR studies of the reaction of 1 with diazo compounds 2* and 2**.

<u>*Reactivity of complex **1** with **2****</u>

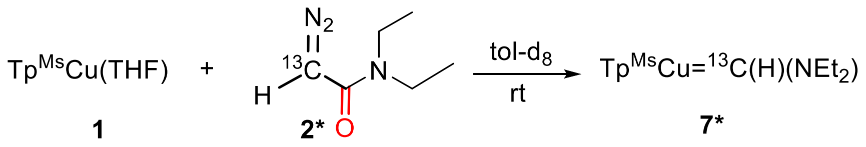

A ^{13}C-labeled 2-diazo-*N,N'*-diethyl acetamide (**2***, 42.6 mg, 0.3 mmol) solution in 0.3 mL of toluene-d$_8$ was added over a solution of Tp^{Ms}Cu(THF) (**1**, 42 mg, 0.06 mmol) in 0.5 mL of toluene-d$_8$ at room temperature, and the mixture was transferred to a NMR tube for reaction monitoring.

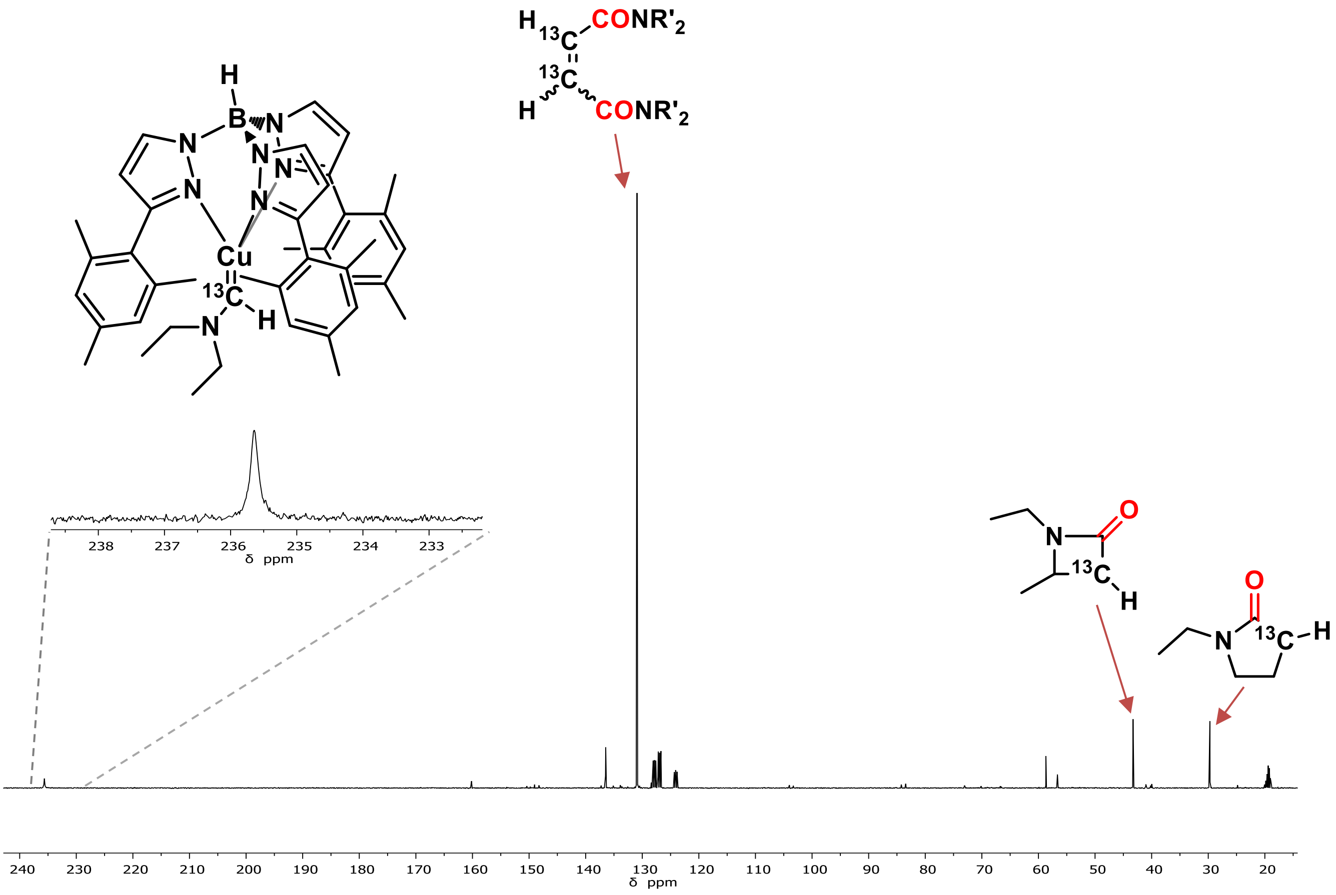

Figure S12**:^{13}C NMR spectrum (100 MHz, tol-d$_8$) of the reaction of complex **1** with 5 equiv of **2*.*

Reactivity of complex **1** with **2****

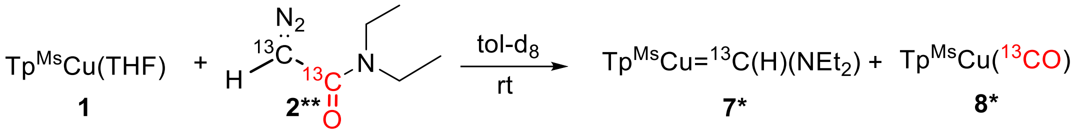

The same procedure was followed for **2**** as substrate. Figure S13 shows the NMR spectrum of the reaction mixture.

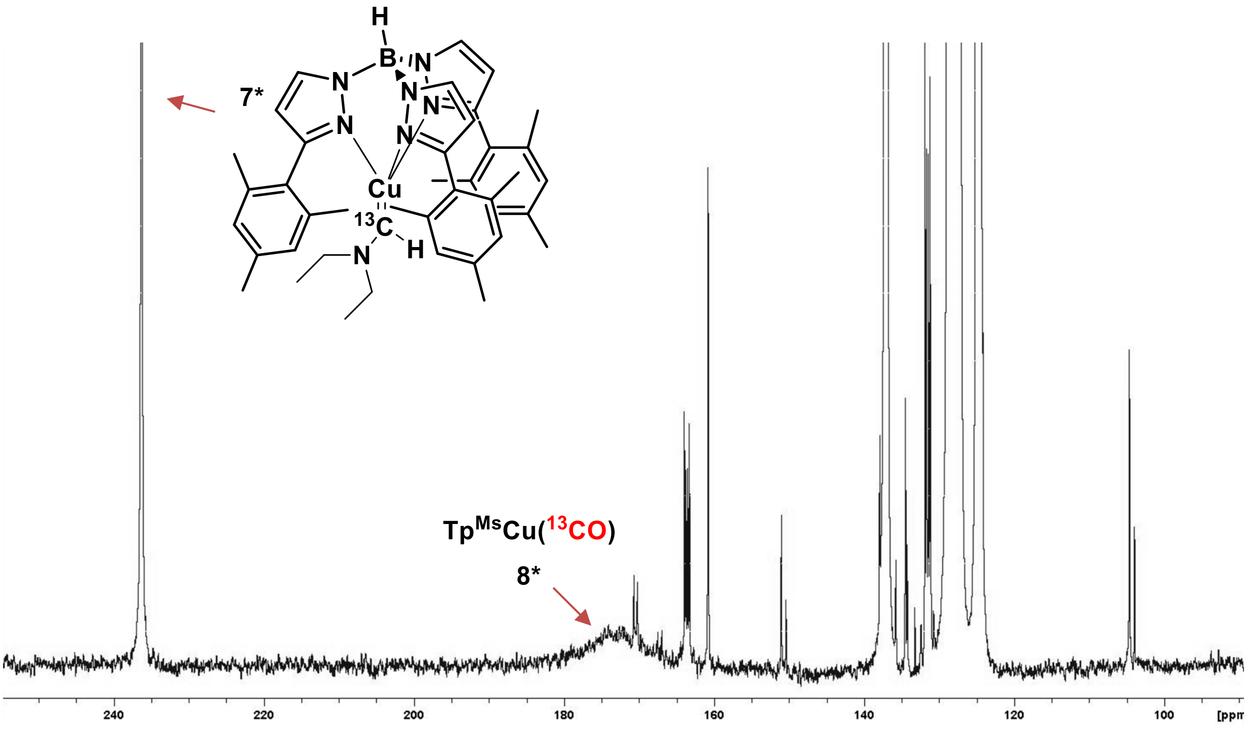

Figure S13**: ^{13}C NMR spectrum (125 MHz, tol-d$_8$) of the reaction of complex **1** with 5 equiv of **2*.*

Comparison of NMR spectra

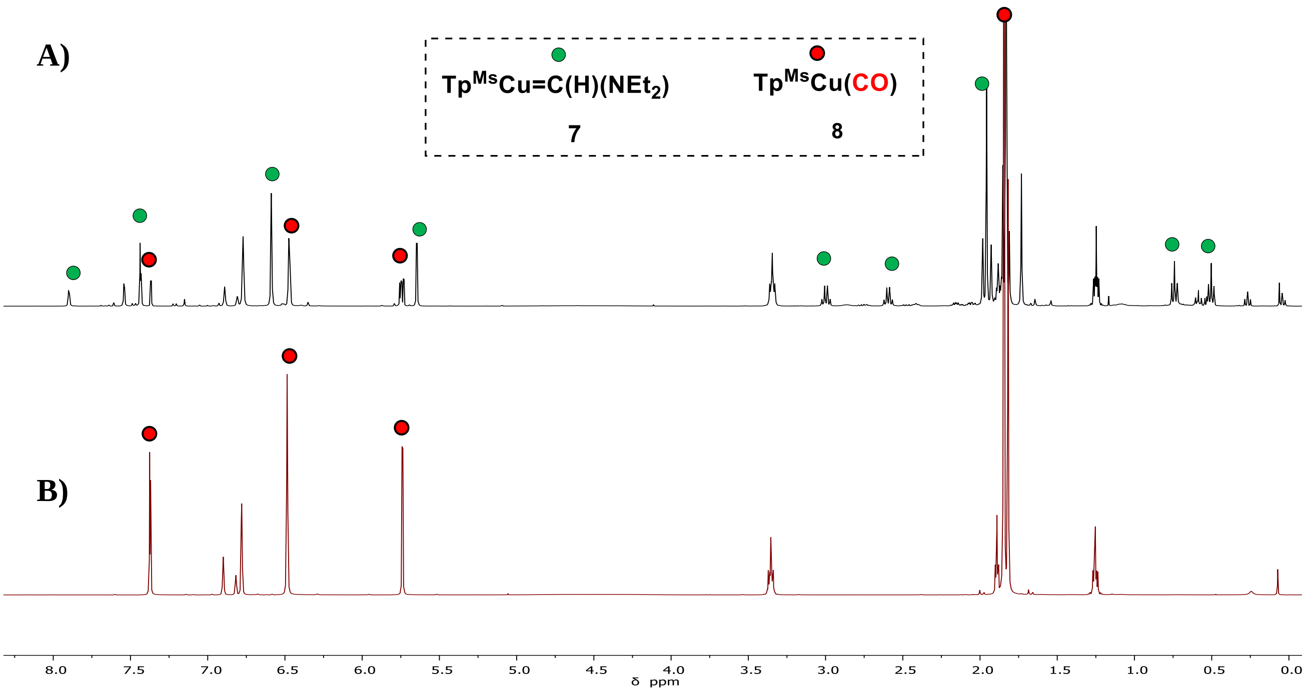

***Figure S14**: **A)** ^{1}H NMR spectrum (400 MHz, tol-d_8) of the crude of the reaction of complex **1** with 5 equiv of **2**, **B)** ^{1}H NMR spectrum (400 MHz, tol-d_8) of independently synthetized complex $Tp^{Ms}Cu(CO)$ **8**.*

Comparison of IR spectra

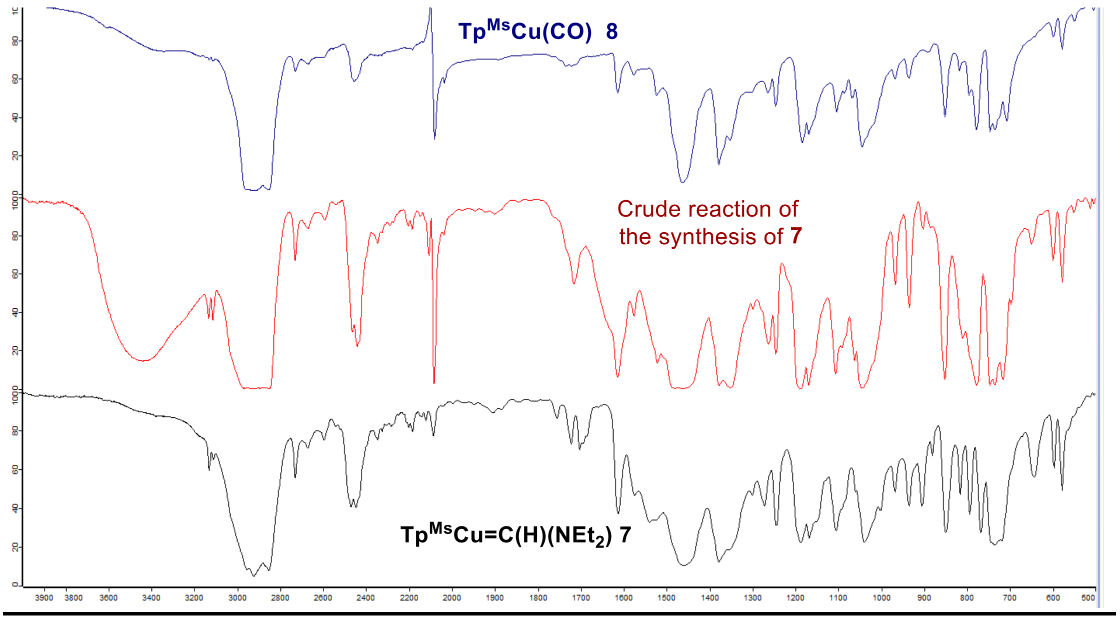

***Figure S15**: Comparison of IR spectra of $Tp^{Ms}Cu(CO)$ **8** (blue), crude of reaction of complex **1** with 5 equiv of **2**, (red) and complex $Tp^{Ms}Cu(CHNEt_2)$ **7** (black).*

5. Factors affecting the synthetic procedure of 7.

Yield of complex 7

In an ampoule under nitrogen atmosphere, the $Tp^{Ms}Cu(THF)$ complex (**1**, 30 mg, 0.0427 mmol) was dissolved in 5 mL of dry toluene. A solution of 2-diazo-*N*,*N′*-diethylacetamide (**2**, 30.1 mg, 0.21 mmol) in 2.5 mL of toluene was transferred via cannula. The results obtained with the different reaction conditions modifying the temperature (° C) and the reaction time (h) are shown below.

Temperature variation ***Time variation***

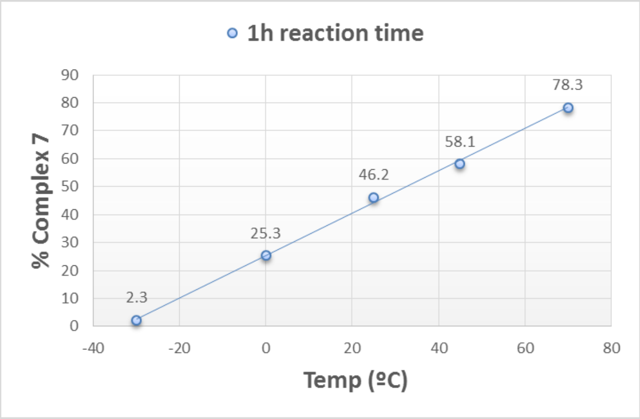

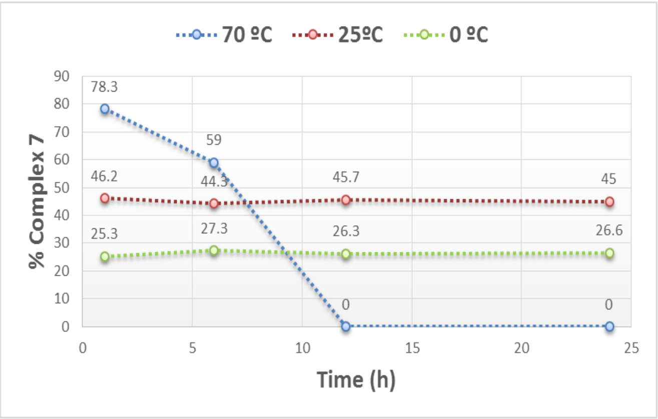

Reactivity of complex 7 with CO

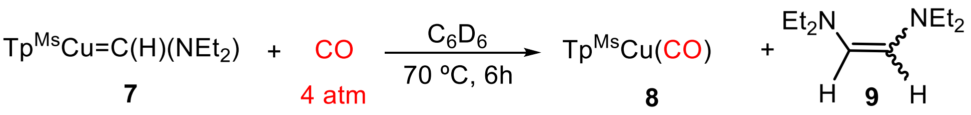

A solution of complex **7** in toluene-d_8 was transferred to a pressure NMR tube and submitted to 4 atm of carbon monoxide for 6 h at 70 °C. ^{1}H NMR studies showed the formation of complex **8** and compound **9**.

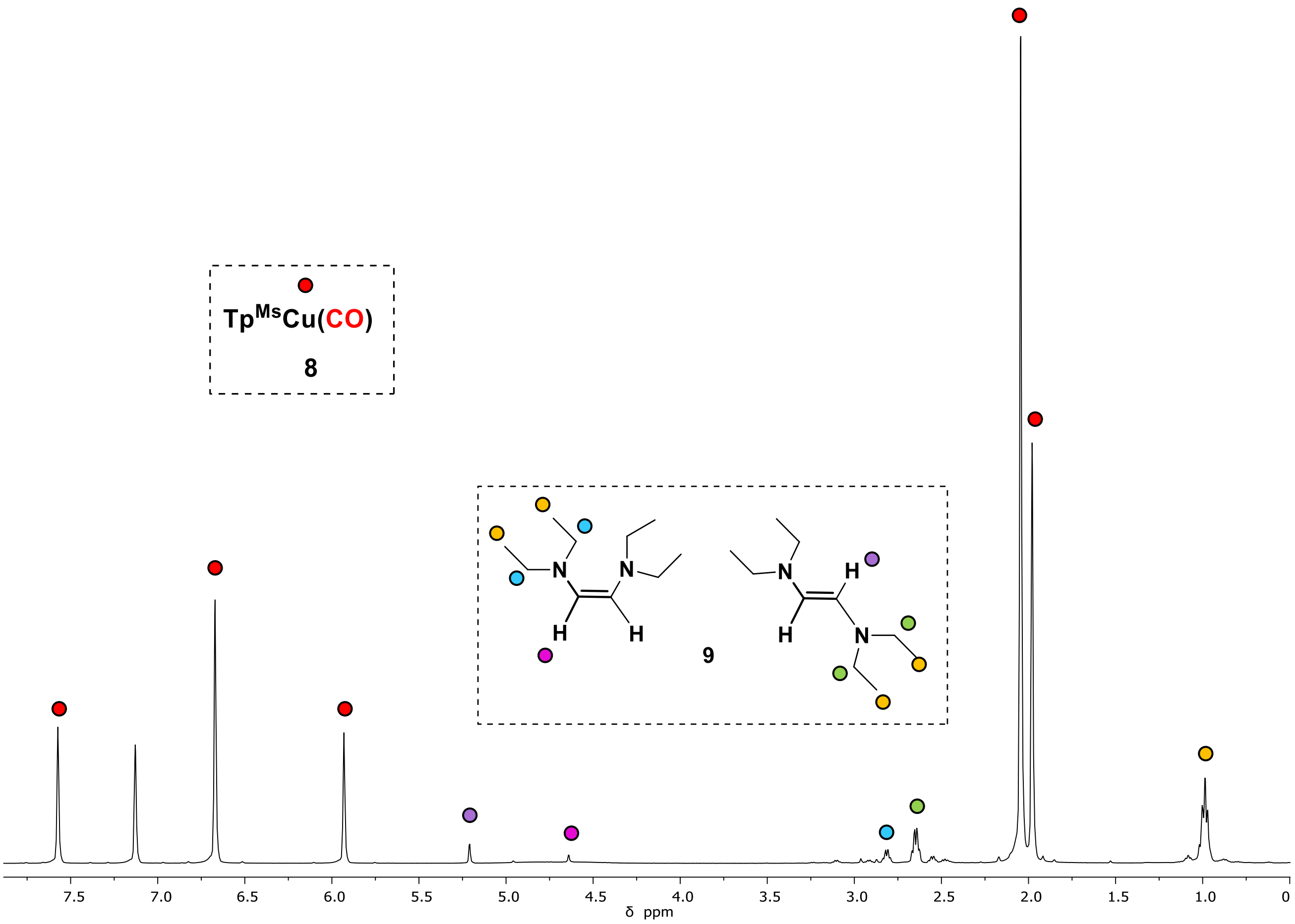

***Figure S16**: ^{1}H NMR spectrum (500 MHz, C_6D_6) of the reaction of complex **7** with 4 atm CO at 70 ºC for 6 h.*

6. Computational Methods

The calculations presented in this computational mechanistic study have been performed by optimization of minima and transition states with the B3LYP functional[3] including the D3 correction developed by Grimme and co-workers[4], B3LYP-D3, and as implemented in Gaussian 09.[5] Two different types of basis sets where

---

3 (a) A. D. Becke. Density functional thermochemistry: III. The role of exact exchange. *J.Chem. Phys.* **1993**, *98*, 5648−5652; (b) Stephens, P. J.; Devlin, F. J.; Chabalowski, C. F.; Frisch, M. J. Ab initio calculation of vibrational absorption and circular dichroism spectra using density functional force fields. *J. Phys. Chem.* **1994**, *98*, 11623−11627. (c) Lee, C.; Yang, W. T.; Parr, R. G. Development of the Colle-Salvetti Correlation-Energy Formula into a Functional of the Electron Density. *Phys. Rev. B: Condens. Matter Mater. Phys.* **1988,** *37*, 785−789.

4 Grimme, S.; Antony, J.; Ehrlich, S.; Krieg, H. A. Consistent and accurate ab initio parameterization of density functional dispersion correction (DFT-D) for the 94 elements H-Pu. *J. Chem. Phys.* **2010**, *132*, 154104.

5 Frisch, M.J.; Trucks, G.W.; Schlegel, H.B.; Scuseria, G.E.; Robb, M.A.; Cheeseman, J.R.; Scalmani, G.; Barone, V.; Mennucci, B.; Petersson, G.A.; Nakatsuji, H.; Caricato, M.; Li, X.; Hratchian, H.P.; Izmaylov, A.F.; Bloino, J.; Zheng, G.; Sonnenberg, J.L.; Hada, M.; Ehara, M.; Toyota, K.; Fukuda, R.; Hasegawa, J.; Ishida, M.; Nakajima, T.; Honda, Y.; Kitao, O.; Nakai, H.; Vreven, T.; Montgomery, J.A., Jr.; Peralta, J.E.; Ogliaro, F.; Bearpark, M.; Heyd, J.J.; Brothers, E.; Kudin, K.N.; Staroverov, V.N.; Kobayashi, R.; Normand, J.; Raghavachari, K.; Rendell, A.; Burant, J.C.; Iyengar, S.S.; Tomasi, J.; Cossi, M.; Rega, N.; Millam, N.J.; Klene, M.; Knox, J.E.; Cross, J.B.; Bakken, V.; Adamo, C.; Jaramillo, J.; Gomperts, R.; Stratmann, R.E.; Yazyev, O.; Austin, A. J.; Cammi, R.; Pomelli, C.; Ochterski,

used: For most calculations BS1 was used. In BS1 the 6-31+G(d,p) basis set[6] was used for all atoms except for copper, for which the Stuttgart−Dresden(SDD) basis set with effective core potential (ECP) was used instead.[7] For a reduced amount of calculations, those including two copper atoms and two ligands a smaller basis set, BS2, was used. The BS2 consists on the 6-31+G(d)[6] basis set for the atoms that come from the initial molecule of diazoacetamide, the 6-31g[6] basis sets for the atoms of the Tp^{Ms} ligand and the lanl2dz with an extra f-type function for copper[8]. BS2 was also used in initial calculations to explore the PES. The energies of the larger calculations with BS2, where merged with the energies obtained with BS1 taking the metallocarbene **7** as reference to join the free energy profiles. This species was chosen as it is more similar in nature to the large systems, although taking this species or the catalyst **1** and diazoacemide as reference has only a minor effects in the energies. Frequency calculations were carried out at the same level to obtain the free energies and ensure the nature of each stationary point. Solvent effects were taken into account by using the SMD[9] solvation model and using the default options for toluene and dichloromethane. A microkinetic model[10] was used along the investigations, the details of it can be found in the section of this Supporting Information called "Details of the Microkinetic Model". GoodVibes[11] program has been used to apply Cramer and Truhlar's[12] quasi-harmonic approximation low frequency vibrations (for mode details see ref [13]), to perform the change of the standard state from 1 atm to 1 M and to perform the changes of temperature. The geometries of all species relevant for this

J. W.; Martin, R.L.; Morokuma, K.; Zakrzewski, V.G.; Voth, G.A.; Salvador, P.; Dannenberg, J.J.; Dapprich, S.; Daniels, A.D.; Farkas, Ö.; Foresman, J.B.; Ortiz, J.V.; Cioslowski, J.; Fox, D.J. Gaussian, Inc., Wallingford CT, 2009.

6 (a) Ditchfield, R.; Hehre, W. J.; People, J. A. Self-consistent molecular-orbital. An extended gaussian-type basis for molecular-orbital studies of organic molecules. *J. Chem. Phys.* **1971**, 54, 724−728; (b) Hehre, W. J.; Ditchfield, R.; Pople. J. A. Self-Consistent Molecular Orbital Methods. XII. Further Extensions of Gaussian-Type Basis Sets for Use in Molecular Orbital Studies of Organic Molecules. *J. Chem. Phys.***1972**, *56*, 2257−2261; (c) Hariharan, P. C.; People, J. A.; Influence of polarization functions on molecular-orbital hydrogenation energies. *Theor. Chim. Acta* **1973**, *28*, 213−222; (d) Dill, J. D.; Pople, J. A. Self-consistent molecular orbital methods. XV. Extended Gaussian-type basis sets for lithium, beryllium, and boron. *J. Chem. Phys.* **1975**, *62*, 2921-2923; (e) Clark, T.; Chandrasekhar, J.; Spitznagel, G. W.; Schleyer, P. V. R. Efficient diffuse function-augmented basis sets for anion calculations. III. The 3-21+G basis set for first-row elements, Li-F. *J. Comput. Chem.* **1983**, *4*, 294-301.

7 Andrae, D.; Haeussermann, U.; Dolg, M.; Stoll, H.; Preuss, H. Energy-adjusted ab initio pseudopotentials for the 2nd and 3rd rowtransition-elements. *Theor. Chem. Acc.* **1990**, *77*, 123−141.

8 (a) Hay, P. J.; Wadt, W. R. Ab initio effective core potentials for molecular calculations. Potentials for K to Au including the outermost core orbitals. *J. Chem. Phys.* **1985**, *82*, 299-310; (b) Ehlers, A. W.; Böhme, M.; Dapprich, S.; Gobbi, A.; Höllwarth, A.; Jonas, V.; Köhler, K. F.; Stegmann, R.; Veldkamp, A.; Frenking, G. A set of f-polarization functions for pseudo-potential basis sets of the transition metals Sc-Cu, Y-Ag and La-Au. *Chem. Phys. Lett.* **1993**, *208*, 111-114.

9 Marenich, A. V.; Cramer, C. J.; Truhlar, D. J. Universal solvation model based on solute electron density and on a continuum model of the solvent defined by the bulk dielectric constant and atomic surface tensions. *J. Phys. Chem. B* **2009**, *113*, 6378−6396.

10 Besora, M.; Maseras, F. Microkinetic modeling in homogeneous catalysis. *WIREs Comput Mol Sci.* **2018**, 8:e1372.

11 Luchini, G.; Alegre-Requena, J. V.; Funes-Ardoiz, I; Paton, R. S. GoodVibes: Automated Thermochemistry for Heterogeneous Computational Chemistry Data. *F1000Research* **2020**, *9*, 291.

12 Ribeiro, R. F.; Marenich, A. V.; Cramer, C. J.; Truhlar, D: G. Use of Solution-Phase Vibrational Frequencies in Continuum Models for the Free Energy of Solvation. *J. Phys. Chem. B* **2011**, *115*, 14556–14562.

13 Besora, M.; Vidossich, P.; Lledós, A.; Ujaque, G.; Maseras, F. Calculation of Reaction Free Energies in Solution: A Comparison of Current Approaches. *J. Phys. Chem. A* **2018**, *122*, 1392–1399.

study are included in a data set collection of computational results available in the ioChem-BD repository and can be accessed via https://doi.org/10.19061/iochem-bd-1-202.

6.1. Detailed Reaction Mechanism in Toluene

We have searched for different plausible mechanisms for the formation of all species detailed in this section, we have looked for different intermediates and different types of transition states, including monomeric and dimeric species. The mechanisms detailed below are the preferred ones, the lowest in energy in all cases. The non-preferred reaction mechanisms are omitted for simplicity.

A. Reaction mechanism for the formation of the metallocarbene **6** from the diazoacetamide and catalyst **1.**

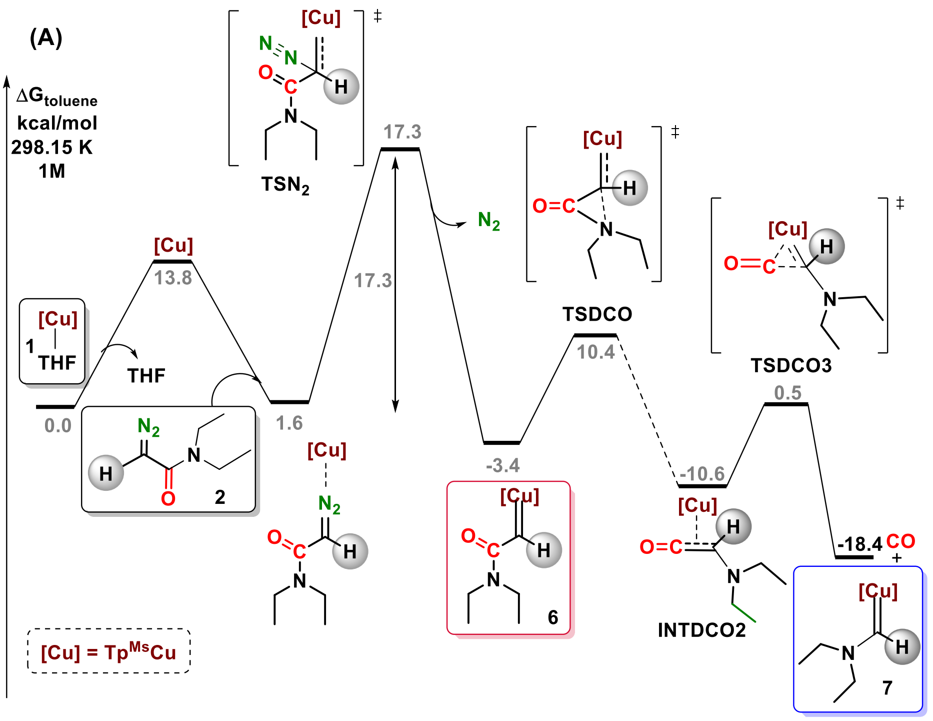

***Figure S17**: Free energy profile for the formation of the metallocarbene **6** from the diazoacetamide and catalyst **1**, followed by decarbonylation and formation of metallocarbene **7**. Free energies in toluene, in kcal.mol^{-1} and at 298 K.*

The mechanism starts from the copper catalyst Tp^{Ms}Cu(THF) complex **1**, see Fig S17. The complex first undergoes THF decoordination followed by diazoacetamide coordination, forming an adduct of similar stability

to **1**. Then, nitrogen extrusion takes place through a high energy transition state, located at 17.3 $kcal.mol^{-1}$, and forms the intermediate metallocarbene **6**, which is slightly more stable than reactants (-3.4 $kcal.mol^{-1}$).

The intermediate metallocarbene **6** can react in different ways forming either pyrrolidinone **3**, azetidinone **4**, olefin **5**, the decarbonylated metallocarbene **7** or olefin **9**. The different mechanisms have been explored and are detailed in this supporting information. We start by detailing the decarbonylation for the formation of metallocarbene **7**.

B. Reaction mechanism for the decarbonylation, formation of metallocarbene **7**.

To undergo decarbonylation the metallacorbene **6**, must go through transition state TSDCO, see Fig. S17. In this transition state (TS) the NEt_2 moiety in the metallocarbene **6** rotates around the N–C(O) bond and becomes perpendicular to the (OC)CH plane, a new bond between the nitrogen and the carbene carbon is being formed. The TS leads to INTDCO1 an unstable three-member cyclic intermediate. This species evolves through a low energy transition state, TSDCO2. This TSDCO2 breaks the N-C(=O) bond and forms INTDCO2, a rather stable ketene intermediate. TSDCO3 undergoes OC=C bond cleavage and leads to the formation the new decarbonylated metallocarbene **7**, which is 15.0 $kcal.mol^{-1}$ below the initial metallocarbene **6**. The barrier for the decarbonylation process is low at 298.15 K (13.8 $kcal.mol^{-1}$) while the reverse barrier is too high to take place.

Please note that two molecules of decarbonylated metallocarbene **7** can form a weak dimer, where the two metallocarbenes hold together thanks to weak interactions between Tp ligands and the carbenes. This dimer is located at +0.1 $kcal.mol^{-1}$ above the two monomers at 298 K and does not have an important role at this temperature or above, however at lower temperatures, due to entropic effects, it is more stable. For instance at 243 K it is 1.6 $kcal.mol^{-1}$ more stable than the separate monomers.

Free energies of all involved species at all temperatures can be found in the “Effect of the Temperature in the Free Energy Profile” section of this Supporting Information.

C. Reaction mechanism for the formation of the olefin **9** from decarbonylated metallocarbene **7**.

The computationally postulated reaction mechanism is presented in Fig. S18. The mechanism of formation of olefin **9** needs two metallocarbene 7 units to take place. Although it could be that one of them is never really formed as the mechanism goes one step back in the decarbonlyation mechanism, as it needs of INTDCO2 (see Fig. S17 and previous subsection). The metallocarbene **7** needs to add back a molecule of CO to form the above mentioned intermediate INTDCO2, this reacts with a new metallocarbene **7** forming the bulky unstable INTCC1

constituted by two copper complexes hold together by week interactions. This species will evolve to the similar and more stable intermediate INTCC2.

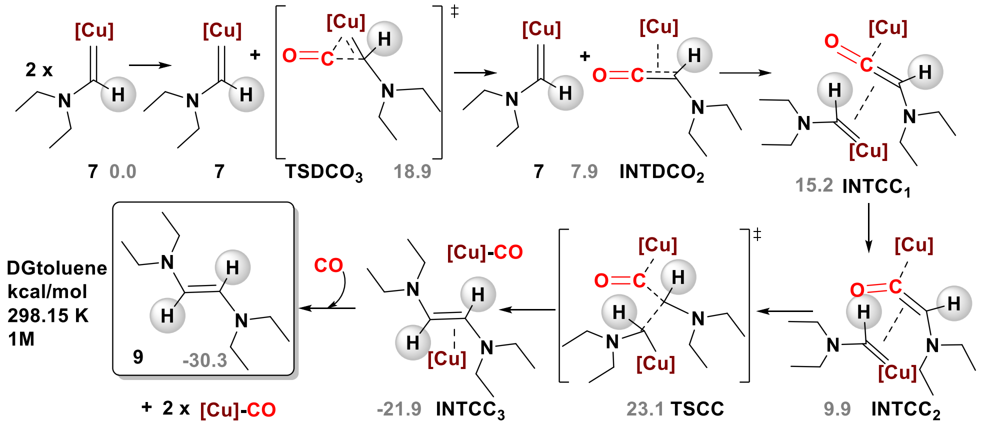

***Figure S18**: Reaction mechanism for the formation of the olefin **9** from decarbonylated metallocarbene **7**. Free energies in toluene, in kcal.mol^{-1} and at 298 K.*

INTCC2 differs from the previous one, in the coordination of the ketene to copper. In INTCC1 coordination is done mainly through the C=C bond and the O=C=C angle is almost linear, while in the energetically favored INTCC2 it is mainly coordinated through the internal carbon (O)C(C), and the OCC angle is clearly bend. The transition state between both intermediates, has been searched and no transition state could be located, neither seen in scans. Hence it is expected to be a non-barrier or a low barrier process. From INTCC2, TSCC located 23.1 kcal.mol^{-1} above the two molecules of metallocarbene **7** can form the new C=C bond. After the transition state the stable olefin **9** is released.

We have also investigated other reaction mechanisms for the same process, the most relevant are presented in Figure S19. Formation of a dimer of two decarbonylated metallocarbenes (**7**)$_2$ and direct formation of the olefin through **TSCC2**, seems and obvious reaction mechanism (a in Figure S19), however the barrier is very high 39.7 kcal.mol^{-1}. Another possibility is the formation of a free decarbonylated metallocarbene (**FC**). If the formation of the free metallocarbene takes place directly from **7** (b in Figure S19), the barrier is expected to be above 40 kcal.mol^{-1}. We haven't been able to locate the transition state to break the Cu=C bond. However as the direct products are located at 39.2 kcal.mol^{-1} above metallocarbene **7**, simply by taking into account diffusion we know we should have a barrier above 43 kcal.mol^{-1}, too high to take place at the experimental conditions.

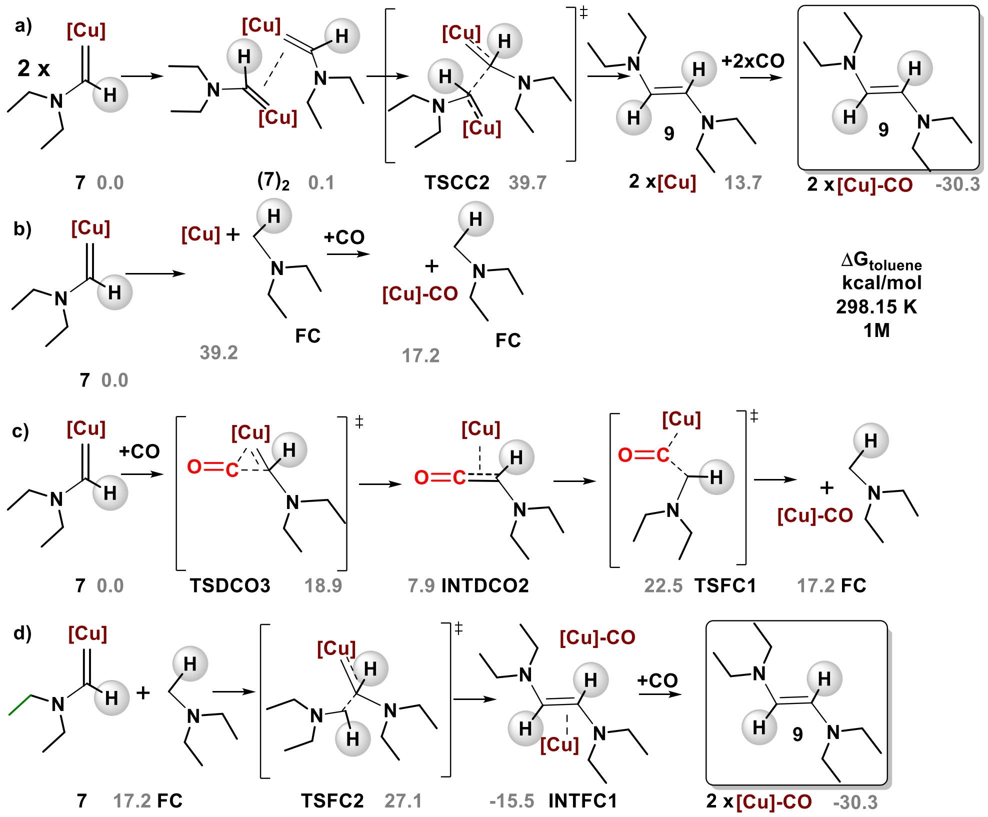

***Figure S19**:Other reaction mechanisms explored for the formation of the olefin **9** from decarbonylated metallocarbene **7**. Please note that the actual postulated mechanism is presented in Figure S18. Free energies in toluene, in kcal.mol^{-1} and at 298 K.*

Another possibility lower in energy consist on generating the free decarbonylated metallocarbene, not directly from **7** but from **INTDCO2** intermediate. This intermediate is found in the process of decarbonylation/decarbonylation and has relevance indeed in the decarbonylation process but also in the already postulated mechanism for the formation of olefin **9** in Figure S18. This mechanism presented in Figure S19 (c)starts by the addition of CO and formation of **INTDCO2** through **TSDCO3**. From this intermediate the (O)C=C bond is broken at the same time as the carbonyl coordinates to the metal center. The free decarbonylated metallocarbene **FC** is generated through **TSFC1**. This transition state is located at 22.5 kcal.mol^{-1}, the barrier is accessible at the experimental temperatures however he product formed is rather unstable (+17.2 kcal.mol^{-1}). Formation of olefin **9** from **FC** would require reacting with a new molecule of metallocarbene **7**. Formation of olefin **9** could be achieved through **TSFC2** (d) with an overall barrier from two **7** molecules of 27.1 kcal.mol^{-1}, but this barrier is relatively high and also 4 kcal.mol^{-1} higher than the barrier for the actual postulated

mechanism through **TSCC** in Figure S18. One could envision another mechanism consisting on the reaction of two **FC** molecules, however these two species are very unstable (2 x 17.2 kcal.mol^{-1}). Hence the mechanisms depicted in Figure S19 can be discarded in front of the mechanism presented in Figure S18.

D. <u>Reaction mechanism for the formation o olefin **5** from metallocarbene **6**.</u>

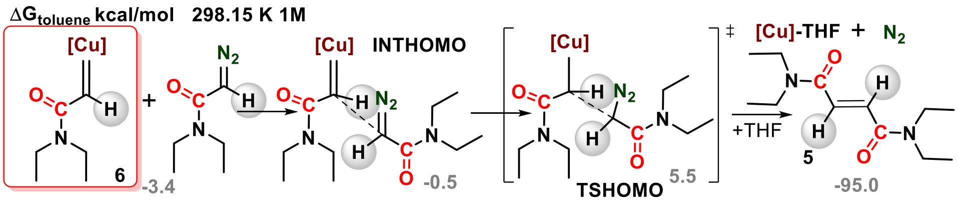

***Figure 20**: Reaction mechanism for the formation of olefin **5** from metallocarbene **6**. Free energies in toluene and in kcal.mol^{-1} at 298 K.*

The metallocarbene 6 formed from the diazoacetamide (see Figure S17) can react with a second molecule of diazoacetamide and form an olefin resulting from the homocoupling of two reactants. The transition state for the process has been located 8.9 kcal.mol^{-1} above the metallocarbene **6**. This transition state an others isomers have been difficult to locate as they have a relatively low energy barrier and because the negative frequency associated to the TS is also relatively low (≈ -80 cm^{-1}).

6.2. Detailed Reaction Mechanism in Dichloromethane

A. <u>Reaction mechanism for the formation of the metallocarbene 6 from the diazoacetamide and catalyst 1.</u>

The overall mechanism is similar to the one reported in toluene in previous section. For a discussion on the formation of metallocarbene 6 see previous section, the results are presented in Fig S21. Continuum solvent effects predict a slightly larger barrier for the N_2 extrusion in dichloromethane (17.9 kcal.mol^{-1}) than in toluene (17.3 kcal.mol^{-1}).

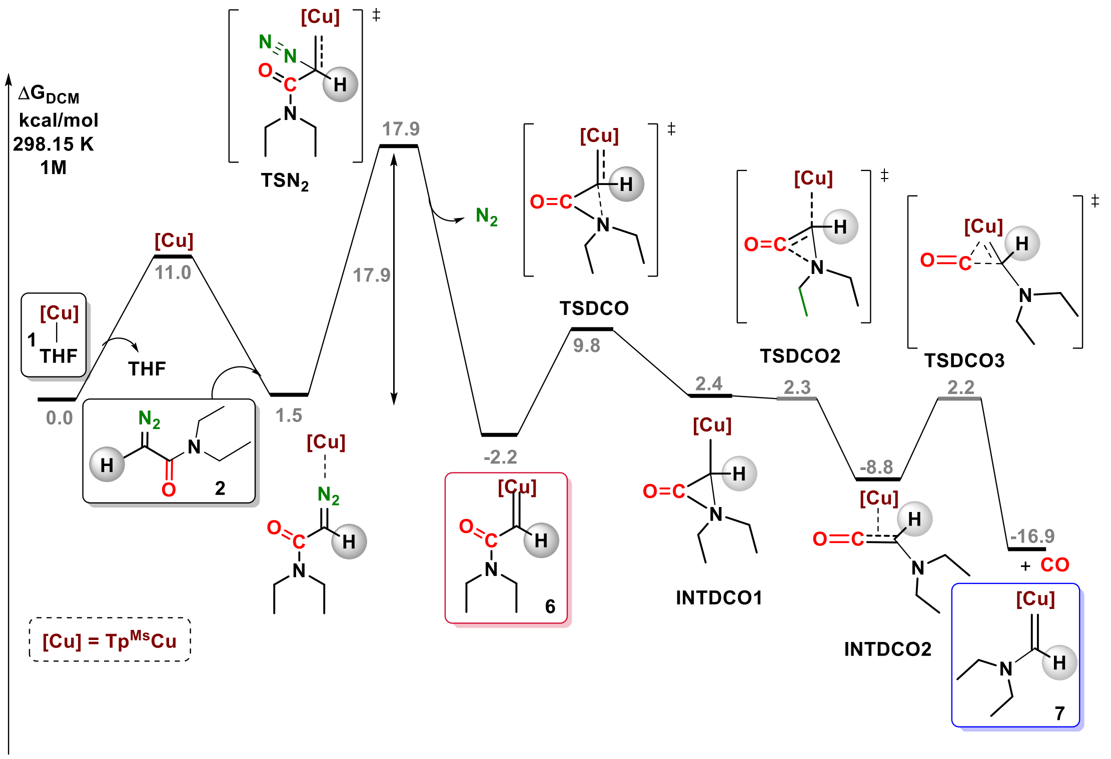

Figure S21: *Free energy profile for the formation of the metallocarbene **6** from the diazoacetamide and catalyst **1**, followed by decarbonylation and formation of metallocarbene **7**. Free energies in dichloromethane, in kcal.mol^{-1} and at 298 K.*

B. <u>Reaction mechanism for the decarbonylation, formation of metallocarbene **7**.</u>

The decarbonylation mechanism is presented in Fig S21 and explained in detail in previous section for toluene. However there is an issue that needs to be commented on. In dichloromethane TSDCO2 has slightly lower energy than INTDCO1, the preceding intermediate. The energy of the TS is lower than the previous intermediate in free energy terms, but higher in the potential energy surface. This does not occur in toluene where the barrier is only slightly larger.

C. <u>Reaction mechanism for the formation of the olefin **9** from decarbonylated metallocarbene **7**.</u>

For a discussion on the formation of olefin **9** see previous section for equivalent in toluene, the results in dichloromethane are presented in Fig S22. The barrier for the formation of the olefin **9** is a little higher.

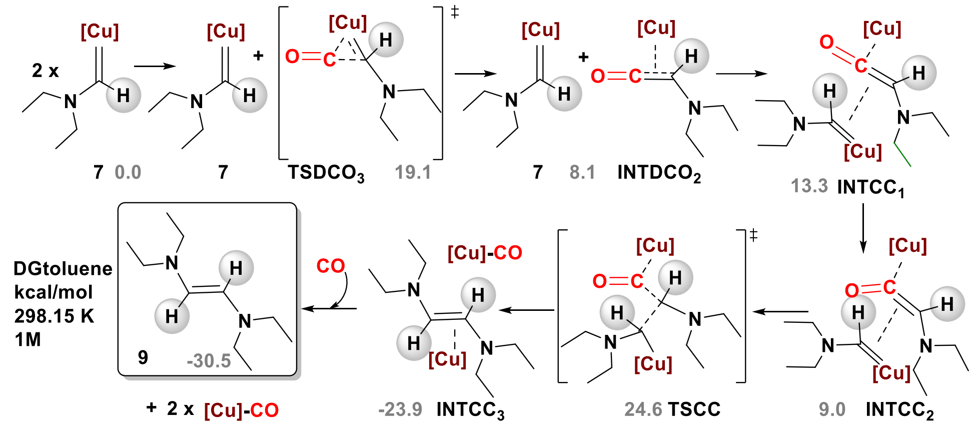

Figure S22: *Reaction mechanism for the formation of the olefin* ***9*** *from decarbonylated metallocarbene* ***7****. Free energies in dichloromethane, in kcal.mol*$^{-1}$ *and at 298 K.*

Results for other discarded mechanisms are presented in Figure S23. They are very similar to the ones reported for toluene in Figure S19, please see results on toluene for a discussion on the different reaction pathways.

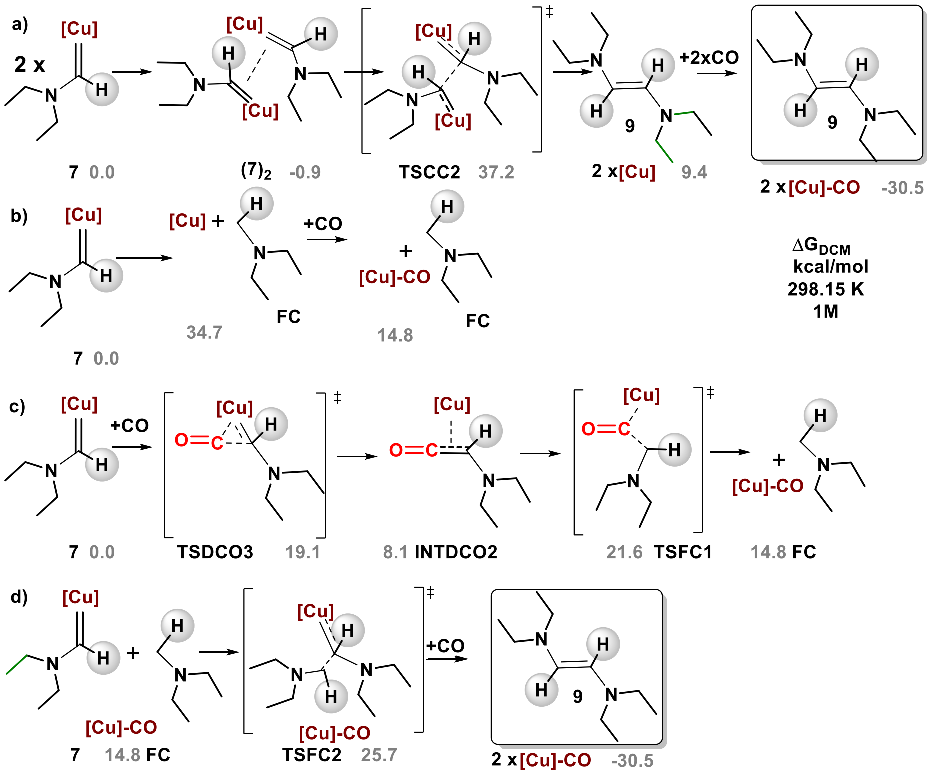

Figure S23: *Other reaction mechanisms explored for the formation of the olefin* ***9*** *from decarbonylated metallocarbene* ***7****. Please note that the actual postulated mechanism is presented in* ***Figure S22****. Free energies in dichloromethane, in kcal.mol*$^{-1}$ *and at 298 K.*

D. Reaction mechanism for the formation of olefin **5** from metallocarbene **6**.

For a discussion on the formation of olefin **5** see previous section for equivalent in toluene, the results in dichloromethane are presented in Fig S24.

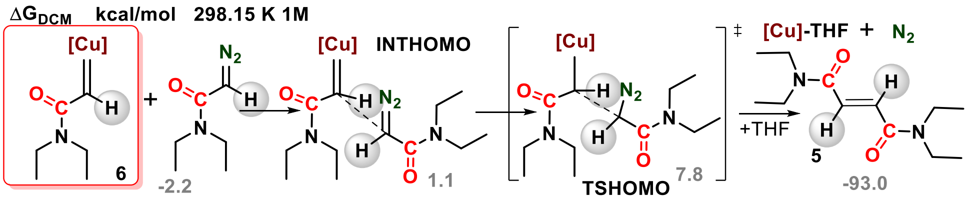

Figure S24: *Reaction mechanism for the formation of olefin* **5** *from metallocarbene* **6**. *Free energies in dichloromethane, in kcal.mol^{-1} and at 298* K.

6.3 Formation of pyrrolidinone 3 and azetidinone 4 from metallocarbene 6.

Experimentally it has been observed that when the reaction is carried in dichloromethane instead of toluene, in addition to the conversion of copper metallocarbene **6** into olefin **5** and decarbonylated metallacorbene **7** (and olefin **9**), two other catalytic processes take place. Both involve carbene insertion into C-H bonds, and the experimentally observed products are pyrrolidinone **3** and azetidinone **4**. The reaction pathways for the formation of these two products from metallocarbene **6** have been computed and are presented in Fig S25.

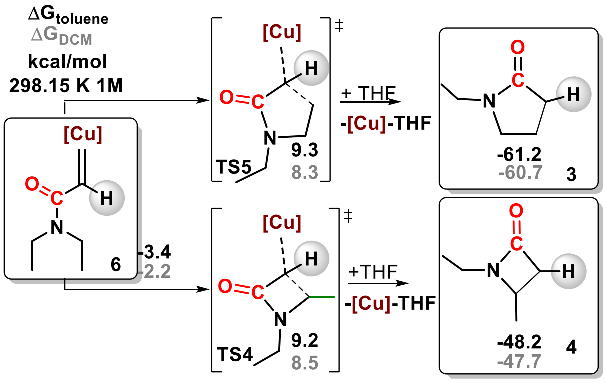

Figure S25: *Reaction mechanism for the formation of pyrrolidinone* **3** *and azetidinone* **4** *from metallocarbene* **6**. *Free energies in toluene (black) and dichloromethane (green), in kcal.mol^{-1} and at 298 K.*

In dichloromethane the located transition states follow very well the experimental yields, see Table S1. Not only the barriers but also the kinetics, as please note that formation of **3**, **4** and **7** are intramolecular reactions

whilst formation of **5** is a bimolecular reaction with different kinetics. Computational and experimental results show a very good agreement.

***Table S1**: Summary of the experimental yields and computational reaction barriers from metallocarbene **6** to the different observed products. Free energies in dichloromethane or toluene, in kcal.mol^{-1} and at 298 K.*

	TS4	TS5	TSDCO	TSHOMO
	product 4	product 3	carbene 7 (or 9)	olefin 5
dichloromethane 298.15 K				
$\Delta G^{‡}$ comp.	8.5	8.3	9.8	7.8
Experimental yield	8%[a)]	12%[a)]	present[a)]	53 %[a)]
toluene 298.15 K				
$\Delta G^{‡}$ comp.	9.2	9.3	10.4	5.5
Experimental yield.	~ 0 [b)]	~ 0 [b)]	46.2 [b)]	22 [b)]

*a) These experiments were carried at high concentration of diazoacetamide 2.5 10^{-2} M and 3.1 10^{-4} M in [Cu]-THF, **1**. Evidences of the presence of carbene 7 were done as 27 % of diazoacetamide remained unreactive.*

*b) These experiments were carried at high concentration of diazoacetamide 8.5 10^{-2} M and 8.5 10^{-3} M in [Cu]-THF, **1**.*

In toluene the computed barriers do not agree that well, as metallocarbene 7 is observed while products **3** and **4** are not. However it does agree with the experimental results in the fact that the product of the homocoupling product is the preferred at 298.15 K. Hence in toluene we can deduce that the computational barriers for the formation of pyrrolidinone **3**, azetidinone **4** are overstabilized, probably by a couple of kcal.mol^{-1}. As the agreement in dichloromethane is very good, it could be that our continuum model for toluene does not provide a good description of the differences between ring closures and the rotation for the decarbonylation. Indeed the three TSs are intramolecular, but the ones of carbene insertion into C-H bonds differ significantly from the decarbonylation one.

7. Effect of the Temperature in the Free Energy Profile

To reproduce the experimental results the free energy barriers have been recomputed at 243.15, 273.15, 298.15, 318.15 and 343.15 K. The free energy values of all the relevant species in the mechanism are provided in Table S2 for toluene.

Table S2: *Computational free energies in kcal.mol^{-1} in toluene for the relevant species in the different reaction pathways at 243.15, 273.15, 298.15, 318.15 and 343.15 K.*

	243.15	273.15	298.15	318.15	343.15
**1,** [Cu]-THF	0.0	0.0	0.0	0.0	0.0
[Cu] + THF	15.6	14.6	13.8	13.1	12.4
[Cu]-diazoacetamide	0.8	1.2	1.6	1.8	2.2
TSN2	16.6	17.0	17.3	17.5	17.8
**6**	-2.4	-2.9	-3.4	-3.7	-4.2
TS4	9.9	9.5	9.2	8.9	8.6
TS5	10.1	9.7	9.3	9.1	8.8
INTHOMO	-2.1	-1.2	-0.5	0.1	0.8
TSHOMO	3.6	4.6	5.5	6.2	7.1
TSDCO	11.2	10.7	10.4	10.1	9.7
INTDCO2	*-9.5*	*-10.1*	*-10.6*	*-10.9*	*-11.4*
TSDCO3	*1.6*	*1.0*	*0.5*	*0.1*	*-0.4*
**7**	-15.7	-17.2	-18.4	-19.4	-20.6
[Cu]CO, **8**	-8.2	-8.2	-8.3	-8.3	-8.3
INTCC1	-21.4	-21.5	-21.6	-21.7	-21.8
INTCC2	-26.7	-26.8	-26.9	-26.9	-26.9
TSCC	-13.6	-13.7	-13.8	-13.8	-13.8
Dimer (**7**)$_2$	-34.8	-35.9	-36.8	-37.5	-38.3

8. Details of the Microkinetic Model

We constructed a microkinetic model with all relevant species of the reaction mechanism for toluene. All species were included with the energies reported in the Table S2, and all the relation between them. Please for details about how to obtain the reaction rates and how to construct the model from data obtained computationally, refer to our recent review on this topic [10]. Simulations were run with the Copasi software.[14] The chemical equations used can be checked in the Copasi Parameter File section of this Supporting Information. For the sake of simplicity formation of **3** and **4** was not included in the model as its formation is not experimentally observed in toluene.

All diffusion controlled steps were taken into account with the Stokes–Einstein equation with the Smoluchowski relation. The diffusion rate constant k_D can be approximated as $k_D = 8k_BT/3\eta$, where k_B is the Boltzmann constant, T is the temperature and η is the fluid viscosity.[15]. The toluene viscosity at the different temperatures was obtained from the literature and the corresponding k_D computed.[16] The reaction rates at different temperatures and associated free energies are presented in Table S3.

Table S3: *Temperature (K), rate constant and associated free energy barriers for the diffusion controlled steps ΔG_D (kcal.mol^{-1}) in toluene.*

T (K)	k_D	ΔG_D
243.15	4.37E+009	3.4
273.15	7.90E+009	3.6
**298.15**	**1.19E+010**	**3.7**
318.15	1.59E+010	3.8
343.15	2.18E+010	3.9

With that, the microkinetic model we run with the data provided showed a too slow reaction for the formation of **9** at high temperatures and a deviated dependence of temperature for the formation of **7**.

14 Hoops, S.; Sahle, S.; Gauges, R.; Lee, C.; Pahle, J.; Simus, N.; Singhal, M.; Xu, L.; Mendes, P.; Kummer, U. COPASI: a COmplex PAthway SImulator. *Bioinformatics* **2006,** *2,* 3067-3074.
15 Rush, L. E.; Pringle, P. G.; Harvey, J. N. Computational kinetics of cobalt-catalyzed alkene hydroformylation. *Angew. Chem., Int. Ed.* **2014**, *53,* 8672–8676.
16 Krall, A. H.; Sengers, J. V. Viscosity of Liquid Toluene at Temperatures from 25 to 150 °C and at Pressures up to 30MPa. *J. Chem. Eng .Data* **1992**, *37,* 349-355.

Microkinetic models are very sensible to some of the energies introduced and of the postulated mechanism. Unluckily barrier calculation by DFT means has associated important errors, depending on the systems we can get fairly good results partly due to error cancellation, but in other occasions errors can be large. Also in the calculation of the reaction rates from computed free energies necessary to build the microkinetic model we perform important simplifications (assuming ideal preexponential factors and concentrations).

When comparing raw computational and experimental free energies we accept some discrepancies as "normal". We should do the same in microkinetic models, and that has already been done by some of us,[17] and followed by others[18] through adjustment of the computed free energy profiles.

We have carefully check what was necessary to adjust our energies to reproduce the experimental observations and we found out that the energies of the two metallocarbenes were likely to have important errors. Please note that the decrease of the transition state barriers by lowering the transition states didn't match with experiments. We adjusted two values:

1) The diffusion barrier between the metallocarbene **6** and the INTHOMO (and adduct between the metallocarbene and the diazoacetamide) had to be adjusted 6.0 kcal.mol^{-1} higher to reproduce the experimental trends of Fig 4A of the main text. This is equivalent to increasing the energy of the metallocarbene by the same amount. Of course we cannot be sure if we have two errors added up, like an error of 4 kcal.mol^{-1} in the energy of the metallocarbene **6** plus 2 kcal.mol^{-1} in the diffusion of the diazoacetamide.

2) The energy of the metallocarbene 7 must also be adjusted to a higher energy, in this case to 3.8 kcal.mol^{-1}.

With these adjustments the microkinetic model can reproduce in a satisfactory manner the experimental results, supporting the postulated mechanism.

17 Pérez-Soto, R.; Besora, M.; Maseras, F. The Challenge of Reproducing with Calculations Raw Experimental Kinetic Data for an Organic Reaction. *Org. Lett.* **2020**, *22*, 2873–2877.
18 Sanders, J. N.; Jun, H.; Yu, R. A.; Gleason, J. L.; Houk K. N. Mechanism of an Organocatalytic Cope Rearrangement Involving Iminium Intermediates: Elucidating the Role of Catalyst Ring Size. *J. Am. Chem. Soc.* **2020**, *142*, 16877-16886.

9. Results Of the Microkinetic Model at Different Temperatures

The most relevant results of the microkinetic model are presented in Table S3. The results are also plotted together with the experimental values in Fig S26.

Please note that at low temperatures there is a very small portion of **7** that is in the form of a weak dimer. The concentration of the dimer has been added up (twice as it is a dimer) to the concentration of **7** and the contribution of both is presented as % complex **7**.

Table S4: *Results of the Microkinetic Model. % of compound **7** and it's dimer (**7**)$_2$ versus time in hours at the different studied temperatures 243.15, 273.15, 298.15, 318.15 and 343.15 K.*

	**7** + (**7**)$_2$	**7** + (**7**)$_2$	**7**	**7**	**7**
**time (h)/T(K)**	**243**	**273**	**298**	**318**	**343**
**0**	0.0	0.0	0.0	0.0	0.0
**0.25**	0.4	22.6	46.4	55.5	73.3
**0.5**	1.5	22.6	46.4	54.9	78.2
**0.75**	3.1	22.6	46.4	54.2	80.2
**1**	3.9	22.6	46.4	53.5	80.8
**1.25**	4.3	22.6	46.4	52.9	80.6
**1.5**	4.5	22.6	46.4	52.2	80.0
**1.75**	4.6	22.6	46.4	51.5	79.2
**2**	4.7	22.6	46.4	50.9	78.2
**2.25**	4.7	22.6	46.4	50.2	77.1
**2.5**	4.7	22.6	46.4	49.6	75.9
**2.75**	4.6	22.6	46.4	49.0	74.7
**3**	4.6	22.6	46.4	48.3	73.5
**3.25**	4.6	22.6	46.4	47.7	72.2
**3.5**	4.5	22.6	46.4	47.1	71.0
**3.75**	4.5	22.6	46.4	46.5	69.7
**4**	4.5	22.6	46.4	45.9	68.4
**4.25**	4.5	22.6	46.4	45.4	67.2
**4.5**	4.4	22.6	46.4	44.8	65.9

**4.75**	4.4	22.6	46.4	44.2	64.7
**5**	4.4	22.6	46.4	43.7	63.4
**5.25**	4.4	22.6	46.4	43.1	62.2
**5.5**	4.4	22.6	46.4	42.6	61.0
**5.75**	4.4	22.6	46.4	42.1	59.8
**6**	4.4	22.6	46.4	41.6	58.6
**6.25**	4.4	22.6	46.4	41.1	57.5
**6.5**	4.4	22.6	46.4	40.6	56.3
**6.75**	4.3	22.6	46.4	40.1	55.2
**7**	4.3	22.6	46.4	39.6	54.1
**7.25**	4.3	22.6	46.4	39.1	53.0
**7.5**	4.3	22.6	46.4	38.7	51.9
**7.75**	4.3	22.6	46.4	38.2	50.8
**8**	4.3	22.6	46.4	37.7	49.8
**8.25**	4.3	22.6	46.4	37.3	48.8
**8.5**	4.3	22.6	46.4	36.9	47.8
**8.75**	4.3	22.6	46.4	36.4	46.8
**9**	4.3	22.6	46.4	36.0	45.9
**9.25**	4.3	22.6	46.4	35.6	44.9
**9.5**	4.3	22.6	46.4	35.2	44.0
**9.75**	4.3	22.6	46.4	34.8	43.1
**10**	4.3	22.6	46.4	34.4	42.2
**10.25**	4.3	22.6	46.4	34.0	41.4
**10.5**	4.3	22.6	46.4	33.6	40.5
**10.75**	4.3	22.6	46.4	33.3	39.7
**11**	4.3	22.6	46.4	32.9	38.9
**11.25**	4.3	22.6	46.4	32.5	38.1
**11.5**	4.3	22.6	46.4	32.2	37.3
**11.75**	4.3	22.6	46.4	31.8	36.5
**12**	4.3	22.6	46.4	31.5	35.8
**12.25**	4.3	22.6	46.4	31.2	35.1

**12.5**	4.3	22.6	46.4	30.8	34.4
**12.75**	4.3	22.6	46.4	30.5	33.7
**13**	4.3	22.6	46.4	30.2	33.0
**13.25**	4.3	22.6	46.4	29.9	32.3
**13.5**	4.3	22.6	46.4	29.6	31.7
**13.75**	4.3	22.6	46.4	29.3	31.0
**14**	4.3	22.6	46.4	29.0	30.4
**14.25**	4.3	22.6	46.4	28.7	29.8
**14.5**	4.3	22.6	46.4	28.4	29.2
**14.75**	4.3	22.6	46.4	28.1	28.6
**15**	4.3	22.6	46.4	27.8	28.0
**15.25**	4.3	22.6	46.4	27.5	27.5
**15.5**	4.3	22.6	46.4	27.3	26.9
**15.75**	4.3	22.6	46.4	27.0	26.4
**16**	4.3	22.6	46.4	26.7	25.9
**16.25**	4.3	22.6	46.4	26.5	25.4
**16.5**	4.3	22.6	46.4	26.2	24.9
**16.75**	4.3	22.6	46.4	26.0	24.4
**17**	4.3	22.6	46.4	25.7	23.9
**17.25**	4.3	22.6	46.4	25.5	23.4
**17.5**	4.3	22.6	46.4	25.2	23.0
**17.75**	4.3	22.6	46.4	25.0	22.5
**18**	4.3	22.6	46.4	24.8	22.1
**18.25**	4.3	22.6	46.4	24.5	21.6
**18.5**	4.3	22.6	46.4	24.3	21.2
**18.75**	4.3	22.6	46.4	24.1	20.8
**19**	4.3	22.6	46.4	23.9	20.4
**19.25**	4.3	22.6	46.4	23.7	20.0
**19.5**	4.3	22.6	46.4	23.5	19.7
**19.75**	4.3	22.6	46.4	23.3	19.3
**20**	4.3	22.6	46.4	23.0	18.9

**20.25**	4.3	22.6	46.4	22.8	18.6
**20.5**	4.3	22.6	46.4	22.6	18.2
**20.75**	4.3	22.6	46.4	22.4	17.9
**21**	4.3	22.6	46.4	22.3	17.5
**21.25**	4.3	22.6	46.4	22.1	17.2
**21.5**	4.3	22.6	46.4	21.9	16.9
**21.75**	4.3	22.6	46.4	21.7	16.6
**22**	4.3	22.6	46.4	21.5	16.3
**22.25**	4.3	22.6	46.4	21.3	16.0
**22.5**	4.3	22.6	46.4	21.2	15.7
**22.75**	4.3	22.6	46.4	21.0	15.4
**23**	4.3	22.6	46.4	20.8	15.2
**23.25**	4.3	22.6	46.4	20.6	14.9
**23.5**	4.3	22.6	46.4	20.5	14.6
**23.75**	4.3	22.6	46.4	20.3	14.4
**24**	4.3	22.6	46.4	20.1	14.1
**24.25**	4.3	22.6	46.4	20.0	13.9
**24.5**	4.3	22.6	46.4	19.8	13.7
**24.75**	4.3	22.6	46.4	19.7	13.4
**25**	4.3	22.6	46.4	19.5	13.2
**25.25**	4.3	22.6	46.4	19.4	13.0
**25.5**	4.3	22.6	46.4	19.2	12.8
**25.75**	4.3	22.6	46.4	19.1	12.6
**26**	4.3	22.6	46.4	18.9	12.4
**26.25**	4.3	22.6	46.4	18.8	12.2
**26.5**	4.3	22.6	46.4	18.6	12.0
**26.75**	4.3	22.6	46.4	18.5	11.8
**27**	4.3	22.6	46.4	18.4	11.6
**27.25**	4.3	22.6	46.4	18.2	11.4
**27.5**	4.3	22.6	46.4	18.1	11.3
**27.75**	4.3	22.6	46.4	18.0	11.1

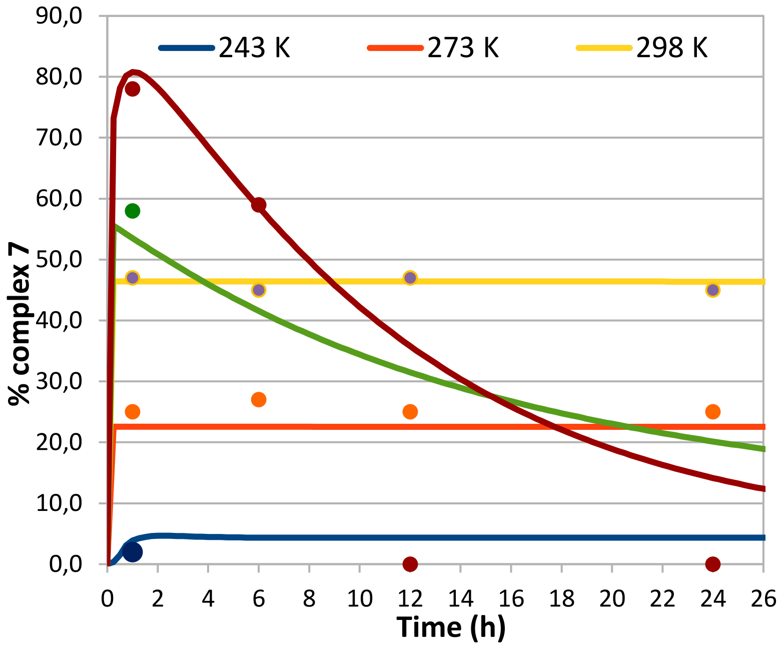

Fig S26. *Evolution over time (h) of the % yield of metallocarbene* ***7*** *as a result of microkinetic simulations using the computationally postulated mechanism and barriers at different temperatures (K) experimental results are added for comparison.*

10. Copasi Parameter Files at Different Temperatures

Detail of the Reactions described in Copasi

	#	Name	Reaction	Rate Law	Flux [mmol/s]	Number Flux [1/(s)]	Noise Expression
1	1	r01	cuthf = cu + thf	Mass action (reversible)	nan	nan	
2	2	r02	cu + diazo = cudiazo	Mass action (reversible)	nan	nan	
3	3	r03	cudiazo = cucarbe + n2	Mass action (reversible)	nan	nan	
4	4	r06	cucarbe = intcarbenew	Mass action (reversible)	nan	nan	
5	5	r04	cucarbe + diazo = addhomo	Mass action (reversible)	nan	nan	
6	6	r05	addhomo -> prodhomo + cu + n2	Mass action (irreversible)	nan	nan	
7	7	r07	intcarbenew = cucarbenew + co	Mass action (reversible)	nan	nan	
8	8	r08	intcarbenew + cucarbenew = dimintnew	Mass action (reversible)	nan	nan	
9	9	r12	cuco = cu + co	Mass action (reversible)	nan	nan	
10	10	r11	prodnew2cu = prodnew2 + cu	Mass action (reversible)	nan	nan	
11	11	r10	addintcuca -> cuco + prodnew2cu	Mass action (irreversible)	nan	nan	
12	12	r13	2 * cucarbenew = dimer	Mass action (reversible)	nan	nan	
13	13	r09	dimintnew = addintcuca	Mass action (reversible)	nan	nan	

343K-Copasi Parameter Overview:

New Model s compartment ml cu mmol/(ml) cuthf mmol/(ml) thf mmol/(ml) cudiazo mmol/(ml) diazo mmol/(ml) cucarbe mmol/(ml) n2 mmol/(ml) cucarbenew mmol/(ml) co mmol/(ml) prodhomo mmol/(ml) cuco mmol/(ml) prodnew2 mmol/(ml) intcarbenew mmol/(ml) prodnew2cu mmol/(ml) addhomo mmol/(ml) addintcuca mmol/(ml) dimer mmol/(ml) dimintnew mmol/(ml) (r01).k1 1/s (r01).k2 ml/(mmol*s) (r02).k1 ml/(mmol*s) (r02).k2 1/s (r03).k1 1/s (r03).k2 ml/(mmol*s) (r06).k1 1/s (r06).k2 1/s (r04).k1 ml/(mmol*s) (r04).k2 1/s (r05).k1 1/s (r07).k1 1/s (r07).k2 ml/(mmol*s) (r08).k1 ml/(mmol*s) (r08).k2 1/s (r12).k1 1/s (r12).k2 ml/(mmol*s) (r11).k1 1/s (r11).k2 ml/(mmol*s) (r10).k1 1/s (r13).k1 ml/(mmol*s) (r13).k2 1/s (r09).k1 1/s (r09).k2 1/s

0 1 0 0.0056 0 0 0.028 0 0 0 0 0 0 0 0 0 0 0 0 0 284 2.18e+10 2.18e+10 6840 767 0.07 9640 0.245 3.27e+06 5.15e+09 3.33e+09 695000 252 1.84e+06 2.18e+10 0.00155 2.18e+10 1.65e+07 2.18e+10 32300 3.32e+08 2.18e+10 2.18e+10 1.24e+07

318K-Copasi Parameter Overview:

New Model s compartment ml cu mmol/(ml) cuthf mmol/(ml) thf mmol/(ml) cudiazo mmol/(ml) diazo mmol/(ml) cucarbe mmol/(ml) n2 mmol/(ml) cucarbenew mmol/(ml) co mmol/(ml) prodhomo mmol/(ml) cuco mmol/(ml) prodnew2 mmol/(ml) intcarbenew mmol/(ml) prodnew2cu mmol/(ml) addhomo mmol/(ml) addintcuca mmol/(ml) dimer mmol/(ml) dimintnew mmol/(ml) (r01).k1 1/s (r01).k2 ml/(mmol*s) (r02).k1 ml/(mmol*s) (r02).k2 1/s (r03).k1 1/s (r03).k2 ml/(mmol*s) (r06).k1 1/s (r06).k2 1/s (r04).k1 ml/(mmol*s) (r04).k2 1/s (r05).k1 1/s (r07).k1 1/s (r07).k2 ml/(mmol*s) (r08).k1 ml/(mmol*s) (r08).k2 1/s (r12).k1 1/s (r12).k2 ml/(mmol*s) (r11).k1 1/s (r11).k2 ml/(mmol*s) (r10).k1 1/s (r13).k1 ml/(mmol*s) (r13).k2 1/s (r09).k1 1/s (r09).k2 1/s

0 1 0 0.0056 0 0 0.028 0 0 0 0 0 0 0 0 0 0 0 0 0 14.7 1.59e+10 1.59e+10 265 110 0.0166 2110 0.0238 1.19e+06 5.11e+08 2.09e+09 176000 110 7.62e+06 1.59e+10 3.07e-05 1.59e+10 870000 1.59e+10 6650 2.01e+09 1.59e+10 1.59e+10 4.45e+06

298K-Copasi Parameter Overview:

New Model s compartment ml cu mmol/(ml) cuthf mmol/(ml) thf mmol/(ml) cudiazo mmol/(ml) diazo mmol/(ml) cucarbe mmol/(ml) n2 mmol/(ml) cucarbenew mmol/(ml) co mmol/(ml) prodhomo mmol/(ml) cuco mmol/(ml) prodnew2 mmol/(ml) intcarbenew mmol/(ml) prodnew2cu mmol/(ml) addhomo mmol/(ml) addintcuca mmol/(ml) dimer mmol/(ml) dimintnew mmol/(ml) (r01).k1 1/s (r01).k2 ml/(mmol*s) (r02).k1 ml/(mmol*s) (r02).k2 1/s (r03).k1 1/s (r03).k2 ml/(mmol*s) (r06).k1 1/s (r06).k2 1/s (r04).k1 ml/(mmol*s) (r04).k2 1/s (r05).k1 1/s (r07).k1 1/s (r07).k2 ml/(mmol*s) (r08).k1 ml/(mmol*s) (r08).k2 1/s (r12).k1 1/s (r12).k2 ml/(mmol*s) (r11).k1 1/s (r11).k2 ml/(mmol*s) (r10).k1 1/s (r13).k1 ml/(mmol*s) (r13).k2 1/s (r09).k1 1/s (r09).k2 1/s

0 1 0 0.0056 0 0 0.028 0 0 0 0 0 0 0 0 0 0 0 0 0 0.949 1.19e+10 1.19e+10 13.1 18.6 0.00446 518 0.00278 474000 6.07e+07 2.52e+08 49800 52 2.88e+07 1.19e+10 8.26e-07 1.19e+10 62400 1.19e+10 1560 1.08e+10 1.19e+10 1.19e+10 1.75e+06

273K-Copasi Parameter Overview:

New Model s	compartment ml	cu mmol/(ml)	cuthf mmol/(ml)	thf mmol/(ml)	cudiazo mmol/(ml)	diazo mmol/(ml)	cucarbe mmol/(ml)	n2 mmol/(ml)	cucarbenew mmol/(ml)	co mmol/(ml)
0	1	0	0.0056	0	0	0.028	0	0	0	0

prodhomo mmol/(ml)	cuco mmol/(ml)	prodnew2 mmol/(ml)	intcarbenew mmol/(ml)	prodnew2cu mmol/(ml)	addhomo mmol/(ml)	addintcuca mmol/(ml)	dimer mmol/(ml)	dimintnew mmol/(ml)	(r01).k1 1/s	(r01).k2 ml/(mmol*s)
0	0	0	0	0	0	0	0	0	0.0171	7.9e+09

(r02).k1 ml/(mmol*s)	(r02).k2 1/s	(r03).k1 1/s	(r03).k2 ml/(mmol*s)	(r06).k1 1/s	(r06).k2 1/s	(r04).k1 ml/(mmol*s)	(r04).k2 1/s	(r05).k1 1/s	(r07).k1 1/s	(r07).k2 ml/(mmol*s)
7.9e+09	0.165	1.4	0.000664	67.4	1.21e-06	124000	2.72e+06	1.16e+08	7970	17.7

(r08).k1 ml/(mmol*s)	(r08).k2 1/s	(r12).k1 1/s	(r12).k2 ml/(mmol*s)	(r11).k1 1/s	(r11).k2 ml/(mmol*s)	(r10).k1 1/s	(r13).k1 ml/(mmol*s)	(r13).k2 1/s	(r09).k1 1/s	(r09).k2 1/s
2.01e+08	7.9e+09	4.26e-09	7.9e+09	1240	7.9e+09	190	7.9e+09	4.97e+08	7.9e+09	456000

243K-Copasi Parameter Overview:

New Model s	compartment ml	cu mmol/(ml)	cuthf mmol/(ml)	thf mmol/(ml)	cudiazo mmol/(ml)	diazo mmol/(ml)	cucarbe mmol/(ml)	n2 mmol/(ml)	cucarbenew mmol/(ml)	co mmol/(ml)
0	1	0	0.0056	0	0	0.028	0	0	0	0

prodhomo mmol/(ml)	cuco mmol/(ml)	prodnew2 mmol/(ml)	intcarbenew mmol/(ml)	prodnew2cu mmol/(ml)	addhomo mmol/(ml)	addintcuca mmol/(ml)	dimer mmol/(ml)	dimintnew mmol/(ml)	(r01).k1 1/s	(r01).k2 ml/(mmol*s)
0	0	0	0	0	0	0	0	0	4.43e-05	4.37e+09

(r02).k1 ml/(mmol*s)	(r02).k2 1/s	(r03).k1 1/s	(r03).k2 ml/(mmol*s)	(r06).k1 1/s	(r06).k2 1/s	(r04).k1 ml/(mmol*s)	(r04).k2 1/s	(r05).k1 1/s	(r07).k1 1/s	(r07).k2 ml/(mmol*s)
4.37e+09	0.000256	0.0312	4.09e-05	3.35	1.21e-06	17500	28000	3.72e+07	544	3.71

(r08).k1 ml/(mmol*s)	(r08).k2 1/s	(r12).k1 1/s	(r12).k2 ml/(mmol*s)	(r11).k1 1/s	(r11).k2 ml/(mmol*s)	(r10).k1 1/s	(r13).k1 ml/(mmol*s)	(r13).k2 1/s	(r09).k1 1/s	(r09).k2 1/s
3.67e+09	4.37e+09	1.83e-12	4.37e+09	4.43	4.37e+09	8.64	4.37e+09	3.96e+06	4.37e+09	64900

11. Energies and Coordinates of the Computed Structures

Potencial Energies (hartree) and Cartesian Coordinates (Å) of all relevant species in Toluene.

TOL_CuTHF

Energy (POTENTIAL) = -2179.53963349 Eh

	Atom	X	Y	Z
1	Cu	-0.0285	0.0535	-0.5534
2	N	-0.3465	1.8604	-1.5807
3	C	-0.6051	3.1447	-1.2944
4	C	-0.7509	2.9113	-3.4972
5	C	-0.8740	3.8555	-2.4858
6	H	-0.8611	2.9996	-4.5689
7	H	-1.1122	4.9046	-2.5850
8	N	1.5022	-0.4494	-1.9349
9	C	2.7622	-0.9042	-1.9044
10	C	3.2917	-0.9311	-3.2147
11	C	2.2590	-0.4706	-4.0230
12	H	4.2765	-1.2529	-3.5211
13	H	2.2039	-0.3297	-5.0934
14	N	-1.4605	-0.8826	-1.7811
15	C	-2.5122	-1.7043	-1.6529
16	C	-3.0360	-2.0128	-2.9286
17	C	-2.2336	-1.3120	-3.8200
18	H	-3.8844	-2.6417	-3.1566
19	H	-2.2678	-1.2368	-4.8978
20	N	-0.4336	1.7235	-2.9318
21	N	-1.2959	-0.6421	-3.1102
22	N	1.1968	-0.1882	-3.2323
23	H	-0.2895	0.4655	-4.8036
24	B	-0.2079	0.3464	-3.6102
25	C	-0.2085	-1.5175	2.1139
26	O	0.2372	-0.3203	1.4372
27	C	1.2996	0.2981	2.2179
28	C	1.2724	-0.3905	3.5957
29	C	-0.0582	-1.1699	3.5875
30	H	-1.2280	-1.7107	1.7882
31	H	0.4337	-2.3600	1.8293
32	H	2.2488	0.1534	1.6950
33	H	1.0647	1.3625	2.2668
34	H	2.1147	-1.0822	3.6893
35	H	1.3327	0.3329	4.4137
36	H	-0.0383	-2.0563	4.2283
37	H	-0.8905	-0.5301	3.9012
38	C	-0.5472	3.6176	0.1098
39	C	-1.7227	4.0194	0.7777
40	C	0.6934	3.6333	0.7860
41	C	-1.6384	4.4298	2.1154
42	C	0.7350	4.0625	2.1179
43	C	-0.4197	4.4636	2.8018
44	H	-2.5481	4.7257	2.6343
45	H	1.6918	4.0760	2.6368
46	C	3.3454	-1.3151	-0.6047
47	C	4.3974	-0.5749	-0.0223
48	C	2.7925	-2.4206	0.0774
49	C	4.8734	-0.9491	1.2402
50	C	3.2988	-2.7607	1.3393
51	C	4.3355	-2.0379	1.9386
52	H	5.6755	-0.3710	1.6954
53	H	2.8705	-3.6110	1.8662
54	C	-2.9749	-2.1061	-0.3023
55	C	-2.8391	-3.4406	0.1319
56	C	-3.5201	-1.1290	0.5634
57	C	-3.2476	-3.7793	1.4301
58	C	-3.9206	-1.5112	1.8489
59	C	-3.7885	-2.8299	2.3041
60	H	-3.1311	-4.8071	1.7678
61	H	-4.3364	-0.7585	2.5162
62	C	-3.0625	4.0122	0.0766
63	H	-3.1656	4.8757	-0.5921
64	H	-3.1931	3.1181	-0.5393
65	H	-3.8827	4.0552	0.7998
66	C	1.9588	3.1709	0.1005
67	H	1.9052	2.1046	-0.1405
68	H	2.1153	3.6953	-0.8489
69	H	2.8342	3.3384	0.7351
70	C	-0.3435	4.9503	4.2301
71	H	-0.1170	6.0241	4.2667
72	H	-1.2910	4.7990	4.7581
73	H	0.4442	4.4340	4.7896
74	C	1.6647	-3.2267	-0.5265
75	H	0.7394	-2.6432	-0.5709
76	H	1.8930	-3.5208	-1.5568
77	H	1.4750	-4.1341	0.0546
78	C	4.9853	0.6260	-0.7283
79	H	5.5413	0.3326	-1.6260
80	H	4.2031	1.3199	-1.0547
81	H	5.6721	1.1692	-0.0721
82	C	4.8647	-2.4099	3.3040
83	H	5.8948	-2.7830	3.2427
84	H	4.8782	-1.5440	3.9769
85	H	4.2574	-3.1909	3.7725
86	C	-3.6431	0.3148	0.1341
87	H	-2.6568	0.7861	0.0687
88	H	-4.0972	0.4031	-0.8587
89	H	-4.2482	0.8852	0.8448
90	C	-2.2366	-4.4940	-0.7699
91	H	-2.8908	-4.7178	-1.6201
92	H	-1.2798	-4.1616	-1.1871
93	H	-2.0664	-5.4272	-0.2245
94	C	-4.1882	-3.1971	3.7142
95	H	-3.4733	-2.7913	4.4421
96	H	-5.1732	-2.7901	3.9697
97	H	-4.2234	-4.2820	3.8547

TOL_CuCO

Energy (POTENTIAL) = -2060.38771653 Eh

	Atom	X	Y	Z
1	Cu	16.0485	-4.2959	6.0613
2	N	17.0698	-4.4294	7.8922
3	C	16.9291	-3.9097	9.1206
4	C	18.8267	-5.0664	9.0878
5	C	18.0270	-4.2956	9.9220
6	H	19.7675	-5.5670	9.2691
7	H	18.2019	-4.0447	10.9579
8	N	17.8867	-3.7688	5.2220
9	C	18.3620	-2.7352	4.5120
10	C	19.7569	-2.8842	4.3422
11	C	20.0761	-4.0597	5.0089
12	H	20.4253	-2.2232	3.8103
13	H	21.0232	-4.5605	5.1527
14	N	16.4580	-6.3206	5.7263
15	C	15.7556	-7.3892	5.3186
16	C	16.5836	-8.5300	5.3280
17	C	17.8203	-8.0700	5.7693
18	H	16.3128	-9.5386	5.0521
19	H	18.7542	-8.5890	5.9340
20	N	18.2344	-5.1325	7.8738
21	N	17.7202	-6.7424	6.0008
22	N	18.9393	-4.5750	5.5305
23	H	19.8147	-6.2884	6.7285
24	C	14.4388	-3.5285	5.7670
25	O	13.4067	-3.0671	5.5757
26	B	18.7632	-5.7395	6.5434
27	C	17.4648	-1.6328	4.0853
28	C	16.4515	-1.8557	3.1314
29	C	17.6135	-0.3550	4.6750
30	C	15.6179	-0.7900	2.7613
31	C	16.7601	0.6789	4.2784
32	C	15.7576	0.4839	3.3181
33	H	14.8408	-0.9651	2.0203
34	H	16.8729	1.6604	4.7350
35	C	14.3283	-7.2027	4.9582
36	C	13.3278	-7.4014	5.9303
37	C	13.9911	-6.7367	3.6691
38	C	12.0005	-7.0846	5.6090
39	C	12.6542	-6.4361	3.3876
40	C	11.6461	-6.5885	4.3495
41	H	11.2289	-7.2200	6.3641
42	H	12.3927	-6.0660	2.3980
43	C	15.7644	-3.0460	9.4353
44	C	15.9650	-1.6752	9.7072
45	C	14.4549	-3.5778	9.4128
46	C	14.8518	-0.8607	9.9555
47	C	13.3719	-2.7275	9.6616
48	C	13.5476	-1.3657	9.9357
49	H	15.0098	0.1962	10.1591
50	H	12.3644	-3.1389	9.6411
51	C	16.2234	-3.2213	2.5293
52	H	15.6536	-3.8526	3.2184
53	H	17.1627	-3.7443	2.3261
54	H	15.6567	-3.1510	1.5958
55	C	18.6610	-0.0999	5.7356
56	H	19.6736	-0.1048	5.3168
57	H	18.6367	-0.8719	6.5119
58	H	18.5024	0.8705	6.2156
59	C	14.8635	1.6285	2.9029
60	H	15.4486	2.4514	2.4743
61	H	14.3153	2.0359	3.7611
62	H	14.1291	1.3146	2.1546
63	C	15.0500	-6.5973	2.5992
64	H	15.3473	-7.5830	2.2192
65	H	15.9558	-6.1216	2.9829
66	H	14.6836	-6.0106	1.7521
67	C	13.6663	-7.9707	7.2890
68	H	14.5883	-7.5452	7.6935
69	H	13.8181	-9.0559	7.2272
70	H	12.8599	-7.7906	8.0057
71	C	10.2218	-6.1958	4.0349
72	H	9.5179	-6.6124	4.7623
73	H	9.9224	-6.5383	3.0379
74	H	10.1062	-5.1044	4.0509
75	C	14.1992	-5.0338	9.1024
76	H	14.9521	-5.6873	9.5526

77	H	14.2306	-5.2148	8.0232
78	H	13.2131	-5.3429	9.4617
79	C	17.3506	-1.0690	9.7154
80	H	17.9193	-1.3556	8.8247
81	H	17.9327	-1.4005	10.5830
82	H	17.2978	0.0235	9.7473
83	C	12.3557	-0.4798	10.2133
84	H	11.8520	-0.7757	11.1420
85	H	11.6136	-0.5468	9.4088
86	H	12.6485	0.5702	10.3126

TOL_TSCC_BS2
Energy (POTENTIAL) = -4508.05743488 Eh

	Atom	X	Y	Z
1	Cu	6.0874	0.4691	-1.3687
2	N	4.4918	0.9147	-0.0563
3	C	3.2622	1.4287	-0.2743
4	C	3.2757	0.5946	1.7895
5	C	2.4567	1.2394	0.8746
6	H	3.0899	0.2768	2.8037
7	H	1.4315	1.5470	1.0029
8	N	6.4696	2.4197	1.7858
9	C	7.3209	3.2398	2.4374
10	C	8.0550	2.5081	3.4108
11	C	7.5751	1.2088	3.3141
12	H	8.7972	2.8874	4.0953
13	H	7.8273	0.3218	3.8759
14	N	6.6899	-1.1656	-0.1952
15	C	7.3506	-2.3138	-0.4819
16	C	7.6651	-2.9921	0.7179
17	C	7.1314	-2.2042	1.7288
18	H	8.1729	-3.9387	0.8113
19	H	7.1039	-2.3546	2.7967
20	N	4.4908	0.4052	1.2077
21	N	6.5484	-1.1174	1.1588
22	N	6.6276	1.1881	2.3299
23	H	5.4156	-0.5777	2.9816
24	C	6.3326	1.6220	-2.9991
25	C	5.5376	-0.0127	-4.6092
26	H	6.2120	-0.6356	-4.0178
27	H	5.8465	-0.0883	-5.6517
28	B	5.7536	-0.0394	1.9548
29	C	4.0936	-0.4826	-4.4202
30	H	3.3912	0.0915	-5.0329
31	H	3.7915	-0.3799	-3.3747
32	H	4.0094	-1.5367	-4.7052
33	C	7.3636	4.6913	2.1362
34	C	6.1655	5.4554	2.1727
35	C	8.5829	5.3304	1.8075
36	C	6.2086	6.8147	1.8341
37	C	8.5827	6.6975	1.4850
38	C	7.4065	7.4553	1.4804
39	H	5.2877	7.3927	1.8612
40	H	9.5236	7.1751	1.2220
41	C	7.5359	-2.8366	-1.8571
42	C	6.3804	-3.1911	-2.6076
43	C	8.8212	-3.1312	-2.3712
44	C	6.5360	-3.8315	-3.8428
45	C	8.9293	-3.7699	-3.6195
46	C	7.8038	-4.1461	-4.3602
47	H	5.6491	-4.1250	-4.3981
48	H	9.9204	-3.9993	-4.0040
49	C	2.8629	2.1540	-1.5057
50	C	1.8481	1.6157	-2.3381
51	C	3.3959	3.4347	-1.7855
52	C	1.3656	2.3744	-3.4128

53	C	2.8675	4.1697	-2.8614
54	C	1.8502	3.6654	-3.6795
55	H	0.5891	1.9553	-4.0485
56	H	3.2700	5.1587	-3.0645
57	C	4.8523	4.8468	2.6191
58	H	4.9567	4.3673	3.6008
59	H	4.5042	4.0710	1.9344
60	H	4.0758	5.6150	2.6978
61	C	9.8918	4.5705	1.7597
62	H	9.7573	3.5712	1.3392
63	H	10.3243	4.4402	2.7606
64	H	10.6309	5.1034	1.1514
65	C	7.4122	8.9170	1.0939
66	H	6.8360	9.0874	0.1747
67	H	8.4293	9.2841	0.9206
68	H	6.9591	9.5414	1.8745
69	C	10.1021	-2.8117	-1.6256
70	H	10.6797	-3.7262	-1.4385
71	H	9.9213	-2.3319	-0.6633
72	H	10.7395	-2.1427	-2.2144
73	C	4.9895	-2.9590	-2.0562
74	H	4.7739	-1.8959	-1.9163
75	H	4.8727	-3.4279	-1.0710
76	H	4.2300	-3.3768	-2.7241
77	C	7.9435	-4.9166	-5.6541
78	H	7.9279	-5.9991	-5.4667
79	H	8.8862	-4.6886	-6.1628
80	H	7.1250	-4.6960	-6.3468
81	C	4.4865	4.0634	-0.9456
82	H	4.0749	4.8534	-0.3047
83	H	5.2401	4.5349	-1.5828
84	H	4.9968	3.3463	-0.3033
85	C	1.2664	0.2429	-2.0733
86	H	0.5200	0.2702	-1.2688
87	H	2.0340	-0.4740	-1.7644
88	H	0.7725	-0.1501	-2.9684
89	C	1.3200	4.4599	-4.8515
90	H	1.6385	5.5066	-4.8039
91	H	0.2241	4.4421	-4.8869
92	H	1.6785	4.0490	-5.8057
93	H	6.3046	2.6937	-2.7936
94	N	5.7320	1.3739	-4.1662
95	C	5.0105	2.4280	-4.9182
96	H	3.9917	2.4649	-4.5239
97	C	4.9670	2.2469	-6.4340
98	H	4.4859	3.1317	-6.8694
99	H	5.9660	2.1497	-6.8602
100	H	4.3726	1.3768	-6.7329
101	H	5.4965	3.3730	-4.6686
102	Cu	10.5281	2.5597	-4.8985
103	N	10.8998	2.0812	-7.0408
104	C	10.3081	1.4086	-8.0504
105	C	12.0096	2.5563	-8.9215
106	C	10.9862	1.6837	-9.2660
107	H	12.7778	3.0212	-9.5202
108	H	10.7588	1.2854	-10.2415
109	N	12.6760	2.3516	-4.5237
110	C	13.5255	1.7276	-3.6774
111	C	14.8440	1.7855	-4.1902
112	C	14.7376	2.4900	-5.3818
113	H	15.7351	1.3893	-3.7300
114	H	15.4870	2.7873	-6.0990
115	N	10.9232	4.6659	-5.3639
116	C	10.4614	5.8946	-5.0473
117	C	11.3031	6.8818	-5.6176
118	C	12.2867	6.1728	-6.2947

119	H	11.1856	7.9514	-5.5489
120	H	13.1282	6.5099	-6.8801
121	N	11.9299	2.7779	-7.5819
122	N	12.0359	4.8473	-6.1252
123	N	13.4317	2.8191	-5.5562
124	H	13.7373	4.0915	-7.3817
125	C	8.2986	1.3056	-3.1428
126	C	8.6355	2.0157	-4.5153
127	O	7.8282	2.1368	-5.3949
128	C	9.5444	0.5331	-1.2031
129	H	10.6093	0.4100	-1.4202
130	H	9.0312	-0.3751	-1.5149
131	B	12.8374	3.6619	-6.7074
132	C	9.3165	0.7413	0.2892
133	H	8.2763	0.9934	0.4931
134	H	9.9531	1.5296	0.6956
135	H	9.5552	-0.1823	0.8260
136	C	13.1450	1.2048	-2.3430
137	C	13.1474	-0.1844	-2.0844
138	C	12.9108	2.1222	-1.2887
139	C	12.9320	-0.6355	-0.7719
140	C	12.6988	1.6319	0.0075
141	C	12.7157	0.2568	0.2883
142	H	12.9239	-1.7046	-0.5765
143	H	12.5193	2.3371	0.8156
144	C	9.2200	6.1362	-4.2728
145	C	7.9581	5.8576	-4.8504
146	C	9.2932	6.7434	-2.9955
147	C	6.7933	6.2058	-4.1438
148	C	8.1095	7.0923	-2.3340
149	C	6.8492	6.8447	-2.8963
150	H	5.8250	6.0020	-4.5955
151	H	8.1692	7.5452	-1.3492
152	C	9.1540	0.5058	-7.8494
153	C	7.9623	0.7165	-8.5855
154	C	9.2373	-0.5821	-6.9464
155	C	6.8967	-0.1836	-8.4399
156	C	8.1436	-1.4503	-6.8218
157	C	6.9708	-1.2788	-7.5703
158	H	5.9839	-0.0146	-9.0063
159	H	8.2079	-2.2699	-6.1155
160	C	13.3578	-1.1769	-3.2072
161	H	14.3450	-1.0618	-3.6698
162	H	12.6211	-1.0329	-4.0074
163	H	13.2740	-2.2067	-2.8460
164	C	12.9142	3.6125	-1.5511
165	H	12.2083	3.8813	-2.3429
166	H	13.9027	3.9523	-1.8869
167	H	12.6524	4.1722	-0.6477
168	C	12.5017	-0.2457	1.6982
169	H	12.1140	-1.2703	1.7046
170	H	11.7930	0.3862	2.2452
171	H	13.4420	-0.2484	2.2669
172	C	10.6273	7.0021	-2.3282
173	H	11.1763	7.8208	-2.8094
174	H	11.2767	6.1206	-2.3752
175	H	10.4892	7.2672	-1.2747
176	C	7.8568	5.2474	-6.2318
177	H	8.4151	4.3118	-6.3048
178	H	8.2674	5.9274	-6.9907
179	H	6.8144	5.0395	-6.4941
180	C	5.5990	7.2798	-2.1654
181	H	5.5009	8.3741	-2.1726
182	H	5.6194	6.9620	-1.1169
183	H	4.6952	6.8690	-2.6256
184	C	10.4735	-0.8348	-6.1084

185	H	11.3941	-0.7139	-6.6900
186	H	10.4539	-1.8454	-5.6873
187	H	10.5424	-0.1245	-5.2751
188	C	7.7948	1.9154	-9.4956
189	H	8.3349	1.7968	-10.4429
190	H	8.1746	2.8292	-9.0256
191	H	6.7381	2.0709	-9.7391
192	C	5.8158	-2.2471	-7.4380
193	H	5.9490	-3.1189	-8.0935
194	H	4.8649	-1.7768	-7.7137
195	H	5.7246	-2.6206	-6.4128
196	H	8.3034	0.2353	-3.3616
197	N	9.0076	1.6105	-2.0387
198	C	9.3674	2.9917	-1.7413
199	H	10.1717	2.9616	-1.0053
200	C	8.2134	3.8579	-1.2402
201	H	8.6010	4.8004	-0.8507
202	H	7.5296	4.1090	-2.0526
203	H	7.6535	3.3605	-0.4446
204	H	9.7911	3.4351	-2.6580

TOL_INTCC2_BS2
Energy (POTENTIAL) = -4508.07893487 Eh

	Atom	X	Y	Z
1	Cu	-2.4732	-1.5578	1.6665
2	N	-3.8546	-1.2756	3.2198
3	C	-5.1107	-0.7931	3.1539
4	C	-4.7118	-1.3462	5.2725
5	C	-5.6959	-0.8201	4.4414
6	H	-4.7118	-1.5373	6.3346
7	H	-6.6901	-0.5034	4.7135
8	N	-1.5414	0.4187	4.6443
9	C	-0.6330	1.2710	5.1674
10	C	0.1810	0.5934	6.1168
11	C	-0.3069	-0.7056	6.1400
12	H	0.9785	1.0080	6.7127
13	H	-0.0123	-1.5576	6.7342
14	N	-1.6176	-3.1146	2.7290
15	C	-0.9348	-4.2097	2.3223
16	C	-0.3918	-4.8721	3.4480
17	C	-0.8139	-4.1290	4.5425
18	H	0.1877	-5.7810	3.4486
19	H	-0.6618	-4.2866	5.5989
20	N	-3.6172	-1.6178	4.5107
21	N	-1.5522	-3.0807	4.0888
22	N	-1.3375	-0.7785	5.2460
23	H	-2.3587	-2.5740	6.0935
24	C	-2.5346	-0.4244	0.1138
25	C	-2.8803	-2.0557	-1.6305
26	H	-2.4106	-2.6768	-0.8684
27	H	-2.2836	-2.1421	-2.5390
28	B	-2.2304	-2.0292	5.0238
29	C	-4.3229	-2.4920	-1.8728
30	H	-4.7971	-1.9095	-2.6704
31	H	-4.9189	-2.3700	-0.9639
32	H	-4.3427	-3.5467	-2.1679
33	C	-0.5961	2.7012	4.7780
34	C	-1.7887	3.4771	4.8115
35	C	0.6170	3.3194	4.3896
36	C	-1.7480	4.8178	4.4071
37	C	0.6141	4.6704	4.0022
38	C	-0.5562	5.4362	3.9946
39	H	-2.6640	5.4031	4.4374
40	H	1.5518	5.1304	3.6994
41	C	-0.9012	-4.6750	0.9160
42	C	-2.1118	-5.0138	0.2571
43	C	0.3380	-4.8989	0.2633
44	C	-2.0556	-5.5863	-1.0239
45	C	0.3458	-5.4656	-1.0201
46	C	-0.8385	-5.8362	-1.6741
47	H	-2.9848	-5.8693	-1.5122
48	H	1.3006	-5.6424	-1.5100
49	C	-5.6699	-0.3363	1.8576
50	C	-6.5584	-1.1709	1.1399
51	C	-5.2833	0.9147	1.3226
52	C	-7.0578	-0.7353	-0.0960
53	C	-5.8039	1.3135	0.0808
54	C	-6.6906	0.5043	-0.6425
55	H	-7.7378	-1.3795	-0.6484
56	H	-5.5078	2.2768	-0.3278
57	C	-3.0852	2.9081	5.3492
58	H	-2.9498	2.5362	6.3735
59	H	-3.4389	2.0601	4.7620
60	H	-3.8672	3.6744	5.3683
61	C	1.9361	2.5760	4.3771
62	H	1.8062	1.5203	4.1339
63	H	2.4315	2.6178	5.3568
64	H	2.6245	3.0184	3.6494
65	C	-0.5457	6.8877	3.5719
66	H	-1.2112	7.0616	2.7173
67	H	0.4583	7.2154	3.2833
68	H	-0.8889	7.5425	4.3836
69	C	1.6624	-4.5792	0.9247
70	H	2.0499	-5.4455	1.4781
71	H	1.5865	-3.7558	1.6361
72	H	2.4136	-4.3095	0.1760
73	C	-3.4620	-4.7950	0.9073
74	H	-3.7470	-3.7356	0.8953
75	H	-3.4628	-5.0998	1.9592
76	H	-4.2425	-5.3562	0.3833
77	C	-0.7902	-6.5146	-3.0248
78	H	-0.2889	-7.4888	-2.9553
79	H	-0.2327	-5.9194	-3.7580
80	H	-1.7938	-6.6866	-3.4262
81	C	-4.3357	1.8237	2.0724
82	H	-4.8387	2.2977	2.9246
83	H	-3.9591	2.6236	1.4266
84	H	-3.4795	1.2752	2.4745
85	C	-6.9351	-2.5346	1.6775
86	H	-7.4637	-2.4627	2.6352
87	H	-6.0430	-3.1477	1.8585
88	H	-7.5802	-3.0718	0.9749
89	C	-7.2048	0.9291	-1.9996
90	H	-7.0330	1.9956	-2.1794
91	H	-8.2797	0.7382	-2.1018
92	H	-6.7042	0.3743	-2.8062
93	H	-2.5024	0.6567	0.2753
94	N	-2.7842	-0.6670	-1.1465
95	C	-3.1102	0.4214	-2.1127
96	H	-4.1970	0.5566	-2.0869
97	C	-2.6329	0.1745	-3.5371
98	H	-2.8025	1.0846	-4.1233
99	H	-1.5647	-0.0412	-3.5754
100	H	-3.1753	-0.6414	-4.0248
101	H	-2.6446	1.3279	-1.7212
102	Cu	2.6530	1.0864	-2.2438
103	N	3.1136	0.3998	-4.3129
104	C	2.5209	-0.3103	-5.2951
105	C	4.3694	0.5975	-6.1491
106	C	3.2898	-0.2093	-6.4834
107	H	5.2120	0.9328	-6.7342
108	H	3.0842	-0.6755	-7.4333
109	N	4.7216	0.5601	-1.7732
110	C	5.4162	-0.1648	-0.8681
111	C	6.7689	-0.2708	-1.2733
112	C	6.8451	0.4360	-2.4653
113	H	7.5645	-0.7735	-0.7473
114	H	7.6795	0.6320	-3.1209
115	N	3.3796	3.0753	-2.8042
116	C	3.0518	4.3625	-2.5650
117	C	4.0328	5.2153	-3.1242
118	C	4.9603	4.3626	-3.7139
119	H	4.0448	6.2935	-3.1027
120	H	5.8670	4.5709	-4.2613
121	N	4.2339	0.9502	-4.8425
122	N	4.5423	3.0853	-3.5059
123	N	5.6083	0.9248	-2.7410
124	H	6.1918	2.0328	-4.6092
125	C	0.5779	0.0385	-0.9791
126	C	0.6757	1.0991	-1.9341
127	O	-0.1267	1.7862	-2.5355
128	C	1.1106	-1.0585	1.1013
129	H	2.1769	-1.2516	0.9483
130	H	0.5473	-1.8844	0.6575
131	B	5.2053	1.7734	-3.9700
132	C	0.7837	-0.9740	2.5832
133	H	-0.2513	-0.6756	2.7482
134	H	1.4319	-0.2744	3.1100
135	H	0.9268	-1.9573	3.0381
136	C	4.8573	-0.6601	0.4110
137	C	4.8149	-2.0469	0.6841
138	C	4.4858	0.2692	1.4145
139	C	4.4200	-2.4809	1.9604
140	C	4.1193	-0.2044	2.6812
141	C	4.0852	-1.5762	2.9769
142	H	4.3768	-3.5475	2.1636
143	H	3.8521	0.5111	3.4538
144	C	1.7994	4.7136	-1.8526
145	C	0.5767	4.6945	-2.5607
146	C	1.8192	5.0346	-0.4764
147	C	-0.6137	4.9624	-1.8695
148	C	0.6077	5.2957	0.1801
149	C	-0.6198	5.2506	-0.4977
150	H	-1.5565	4.9193	-2.4093
151	H	0.6183	5.5099	1.2446
152	C	1.2887	-1.1034	-5.0860
153	C	0.1662	-0.8948	-5.9283
154	C	1.2433	-2.1200	-4.0991
155	C	-0.9486	-1.7350	-5.8066
156	C	0.0889	-2.9134	-3.9856
157	C	-1.0041	-2.7572	-4.8496
158	H	-1.8010	-1.5751	-6.4627
159	H	0.0548	-3.6784	-3.2157
160	C	5.1774	-3.0622	-0.3785
161	H	6.2481	-3.0407	-0.6135
162	H	4.6467	-2.8659	-1.3172
163	H	4.9314	-4.0774	-0.0515
164	C	4.4907	1.7535	1.1272
165	H	3.7824	2.0016	0.3303
166	H	5.4745	2.0946	0.7814
167	H	4.2223	2.3289	2.0178
168	C	3.6906	-2.0582	4.3545
169	H	3.3583	-3.1019	4.3351
170	H	2.8765	-1.4550	4.7713
171	H	4.5341	-1.9948	5.0557
172	C	3.1239	5.1049	0.2876
173	H	3.6620	6.0380	0.0719
174	H	3.7972	4.2855	0.0200

175	H	2.9490	5.0686	1.3682
176	C	0.5524	4.3703	-4.0369
177	H	1.0000	3.3900	-4.2257
178	H	1.1188	5.1097	-4.6186
179	H	-0.4738	4.3494	-4.4169
180	C	-1.9160	5.5005	0.2410
181	H	-2.1314	6.5751	0.3242
182	H	-1.8794	5.0938	1.2578
183	H	-2.7648	5.0399	-0.2772
184	C	2.4210	-2.4031	-3.1892
185	H	3.3526	-2.5015	-3.7590
186	H	2.2583	-3.3281	-2.6265
187	H	2.5909	-1.5938	-2.4715
188	C	0.1367	0.2309	-6.9408
189	H	0.7162	-0.0057	-7.8415
190	H	0.5569	1.1525	-6.5241
191	H	-0.8912	0.4378	-7.2572
192	C	-2.2115	-3.6645	-4.7605
193	H	-2.1899	-4.4306	-5.5476
194	H	-3.1454	-3.1031	-4.8863
195	H	-2.2547	-4.1836	-3.7992
196	H	0.5838	-0.9633	-1.4033
197	N	0.7295	0.1507	0.3405
198	C	0.8642	1.4941	0.9201
199	H	1.4881	1.4157	1.8095
200	C	-0.4723	2.1520	1.2553
201	H	-0.2978	3.1270	1.7147
202	H	-1.0587	2.3091	0.3454
203	H	-1.0439	1.5416	1.9590
204	H	1.4178	2.1062	0.1912

TOL_INTCC1_BS2

Energy (POTENTIAL) = -4508.06938374 Eh

	Atom	X	Y	Z
1	Cu	-2.4753	-1.4453	1.6721
2	N	-3.8458	-1.2188	3.2580
3	C	-5.1064	-0.7448	3.2368
4	C	-4.6569	-1.3659	5.3261
5	C	-5.6635	-0.8214	4.5349
6	H	-4.6309	-1.5917	6.3811
7	H	-6.6536	-0.5209	4.8390
8	N	-1.5585	0.4635	4.6450
9	C	-0.6330	1.3232	5.1228
10	C	0.2713	0.6374	5.9799
11	C	-0.1809	-0.6753	5.9958
12	H	1.1055	1.0521	6.5234
13	H	0.1852	-1.5383	6.5317
14	N	-1.6229	-3.0568	2.6753
15	C	-0.9666	-4.1551	2.2345
16	C	-0.4113	-4.8476	3.3361
17	C	-0.7964	-4.1180	4.4528
18	H	0.1533	-5.7654	3.3062
19	H	-0.6240	-4.2963	5.5027
20	N	-3.5772	-1.6008	4.5316
21	N	-1.5258	-3.0494	4.0336
22	N	-1.2780	-0.7471	5.1846
23	H	-2.2603	-2.5546	6.0685
24	C	-2.5634	-0.2110	0.1981
25	C	-2.9180	-1.7176	-1.6497
26	H	-2.2914	-2.3504	-1.0184
27	H	-2.4675	-1.6877	-2.6412
28	B	-2.1711	-2.0046	4.9971
29	C	-4.3424	-2.2672	-1.6956
30	H	-4.9909	-1.6845	-2.3570
31	H	-4.7869	-2.2550	-0.6972
32	H	-4.3234	-3.3006	-2.0591
33	C	-0.6701	2.7628	4.7671
34	C	-1.8762	3.5021	4.9204
35	C	0.4813	3.4179	4.2690
36	C	-1.9177	4.8416	4.5114
37	C	0.3986	4.7665	3.8835
38	C	-0.7941	5.4905	3.9750
39	H	-2.8458	5.3973	4.6246
40	H	1.2869	5.2534	3.4885
41	C	-0.9688	-4.5978	0.8210
42	C	-2.2027	-4.8813	0.1781
43	C	0.2499	-4.8599	0.1449
44	C	-2.1906	-5.4302	-1.1135
45	C	0.2137	-5.4075	-1.1467
46	C	-0.9944	-5.7181	-1.7872
47	H	-3.1375	-5.6705	-1.5904
48	H	1.1528	-5.6188	-1.6534
49	C	-5.6909	-0.2254	1.9758
50	C	-6.5593	-1.0326	1.2092
51	C	-5.3437	1.0718	1.5258
52	C	-7.0727	-0.5318	0.0017
53	C	-5.8852	1.5394	0.3199
54	C	-6.7441	0.7499	-0.4607
55	H	-7.7340	-1.1588	-0.5912
56	H	-5.6304	2.5421	-0.0164
57	C	-3.0992	2.8923	5.5746
58	H	-2.8668	2.5569	6.5941
59	H	-3.4585	2.0144	5.0367
60	H	-3.9115	3.6239	5.6386
61	C	1.8060	2.7035	4.1049
62	H	1.6674	1.6743	3.7689
63	H	2.3654	2.6613	5.0490
64	H	2.4362	3.2210	3.3743
65	C	-0.8815	6.9257	3.5071
66	H	-1.5271	7.0185	2.6232
67	H	0.1036	7.3229	3.2396
68	H	-1.3063	7.5772	4.2813
69	C	1.5998	-4.6036	0.7842
70	H	2.0062	-5.5200	1.2338
71	H	1.5507	-3.8518	1.5718
72	H	2.3244	-4.2615	0.0373
73	C	-3.5292	-4.6482	0.8703
74	H	-3.7616	-3.5804	0.9506
75	H	-3.5235	-5.0363	1.8952
76	H	-4.3453	-5.1302	0.3227
77	C	-0.9987	-6.3856	-3.1443
78	H	-0.6127	-7.4115	-3.0765
79	H	-0.3657	-5.8528	-3.8634
80	H	-2.0085	-6.4411	-3.5629
81	C	-4.4152	1.9488	2.3337
82	H	-4.9077	2.3042	3.2469
83	H	-4.1025	2.8283	1.7619
84	H	-3.5183	1.4082	2.6507
85	C	-6.9091	-2.4322	1.6651
86	H	-7.4814	-2.4214	2.6007
87	H	-6.0060	-3.0238	1.8588
88	H	-7.5054	-2.9566	0.9113
89	C	-7.2556	1.2589	-1.7895
90	H	-7.5775	2.3050	-1.7246
91	H	-8.1029	0.6669	-2.1508
92	H	-6.4702	1.2117	-2.5582
93	H	-2.5347	0.8581	0.4439
94	N	-2.8440	-0.3631	-1.0703
95	C	-3.1749	0.8275	-1.9205
96	H	-4.0764	1.2694	-1.4883
97	C	-3.3981	0.5732	-3.4083
98	H	-3.6248	1.5368	-3.8812
99	H	-2.5153	0.1642	-3.9035
100	H	-4.2488	-0.0886	-3.5959
101	H	-2.3557	1.5389	-1.7879
102	Cu	2.8940	0.8545	-2.2060
103	N	3.3848	0.3604	-4.2746
104	C	2.8768	-0.3594	-5.2962
105	C	4.7474	0.6067	-6.0230
106	C	3.7131	-0.2296	-6.4311
107	H	5.6118	0.9745	-6.5543
108	H	3.5757	-0.6861	-7.3982
109	N	4.9675	0.5359	-1.6461
110	C	5.6881	-0.1231	-0.7147
111	C	7.0596	-0.0991	-1.0667
112	C	7.1153	0.6201	-2.2547
113	H	7.8789	-0.5283	-0.5123
114	H	7.9533	0.8989	-2.8750
115	N	3.3635	2.8885	-2.6771
116	C	2.8444	4.1197	-2.4975
117	C	3.7318	5.0905	-3.0232
118	C	4.8007	4.3638	-3.5352
119	H	3.5925	6.1596	-3.0362
120	H	5.6981	4.6864	-4.0407
121	N	4.5224	0.9464	-4.7258
122	N	4.5552	3.0442	-3.3144
123	N	5.8478	0.9908	-2.5780
124	H	6.3514	2.2177	-4.3959
125	C	1.3634	-0.3260	-1.1973
126	C	0.4641	-0.0312	-2.1659
127	O	-0.3479	0.3495	-2.9115
128	C	1.0760	-1.1815	1.0022
129	H	2.1072	-1.5635	0.9163
130	H	0.4052	-1.9567	0.6162
131	B	5.3860	1.8286	-3.7919
132	C	0.7645	-0.9510	2.4731
133	H	-0.2510	-0.5875	2.6268
134	H	1.4561	-0.2461	2.9346
135	H	0.8602	-1.9011	3.0048
136	C	5.0870	-0.6590	0.5302
137	C	4.9730	-2.0516	0.7414
138	C	4.6847	0.2468	1.5419
139	C	4.4739	-2.5178	1.9685
140	C	4.1952	-0.2588	2.7540
141	C	4.0884	-1.6381	2.9897
142	H	4.3805	-3.5891	2.1270
143	H	3.8868	0.4371	3.5299
144	C	1.5039	4.3161	-1.8974
145	C	0.3547	3.8930	-2.6058
146	C	1.3683	4.9429	-0.6353
147	C	-0.9114	4.1001	-2.0328
148	C	0.0871	5.1458	-0.1077
149	C	-1.0650	4.7225	-0.7867
150	H	-1.7942	3.7744	-2.5776
151	H	-0.0149	5.6040	0.8722
152	C	1.6180	-1.1212	-5.1330
153	C	0.4640	-0.7370	-5.8570
154	C	1.5536	-2.2086	-4.2286
155	C	-0.7239	-1.4611	-5.6850
156	C	0.3354	-2.8892	-4.0627
157	C	-0.8068	-2.5462	-4.8002
158	H	-1.6059	-1.1672	-6.2491
159	H	0.2841	-3.7029	-3.3465
160	C	5.3665	-3.0337	-0.3409
161	H	6.4379	-2.9794	-0.5685
162	H	4.8374	-2.8276	-1.2792
163	H	5.1393	-4.0620	-0.0416
164	C	4.7941	1.7412	1.3253

165	H	4.2219	2.0602	0.4478
166	H	5.8345	2.0423	1.1465
167	H	4.4252	2.2915	2.1961
168	C	3.5698	-2.1555	4.3124
169	H	3.1480	-3.1616	4.2126
170	H	2.7882	-1.5032	4.7170
171	H	4.3729	-2.2072	5.0609
172	C	2.5876	5.3454	0.1660
173	H	3.1682	6.1313	-0.3309
174	H	3.2662	4.4948	0.3070
175	H	2.2997	5.7150	1.1553
176	C	0.4750	3.2490	-3.9712
177	H	1.0992	2.3517	-3.9413
178	H	0.9433	3.9338	-4.6906
179	H	-0.5077	2.9646	-4.3589
180	C	-2.4255	4.8942	-0.1504
181	H	-2.6607	5.9524	0.0229
182	H	-2.4631	4.3924	0.8252
183	H	-3.2202	4.4744	-0.7772
184	C	2.7827	-2.6914	-3.4840
185	H	3.4740	-3.2036	-4.1676
186	H	2.5055	-3.4012	-2.6980
187	H	3.3463	-1.8707	-3.0323
188	C	0.4873	0.4595	-6.7834
189	H	1.0785	0.2671	-7.6869
190	H	0.9349	1.3319	-6.2938
191	H	-0.5258	0.7289	-7.0992
192	C	-2.0853	-3.3466	-4.6798
193	H	-2.1172	-4.1445	-5.4345
194	H	-2.9700	-2.7192	-4.8391
195	H	-2.1738	-3.8218	-3.6988
196	H	2.0031	-1.1976	-1.3539
197	N	0.9191	-0.0058	0.1297
198	C	1.2690	1.3200	0.6639
199	H	2.0533	1.2318	1.4248
200	C	0.0479	2.0490	1.2158
201	H	0.3257	3.0320	1.6005
202	H	-0.6817	2.1998	0.4149
203	H	-0.4339	1.4940	2.0233
204	H	1.6967	1.9094	-0.1523

TOL_INTCC3_BS2

Energy (POTENTIAL) = -4508.12963890 Eh

	Atom	X	Y	Z
1	Cu	-2.3596	-1.3364	1.6521
2	N	-3.7381	-0.8238	3.0877
3	C	-4.8935	-0.1283	3.0421
4	C	-4.6655	-0.8994	5.1156
5	C	-5.5147	-0.1520	4.3120
6	H	-4.7291	-1.1611	6.1603
7	H	-6.4505	0.3099	4.5829
8	N	-1.5424	0.5230	4.8269
9	C	-0.5010	1.2601	5.2679
10	C	0.4751	0.4090	5.8591
11	C	-0.0527	-0.8717	5.7467
12	H	1.4118	0.6916	6.3123
13	H	0.3406	-1.8245	6.0682
14	N	-1.7195	-3.0831	2.7114
15	C	-1.1614	-4.2637	2.3542
16	C	-0.8299	-5.0033	3.5134
17	C	-1.2354	-4.2092	4.5775
18	H	-0.3884	-5.9863	3.5501
19	H	-1.2022	-4.3849	5.6416
20	N	-3.6091	-1.2995	4.3601
21	N	-1.7663	-3.0640	4.0732
22	N	-1.2595	-0.7675	5.1133
23	H	-2.5746	-2.3720	6.0165
24	C	-2.2117	-0.3040	-0.3761
25	C	-3.0775	-2.1220	-1.7381
26	H	-2.7918	-2.7739	-0.9088
27	H	-2.2364	-2.1423	-2.4510
28	B	-2.3173	-1.8969	4.9355
29	C	-4.3372	-2.6905	-2.3865
30	H	-4.5967	-2.1767	-3.3190
31	H	-5.1920	-2.6189	-1.7051
32	H	-4.1773	-3.7465	-2.6278
33	C	-0.5001	2.7250	5.0395
34	C	-1.6710	3.4843	5.3155
35	C	0.6228	3.3753	4.4673
36	C	-1.7129	4.8397	4.9594
37	C	0.5378	4.7367	4.1322
38	C	-0.6261	5.4833	4.3495
39	H	-2.6148	5.4095	5.1711
40	H	1.4019	5.2193	3.6815
41	C	-1.0355	-4.7369	0.9534
42	C	-2.2081	-5.0436	0.2169
43	C	0.2360	-5.0135	0.3938
44	C	-2.0882	-5.6369	-1.0485
45	C	0.3108	-5.5964	-0.8815
46	C	-0.8374	-5.9398	-1.6084
47	H	-2.9913	-5.8935	-1.5961
48	H	1.2905	-5.8158	-1.2999
49	C	-5.3988	0.5394	1.8184
50	C	-6.1023	-0.2050	0.8448
51	C	-5.2371	1.9360	1.6671
52	C	-6.6595	0.4637	-0.2535
53	C	-5.7883	2.5654	0.5397
54	C	-6.5079	1.8471	-0.4265
55	H	-7.2097	-0.1088	-0.9964
56	H	-5.6601	3.6381	0.4204
57	C	-2.8708	2.8755	6.0117
58	H	-2.5657	2.2910	6.8878
59	H	-3.4134	2.1867	5.3602
60	H	-3.5613	3.6582	6.3445
61	C	1.9142	2.6453	4.1655
62	H	1.7331	1.6801	3.6858
63	H	2.4953	2.4472	5.0752
64	H	2.5453	3.2413	3.4983
65	C	-0.7201	6.9315	3.9250
66	H	-1.3063	7.0388	3.0016
67	H	0.2697	7.3613	3.7363
68	H	-1.2131	7.5442	4.6894
69	C	1.5238	-4.7533	1.1476
70	H	1.8817	-5.6682	1.6397
71	H	1.4063	-3.9971	1.9247
72	H	2.3152	-4.4225	0.4659
73	C	-3.5814	-4.7931	0.8028
74	H	-3.7159	-3.7465	1.0905
75	H	-3.7359	-5.3834	1.7155
76	H	-4.3688	-5.0569	0.0898
77	C	-0.7215	-6.6655	-2.9301
78	H	-0.3543	-7.6896	-2.7797
79	H	-0.0177	-6.1694	-3.6082
80	H	-1.6871	-6.7341	-3.4401
81	C	-4.4915	2.7418	2.7077
82	H	-5.0405	2.7682	3.6567
83	H	-4.3447	3.7743	2.3786
84	H	-3.5097	2.3096	2.9276
85	C	-6.2374	-1.7041	0.9783
86	H	-6.6141	-1.9924	1.9668
87	H	-5.2626	-2.1903	0.8567
88	H	-6.9155	-2.1120	0.2218
89	C	-7.0507	2.5335	-1.6590
90	H	-7.2837	3.5866	-1.4672
91	H	-7.9607	2.0449	-2.0250
92	H	-6.3152	2.5063	-2.4766
93	H	-2.3153	0.7501	-0.1479
94	N	-3.2612	-0.7861	-1.1810
95	C	-3.8654	0.2295	-2.0520
96	H	-4.8598	-0.1195	-2.3437
97	C	-3.0475	0.5943	-3.2978
98	H	-3.5768	1.3594	-3.8818
99	H	-2.0659	0.9877	-3.0235
100	H	-2.8853	-0.2723	-3.9481
101	H	-4.0315	1.1205	-1.4394
102	Cu	2.8825	0.8194	-2.3412
103	N	3.4135	0.2882	-4.4210
104	C	2.8968	-0.4449	-5.4285
105	C	4.6463	0.6774	-6.2371
106	C	3.6547	-0.2251	-6.6060
107	H	5.4501	1.1188	-6.8063
108	H	3.4988	-0.6731	-7.5740
109	N	4.9212	0.6289	-1.8295
110	C	5.6662	0.0107	-0.8905
111	C	7.0337	0.0760	-1.2509
112	C	7.0600	0.7765	-2.4500
113	H	7.8693	-0.3125	-0.6915
114	H	7.8856	1.0748	-3.0776
115	N	3.2575	2.8501	-2.8489
116	C	2.6707	4.0437	-2.6303
117	C	3.4755	5.0697	-3.1808
118	C	4.5659	4.4134	-3.7444
119	H	3.2750	6.1290	-3.1737
120	H	5.4225	4.7953	-4.2785
121	N	4.4736	0.9693	-4.9201
122	N	4.4096	3.0802	-3.5297
123	N	5.7817	1.0996	-2.7754
124	H	6.2453	2.3306	-4.6087
125	C	-1.0104	-0.9415	-0.0981
126	C	1.1254	0.1817	-2.2549
127	O	0.0911	-0.0897	-2.6612
128	C	1.1949	-1.1130	0.9469
129	H	2.1686	-0.6940	0.6681
130	H	1.1381	-2.1072	0.4927
131	B	5.2969	1.9034	-4.0054
132	C	1.0929	-1.2259	2.4706
133	H	0.0730	-1.4524	2.7804
134	H	1.4037	-0.3038	2.9644
135	H	1.7413	-2.0243	2.8398
136	C	5.1131	-0.5091	0.3802
137	C	5.1849	-1.8871	0.6844
138	C	4.6196	0.4066	1.3434
139	C	4.8123	-2.3228	1.9661
140	C	4.2786	-0.0663	2.6173
141	C	4.3875	-1.4240	2.9542
142	H	4.8662	-3.3833	2.1991
143	H	3.9179	0.6374	3.3623
144	C	1.3610	4.1283	-1.9414
145	C	0.1954	3.6590	-2.5973
146	C	1.2734	4.6624	-0.6357
147	C	-1.0353	3.7314	-1.9292
148	C	0.0220	4.7165	-0.0018
149	C	-1.1415	4.2541	-0.6299
150	H	-1.9303	3.3689	-2.4292
151	H	-0.0408	5.1106	1.0090
152	C	1.7082	-1.3051	-5.2253
153	C	0.5216	-1.0379	-5.9525
154	C	1.7353	-2.3650	-4.2862

155	C	-0.6017	-1.8505	-5.7500
156	C	0.5847	-3.1488	-4.1078
157	C	-0.5880	-2.9174	-4.8403
158	H	-1.5097	-1.6431	-6.3112
159	H	0.6034	-3.9487	-3.3728
160	C	5.6556	-2.8872	-0.3501
161	H	6.7298	-2.7923	-0.5509
162	H	5.1424	-2.7427	-1.3080
163	H	5.4701	-3.9133	-0.0161
164	C	4.4524	1.8731	1.0078
165	H	3.6799	2.0165	0.2429
166	H	5.3732	2.3064	0.5989
167	H	4.1640	2.4466	1.8936
168	C	4.0516	-1.8990	4.3493
169	H	3.7922	-2.9634	4.3607
170	H	3.2074	-1.3409	4.7686
171	H	4.9039	-1.7617	5.0291
172	C	2.5057	5.1668	0.0839
173	H	2.8960	6.0830	-0.3766
174	H	3.3165	4.4303	0.0575
175	H	2.2816	5.3900	1.1319
176	C	0.2652	3.1120	-4.0083
177	H	0.9932	2.2999	-4.0950
178	H	0.5801	3.8901	-4.7165
179	H	-0.7087	2.7332	-4.3334
180	C	-2.4766	4.2930	0.0769
181	H	-3.1846	4.9525	-0.4426
182	H	-2.3733	4.6517	1.1053
183	H	-2.9372	3.2993	0.1193
184	C	2.9593	-2.6518	-3.4414
185	H	3.8891	-2.5140	-4.0029
186	H	2.9333	-3.6762	-3.0548
187	H	3.0122	-1.9730	-2.5798
188	C	0.4335	0.1283	-6.9144
189	H	1.0153	-0.0444	-7.8279
190	H	0.8206	1.0491	-6.4626
191	H	-0.6047	0.3072	-7.2127
192	C	-1.7983	-3.8092	-4.6799
193	H	-1.7385	-4.6767	-5.3516
194	H	-2.7235	-3.2754	-4.9216
195	H	-1.8832	-4.1894	-3.6588
196	H	-0.8345	-1.9497	-0.4626
197	N	0.1587	-0.2991	0.3110
198	C	0.1669	1.1418	0.5574
199	H	1.2187	1.4374	0.6424
200	C	-0.6021	1.6249	1.7900
201	H	-0.4769	2.7040	1.9072
202	H	-1.6734	1.4177	1.7278
203	H	-0.2476	1.1403	2.6948
204	H	-0.2226	1.6384	-0.3382

TOL_CO_BS2

Energy (POTENTIAL) = -113.310797768 Eh

	Atom	X	Y	Z
1	C	2.3469	-0.0678	0.0000
2	O	3.4840	-0.0678	0.0000

TOL_metallocarbene7_BS2

Energy (POTENTIAL) = -2196.31894013 Eh

	Atom	X	Y	Z
1	Cu	8.4812	10.5155	8.4915
2	N	8.2418	8.2849	6.5133
3	N	7.1994	13.0894	9.7101
4	N	7.0048	12.2167	8.6943
5	N	9.7255	13.1334	9.4189
6	N	9.9865	12.0821	8.5948
7	N	8.6808	11.7516	11.2680
8	N	8.5640	10.5567	10.6357
9	C	7.7277	9.1870	7.3240
10	H	6.6270	9.1198	7.2793
11	C	9.7033	8.1268	6.3755
12	H	9.9450	8.1187	5.3003
13	H	10.1574	9.0134	6.8271
14	C	10.2053	6.8481	7.0557
15	H	9.7630	5.9471	6.5992
16	H	9.9568	6.8592	8.1270
17	H	11.2998	6.7866	6.9463
18	C	7.3928	7.3554	5.7121
19	H	6.6596	7.9724	5.1728
20	H	8.0390	6.8552	4.9761
21	C	6.6775	6.3269	6.5958
22	H	6.0522	5.6681	5.9698
23	H	6.0271	6.8383	7.3209
24	H	7.3999	5.7105	7.1505
25	C	6.0315	13.7349	10.0236
26	H	5.9957	14.4764	10.8144
27	C	5.0389	13.2535	9.1642
28	H	3.9990	13.5540	9.1213
29	C	5.6996	12.2962	8.3419
30	C	5.1921	11.4162	7.2623
31	C	4.1345	10.4976	7.5287
32	C	3.4471	10.4561	8.8833
33	H	4.1789	10.5091	9.7035
34	H	2.8615	9.5301	8.9919
35	H	2.7569	11.3068	9.0148
36	C	3.7413	9.5866	6.5275
37	H	2.9382	8.8784	6.7450
38	C	4.3629	9.5625	5.2610
39	C	3.9305	8.5610	4.2053
40	H	4.0206	7.5243	4.5746
41	H	4.5414	8.6548	3.2939
42	H	2.8745	8.7087	3.9197
43	C	5.4001	10.4820	5.0122
44	H	5.8933	10.4793	4.0374
45	C	5.8312	11.4053	5.9893
46	C	6.9987	12.3220	5.6695
47	H	6.8642	13.3224	6.1081
48	H	7.1258	12.4212	4.5796
49	H	7.9310	11.9138	6.0942
50	C	10.6249	14.1425	9.2064
51	H	10.5784	15.0590	9.7836
52	C	11.5041	13.7274	8.2031
53	H	12.3507	14.2677	7.7970
54	C	11.0694	12.4190	7.8533
55	C	11.6933	11.4503	6.9180
56	C	11.7538	11.7253	5.5236
57	C	11.1431	12.9920	4.9487
58	H	10.1234	13.1540	5.3325
59	H	11.1004	12.9387	3.8499
60	H	11.7309	13.8851	5.2200
61	C	12.3805	10.7975	4.6650
62	H	12.4132	11.0078	3.5935
63	C	12.9665	9.6123	5.1552
64	C	13.6754	8.6472	4.2217
65	H	14.7588	8.8606	4.1745
66	H	13.2808	8.7205	3.1957
67	H	13.5635	7.6050	4.5631
68	C	12.8927	9.3544	6.5393
69	H	13.3315	8.4370	6.9378
70	C	12.2564	10.2446	7.4283
71	C	12.1387	9.8803	8.8973
72	H	11.0987	9.6168	9.1508
73	H	12.4101	10.7270	9.5482
74	H	12.7848	9.0228	9.1394
75	C	8.9249	11.5631	12.6012
76	H	9.0405	12.4079	13.2710
77	C	8.9722	10.1849	12.8362
78	H	9.1377	9.6789	13.7796
79	C	8.7361	9.5879	11.5670
80	C	8.6674	8.1611	11.1682
81	C	9.8106	7.3244	11.3108
82	C	11.1057	7.8554	11.9027
83	H	11.0561	7.9043	13.0045
84	H	11.3206	8.8738	11.5467
85	H	11.9510	7.2016	11.6369
86	C	9.7400	5.9828	10.8827
87	H	10.6241	5.3490	10.9855
88	C	8.5617	5.4449	10.3243
89	C	8.5189	4.0053	9.8440
90	H	7.4916	3.7016	9.5886
91	H	8.9003	3.3123	10.6128
92	H	9.1462	3.8660	8.9451
93	C	7.4372	6.2850	10.2028
94	H	6.5128	5.8816	9.7846
95	C	7.4682	7.6355	10.6089
96	C	6.2250	8.4947	10.4545
97	H	6.3743	9.2768	9.6953
98	H	5.9804	9.0140	11.3961
99	H	5.3611	7.8796	10.1589
100	B	8.5642	13.0834	10.4578
101	H	8.6376	14.0306	11.2124

TOL_INTHOMO

Energy (POTENTIAL) = -2787.04601997 Eh

	Atom	X	Y	Z
1	Cu	0.3126	-0.1982	-0.9090
2	N	1.3739	-1.1181	-2.4136
3	C	2.6585	-1.4805	-2.5733
4	C	1.6015	-1.8919	-4.4823
5	C	2.8503	-1.9735	-3.8808
6	H	1.2699	-2.1643	-5.4743
7	H	3.7683	-2.3517	-4.3060
8	N	-0.5169	1.1047	-2.4356
9	C	-0.7391	2.4191	-2.6244
10	C	-1.3392	2.6241	-3.8877
11	C	-1.4508	1.3593	-4.4416
12	H	-1.6244	3.5669	-4.3299
13	H	-1.8336	1.0319	-5.3978
14	N	-1.4118	-1.4996	-1.3946
15	C	-2.3050	-2.1985	-0.6833
16	C	-3.0779	-3.0038	-1.5520
17	C	-2.5922	-2.7213	-2.8238
18	H	-3.8843	-3.6722	-1.2867
19	H	-2.8889	-3.0884	-3.7964
20	N	0.7323	-1.3791	-3.5856
21	N	-1.5939	-1.8157	-2.6990
22	N	-0.9566	0.4653	-3.5575
23	H	-1.1269	-1.3707	-4.8659
24	C	0.4886	-0.0613	0.9156
25	C	1.2806	0.8643	1.7312
26	O	2.4125	0.3837	1.9610
27	C	1.7870	3.0284	2.7182
28	H	1.3945	4.0263	2.5022
29	H	2.7394	2.9287	2.1880
30	B	-0.7712	-1.0604	-3.7614
31	C	2.0121	2.8428	4.2203
32	H	2.4585	1.8669	4.4271
33	H	1.0775	2.9169	4.7823

34	H	2.6978	3.6162	4.5865
35	C	-0.3412	3.4623	-1.6501
36	C	1.0094	3.6059	-1.2618
37	C	-1.3145	4.3706	-1.1623
38	C	1.3605	4.6565	-0.4008
39	C	-0.9161	5.4059	-0.3105
40	C	0.4194	5.5675	0.0836
41	H	2.4020	4.7607	-0.1052
42	H	-1.6686	6.0935	0.0710
43	C	-2.4098	-1.9628	0.7772
44	C	-1.9863	-2.9353	1.7046
45	C	-2.8920	-0.7122	1.2276
46	C	-2.0658	-2.6492	3.0743
47	C	-2.9538	-0.4673	2.6046
48	C	-2.5534	-1.4242	3.5459
49	H	-1.7303	-3.3932	3.7912
50	H	-3.3242	0.4940	2.9531
51	C	3.6270	-1.4197	-1.4541
52	C	3.4861	-2.3265	-0.3816
53	C	4.6953	-0.5003	-1.4728
54	C	4.4199	-2.2925	0.6583
55	C	5.6004	-0.4914	-0.4035
56	C	5.4812	-1.3807	0.6692
57	H	4.3179	-2.9907	1.4847
58	H	6.4167	0.2286	-0.4100
59	C	2.0947	2.6680	-1.7379
60	H	1.8954	2.2833	-2.7408
61	H	2.1805	1.7980	-1.0766
62	H	3.0661	3.1714	-1.7424
63	C	-2.7786	4.2302	-1.5176
64	H	-3.0980	3.1837	-1.5033
65	H	-2.9931	4.6141	-2.5214
66	H	-3.4023	4.7892	-0.8132
67	C	0.8108	6.6734	1.0348
68	H	0.3165	6.5512	2.0071
69	H	0.5148	7.6560	0.6484
70	H	1.8911	6.6923	1.2086
71	C	-3.3288	0.3540	0.2474
72	H	-4.0844	-0.0300	-0.4477
73	H	-2.4911	0.6981	-0.3675
74	H	-3.7555	1.2152	0.7700
75	C	-1.4244	-4.2596	1.2394
76	H	-0.6431	-4.1201	0.4841
77	H	-2.1975	-4.8861	0.7796
78	H	-0.9974	-4.8169	2.0776
79	C	-2.6695	-1.1581	5.0277
80	H	-1.9028	-1.6986	5.5900
81	H	-3.6494	-1.4843	5.4016
82	H	-2.5746	-0.0901	5.2506
83	C	4.8821	0.4639	-2.6227
84	H	3.9289	0.8617	-2.9799
85	H	5.3637	-0.0251	-3.4788
86	H	5.5155	1.3059	-2.3268
87	C	2.3332	-3.3026	-0.3350
88	H	2.1444	-3.7593	-1.3118
89	H	1.4068	-2.7926	-0.0453
90	H	2.5268	-4.1049	0.3819
91	C	6.4383	-1.3267	1.8361
92	H	6.0559	-0.6584	2.6187
93	H	7.4219	-0.9509	1.5352
94	H	6.5761	-2.3142	2.2898
95	H	0.0284	-0.8262	1.5569
96	N	0.8463	2.0698	2.1236
97	C	-0.5416	2.4631	1.8612
98	H	-0.5420	3.2992	1.1605
99	C	-1.3206	2.8275	3.1238
100	H	-2.3508	3.0833	2.8529
101	H	-1.3397	1.9968	3.8338
102	H	-0.8863	3.7006	3.6219
103	H	-1.0265	1.6224	1.3537
104	N	0.8340	-0.9478	4.3766
105	N	0.3800	0.0434	4.6987
106	C	1.3080	-2.1171	4.0426
107	C	0.8199	-3.2472	4.8542
108	O	0.1409	-3.0447	5.8766
109	N	1.1376	-4.5077	4.4318
110	C	1.8448	-4.8062	3.1847
111	H	1.4475	-5.7530	2.8030
112	H	1.5874	-4.0531	2.4380
113	C	0.7548	-5.6443	5.2784
114	H	0.7642	-5.3122	6.3184
115	H	1.5264	-6.4137	5.1643
116	C	-0.6218	-6.2101	4.9213
117	H	-0.8576	-7.0675	5.5626
118	H	-1.3961	-5.4511	5.0664
119	H	-0.6578	-6.5493	3.8799
120	C	3.3615	-4.9082	3.3666
121	H	3.6128	-5.6636	4.1191
122	H	3.8455	-5.1960	2.4269
123	H	3.7824	-3.9527	3.6964
124	H	2.0191	-2.1154	3.2291

TOL_TSHOMO

Energy (POTENTIAL) = -2787.03968098 Eh

	Atom	X	Y	Z
1	Cu	1.1949	0.5047	0.7384
2	N	2.1036	2.3749	0.8073
3	C	1.7516	3.6534	0.5594
4	C	3.8843	3.6562	1.1792
5	C	2.8633	4.4981	0.7709
6	H	4.9025	3.8645	1.4752
7	H	2.8936	5.5717	0.6616
8	N	3.0945	-0.5471	0.2939
9	C	3.4810	-1.6678	-0.3398
10	C	4.8745	-1.8471	-0.1880
11	C	5.2910	-0.7711	0.5820
12	H	5.4773	-2.6475	-0.5915
13	H	6.2674	-0.4969	0.9561
14	N	1.9265	0.3051	2.8438
15	C	1.4240	0.0010	4.0506
16	C	2.4033	0.2190	5.0476
17	C	3.5190	0.6700	4.3573
18	H	2.3012	0.0569	6.1107
19	H	4.5021	0.9588	4.7015
20	N	3.4115	2.3910	1.1938
21	N	3.2091	0.7131	3.0413
22	N	4.2079	-0.0086	0.8563
23	H	5.1809	1.4726	2.2412
24	C	-0.3182	-0.1646	-0.0830
25	C	-1.6442	-0.4058	0.5551
26	O	-2.2543	0.5618	1.0479
27	C	-3.4835	-1.8898	1.1062
28	H	-3.6879	-1.0622	1.7856
29	H	-3.4945	-2.8073	1.6981
30	B	4.0884	1.1607	1.8494
31	C	-4.5391	-1.9441	-0.0013
32	H	-4.3572	-2.7745	-0.6925
33	H	-4.5333	-1.0147	-0.5749
34	H	-5.5372	-2.0780	0.4319
35	C	2.5169	-2.5362	-1.0585
36	C	1.9014	-2.0759	-2.2441
37	C	2.2391	-3.8322	-0.5753
38	C	0.9891	-2.9043	-2.9055
39	C	1.3367	-4.6394	-1.2818
40	C	0.6920	-4.1909	-2.4390
41	H	0.4969	-2.5348	-3.8021
42	H	1.1205	-5.6374	-0.9058
43	C	0.0477	-0.5229	4.2143
44	C	-0.9113	0.2047	4.9533
45	C	-0.2883	-1.7792	3.6649
46	C	-2.1935	-0.3320	5.1169
47	C	-1.5742	-2.2895	3.8763
48	C	-2.5442	-1.5804	4.5912
49	H	-2.9363	0.2381	5.6721
50	H	-1.8253	-3.2648	3.4682
51	C	0.3705	4.0819	0.2310
52	C	-0.6878	3.8148	1.1324
53	C	0.1233	4.8436	-0.9372
54	C	-1.9717	4.2954	0.8326
55	C	-1.1757	5.2948	-1.1972
56	C	-2.2365	5.0422	-0.3193
57	H	-2.7810	4.0805	1.5265
58	H	-1.3611	5.8652	-2.1055
59	C	2.2537	-0.7245	-2.8121
60	H	3.2917	-0.7130	-3.1689
61	H	2.1767	0.0579	-2.0559
62	H	1.6030	-0.4646	-3.6507
63	C	2.8957	-4.3691	0.6772
64	H	2.9778	-3.6043	1.4534
65	H	3.9128	-4.7247	0.4721
66	H	2.3286	-5.2125	1.0835
67	C	-0.3416	-5.0422	-3.1365
68	H	-0.2353	-6.1011	-2.8792
69	H	-0.2726	-4.9478	-4.2256
70	H	-1.3566	-4.7334	-2.8514
71	C	0.7179	-2.5787	2.8701
72	H	1.6795	-2.6390	3.3907
73	H	0.9203	-2.1129	1.9008
74	H	0.3603	-3.5966	2.6930
75	C	-0.5803	1.5352	5.5914
76	H	0.1201	2.1171	4.9893
77	H	-0.1164	1.3956	6.5762
78	H	-1.4861	2.1317	5.7386
79	C	-3.9409	-2.1272	4.7712
80	H	-4.2980	-1.9848	5.7975
81	H	-3.9877	-3.1973	4.5448
82	H	-4.6525	-1.6172	4.1090
83	C	1.2243	5.1986	-1.9118
84	H	1.9937	4.4258	-1.9725
85	H	1.7266	6.1270	-1.6125
86	H	0.8169	5.3565	-2.9153
87	C	-0.4875	3.0377	2.4094
88	H	0.5174	3.1685	2.8158
89	H	-0.6418	1.9686	2.2350
90	H	-1.2156	3.3504	3.1635
91	C	-3.6147	5.5946	-0.5974
92	H	-4.3869	5.0505	-0.0455
93	H	-3.8598	5.5505	-1.6644
94	H	-3.6791	6.6483	-0.2965
95	H	-0.2768	-0.5762	-1.0975
96	N	-2.1339	-1.6789	0.5774
97	C	-1.4658	-2.7566	-0.1779
98	H	-1.7333	-2.6845	-1.2402
99	C	-1.7836	-4.1575	0.3390
100	H	-1.1782	-4.8773	-0.2167
101	H	-1.5361	-4.2597	1.3993
102	H	-2.8344	-4.4284	0.1990
103	H	-0.3859	-2.6060	-0.1089

104	N	-0.1217	1.9422	-2.1503
105	N	0.9086	2.1193	-2.5878
106	C	-1.3080	1.6806	-1.6551
107	C	-2.1759	0.8215	-2.4991
108	O	-1.6699	-0.0336	-3.2446
109	N	-3.5248	0.9925	-2.3879
110	C	-4.1732	2.1705	-1.7917
111	H	-3.4242	2.9485	-1.6451
112	H	-4.8831	2.5627	-2.5311
113	C	-4.3834	0.0893	-3.1683
114	H	-5.3623	0.0691	-2.6797
115	H	-3.9607	-0.9160	-3.1094
116	C	-4.5231	0.5165	-4.6321
117	H	-5.1998	-0.1645	-5.1612
118	H	-4.9327	1.5296	-4.7155
119	H	-3.5492	0.4906	-5.1284
120	C	-4.8908	1.8740	-0.4748
121	H	-4.1871	1.4938	0.2693
122	H	-5.3539	2.7905	-0.0923
123	H	-5.6893	1.1364	-0.6112
124	H	-1.5974	2.2629	-0.7921

TOL_olefin5
Energy (POTENTIAL) = -730.590397992 Eh

	Atom	X	Y	Z
1	C	0.6614	-0.2890	2.8136
2	C	1.4614	0.9617	2.6251
3	O	2.6327	1.0270	3.0311
4	C	1.6360	3.2218	1.7195
5	C	1.3192	4.3696	2.6826
6	H	1.4972	4.0657	3.7193
7	H	0.2797	4.7020	2.5923
8	H	1.9643	5.2280	2.4622
9	H	-0.3122	-0.4045	2.3524
10	N	0.8540	2.0047	1.9776
11	C	-0.5701	2.0280	1.5995
12	H	-0.6872	2.8575	0.8966
13	C	-1.5329	2.2132	2.7772
14	H	-2.5643	2.2653	2.4094
15	H	-1.4710	1.3796	3.4837
16	H	-1.3156	3.1350	3.3248
17	H	-0.8213	1.1212	1.0402
18	C	1.1359	-1.2947	3.5608
19	C	0.3279	-2.5375	3.7655
20	O	-0.8679	-2.5816	3.4340
21	N	0.9516	-3.6020	4.3526
22	C	2.3890	-3.6747	4.6536
23	H	2.9524	-3.1021	3.9139
24	C	0.1554	-4.7980	4.6565
25	H	-0.8597	-4.4772	4.8992
26	H	0.5872	-5.2597	5.5509
27	C	0.1286	-5.7955	3.4957
28	H	-0.4486	-6.6856	3.7714
29	H	-0.3372	-5.3421	2.6157
30	H	1.1403	-6.1189	3.2248
31	C	2.7250	-3.2123	6.0730
32	H	2.1728	-3.7964	6.8173
33	H	3.7962	-3.3334	6.2707
34	H	2.4673	-2.1573	6.2134
35	H	2.1046	-1.1774	4.0328
36	H	2.6900	2.9530	1.7958
37	H	1.4387	3.5304	0.6856
38	H	2.6909	-4.7184	4.5165

TOL_azetidinone4
Energy (POTENTIAL) = -365.284841007 Eh

	Atom	X	Y	Z
1	C	-3.8043	0.2331	0.7012
2	C	-2.5878	-0.6984	0.9950
3	H	-2.4606	-1.5257	0.2908
4	H	-2.5022	-1.0646	2.0212
5	N	-2.8158	1.2978	0.4173
6	C	-1.7179	0.5316	0.6846
7	C	-4.7024	-0.1340	-0.4702
8	H	-5.3131	-1.0094	-0.2212
9	H	-5.3834	0.6873	-0.7219
10	H	-4.1049	-0.3689	-1.3585
11	H	-4.3996	0.4584	1.5951
12	C	-2.9701	2.6938	0.0609
13	H	-1.9726	3.0619	-0.1982
14	H	-3.5912	2.7631	-0.8417
15	C	-3.5781	3.5281	1.1922
16	H	-2.9482	3.4851	2.0870
17	H	-3.6703	4.5757	0.8846
18	H	-4.5780	3.1674	1.4589
19	O	-0.5213	0.7825	0.6715

TOL_pyrrolidinone3
Energy (POTENTIAL) = -365.308626735 Eh

	Atom	X	Y	Z
1	C	-4.4004	0.6975	0.6850
2	C	-2.6063	-0.7869	0.0321
3	H	-2.2175	-1.3645	-0.8099
4	H	-2.1763	-1.2087	0.9497
5	N	-3.2222	1.4566	0.2685
6	C	-2.1572	0.6689	-0.0628
7	C	-4.1402	-0.7039	0.0960
8	H	-4.5968	-1.4952	0.6958
9	H	-4.5592	-0.7592	-0.9143
10	H	-5.3117	1.1647	0.2955
11	C	-3.1367	2.9029	0.4048
12	H	-2.2514	3.2204	-0.1523
13	H	-4.0178	3.3453	-0.0775
14	C	-3.0422	3.3645	1.8629
15	H	-2.1551	2.9411	2.3460
16	H	-2.9678	4.4568	1.9080
17	H	-3.9253	3.0655	2.4386
18	O	-1.0320	1.0703	-0.3642
19	H	-4.4762	0.6698	1.7817

TOL_TS5
Energy (POTENTIAL) = -2312.25881089 Eh

	Atom	X	Y	Z
1	Cu	4.1151	3.4427	2.7286
2	N	4.7112	5.4180	2.0662
3	C	4.1282	6.6121	1.8810
4	C	6.2164	6.7733	1.1488
5	C	5.0473	7.5091	1.2912
6	H	7.1862	7.0477	0.7580
7	H	4.8808	8.5439	1.0298
8	N	5.1974	2.6816	1.0407
9	C	5.0163	1.7429	0.1003
10	C	6.1926	1.6047	-0.6724
11	C	7.0784	2.5297	-0.1394
12	H	6.3525	0.9389	-1.5079
13	H	8.0910	2.7874	-0.4167
14	N	5.9504	3.4365	3.8575
15	C	6.2947	3.1945	5.1312
16	C	7.6561	3.5184	5.3274
17	C	8.0968	3.9655	4.0882
18	H	8.2283	3.4249	6.2390
19	H	9.0664	4.3138	3.7607
20	N	5.9901	5.5257	1.6188
21	N	7.0565	3.9062	3.2250
22	N	6.4611	3.1629	0.8839
23	H	8.0670	4.6754	1.3590
24	C	2.4553	2.8565	3.5861
25	C	1.7939	1.5345	3.3348
26	O	2.3313	0.5030	3.7503
27	C	-0.0994	0.3097	2.3394
28	B	6.9796	4.3367	1.7440
29	C	3.7110	1.0710	-0.1072
30	C	2.6092	1.8214	-0.5704
31	C	3.5874	-0.3228	0.0872
32	C	1.4145	1.1550	-0.8764
33	C	2.3760	-0.9471	-0.2280
34	C	1.2843	-0.2296	-0.7317
35	H	0.5705	1.7322	-1.2495
36	H	2.2790	-2.0199	-0.0726
37	C	5.2847	2.6502	6.0691
38	C	4.7656	1.3557	5.8471
39	C	4.8040	3.4385	7.1370
40	C	3.7525	0.8812	6.6894
41	C	3.7898	2.9246	7.9550
42	C	3.2492	1.6491	7.7452
43	H	3.3347	-0.1036	6.4984
44	H	3.4087	3.5372	8.7703
45	C	2.7415	6.8724	2.3390
46	C	2.4543	6.8702	3.7254
47	C	1.7219	7.1582	1.4071
48	C	1.1575	7.1818	4.1499
49	C	0.4323	7.4538	1.8759
50	C	0.1332	7.4854	3.2426
51	H	0.9396	7.1861	5.2162
52	H	-0.3535	7.6680	1.1543
53	C	2.6995	3.3212	-0.7423
54	H	3.5602	3.6044	-1.3588
55	H	2.8394	3.8221	0.2214
56	H	1.7957	3.7180	-1.2147
57	C	4.7290	-1.1297	0.6615
58	H	5.0769	-0.6964	1.6058
59	H	5.5909	-1.1574	-0.0140
60	H	4.4197	-2.1611	0.8558
61	C	0.0043	-0.9429	-1.0996
62	H	0.1407	-1.5582	-1.9980
63	H	-0.8079	-0.2380	-1.3050
64	H	-0.3242	-1.6156	-0.2986
65	C	5.3622	4.8178	7.4090
66	H	5.4976	5.3926	6.4886
67	H	6.3456	4.7596	7.8917
68	H	4.7026	5.3837	8.0745
69	C	5.2784	0.4952	4.7161
70	H	6.3740	0.4652	4.7071
71	H	4.9532	0.8890	3.7494
72	H	4.8980	-0.5256	4.8004
73	C	2.1708	1.1057	8.6540
74	H	2.6070	0.6058	9.5291
75	H	1.5457	0.3697	8.1375
76	H	1.5191	1.9031	9.0282
77	C	1.9971	7.1525	-0.0797
78	H	2.6389	6.3150	-0.3690
79	H	2.5128	8.0685	-0.3918
80	H	1.0654	7.0875	-0.6504
81	C	3.5181	6.5247	4.7414
82	H	4.4334	7.1031	4.5766
83	H	3.8040	5.4707	4.6667
84	H	3.1667	6.7148	5.7594
85	C	-1.2485	7.8467	3.7349

86	H	-1.9914	7.7850	2.9332
87	H	-1.2689	8.8725	4.1247
88	H	-1.5703	7.1875	4.5492
89	H	2.4594	3.0773	4.6563
90	N	0.6027	1.5468	2.6680
91	C	0.0897	2.8283	2.2183
92	C	0.6439	3.8880	3.1399
93	H	1.9142	3.8450	3.0260
94	H	0.2650	3.8702	4.1580
95	H	0.6417	4.8985	2.7270
96	H	-1.0059	2.8308	2.2613
97	C	-1.2040	-0.0137	3.3486
98	H	-1.9478	0.7905	3.3991
99	H	-1.7252	-0.9345	3.0619
100	H	-0.7814	-0.1552	4.3486
101	H	0.6437	-0.4896	2.3089
102	H	0.3871	3.0237	1.1783
103	H	-0.5131	0.4143	1.3323

TOL_TS4

Energy (POTENTIAL) = -2312.25880215 Eh

	Atom	X	Y	Z
1	Cu	4.1238	3.4383	2.7374
2	N	4.7252	5.4060	2.0778
3	C	4.1423	6.5984	1.8824
4	C	6.2331	6.7562	1.1573
5	C	5.0630	7.4921	1.2906
6	H	7.2039	7.0283	0.7675
7	H	4.8961	8.5250	1.0214
8	N	5.2014	2.6752	1.0409
9	C	5.0115	1.7559	0.0825
10	C	6.1839	1.6281	-0.6982
11	C	7.0764	2.5380	-0.1513
12	H	6.3377	0.9781	-1.5472
13	H	8.0887	2.7967	-0.4285
14	N	5.9586	3.4210	3.8670
15	C	6.2977	3.1848	5.1431
16	C	7.6595	3.5051	5.3422
17	C	8.1056	3.9446	4.1019
18	H	8.2288	3.4142	6.2559
19	H	9.0772	4.2888	3.7762
20	N	6.0060	5.5117	1.6348
21	N	7.0678	3.8839	3.2356
22	N	6.4671	3.1530	0.8880
23	H	8.0800	4.6554	1.3715
24	C	2.4833	2.8233	3.6113
25	C	1.8192	1.5111	3.3209
26	O	2.3719	0.4638	3.6723
27	C	-0.0936	0.3194	2.3283
28	B	6.9917	4.3188	1.7559
29	C	3.7048	1.0876	-0.1281
30	C	2.5879	1.8489	-0.5347
31	C	3.5927	-0.3142	0.0101
32	C	1.3879	1.1883	-0.8336
33	C	2.3763	-0.9323	-0.2972
34	C	1.2666	-0.2011	-0.7381
35	H	0.5321	1.7755	-1.1612
36	H	2.2897	-2.0114	-0.1846
37	C	5.2773	2.6504	6.0756
38	C	4.7587	1.3555	5.8572
39	C	4.7791	3.4512	7.1262
40	C	3.7242	0.8944	6.6815
41	C	3.7445	2.9508	7.9266
42	C	3.2008	1.6761	7.7171
43	H	3.3071	-0.0910	6.4918
44	H	3.3498	3.5730	8.7280
45	C	2.7533	6.8619	2.3311
46	C	2.4622	6.8869	3.7148
47	C	1.7344	7.1231	1.3893
48	C	1.1638	7.2100	4.1303
49	C	0.4440	7.4238	1.8480
50	C	0.1423	7.4914	3.2143
51	H	0.9442	7.2362	5.1956
52	H	-0.3420	7.6165	1.1202
53	C	2.6657	3.3539	-0.6590
54	H	3.5211	3.6628	-1.2702
55	H	2.8065	3.8259	0.3188
56	H	1.7563	3.7587	-1.1136
57	C	4.7552	-1.1410	0.5095
58	H	5.1676	-0.7216	1.4337
59	H	5.5732	-1.1750	-0.2184
60	H	4.4433	-2.1698	0.7132
61	C	-0.0236	-0.9063	-1.0850
62	H	0.1059	-1.5613	-1.9556
63	H	-0.8222	-0.1959	-1.3220
64	H	-0.3678	-1.5389	-0.2579
65	C	5.3415	4.8287	7.3980
66	H	5.4936	5.3975	6.4765
67	H	6.3176	4.7665	7.8946
68	H	4.6765	5.4024	8.0514
69	C	5.2904	0.4824	4.7452
70	H	6.3861	0.4742	4.7370
71	H	4.9555	0.8495	3.7713
72	H	4.9305	-0.5440	4.8504
73	C	2.0995	1.1499	8.6084
74	H	2.5111	0.7108	9.5268
75	H	1.5166	0.3702	8.1070
76	H	1.4113	1.9470	8.9120
77	C	2.0162	7.0904	-0.0960
78	H	2.6923	6.2734	-0.3628
79	H	2.4959	8.0193	-0.4276
80	H	1.0903	6.9754	-0.6683
81	C	3.5217	6.5539	4.7392
82	H	4.4391	7.1279	4.5699
83	H	3.8050	5.4980	4.6786
84	H	3.1675	6.7581	5.7534
85	C	-1.2425	7.8727	3.6824
86	H	-2.0155	7.4772	3.0143
87	H	-1.3619	8.9636	3.7048
88	H	-1.4423	7.5017	4.6929
89	H	2.5190	2.9978	4.6894
90	N	0.6094	1.5468	2.6896
91	C	0.0689	2.8439	2.3255
92	C	0.6557	3.8644	3.2716
93	H	1.9220	3.8314	3.1101
94	H	0.3150	3.8026	4.3016
95	H	0.6345	4.8914	2.9028
96	H	-1.0238	2.8355	2.4160
97	C	-1.1226	-0.0912	3.3841
98	H	-1.8717	0.6943	3.5407
99	H	-1.6492	-1.0001	3.0705
100	H	-0.6305	-0.2924	4.3410
101	H	0.6575	-0.4618	2.1970
102	H	0.3185	3.0943	1.2844
103	H	-0.5737	0.4803	1.3585

TOL_metallocarbene6

Energy (POTENTIAL) = -2312.27930916 Eh

	Atom	X	Y	Z
1	Cu	0.6223	-0.3747	-0.3129
2	N	1.3909	0.0494	-2.1699
3	C	1.5342	1.1787	-2.8824
4	C	2.3866	-0.4893	-4.0760
5	C	2.1622	0.8815	-4.1107
6	H	2.8485	-1.1522	-4.7939
7	H	2.4216	1.5743	-4.8975
8	N	-0.3353	-2.1454	-1.3080
9	C	-1.5235	-2.7659	-1.3176
10	C	-1.4473	-3.9390	-2.1050
11	C	-0.1417	-3.9687	-2.5725
12	H	-2.2417	-4.6414	-2.3117
13	H	0.3687	-4.6708	-3.2169
14	N	2.2878	-1.6418	0.0156
15	C	3.1635	-1.7755	1.0243
16	C	4.2030	-2.6470	0.6318
17	C	3.8864	-3.0198	-0.6690
18	H	5.0508	-2.9617	1.2225
19	H	4.3911	-3.6755	-1.3645
20	N	1.9158	-0.9677	-2.9040
21	N	2.7341	-2.4035	-1.0150
22	N	0.5040	-2.8847	-2.0837
23	H	2.4883	-3.1455	-3.1366
24	C	-0.0511	0.6683	1.0316
25	C	-1.3450	0.6584	1.7242
26	O	-1.3375	-0.1844	2.6449
27	C	-3.6235	1.3319	2.1805
28	H	-3.8010	0.2700	2.3635
29	H	-4.4369	1.6942	1.5456
30	B	1.9507	-2.4180	-2.3464
31	C	-3.5794	2.0910	3.5091
32	H	-3.4183	3.1630	3.3603
33	H	-2.7739	1.7043	4.1404
34	H	-4.5261	1.9583	4.0461
35	C	-2.7259	-2.1777	-0.6772
36	C	-3.2861	-1.0004	-1.2176
37	C	-3.3458	-2.8200	0.4169
38	C	-4.4831	-0.5090	-0.6788
39	C	-4.5338	-2.2893	0.9314
40	C	-5.1279	-1.1439	0.3867
41	H	-4.9226	0.3901	-1.1063
42	H	-5.0055	-2.7794	1.7811
43	C	2.9350	-1.0606	2.3017
44	C	3.7851	-0.0005	2.6842
45	C	1.8269	-1.4134	3.1028
46	C	3.5027	0.7016	3.8627
47	C	1.5824	-0.6849	4.2732
48	C	2.4051	0.3749	4.6698
49	H	4.1502	1.5272	4.1527
50	H	0.7150	-0.9403	4.8762
51	C	1.0899	2.4849	-2.3401
52	C	1.7557	3.0448	-1.2242
53	C	0.0109	3.1674	-2.9387
54	C	1.3216	4.2793	-0.7283
55	C	-0.3852	4.4057	-2.4135
56	C	0.2591	4.9804	-1.3138
57	H	1.8346	4.7128	0.1283
58	H	-1.2193	4.9300	-2.8756
59	C	-2.6125	-0.2653	-2.3539
60	H	-2.4049	-0.9318	-3.1987
61	H	-1.6473	0.1420	-2.0377
62	H	-3.2348	0.5594	-2.7140
63	C	-2.7293	-4.0487	1.0454
64	H	-1.6893	-3.8616	1.3349
65	H	-2.7194	-4.8977	0.3532
66	H	-3.2812	-4.3497	1.9409
67	C	-6.4169	-0.6037	0.9601
68	H	-7.2337	-1.3281	0.8535
69	H	-6.7249	0.3198	0.4596

70	H	-6.3180	-0.3896	2.0314
71	C	0.8994	-2.5363	2.7024
72	H	1.4574	-3.4214	2.3775
73	H	0.2600	-2.2295	1.8697
74	H	0.2475	-2.8189	3.5333
75	C	4.9773	0.3930	1.8401
76	H	4.7247	0.4381	0.7765
77	H	5.7952	-0.3308	1.9400
78	H	5.3633	1.3715	2.1420
79	C	2.1303	1.1305	5.9491
80	H	2.4714	2.1695	5.8841
81	H	2.6507	0.6695	6.7990
82	H	1.0612	1.1372	6.1866
83	C	-0.7210	2.5868	-4.1277
84	H	-0.9395	1.5239	-3.9885
85	H	-0.1244	2.6697	-5.0439
86	H	-1.6660	3.1112	-4.2995
87	C	2.9284	2.3434	-0.5773
88	H	3.6621	2.0221	-1.3245
89	H	2.6119	1.4420	-0.0451
90	H	3.4314	2.9997	0.1386
91	C	-0.1658	6.3223	-0.7662
92	H	-1.0619	6.6983	-1.2694
93	H	0.6271	7.0694	-0.8969
94	H	-0.3801	6.2666	0.3074
95	H	0.6361	1.3144	1.5985
96	N	-2.3825	1.4487	1.4037
97	C	-2.3068	2.3715	0.2694
98	H	-1.3355	2.2259	-0.2165
99	C	-2.4789	3.8370	0.6667
100	H	-2.3935	4.4693	-0.2209
101	H	-3.4602	4.0193	1.1166
102	H	-1.7080	4.1407	1.3820
103	H	-3.0719	2.0803	-0.4611

TOL_metallocarbene7

Energy (POTENTIAL) = -2198.97560717 Eh

	Atom	X	Y	Z
1	Cu	8.5625	10.4570	8.5268
2	N	8.2959	8.2335	6.5754
3	N	7.2184	13.0489	9.7001
4	N	6.9661	12.0983	8.7692
5	N	9.7319	13.0630	9.4592
6	N	9.9570	12.0541	8.5715
7	N	8.6488	11.6902	11.2735
8	N	8.6467	10.4891	10.6369
9	C	7.8113	9.1560	7.3730
10	H	6.7135	9.1235	7.3262
11	C	9.7488	8.0476	6.4269
12	H	9.9875	8.0494	5.3571
13	H	10.2125	8.9240	6.8743
14	C	10.2501	6.7703	7.0965
15	H	9.8128	5.8720	6.6464
16	H	10.0110	6.7773	8.1639
17	H	11.3375	6.7046	6.9852
18	C	7.4217	7.3283	5.7849
19	H	6.7028	7.9537	5.2474
20	H	8.0460	6.8232	5.0429
21	C	6.6913	6.3080	6.6561
22	H	6.0667	5.6621	6.0289
23	H	6.0421	6.8129	7.3774
24	H	7.3954	5.6813	7.2106
25	C	6.1687	13.8994	9.8008
26	H	6.1916	14.7293	10.4935
27	C	5.1925	13.4844	8.9023
28	H	4.2299	13.9390	8.7167
29	C	5.7468	12.3444	8.2732
30	C	5.2108	11.4533	7.2141
31	C	4.1510	10.5652	7.5023
32	C	3.4944	10.5559	8.8645
33	H	4.2399	10.5341	9.6658
34	H	2.8414	9.6852	8.9796
35	H	2.8862	11.4538	9.0237
36	C	3.7341	9.6607	6.5185
37	H	2.9268	8.9667	6.7462
38	C	4.3377	9.6154	5.2547
39	C	3.8765	8.6144	4.2209
40	H	3.9388	7.5884	4.6044
41	H	4.4790	8.6701	3.3088
42	H	2.8306	8.7863	3.9384
43	C	5.3833	10.5058	4.9899
44	H	5.8711	10.4797	4.0175
45	C	5.8361	11.4227	5.9496
46	C	7.0174	12.3124	5.6404
47	H	6.8318	13.3504	5.9362
48	H	7.2549	12.2928	4.5723
49	H	7.8990	11.9783	6.1983
50	C	10.6910	14.0087	9.3403
51	H	10.6795	14.8860	9.9717
52	C	11.5743	13.6103	8.3466
53	H	12.4618	14.1219	8.0038
54	C	11.0740	12.3680	7.8971
55	C	11.6775	11.4379	6.9104
56	C	11.6530	11.7392	5.5315
57	C	10.9627	12.9848	5.0241
58	H	9.9533	13.0794	5.4375
59	H	10.8863	12.9747	3.9325
60	H	11.5085	13.8907	5.3127
61	C	12.2725	10.8620	4.6332
62	H	12.2460	11.0899	3.5690
63	C	12.9284	9.7022	5.0672
64	C	13.6226	8.7954	4.0779
65	H	14.5490	9.2529	3.7074
66	H	12.9908	8.5960	3.2045
67	H	13.8881	7.8345	4.5303
68	C	12.9311	9.4185	6.4366
69	H	13.4201	8.5138	6.7921
70	C	12.3064	10.2601	7.3672
71	C	12.2643	9.8720	8.8266
72	H	11.2405	9.6214	9.1264
73	H	12.5849	10.6940	9.4757
74	H	12.9018	9.0053	9.0241
75	C	8.6738	11.5026	12.6128
76	H	8.6752	12.3469	13.2881
77	C	8.6903	10.1353	12.8609
78	H	8.7095	9.6365	13.8191
79	C	8.6678	9.5375	11.5817
80	C	8.6422	8.1066	11.1904
81	C	9.8014	7.3127	11.3213
82	C	11.0795	7.8902	11.8860
83	H	11.0218	7.9957	12.9763
84	H	11.2837	8.8864	11.4824
85	H	11.9351	7.2461	11.6602
86	C	9.7578	5.9752	10.9091
87	H	10.6554	5.3655	10.9977
88	C	8.5950	5.4062	10.3742
89	C	8.5899	3.9713	9.9013
90	H	7.5802	3.6360	9.6443
91	H	8.9850	3.2943	10.6677
92	H	9.2192	3.8485	9.0102
93	C	7.4570	6.2114	10.2591
94	H	6.5454	5.7872	9.8444
95	C	7.4589	7.5553	10.6551
96	C	6.2099	8.3938	10.5081
97	H	6.3876	9.2518	9.8538
98	H	5.8882	8.7991	11.4749
99	H	5.3848	7.8063	10.0948
100	B	8.5581	13.0134	10.4687
101	H	8.6274	13.9505	11.2191

TOL_TSDCO

Energy (POTENTIAL) = -2312.25817236 Eh

	Atom	X	Y	Z
1	Cu	0.4846	-0.4396	-0.3499
2	N	1.2797	0.0309	-2.2268
3	C	1.3848	1.1590	-2.9454
4	C	2.4309	-0.4507	-4.0614
5	C	2.1069	0.8993	-4.1302
6	H	2.9820	-1.0848	-4.7415
7	H	2.3601	1.6012	-4.9114
8	N	-0.3350	-2.2668	-1.3923
9	C	-1.4736	-2.9740	-1.4530
10	C	-1.2823	-4.1287	-2.2479
11	C	0.0384	-4.0585	-2.6644
12	H	-2.0139	-4.8853	-2.4910
13	H	0.6229	-4.7149	-3.2937
14	N	2.1761	-1.5855	0.0412
15	C	2.9752	-1.6918	1.1135
16	C	4.0696	-2.5267	0.7997
17	C	3.8627	-2.9065	-0.5221
18	H	4.8833	-2.8131	1.4497
19	H	4.4397	-3.5439	-1.1773
20	N	1.9259	-0.9498	-2.9112
21	N	2.7210	-2.3262	-0.9547
22	N	0.5852	-2.9367	-2.1411
23	H	2.6359	-3.0922	-3.0816
24	C	-0.0539	0.5826	1.0959
25	C	-1.2932	0.6356	1.8167
26	O	-1.9856	-0.1881	2.3861
27	C	-2.9317	2.0571	0.6271
28	H	-3.0370	1.1267	0.0666
29	H	-2.7823	2.8628	-0.1013
30	B	2.0147	-2.3871	-2.3331
31	C	-4.1893	2.2988	1.4677
32	H	-4.1129	3.2225	2.0524
33	H	-4.3547	1.4643	2.1540
34	H	-5.0628	2.3910	0.8117
35	C	-2.7260	-2.4997	-0.8171
36	C	-3.3327	-1.3103	-1.2735
37	C	-3.3344	-3.2540	0.2114
38	C	-4.5484	-0.9056	-0.7040
39	C	-4.5418	-2.8075	0.7591
40	C	-5.1666	-1.6357	0.3149
41	H	-5.0193	0.0072	-1.0632
42	H	-4.9989	-3.3791	1.5648
43	C	2.6258	-0.9620	2.3558
44	C	3.3894	0.1568	2.7532
45	C	1.4807	-1.3429	3.0919
46	C	2.9962	0.8773	3.8879
47	C	1.1234	-0.5922	4.2205
48	C	1.8696	0.5155	4.6382
49	H	3.5795	1.7453	4.1896
50	H	0.2343	-0.8768	4.7778
51	C	0.8100	2.4258	-2.4292
52	C	1.4216	3.0669	-1.3310
53	C	-0.3574	2.9671	-3.0088
54	C	0.8540	4.2472	-0.8339
55	C	-0.9012	4.1398	-2.4703

56	C	-0.3091	4.7963	-1.3839
57	H	1.3308	4.7461	0.0074
58	H	-1.8102	4.5493	-2.9077
59	C	-2.6840	-0.4534	-2.3358
60	H	-2.3186	-1.0527	-3.1759
61	H	-1.8141	0.0755	-1.9314
62	H	-3.3858	0.2920	-2.7224
63	C	-2.6894	-4.5145	0.7428
64	H	-1.6308	-4.3536	0.9735
65	H	-2.7348	-5.3325	0.0149
66	H	-3.1898	-4.8514	1.6557
67	C	-6.4518	-1.1619	0.9515
68	H	-6.8681	-0.2977	0.4241
69	H	-6.2852	-0.8673	1.9952
70	H	-7.2126	-1.9515	0.9569
71	C	0.6387	-2.5265	2.6726
72	H	1.2622	-3.3982	2.4455
73	H	0.0641	-2.2989	1.7698
74	H	-0.0710	-2.7987	3.4584
75	C	4.6049	0.5926	1.9662
76	H	4.4075	0.6020	0.8901
77	H	5.4494	-0.0883	2.1261
78	H	4.9264	1.5953	2.2647
79	C	1.4821	1.2901	5.8766
80	H	1.7542	2.3478	5.7924
81	H	1.9942	0.8929	6.7629
82	H	0.4060	1.2300	6.0684
83	C	-1.0209	2.2956	-4.1895
84	H	-1.0931	1.2133	-4.0498
85	H	-0.4486	2.4579	-5.1109
86	H	-2.0289	2.6899	-4.3510
87	C	2.6605	2.4924	-0.6835
88	H	3.4627	2.3402	-1.4145
89	H	2.4547	1.5138	-0.2400
90	H	3.0374	3.1521	0.1038
91	C	-0.9182	6.0605	-0.8234
92	H	-1.9813	5.9225	-0.5919
93	H	-0.8526	6.8864	-1.5426
94	H	-0.4117	6.3782	0.0935
95	H	0.6931	1.2714	1.5087
96	N	-1.7052	1.9457	1.4241
97	C	-1.1307	3.1644	2.0314
98	H	-0.2335	3.4688	1.4842
99	C	-0.8304	3.0026	3.5203
100	H	-0.4292	3.9400	3.9216
101	H	-1.7341	2.7414	4.0806
102	H	-0.0851	2.2212	3.6915
103	H	-1.8686	3.9582	1.8828

TOL_TSDCO2

Energy (POTENTIAL) = -2312.27188179 Eh

	Atom	X	Y	Z
1	Cu	0.2451	-0.5116	-0.2796
2	N	1.1031	0.0947	-2.1803
3	C	1.2080	1.2168	-2.9082
4	C	2.1813	-0.4120	-4.0581
5	C	1.8851	0.9435	-4.1182
6	H	2.6979	-1.0578	-4.7544
7	H	2.1281	1.6389	-4.9085
8	N	-0.4872	-2.2168	-1.2712
9	C	-1.6322	-2.9158	-1.2840
10	C	-1.4764	-4.0784	-2.0702
11	C	-0.1691	-4.0187	-2.5349
12	H	-2.2199	-4.8341	-2.2781
13	H	0.3879	-4.6851	-3.1785
14	N	2.1367	-1.5785	0.0585
15	C	3.0439	-1.6622	1.0417
16	C	4.1406	-2.4466	0.6165
17	C	3.8233	-2.8219	-0.6832
18	H	5.0251	-2.7035	1.1815
19	H	4.3613	-3.4256	-1.4008
20	N	1.7025	-0.8989	-2.8899
21	N	2.6185	-2.2875	-0.9926
22	N	0.4035	-2.8953	-2.0464
23	H	2.3902	-3.0400	-3.1086
24	C	-0.1139	0.6490	1.3065
25	C	-1.1717	0.4068	2.1647
26	O	-1.8859	-0.1084	2.9777
27	C	-2.1075	1.8405	0.1338
28	H	-2.1886	0.8314	-0.2673
29	H	-1.5537	2.4491	-0.5862
30	B	1.8214	-2.3369	-2.3164
31	C	-3.4942	2.4132	0.4034
32	H	-3.4633	3.4593	0.7230
33	H	-4.0250	1.8267	1.1595
34	H	-4.0735	2.3732	-0.5241
35	C	-2.8475	-2.3826	-0.6229
36	C	-3.5142	-1.2853	-1.2150
37	C	-3.3374	-2.9546	0.5661
38	C	-4.6676	-0.7852	-0.6039
39	C	-4.4952	-2.4194	1.1496
40	C	-5.1737	-1.3376	0.5809
41	H	-5.1840	0.0578	-1.0586
42	H	-4.8644	-2.8507	2.0775
43	C	2.7880	-0.9672	2.3262
44	C	3.5660	0.1557	2.6883
45	C	1.7136	-1.3787	3.1436
46	C	3.2514	0.8497	3.8625
47	C	1.4362	-0.6579	4.3135
48	C	2.1911	0.4572	4.6900
49	H	3.8408	1.7244	4.1326
50	H	0.5974	-0.9678	4.9320
51	C	0.6769	2.4994	-2.3848
52	C	1.2758	3.0897	-1.2509
53	C	-0.4343	3.1170	-3.0018
54	C	0.7445	4.2861	-0.7486
55	C	-0.9329	4.3113	-2.4675
56	C	-0.3590	4.9114	-1.3389
57	H	1.2086	4.7411	0.1241
58	H	-1.7983	4.7780	-2.9344
59	C	-2.9866	-0.6570	-2.4857
60	H	-2.9508	-1.3861	-3.3038
61	H	-1.9625	-0.2934	-2.3521
62	H	-3.6159	0.1798	-2.8033
63	C	-2.6220	-4.1149	1.2190
64	H	-1.5544	-3.9046	1.3432
65	H	-2.6976	-5.0259	0.6138
66	H	-3.0433	-4.3308	2.2054
67	C	-6.4136	-0.7599	1.2222
68	H	-6.2630	0.2912	1.4989
69	H	-6.6900	-1.3056	2.1296
70	H	-7.2688	-0.7921	0.5361
71	C	0.8344	-2.5445	2.7534
72	H	1.4278	-3.4134	2.4490
73	H	0.2016	-2.2838	1.8981
74	H	0.1821	-2.8379	3.5807
75	C	4.7070	0.6284	1.8154
76	H	4.4123	0.6846	0.7629
77	H	5.5611	-0.0568	1.8674
78	H	5.0556	1.6175	2.1284
79	C	1.8774	1.2268	5.9518
80	H	2.6021	0.9992	6.7443
81	H	0.8826	0.9807	6.3362
82	H	1.9176	2.3086	5.7799
83	C	-1.1048	2.4892	-4.2027
84	H	-0.4589	2.5191	-5.0872
85	H	-2.0344	3.0108	-4.4505
86	H	-1.3407	1.4353	-4.0208
87	C	2.4737	2.4556	-0.5816
88	H	3.2954	2.3158	-1.2934
89	H	2.2307	1.4640	-0.1912
90	H	2.8403	3.0754	0.2421
91	C	-0.9381	6.1851	-0.7684
92	H	-0.9387	6.9910	-1.5120
93	H	-0.3694	6.5326	0.0998
94	H	-1.9791	6.0415	-0.4524
95	H	0.8452	0.9803	1.6980
96	N	-1.2602	1.7327	1.3507
97	C	-1.1128	3.0077	2.1156
98	H	-0.9682	3.7978	1.3724
99	C	0.0263	3.0267	3.1267
100	H	-0.0072	3.9818	3.6609
101	H	-0.0597	2.2274	3.8675
102	H	1.0051	2.9459	2.6472
103	H	-2.0662	3.1854	2.6226

TOL_INTDCO1

Energy (POTENTIAL) = -2312.27217133 Eh

	Atom	X	Y	Z
1	Cu	0.2233	-0.5138	-0.2975
2	N	1.0954	0.0798	-2.1940
3	C	1.2117	1.1992	-2.9244
4	C	2.1878	-0.4372	-4.0610
5	C	1.8979	0.9193	-4.1277
6	H	2.7082	-1.0872	-4.7504
7	H	2.1513	1.6116	-4.9174
8	N	-0.4963	-2.2349	-1.2753
9	C	-1.6357	-2.9437	-1.2748
10	C	-1.4749	-4.1117	-2.0517
11	C	-0.1705	-4.0454	-2.5243
12	H	-2.2128	-4.8759	-2.2483
13	H	0.3886	-4.7133	-3.1644
14	N	2.1138	-1.5816	0.0590
15	C	3.0105	-1.6680	1.0516
16	C	4.1066	-2.4604	0.6399
17	C	3.7999	-2.8380	-0.6617
18	H	4.9838	-2.7208	1.2143
19	H	4.3410	-3.4477	-1.3718
20	N	1.6969	-0.9185	-2.8955
21	N	2.6017	-2.2969	-0.9850
22	N	0.3955	-2.9129	-2.0489
23	H	2.3882	-3.0597	-3.0994
24	C	-0.1448	0.6583	1.2778
25	C	-1.2141	0.4401	2.1288
26	O	-1.9421	-0.0582	2.9402
27	C	-2.1136	1.8747	0.0847
28	H	-2.2044	0.8654	-0.3136
29	H	-1.5470	2.4736	-0.6332
30	B	1.8133	-2.3533	-2.3144
31	C	-3.4946	2.4671	0.3417
32	H	-3.4514	3.5130	0.6609
33	H	-4.0427	1.8895	1.0924
34	H	-4.0653	2.4363	-0.5917
35	C	-2.8417	-2.4119	-0.5969
36	C	-3.5093	-1.3091	-1.1698
37	C	-3.3104	-2.9839	0.6042
38	C	-4.6381	-0.7901	-0.5226
39	C	-4.4392	-2.4324	1.2209

40	C	-5.1135	-1.3323	0.6765
41	H	-5.1531	0.0611	-0.9621
42	H	-4.7889	-2.8578	2.1596
43	C	2.7467	-0.9672	2.3315
44	C	3.5297	0.1508	2.6981
45	C	1.6621	-1.3688	3.1402
46	C	3.2100	0.8496	3.8682
47	C	1.3793	-0.6429	4.3058
48	C	2.1393	0.4671	4.6869
49	H	3.8039	1.7201	4.1419
50	H	0.5324	-0.9450	4.9173
51	C	0.6900	2.4886	-2.4085
52	C	1.2936	3.0797	-1.2775
53	C	-0.4131	3.1146	-3.0315
54	C	0.7756	4.2859	-0.7847
55	C	-0.8976	4.3191	-2.5076
56	C	-0.3185	4.9206	-1.3823
57	H	1.2431	4.7411	0.0861
58	H	-1.7567	4.7926	-2.9793
59	C	-3.0135	-0.6882	-2.4572
60	H	-3.0030	-1.4212	-3.2725
61	H	-1.9847	-0.3279	-2.3539
62	H	-3.6481	0.1495	-2.7619
63	C	-2.5960	-4.1598	1.2295
64	H	-1.5208	-3.9713	1.3166
65	H	-2.7105	-5.0670	0.6248
66	H	-2.9874	-4.3706	2.2292
67	C	-6.2999	-0.7297	1.3916
68	H	-6.8066	0.0173	0.7723
69	H	-5.9864	-0.2376	2.3212
70	H	-7.0348	-1.4954	1.6660
71	C	0.7781	-2.5296	2.7462
72	H	1.3675	-3.4000	2.4390
73	H	0.1460	-2.2636	1.8919
74	H	0.1241	-2.8225	3.5723
75	C	4.6817	0.6136	1.8344
76	H	4.3971	0.6682	0.7790
77	H	5.5311	-0.0765	1.8964
78	H	5.0333	1.6017	2.1475
79	C	1.8208	1.2413	5.9446
80	H	0.8203	1.0048	6.3201
81	H	1.8723	2.3225	5.7715
82	H	2.5361	1.0084	6.7440
83	C	-1.0928	2.4822	-4.2246
84	H	-0.4409	2.4746	-5.1050
85	H	-2.0059	3.0243	-4.4891
86	H	-1.3600	1.4394	-4.0221
87	C	2.4807	2.4348	-0.5995
88	H	3.3001	2.2709	-1.3087
89	H	2.2209	1.4527	-0.1956
90	H	2.8573	3.0595	0.2160
91	C	-0.8829	6.2058	-0.8229
92	H	-0.8609	7.0092	-1.5690
93	H	-0.3189	6.5466	0.0511
94	H	-1.9299	6.0808	-0.5195
95	H	0.8142	0.9795	1.6780
96	N	-1.2760	1.7604	1.3077
97	C	-1.1158	3.0368	2.0681
98	H	-0.9583	3.8219	1.3223
99	C	0.0193	3.0455	3.0839
100	H	-0.0043	4.0031	3.6143
101	H	-0.0798	2.2503	3.8276
102	H	0.9989	2.9504	2.6089
103	H	-2.0691	3.2285	2.5703

TOL_TSDCO3

Energy (POTENTIAL) = -2312.27109816 Eh

	Atom	X	Y	Z
1	Cu	0.2290	-0.5151	-0.2519
2	N	1.0663	0.0762	-2.1723
3	C	1.1270	1.1713	-2.9474
4	C	2.1143	-0.4772	-4.0570
5	C	1.7844	0.8665	-4.1606
6	H	2.6284	-1.1382	-4.7406
7	H	1.9910	1.5383	-4.9811
8	N	-0.4835	-2.2317	-1.2125
9	C	-1.6485	-2.8969	-1.2594
10	C	-1.5076	-4.0497	-2.0632
11	C	-0.1911	-4.0173	-2.5035
12	H	-2.2670	-4.7811	-2.2984
13	H	0.3584	-4.6849	-3.1521
14	N	2.1463	-1.5831	0.0979
15	C	3.0651	-1.6534	1.0716
16	C	4.1711	-2.4170	0.6353
17	C	3.8467	-2.7978	-0.6615
18	H	5.0652	-2.6589	1.1918
19	H	4.3875	-3.3915	-1.3853
20	N	1.6744	-0.9298	-2.8608
21	N	2.6292	-2.2854	-0.9571
22	N	0.4033	-2.9202	-1.9827
23	H	2.3821	-3.0638	-3.0595
24	C	-0.0494	0.7848	1.2040
25	C	-1.0126	-0.2114	2.3215
26	O	-2.0506	-0.7209	2.3572
27	C	-1.8833	2.1113	0.2518
28	H	-1.9876	1.1713	-0.2920
29	H	-1.6922	2.8972	-0.4866
30	B	1.8167	-2.3572	-2.2680
31	C	-3.1650	2.4204	1.0302
32	H	-3.1069	3.3878	1.5401
33	H	-3.3827	1.6475	1.7723
34	H	-4.0083	2.4645	0.3332
35	C	-2.8620	-2.3474	-0.6079
36	C	-3.4512	-1.1768	-1.1356
37	C	-3.4288	-2.9842	0.5142
38	C	-4.6003	-0.6634	-0.5227
39	C	-4.5825	-2.4414	1.0923
40	C	-5.1811	-1.2802	0.5919
41	H	-5.0561	0.2376	-0.9284
42	H	-5.0115	-2.9257	1.9674
43	C	2.7963	-0.9874	2.3692
44	C	3.4802	0.1974	2.7170
45	C	1.7896	-1.5015	3.2136
46	C	3.1329	0.8571	3.9026
47	C	1.4803	-0.8160	4.3961
48	C	2.1357	0.3666	4.7558
49	H	3.6443	1.7829	4.1600
50	H	0.6938	-1.2054	5.0389
51	C	0.5878	2.4799	-2.5015
52	C	1.2108	3.1687	-1.4375
53	C	-0.5157	3.0564	-3.1671
54	C	0.7221	4.4267	-1.0647
55	C	-0.9758	4.3157	-2.7591
56	C	-0.3690	5.0190	-1.7118
57	H	1.2061	4.9573	-0.2477
58	H	-1.8303	4.7557	-3.2701
59	C	-2.8707	-0.4921	-2.3530
60	H	-2.8857	-1.1566	-3.2256
61	H	-1.8253	-0.2119	-2.1971
62	H	-3.4360	0.4101	-2.6048
63	C	-2.7895	-4.2175	1.1105
64	H	-1.7233	-4.0564	1.3054
65	H	-2.8607	-5.0783	0.4358
66	H	-3.2709	-4.4911	2.0543
67	C	-6.3908	-0.6793	1.2682
68	H	-7.0385	-1.4514	1.6971
69	H	-6.9892	-0.0840	0.5704
70	H	-6.0891	-0.0151	2.0893
71	C	1.0290	-2.7536	2.8373
72	H	1.7088	-3.5989	2.6791
73	H	0.4840	-2.6173	1.8972
74	H	0.3129	-3.0292	3.6169
75	C	4.5511	0.7703	1.8167
76	H	4.2283	0.7963	0.7715
77	H	5.4649	0.1652	1.8496
78	H	4.8160	1.7881	2.1199
79	C	1.7843	1.0961	6.0315
80	H	0.7858	0.8232	6.3882
81	H	1.8092	2.1824	5.8919
82	H	2.4955	0.8562	6.8327
83	C	-1.2044	2.3409	-4.3078
84	H	-0.6267	2.4207	-5.2365
85	H	-2.1941	2.7684	-4.4964
86	H	-1.3243	1.2736	-4.1019
87	C	2.3929	2.5694	-0.7131
88	H	3.2168	2.3588	-1.4049
89	H	2.1275	1.6159	-0.2498
90	H	2.7671	3.2440	0.0625
91	C	-0.9088	6.3562	-1.2614
92	H	-1.3049	6.9340	-2.1033
93	H	-0.1361	6.9576	-0.7712
94	H	-1.7299	6.2285	-0.5429
95	H	0.8623	0.8868	1.7981
96	N	-0.6854	1.9762	1.0877
97	C	-0.3038	3.1102	1.9449
98	H	0.7831	3.2283	1.8724
99	C	-0.7049	2.9333	3.4121
100	H	-0.3631	3.7959	3.9953
101	H	-1.7893	2.8481	3.5299
102	H	-0.2414	2.0373	3.8378
103	H	-0.7535	4.0132	1.5235

TOL_INTDCO2

Energy (POTENTIAL) = -2312.29071653 Eh

	Atom	X	Y	Z
1	Cu	0.2679	-0.4773	-0.2708
2	N	1.0691	0.0923	-2.1526
3	C	1.1432	1.1709	-2.9527
4	C	2.0484	-0.5298	-4.0532
5	C	1.7573	0.8208	-4.1743
6	H	2.5233	-1.2190	-4.7370
7	H	1.9630	1.4719	-5.0112
8	N	-0.5413	-2.1857	-1.1849
9	C	-1.7179	-2.8317	-1.1892
10	C	-1.6086	-4.0078	-1.9639
11	C	-0.3002	-4.0101	-2.4290
12	H	-2.3833	-4.7336	-2.1636
13	H	0.2272	-4.7050	-3.0674
14	N	2.1313	-1.5779	0.1154
15	C	3.0766	-1.6540	1.0664
16	C	4.1434	-2.4614	0.6196
17	C	3.7743	-2.8591	-0.6602
18	H	5.0454	-2.7128	1.1585
19	H	4.2797	-3.4815	-1.3853
20	N	1.6288	-0.9478	-2.8390
21	N	2.5665	-2.3183	-0.9377
22	N	0.3201	-2.9085	-1.9500
23	H	2.2893	-3.0971	-3.0347

24	C	-0.2542	0.7206	1.3718
25	C	-0.5644	-0.5659	1.7142
26	O	-1.0024	-1.5475	2.1853
27	C	-1.6555	2.3104	0.1407
28	H	-1.5121	1.5863	-0.6626
29	H	-0.9134	3.1070	-0.0144
30	B	1.7413	-2.3784	-2.2430
31	C	-3.0690	2.8838	0.0496
32	H	-3.2188	3.7442	0.7106
33	H	-3.8077	2.1181	0.3055
34	H	-3.2576	3.2265	-0.9736
35	C	-2.9050	-2.2515	-0.5163
36	C	-3.4863	-1.0748	-1.0414
37	C	-3.4498	-2.8618	0.6303
38	C	-4.6097	-0.5350	-0.4078
39	C	-4.5676	-2.2794	1.2430
40	C	-5.1604	-1.1164	0.7414
41	H	-5.0637	0.3653	-0.8164
42	H	-4.9771	-2.7415	2.1390
43	C	2.8725	-0.9300	2.3456
44	C	3.4403	0.3491	2.5338
45	C	2.0525	-1.4983	3.3420
46	C	3.1157	1.0692	3.6894
47	C	1.7515	-0.7432	4.4843
48	C	2.2600	0.5469	4.6689
49	H	3.5342	2.0649	3.8267
50	H	1.0958	-1.1710	5.2393
51	C	0.6681	2.5131	-2.5369
52	C	1.3708	3.2269	-1.5429
53	C	-0.4481	3.0982	-3.1719
54	C	0.9542	4.5215	-1.2104
55	C	-0.8432	4.3888	-2.7969
56	C	-0.1526	5.1204	-1.8239
57	H	1.5006	5.0706	-0.4461
58	H	-1.7121	4.8339	-3.2780
59	C	-2.9186	-0.4082	-2.2739
60	H	-2.9296	-1.0868	-3.1355
61	H	-1.8753	-0.1176	-2.1246
62	H	-3.4908	0.4858	-2.5370
63	C	-2.8355	-4.1142	1.2108
64	H	-1.7495	-4.0172	1.2989
65	H	-3.0311	-4.9883	0.5783
66	H	-3.2411	-4.3244	2.2052
67	C	-6.3425	-0.4777	1.4320
68	H	-7.1118	-0.1747	0.7125
69	H	-6.0358	0.4245	1.9772
70	H	-6.8038	-1.1586	2.1543
71	C	1.5302	-2.9090	3.1999
72	H	2.3490	-3.6316	3.3071
73	H	1.0803	-3.0803	2.2191
74	H	0.7804	-3.1323	3.9636
75	C	4.4130	0.9272	1.5312
76	H	4.1536	0.6609	0.5046
77	H	5.4245	0.5423	1.7146
78	H	4.4580	2.0177	1.6054
79	C	1.9058	1.3649	5.8886
80	H	1.1654	0.8567	6.5136
81	H	1.4930	2.3400	5.6042
82	H	2.7910	1.5577	6.5073
83	C	-1.2143	2.3629	-4.2487
84	H	-0.6863	2.4034	-5.2091
85	H	-2.2037	2.8067	-4.3973
86	H	-1.3459	1.3052	-4.0056
87	C	2.5390	2.5987	-0.8245
88	H	3.2784	2.1969	-1.5260
89	H	2.2056	1.7571	-0.2096
90	H	3.0387	3.3221	-0.1745
91	C	-0.6218	6.4957	-1.4120
92	H	-1.0453	7.0471	-2.2583
93	H	0.1959	7.0912	-0.9932
94	H	-1.4043	6.4279	-0.6443
95	H	0.7470	1.0901	1.6179
96	N	-1.4063	1.5917	1.4013
97	C	-1.3719	2.4585	2.5901
98	H	-0.4504	3.0739	2.5913
99	C	-1.4776	1.6818	3.9019
100	H	-1.5253	2.3801	4.7451
101	H	-2.3833	1.0656	3.9113
102	H	-0.6155	1.0274	4.0616
103	H	-2.2164	3.1493	2.5192

TOL_INTDiazo

Energy (POTENTIAL) = -2421.81281832 Eh

	Atom	X	Y	Z
1	Cu	-1.3165	-0.0434	-0.1829
2	N	-2.6731	0.8522	-1.4497
3	C	-2.6478	1.8868	-2.3008
4	C	-4.6959	1.0234	-2.3369
5	C	-3.9224	2.0379	-2.8903
6	H	-5.7291	0.7500	-2.4992
7	H	-4.2255	2.7746	-3.6200
8	N	-2.7430	-0.2371	1.2851
9	C	-2.7671	-0.1201	2.6201
10	C	-4.0620	-0.4252	3.0931
11	C	-4.7958	-0.7323	1.9523
12	H	-4.4063	-0.4173	4.1168
13	H	-5.8289	-1.0265	1.8314
14	N	-1.9637	-1.9311	-0.9513
15	C	-1.3877	-3.0582	-1.3870
16	C	-2.3874	-3.9729	-1.7902
17	C	-3.5889	-3.3123	-1.5578
18	H	-2.2482	-4.9687	-2.1853
19	H	-4.6122	-3.6250	-1.7122
20	N	-3.9250	0.3236	-1.4730
21	N	-3.3090	-2.0871	-1.0544
22	N	-3.9832	-0.6137	0.8775
23	H	-5.4009	-1.2497	-0.7748
24	N	0.4502	0.2818	0.2486
25	N	1.5046	0.2114	0.6901
26	C	2.7065	0.1345	1.1777
27	C	3.8277	0.3133	0.2357
28	O	3.6250	0.6693	-0.9353
29	C	6.2182	0.4207	-0.1663
30	H	5.9929	0.0520	-1.1708
31	H	7.0949	-0.1213	0.2005
32	B	-4.2531	-0.9319	-0.6221
33	C	6.5048	1.9245	-0.2298
34	H	6.7665	2.3259	0.7545
35	H	5.6286	2.4612	-0.6043
36	H	7.3411	2.1188	-0.9118
37	C	-1.5153	0.2473	3.3266
38	C	-0.9321	1.5159	3.1152
39	C	-0.8573	-0.7117	4.1298
40	C	0.3039	1.8012	3.7120
41	C	0.3760	-0.3849	4.7054
42	C	0.9727	0.8677	4.5103
43	H	0.7561	2.7761	3.5418
44	H	0.8859	-1.1285	5.3150
45	C	0.0942	-3.1411	-1.3766
46	C	0.7829	-3.1402	-0.1443
47	C	0.8143	-3.1213	-2.5913
48	C	2.1832	-3.1075	-0.1510
49	C	2.2127	-3.0845	-2.5513
50	C	2.9164	-3.0677	-1.3409
51	H	2.7135	-3.0995	0.7987
52	H	2.7664	-3.0454	-3.4874
53	C	-1.3772	2.6328	-2.4732
54	C	-0.2700	1.9892	-3.0732
55	C	-1.2453	3.9358	-1.9518
56	C	0.9533	2.6640	-3.1307
57	C	-0.0002	4.5754	-2.0293
58	C	1.1119	3.9533	-2.6074
59	H	1.8116	2.1622	-3.5718
60	H	0.1058	5.5751	-1.6127
61	C	-1.5860	2.5570	2.2343
62	H	-2.6769	2.5242	2.3020
63	H	-1.3282	2.3951	1.1815
64	H	-1.2513	3.5630	2.5059
65	C	-1.4517	-2.0832	4.3573
66	H	-1.8139	-2.5266	3.4250
67	H	-2.3077	-2.0394	5.0411
68	H	-0.7127	-2.7616	4.7943
69	C	2.2904	1.2058	5.1674
70	H	2.9603	0.3395	5.2003
71	H	2.1381	1.5320	6.2045
72	H	2.8054	2.0174	4.6440
73	C	0.0963	-3.1065	-3.9220
74	H	-0.6953	-2.3510	-3.9412
75	H	-0.3806	-4.0709	-4.1335
76	H	0.7917	-2.8915	-4.7389
77	C	0.0412	-3.1531	1.1736
78	H	-0.7408	-3.9199	1.1880
79	H	-0.4575	-2.1973	1.3610
80	H	0.7254	-3.3461	2.0053
81	C	4.4215	-2.9488	-1.3267
82	H	4.8392	-3.2277	-0.3538
83	H	4.7210	-1.9145	-1.5336
84	H	4.8833	-3.5848	-2.0902
85	C	-2.4110	4.6254	-1.2788
86	H	-2.8435	3.9989	-0.4905
87	H	-3.2183	4.8412	-1.9879
88	H	-2.0991	5.5725	-0.8282
89	C	-0.3761	0.5819	-3.6166
90	H	-1.2986	0.4367	-4.1885
91	H	-0.3875	-0.1532	-2.8054
92	H	0.4744	0.3475	-4.2631
93	C	2.4580	4.6363	-2.6639
94	H	3.2339	4.0051	-2.2150
95	H	2.4476	5.5929	-2.1316
96	H	2.7612	4.8334	-3.6999
97	H	2.7644	-0.0173	2.2462
98	N	5.0922	0.0740	0.7133
99	C	5.3783	-0.4127	2.0654
100	H	6.2709	-1.0449	2.0034
101	H	4.5675	-1.0732	2.3843
102	C	5.5990	0.7007	3.0952
103	H	4.7375	1.3749	3.1318
104	H	5.7467	0.2728	4.0935
105	H	6.4834	1.2964	2.8492

TOL_TSN2

Energy (POTENTIAL) = -2421.78496239 Eh

	Atom	X	Y	Z
1	Cu	1.2595	0.3527	0.0769
2	N	1.2651	1.9695	1.7269
3	C	0.4806	2.9731	2.1431
4	C	2.1109	2.7483	3.6323
5	C	0.9744	3.4992	3.3594

6	H	2.8185	2.7978	4.4482
7	H	0.5655	4.3157	3.9371
8	N	1.8156	-1.0146	1.5706
9	C	1.5497	-2.3054	1.8293
10	C	2.3706	-2.7606	2.8835
11	C	3.1373	-1.6594	3.2383
12	H	2.3824	-3.7476	3.3222
13	H	3.8964	-1.5355	3.9978
14	N	3.3055	0.8207	-0.1364
15	C	4.1375	1.0692	-1.1609
16	C	5.3988	1.4678	-0.6679
17	C	5.2680	1.4273	0.7134
18	H	6.2755	1.7232	-1.2448
19	H	5.9773	1.6379	1.5013
20	N	2.2611	1.8408	2.6384
21	N	4.0091	1.0367	1.0095
22	N	2.7886	-0.6258	2.4405
23	H	4.2221	0.9129	3.2549
24	C	-0.3661	0.6587	-0.8763
25	C	-1.4726	-0.2919	-1.2528
26	O	-2.4617	-0.2560	-0.5032
27	C	-2.5579	-1.9912	-2.5830
28	H	-2.2160	-2.8429	-3.1771
29	N	0.0677	1.5250	-2.3961
30	N	0.5754	2.2754	-3.0391
31	B	3.3771	0.8036	2.4071
32	C	-3.6473	-1.2146	-3.3262
33	H	-4.5058	-1.8671	-3.5242
34	H	-3.9935	-0.3731	-2.7193
35	H	-3.2822	-0.8273	-4.2831
36	C	-0.6606	3.4126	1.3030
37	C	-1.9892	3.1341	1.6892
38	C	-0.3981	4.0610	0.0772
39	C	-3.0349	3.5111	0.8393
40	C	-1.4743	4.4198	-0.7466
41	C	-2.7993	4.1530	-0.3835
42	H	-4.0579	3.2764	1.1263
43	H	-1.2708	4.9134	-1.6949
44	C	0.4967	-3.0426	1.0928
45	C	-0.8563	-2.6924	1.2893
46	C	0.8408	-4.1056	0.2290
47	C	-1.8469	-3.4258	0.6245
48	C	-0.1812	-4.8037	-0.4249
49	C	-1.5316	-4.4850	-0.2321
50	H	-2.8878	-3.1503	0.7730
51	H	0.0819	-5.6199	-1.0960
52	C	3.7292	0.8420	-2.5667
53	C	3.4776	-0.4764	-3.0082
54	C	3.6460	1.9202	-3.4729
55	C	3.1655	-0.6907	-4.3558
56	C	3.3101	1.6623	-4.8082
57	C	3.0720	0.3638	-5.2717
58	H	2.9913	-1.7090	-4.6967
59	H	3.2298	2.4968	-5.5020
60	C	3.5398	-1.6481	-2.0557
61	H	4.4843	-1.6665	-1.5005
62	H	2.7443	-1.5911	-1.3061
63	H	3.4359	-2.5957	-2.5919
64	C	3.8976	3.3420	-3.0249
65	H	3.4629	3.5390	-2.0408
66	H	4.9710	3.5524	-2.9447
67	H	3.4731	4.0564	-3.7371
68	C	2.6828	0.1161	-6.7098
69	H	3.2421	0.7639	-7.3935
70	H	2.8646	-0.9228	-7.0037
71	H	1.6160	0.3215	-6.8688
72	C	1.0219	4.3277	-0.3686
73	H	1.5789	4.8929	0.3873
74	H	1.5690	3.3908	-0.5149
75	H	1.0425	4.8952	-1.3036
76	C	-2.2800	2.3996	2.9770
77	H	-1.7322	1.4520	3.0203
78	H	-1.9751	2.9837	3.8526
79	H	-3.3472	2.1787	3.0704
80	C	-3.9540	4.5365	-1.2792
81	H	-3.6057	4.9595	-2.2269
82	H	-4.5820	3.6671	-1.5076
83	H	-4.5986	5.2820	-0.7973
84	C	2.2824	-4.5091	0.0106
85	H	2.3930	-5.0717	-0.9221
86	H	2.9488	-3.6436	-0.0292
87	H	2.6431	-5.1500	0.8248
88	C	-1.2390	-1.5468	2.1961
89	H	-0.7638	-1.6404	3.1792
90	H	-0.9140	-0.5920	1.7735
91	H	-2.3220	-1.5001	2.3321
92	C	-2.6124	-5.2708	-0.9378
93	H	-2.4422	-5.3028	-2.0207
94	H	-2.6386	-6.3102	-0.5871
95	H	-3.6026	-4.8379	-0.7657
96	H	-0.8662	1.5554	-0.4995
97	N	-1.3745	-1.1673	-2.2850
98	C	-0.1550	-1.4298	-3.0525
99	H	0.6704	-0.8725	-2.6083
100	C	-0.3067	-1.0965	-4.5385
101	H	0.6168	-1.3354	-5.0704
102	H	-1.1155	-1.6754	-4.9954
103	H	-0.5225	-0.0338	-4.6889
104	H	0.0946	-2.4920	-2.9320
105	H	-2.9514	-2.3804	-1.6431

TOL_N2

Energy (POTENTIAL) = -109.521440025 Eh

	Atom	X	Y	Z
1	N	-1.7377	-0.0018	-0.3640
2	N	-2.4452	-0.8260	-0.5659

TOL_Diazo

Energy (POTENTIAL) = -474.745299153 Eh

	Atom	X	Y	Z
1	N	0.0781	2.1308	1.9680
2	C	0.6149	0.9720	1.6969
3	C	1.1258	0.7957	0.3231
4	O	1.0384	1.7217	-0.4991
5	N	-0.3872	3.1478	2.1771
6	H	0.6171	0.2592	2.5083
7	N	1.7002	-0.4120	0.0214
8	C	1.7227	-1.5546	0.9433
9	H	2.5115	-2.2259	0.5923
10	H	2.0405	-1.2158	1.9356
11	C	2.2282	-0.5982	-1.3377
12	H	3.1194	-1.2309	-1.2595
13	H	2.5422	0.3806	-1.7043
14	C	0.3986	-2.3227	1.0397
15	H	0.1102	-2.7383	0.0702
16	H	0.4976	-3.1505	1.7519
17	H	-0.4156	-1.6759	1.3820
18	C	1.2205	-1.2155	-2.3122
19	H	1.6531	-1.2601	-3.3186
20	H	0.9447	-2.2342	-2.0209
21	H	0.3107	-0.6087	-2.3587

TOL_CU

Energy (POTENTIAL) = -1947.02306159 Eh

	Atom	X	Y	Z
1	Cu	0.0076	0.0053	-0.3617
2	N	-1.5490	0.8148	-1.4253
3	C	-2.7379	1.4191	-1.2887
4	C	-2.3289	1.2205	-3.4639
5	C	-3.2754	1.6961	-2.5660
6	H	-2.3130	1.2215	-4.5447
7	H	-4.2123	2.1840	-2.7917
8	N	1.4776	0.9524	-1.4288
9	C	2.5933	1.6815	-1.2912
10	C	3.0924	2.0244	-2.5681
11	C	2.2036	1.4481	-3.4666
12	H	3.9780	2.6003	-2.7933
13	H	2.1893	1.4450	-4.5474
14	N	0.0907	-1.7393	-1.4438
15	C	0.1468	-3.0736	-1.3217
16	C	0.1578	-3.6635	-2.6063
17	C	0.1035	-2.5956	-3.4919
18	H	0.1919	-4.7163	-2.8448
19	H	0.0874	-2.5703	-4.5724
20	N	-1.3002	0.6934	-2.7599
21	N	0.0665	-1.4483	-2.7748
22	N	1.2397	0.8094	-2.7629
23	H	0.0004	0.0275	-4.4814
24	B	0.0025	0.0211	-3.2805
25	C	3.1029	1.9897	0.0661
26	C	3.1054	3.3206	0.5358
27	C	3.5389	0.9385	0.9062
28	C	3.5498	3.5805	1.8389
29	C	3.9765	1.2441	2.2004
30	C	3.9906	2.5577	2.6862
31	H	3.5426	4.6059	2.2026
32	H	4.3159	0.4362	2.8458
33	C	-3.2737	1.7014	0.0647
34	C	-4.4477	1.0514	0.5115
35	C	-2.5881	2.5910	0.9198
36	C	-4.9155	1.3077	1.8045
37	C	-3.0940	2.8200	2.2078
38	C	-4.2542	2.1909	2.6688
39	H	-5.8138	0.7999	2.1509
40	H	-2.5664	3.5096	2.8634
41	C	0.1850	-3.6932	0.0244
42	C	-0.8936	-3.5019	0.9204
43	C	1.3104	-4.4464	0.4238
44	C	-0.8210	-4.0669	2.1987
45	C	1.3396	-4.9948	1.7137
46	C	0.2868	-4.8152	2.6168
47	H	-1.6533	-3.9229	2.8853
48	H	2.2116	-5.5684	2.0210
49	C	-2.1179	-2.7040	0.5286
50	H	-1.9294	-1.6259	0.5906
51	H	-2.4195	-2.9031	-0.5042
52	H	-2.9606	-2.9320	1.1881
53	C	0.3349	-5.3996	4.0092
54	H	0.3253	-4.6095	4.7705
55	H	-0.5331	-6.0416	4.2015
56	H	1.2370	-6.0000	4.1630
57	C	2.4898	-4.6512	-0.5008
58	H	2.2414	-5.3189	-1.3336
59	H	2.8231	-3.7056	-0.9420
60	H	3.3340	-5.0929	0.0373
61	C	-5.1825	0.0696	-0.3733
62	H	-5.6574	0.5679	-1.2259
63	H	-4.5026	-0.6835	-0.7862

64	H	-5.9661	-0.4497	0.1866
65	C	-1.3218	3.2930	0.4808
66	H	-0.4636	2.6118	0.4944
67	H	-1.4006	3.6688	-0.5444
68	H	-1.0917	4.1337	1.1423
69	C	-4.7967	2.4551	4.0537
70	H	-5.7625	2.9738	4.0077
71	H	-4.9603	1.5196	4.6018
72	H	-4.1140	3.0752	4.6430
73	C	2.6150	4.4582	-0.3323
74	H	3.2985	4.6543	-1.1661
75	H	1.6368	4.2345	-0.7718
76	H	2.5250	5.3813	0.2483
77	C	3.5379	-0.4992	0.4341
78	H	2.5233	-0.9131	0.4085
79	H	3.9320	-0.5897	-0.5835
80	H	4.1405	-1.1287	1.0959
81	C	4.4961	2.8588	4.0775
82	H	4.1435	2.1158	4.8017
83	H	5.5935	2.8449	4.1076
84	H	4.1709	3.8465	4.4200

TOL_THF

Energy (POTENTIAL) = -232.473791462 Eh

	Atom	X	Y	Z
1	C	-0.6140	-0.5813	-0.0000
2	O	0.8192	-0.5621	-0.0000
3	C	1.3402	0.7732	0.0000
4	C	0.1526	1.7595	-0.0000
5	C	-1.1208	0.8767	-0.0000
6	H	-0.9520	-1.1326	-0.8875
7	H	-0.9520	-1.1326	0.8875
8	H	1.9746	0.8966	0.8876
9	H	1.9746	0.8966	-0.8876
10	H	0.1841	2.4080	0.8805
11	H	0.1841	2.4080	-0.8805
12	H	-1.7389	1.0752	0.8804
13	H	-1.7389	1.0752	-0.8804

Potencial Energies (hartree) and Cartesian Coordinates (Ǻ) of all relevant species in Dichloromethane.

DCM_CuTHF
Energy (POTENTIAL) = -2179.54778470 Eh

	Atom	X	Y	Z
1	Cu	-0.0174	0.0336	-0.5430
2	N	-0.3359	1.8594	-1.5844
3	C	-0.5930	3.1448	-1.2996
4	C	-0.7314	2.9116	-3.5043
5	C	-0.8550	3.8565	-2.4931
6	H	-0.8390	3.0004	-4.5766
7	H	-1.0922	4.9060	-2.5943
8	N	1.5074	-0.4621	-1.9424
9	C	2.7684	-0.9165	-1.9185
10	C	3.2910	-0.9441	-3.2320
11	C	2.2540	-0.4846	-4.0361
12	H	4.2760	-1.2623	-3.5426
13	H	2.1945	-0.3436	-5.1067
14	N	-1.4656	-0.8761	-1.7797
15	C	-2.5244	-1.6887	-1.6489
16	C	-3.0548	-1.9919	-2.9239
17	C	-2.2489	-1.2975	-3.8181
18	H	-3.9074	-2.6164	-3.1501
19	H	-2.2860	-1.2223	-4.8962
20	N	-0.4196	1.7225	-2.9363
21	N	-1.3024	-0.6363	-3.1100
22	N	1.1951	-0.2021	-3.2397
23	H	-0.2907	0.4608	-4.8062
24	B	-0.2069	0.3424	-3.6134
25	C	-0.2045	-1.5373	2.1305
26	O	0.2382	-0.3293	1.4668
27	C	1.3119	0.2719	2.2488
28	C	1.2981	-0.4427	3.6130
29	C	-0.0369	-1.2144	3.6075
30	H	-1.2284	-1.7205	1.8124
31	H	0.4318	-2.3767	1.8256
32	H	2.2551	0.1357	1.7126
33	H	1.0820	1.3361	2.3210
34	H	2.1366	-1.1418	3.6820
35	H	1.3732	0.2652	4.4430
36	H	-0.0133	-2.1123	4.2318
37	H	-0.8620	-0.5755	3.9419
38	C	-0.5518	3.6176	0.1057
39	C	-1.7350	4.0418	0.7487
40	C	0.6744	3.6129	0.8087
41	C	-1.6721	4.4521	2.0878
42	C	0.6945	4.0398	2.1428
43	C	-0.4674	4.4627	2.8014
44	H	-2.5873	4.7641	2.5870
45	H	1.6393	4.0330	2.6831
46	C	3.3626	-1.3215	-0.6215
47	C	4.4187	-0.5753	-0.0523
48	C	2.8183	-2.4248	0.0714
49	C	4.9055	-0.9407	1.2087
50	C	3.3350	-2.7561	1.3322
51	C	4.3732	-2.0253	1.9197
52	H	5.7097	-0.3577	1.6540
53	H	2.9099	-3.6018	1.8687
54	C	-2.9861	-2.0888	-0.2972
55	C	-2.8620	-3.4263	0.1329
56	C	-3.5179	-1.1084	0.5738
57	C	-3.2679	-3.7644	1.4327
58	C	-3.9177	-1.4898	1.8602
59	C	-3.7952	-2.8113	2.3119
60	H	-3.1586	-4.7938	1.7677
61	H	-4.3226	-0.7347	2.5314
62	C	-3.0617	4.0508	0.0225
63	H	-3.1230	4.8871	-0.6842
64	H	-3.2106	3.1344	-0.5564
65	H	-3.8916	4.1510	0.7286
66	C	1.9491	3.1322	0.1533
67	H	1.8981	2.0593	-0.0567

68	H	2.1209	3.6304	-0.8075
69	H	2.8144	3.3149	0.7972
70	C	-0.4147	4.9443	4.2325
71	H	-0.1897	6.0183	4.2755
72	H	-1.3715	4.7912	4.7429
73	H	0.3653	4.4263	4.8008
74	C	1.6875	-3.2389	-0.5164
75	H	0.7554	-2.6644	-0.5346
76	H	1.8974	-3.5240	-1.5534
77	H	1.5187	-4.1508	0.0638
78	C	4.9995	0.6226	-0.7696
79	H	5.5275	0.3269	-1.6833
80	H	4.2145	1.3257	-1.0703
81	H	5.7083	1.1555	-0.1286
82	C	4.8996	-2.3731	3.2925
83	H	5.9710	-2.6060	3.2615
84	H	4.7748	-1.5328	3.9865
85	H	4.3787	-3.2380	3.7148
86	C	-3.6300	0.3385	0.1501
87	H	-2.6393	0.8003	0.0795
88	H	-4.0910	0.4351	-0.8390
89	H	-4.2239	0.9118	0.8678
90	C	-2.2747	-4.4844	-0.7738
91	H	-2.9302	-4.6902	-1.6277
92	H	-1.3099	-4.1656	-1.1840
93	H	-2.1233	-5.4233	-0.2328
94	C	-4.1887	-3.1774	3.7240
95	H	-3.4648	-2.7769	4.4457
96	H	-5.1679	-2.7608	3.9864
97	H	-4.2302	-4.2624	3.8623

DCM_Cu

Energy (POTENTIAL) = -1947.03432408 Eh

	Atom	X	Y	Z
1	Cu	-0.0076	0.0103	-0.3247
2	N	-1.5524	0.8182	-1.4339
3	C	-2.7390	1.4298	-1.3044
4	C	-2.3172	1.2357	-3.4783
5	C	-3.2671	1.7145	-2.5847
6	H	-2.2947	1.2395	-4.5593
7	H	-4.2020	2.2048	-2.8154
8	N	1.4737	0.9461	-1.4205
9	C	2.5927	1.6707	-1.2802
10	C	3.1008	2.0089	-2.5555
11	C	2.2137	1.4348	-3.4577
12	H	3.9885	2.5835	-2.7775
13	H	2.2053	1.4300	-4.5390
14	N	0.0851	-1.7361	-1.4418
15	C	0.1359	-3.0710	-1.3183
16	C	0.1386	-3.6643	-2.6024
17	C	0.0863	-2.5976	-3.4902
18	H	0.1721	-4.7179	-2.8393
19	H	0.0676	-2.5745	-4.5711
20	N	-1.2966	0.6998	-2.7674
21	N	0.0582	-1.4486	-2.7734
22	N	1.2430	0.8021	-2.7562
23	H	0.0093	0.0259	-4.4810
24	B	0.0047	0.0201	-3.2805
25	C	3.1002	1.9807	0.0780
26	C	3.1131	3.3145	0.5415
27	C	3.5273	0.9304	0.9247
28	C	3.5591	3.5781	1.8441
29	C	3.9681	1.2392	2.2180
30	C	3.9929	2.5558	2.6971
31	H	3.5594	4.6054	2.2024
32	H	4.3010	0.4317	2.8673
33	C	-3.2854	1.7079	0.0461
34	C	-4.4678	1.0617	0.4779
35	C	-2.6027	2.5879	0.9144
36	C	-4.9471	1.3123	1.7686

37	C	-3.1202	2.8126	2.1994
38	C	-4.2893	2.1872	2.6452
39	H	-5.8517	0.8072	2.1025
40	H	-2.5950	3.4951	2.8644
41	C	0.1893	-3.6897	0.0284
42	C	-0.8700	-3.4866	0.9462
43	C	1.3155	-4.4527	0.4092
44	C	-0.7798	-4.0525	2.2237
45	C	1.3635	-5.0014	1.6994
46	C	0.3286	-4.8121	2.6221
47	H	-1.5979	-3.8994	2.9252
48	H	2.2362	-5.5821	1.9909
49	C	-2.0923	-2.6739	0.5798
50	H	-1.8729	-1.5995	0.5953
51	H	-2.4429	-2.9017	-0.4317
52	H	-2.9099	-2.8607	1.2823
53	C	0.3954	-5.3998	4.0125
54	H	0.3799	-4.6115	4.7755
55	H	-0.4637	-6.0525	4.2087
56	H	1.3068	-5.9885	4.1558
57	C	2.4785	-4.6674	-0.5340
58	H	2.2082	-5.3270	-1.3662
59	H	2.8161	-3.7222	-0.9739
60	H	3.3243	-5.1221	-0.0096
61	C	-5.2011	0.0907	-0.4201
62	H	-5.6610	0.5993	-1.2749
63	H	-4.5219	-0.6657	-0.8290
64	H	-5.9947	-0.4244	0.1292
65	C	-1.3259	3.2830	0.4951
66	H	-0.4777	2.5889	0.5060
67	H	-1.3925	3.6745	-0.5253
68	H	-1.0927	4.1112	1.1710
69	C	-4.8430	2.4459	4.0269
70	H	-5.8100	2.9615	3.9738
71	H	-5.0094	1.5076	4.5695
72	H	-4.1654	3.0661	4.6219
73	C	2.6314	4.4523	-0.3314
74	H	3.3104	4.6302	-1.1730
75	H	1.6455	4.2383	-0.7592
76	H	2.5605	5.3806	0.2435
77	C	3.5111	-0.5110	0.4645
78	H	2.4906	-0.9112	0.4486
79	H	3.8994	-0.6147	-0.5542
80	H	4.1103	-1.1398	1.1299
81	C	4.5013	2.8608	4.0866
82	H	4.1524	2.1173	4.8120
83	H	5.5988	2.8481	4.1127
84	H	4.1755	3.8494	4.4259

DCM_THF

Energy (POTENTIAL) = -232.475436204 Eh

	Atom	X	Y	Z
1	C	-0.6171	-0.5818	-0.0000
2	O	0.8206	-0.5641	-0.0000
3	C	1.3417	0.7760	0.0000
4	C	0.1530	1.7592	-0.0000
5	C	-1.1206	0.8763	-0.0000
6	H	-0.9541	-1.1316	-0.8882
7	H	-0.9541	-1.1316	0.8882
8	H	1.9744	0.8988	0.8883
9	H	1.9744	0.8988	-0.8883
10	H	0.1845	2.4060	0.8815
11	H	0.1845	2.4060	-0.8815
12	H	-1.7371	1.0742	0.8814
13	H	-1.7371	1.0742	-0.8814

DCM_N2

Energy (POTENTIAL) = -109.521919829 Eh

	Atom	X	Y	Z
1	N	-1.7378	-0.0019	-0.3640

2	N	-2.4451	-0.8260	-0.5659

DCM_Diazo
Energy (POTENTIAL) = -474.750581544 Eh

	Atom	X	Y	Z
1	N	0.0724	2.1247	1.9613
2	C	0.6156	0.9697	1.6952
3	C	1.1271	0.7915	0.3232
4	O	1.0376	1.7222	-0.5020
5	N	-0.3992	3.1406	2.1676
6	H	0.6209	0.2586	2.5090
7	N	1.7017	-0.4102	0.0224
8	C	1.7256	-1.5546	0.9475
9	H	2.5160	-2.2225	0.5954
10	H	2.0389	-1.2126	1.9391
11	C	2.2295	-0.6024	-1.3375
12	H	3.1086	-1.2500	-1.2562
13	H	2.5618	0.3707	-1.7040
14	C	0.4024	-2.3231	1.0399
15	H	0.1169	-2.7382	0.0691
16	H	0.5057	-3.1508	1.7513
17	H	-0.4113	-1.6777	1.3861
18	C	1.2143	-1.2059	-2.3125
19	H	1.6508	-1.2605	-3.3168
20	H	0.9236	-2.2185	-2.0154
21	H	0.3126	-0.5868	-2.3639

DCM_TSN2
Energy (POTENTIAL) = -2421.79589091 Eh

	Atom	X	Y	Z
1	Cu	1.2704	0.3340	0.0502
2	N	1.2781	1.9865	1.7175
3	C	0.4847	2.9800	2.1412
4	C	2.1157	2.7564	3.6320
5	C	0.9730	3.5010	3.3631
6	H	2.8208	2.8048	4.4506
7	H	0.5556	4.3080	3.9487
8	N	1.8125	-1.0045	1.5820
9	C	1.5456	-2.2943	1.8463
10	C	2.3671	-2.7468	2.9019
11	C	3.1362	-1.6450	3.2519
12	H	2.3807	-3.7332	3.3428
13	H	3.8968	-1.5205	4.0103
14	N	3.3250	0.8049	-0.1408
15	C	4.1612	1.0513	-1.1629
16	C	5.4197	1.4561	-0.6656
17	C	5.2832	1.4233	0.7156
18	H	6.2973	1.7150	-1.2402
19	H	5.9889	1.6412	1.5052
20	N	2.2739	1.8578	2.6299
21	N	4.0228	1.0305	1.0080
22	N	2.7877	-0.6136	2.4498
23	H	4.2286	0.9228	3.2541
24	C	-0.3425	0.6714	-0.9137
25	C	-1.4629	-0.2636	-1.2799
26	O	-2.4543	-0.1935	-0.5289
27	C	-2.5694	-1.9783	-2.5772
28	H	-2.2345	-2.8402	-3.1594
29	N	0.1146	1.5112	-2.4456
30	N	0.6163	2.2688	-3.0840
31	B	3.3856	0.8127	2.4051
32	C	-3.6499	-1.2028	-3.3346
33	H	-4.5131	-1.8523	-3.5210
34	H	-3.9894	-0.3457	-2.7452
35	H	-3.2792	-0.8380	-4.2978
36	C	-0.6666	3.4061	1.3069
37	C	-1.9885	3.0998	1.6976
38	C	-0.4225	4.0608	0.0803
39	C	-3.0458	3.4577	0.8531
40	C	-1.5098	4.4024	-0.7373

41	C	-2.8282	4.1090	-0.3690
42	H	-4.0631	3.2050	1.1458
43	H	-1.3197	4.8989	-1.6868
44	C	0.4917	-3.0331	1.1119
45	C	-0.8618	-2.6797	1.3022
46	C	0.8366	-4.0998	0.2518
47	C	-1.8521	-3.4134	0.6356
48	C	-0.1846	-4.7968	-0.4055
49	C	-1.5357	-4.4738	-0.2201
50	H	-2.8939	-3.1382	0.7795
51	H	0.0799	-5.6119	-1.0771
52	C	3.7566	0.8294	-2.5710
53	C	3.4838	-0.4829	-3.0184
54	C	3.6900	1.9133	-3.4730
55	C	3.1636	-0.6863	-4.3665
56	C	3.3488	1.6670	-4.8094
57	C	3.0851	0.3740	-5.2776
58	H	2.9660	-1.6991	-4.7101
59	H	3.2808	2.5062	-5.4988
60	C	3.5235	-1.6620	-2.0739
61	H	4.4539	-1.6867	-1.4955
62	H	2.7075	-1.6086	-1.3463
63	H	3.4300	-2.6044	-2.6209
64	C	3.9558	3.3297	-3.0158
65	H	3.4569	3.5444	-2.0654
66	H	5.0264	3.5076	-2.8592
67	H	3.6032	4.0510	-3.7593
68	C	2.6814	0.1385	-6.7136
69	H	3.2208	0.8045	-7.3957
70	H	2.8723	-0.8949	-7.0206
71	H	1.6092	0.3300	-6.8527
72	C	0.9889	4.3494	-0.3787
73	H	1.5510	4.9090	0.3778
74	H	1.5410	3.4197	-0.5498
75	H	0.9898	4.9292	-1.3062
76	C	-2.2608	2.3609	2.9874
77	H	-1.6955	1.4231	3.0297
78	H	-1.9635	2.9525	3.8606
79	H	-3.3240	2.1224	3.0844
80	C	-3.9937	4.4781	-1.2571
81	H	-3.6551	4.8850	-2.2151
82	H	-4.6254	3.6055	-1.4621
83	H	-4.6317	5.2316	-0.7785
84	C	2.2785	-4.5042	0.0367
85	H	2.3861	-5.0832	-0.8859
86	H	2.9411	-3.6366	-0.0223
87	H	2.6426	-5.1279	0.8626
88	C	-1.2470	-1.5233	2.1949
89	H	-0.7439	-1.5802	3.1667
90	H	-0.9580	-0.5723	1.7380
91	H	-2.3271	-1.5003	2.3613
92	C	-2.6145	-5.2505	-0.9382
93	H	-2.4457	-5.2544	-2.0217
94	H	-2.6312	-6.2981	-0.6124
95	H	-3.6056	-4.8258	-0.7524
96	H	-0.8247	1.5853	-0.5565
97	N	-1.3763	-1.1630	-2.2875
98	C	-0.1637	-1.4359	-3.0668
99	H	0.6701	-0.8899	-2.6246
100	C	-0.3213	-1.0976	-4.5507
101	H	0.6016	-1.3359	-5.0845
102	H	-1.1314	-1.6770	-5.0041
103	H	-0.5383	-0.0346	-4.6973
104	H	0.0713	-2.5016	-2.9525
105	H	-2.9685	-2.3518	-1.6330

DCM_INTDiazo
Energy (POTENTIAL) = -2421.82507113 Eh

	Atom	X	Y	Z
1	Cu	-1.3147	-0.0179	-0.1161

2	N	-2.6647	0.8697	-1.4243
3	C	-2.6356	1.9057	-2.2734
4	C	-4.6654	1.0025	-2.3710
5	C	-3.8957	2.0334	-2.9005
6	H	-5.6884	0.7101	-2.5640
7	H	-4.1932	2.7670	-3.6360
8	N	-2.7890	-0.2487	1.2984
9	C	-2.8511	-0.1242	2.6317
10	C	-4.1558	-0.4403	3.0702
11	C	-4.8559	-0.7596	1.9104
12	H	-4.5284	-0.4351	4.0844
13	H	-5.8831	-1.0640	1.7634
14	N	-1.9062	-1.9047	-0.9349
15	C	-1.3052	-3.0184	-1.3737
16	C	-2.2833	-3.9460	-1.8021
17	C	-3.4996	-3.3083	-1.5839
18	H	-2.1204	-4.9348	-2.2065
19	H	-4.5149	-3.6366	-1.7597
20	N	-3.9054	0.3171	-1.4848
21	N	-3.2485	-2.0832	-1.0640
22	N	-4.0138	-0.6381	0.8577
23	H	-5.3649	-1.2944	-0.8417
24	N	0.4276	0.3961	0.3550
25	N	1.4798	0.2848	0.7972
26	C	2.6919	0.1726	1.2477
27	C	3.7760	0.2667	0.2506
28	O	3.5224	0.5755	-0.9285
29	C	6.1408	0.2010	-0.2846
30	H	5.8205	-0.2009	-1.2492
31	H	6.9905	-0.3947	0.0607
32	B	-4.2311	-0.9514	-0.6503
33	C	6.5492	1.6684	-0.4477
34	H	6.9246	2.0861	0.4918
35	H	5.6964	2.2678	-0.7804
36	H	7.3417	1.7528	-1.2008
37	C	-1.6105	0.2545	3.3537
38	C	-1.0561	1.5393	3.1647
39	C	-0.9175	-0.7151	4.1127
40	C	0.1948	1.8282	3.7283
41	C	0.3303	-0.3857	4.6560
42	C	0.9066	0.8786	4.4702
43	H	0.6284	2.8131	3.5679
44	H	0.8733	-1.1398	5.2225
45	C	0.1771	-3.0999	-1.3531
46	C	0.8657	-3.1085	-0.1181
47	C	0.9006	-3.0996	-2.5659
48	C	2.2659	-3.1198	-0.1222
49	C	2.3015	-3.1057	-2.5236
50	C	3.0031	-3.1150	-1.3124
51	H	2.7949	-3.1198	0.8287
52	H	2.8572	-3.0838	-3.4590
53	C	-1.3750	2.6762	-2.4165
54	C	-0.2427	2.0552	-2.9944
55	C	-1.2809	3.9857	-1.9010
56	C	0.9652	2.7606	-3.0411
57	C	-0.0507	4.6564	-1.9668
58	C	1.0844	4.0591	-2.5282
59	H	1.8396	2.2792	-3.4741
60	H	0.0244	5.6614	-1.5564
61	C	-1.7656	2.5851	2.3348
62	H	-2.8268	2.6567	2.5970
63	H	-1.7199	2.3350	1.2693
64	H	-1.3079	3.5694	2.4705
65	C	-1.4871	-2.1004	4.3161
66	H	-1.8204	-2.5398	3.3707
67	H	-2.3585	-2.0792	4.9813
68	H	-0.7426	-2.7674	4.7610
69	C	2.2595	1.2002	5.0607
70	H	2.9552	0.3620	4.9428
71	H	2.1820	1.4036	6.1368

72	H	2.7037	2.0821	4.5893
73	C	0.1902	-3.0640	-3.9005
74	H	-0.5878	-2.2942	-3.9187
75	H	-0.3010	-4.0194	-4.1199
76	H	0.8951	-2.8563	-4.7109
77	C	0.1267	-3.0812	1.2004
78	H	-0.7326	-3.7594	1.1982
79	H	-0.2627	-2.0806	1.4128
80	H	0.7883	-3.3648	2.0243
81	C	4.5131	-3.1294	-1.2808
82	H	4.8959	-2.5130	-0.4611
83	H	4.9356	-2.7536	-2.2178
84	H	4.8942	-4.1484	-1.1315
85	C	-2.4705	4.6500	-1.2445
86	H	-2.8717	4.0306	-0.4335
87	H	-3.2883	4.8104	-1.9559
88	H	-2.1933	5.6219	-0.8255
89	C	-0.3064	0.6418	-3.5286
90	H	-1.2143	0.4720	-4.1170
91	H	-0.3184	-0.0854	-2.7100
92	H	0.5620	0.4219	-4.1563
93	C	2.4103	4.7811	-2.5840
94	H	3.2067	4.1714	-2.1408
95	H	2.3724	5.7346	-2.0479
96	H	2.7040	4.9898	-3.6204
97	H	2.7889	0.0466	2.3169
98	N	5.0468	-0.0022	0.6780
99	C	5.3777	-0.3834	2.0577
100	H	6.2783	-1.0031	2.0117
101	H	4.5870	-1.0305	2.4476
102	C	5.6098	0.8046	2.9968
103	H	4.7350	1.4622	3.0240
104	H	5.8023	0.4462	4.0147
105	H	6.4721	1.3977	2.6767

DCM_metallocarbene6
Energy (POTENTIAL) = -2312.28934831 Eh

	Atom	X	Y	Z
1	Cu	0.5388	-0.4035	-0.3922
2	N	1.3843	0.0246	-2.2125
3	C	1.5199	1.1424	-2.9453
4	C	2.5078	-0.5119	-4.0510
5	C	2.2290	0.8468	-4.1292
6	H	3.0335	-1.1698	-4.7290
7	H	2.4973	1.5337	-4.9188
8	N	-0.2961	-2.1932	-1.3548
9	C	-1.4845	-2.8148	-1.4224
10	C	-1.3773	-3.9617	-2.2439
11	C	-0.0559	-3.9762	-2.6673
12	H	-2.1600	-4.6629	-2.4949
13	H	0.4765	-4.6590	-3.3150
14	N	2.2750	-1.6623	0.0291
15	C	3.0980	-1.8030	1.0776
16	C	4.1559	-2.6757	0.7361
17	C	3.9028	-3.0440	-0.5813
18	H	4.9766	-2.9910	1.3646
19	H	4.4413	-3.6983	-1.2532
20	N	1.9926	-0.9855	-2.8954
21	N	2.7689	-2.4217	-0.9800
22	N	0.5726	-2.9090	-2.1223
23	H	2.5914	-3.1561	-3.1114
24	C	-0.0551	0.5219	1.0756
25	C	-1.3849	0.6307	1.6870
26	O	-1.6454	-0.3816	2.3732
27	C	-3.5058	1.7030	2.1802
28	H	-3.9602	0.7198	2.0380
29	H	-4.1228	2.4334	1.6483
30	B	2.0288	-2.4340	-2.3349
31	C	-3.4400	2.0291	3.6752
32	H	-3.0292	3.0263	3.8567

33	H	-2.8187	1.2960	4.1983
34	H	-4.4480	1.9903	4.1045
35	C	-2.6935	-2.2694	-0.7584
36	C	-3.2080	-1.0144	-1.1525
37	C	-3.3473	-3.0170	0.2488
38	C	-4.3738	-0.5352	-0.5374
39	C	-4.5096	-2.5020	0.8335
40	C	-5.0421	-1.2636	0.4514
41	H	-4.7668	0.4332	-0.8407
42	H	-5.0044	-3.0729	1.6171
43	C	2.7906	-1.0772	2.3339
44	C	3.5383	0.0646	2.6919
45	C	1.6800	-1.4763	3.1095
46	C	3.1574	0.7962	3.8250
47	C	1.3383	-0.7215	4.2392
48	C	2.0605	0.4187	4.6120
49	H	3.7230	1.6868	4.0922
50	H	0.4729	-1.0190	4.8268
51	C	0.9833	2.4469	-2.4873
52	C	1.5928	3.1203	-1.4065
53	C	-0.1189	3.0243	-3.1564
54	C	1.1004	4.3775	-1.0281
55	C	-0.5833	4.2779	-2.7409
56	C	0.0209	4.9768	-1.6874
57	H	1.5728	4.8988	-0.1984
58	H	-1.4347	4.7217	-3.2538
59	C	-2.5299	-0.1722	-2.2086
60	H	-2.2151	-0.7730	-3.0678
61	H	-1.6263	0.3015	-1.8110
62	H	-3.1964	0.6190	-2.5643
63	C	-2.7975	-4.3453	0.7196
64	H	-1.7282	-4.2753	0.9482
65	H	-2.9099	-5.1241	-0.0432
66	H	-3.3183	-4.6825	1.6208
67	C	-6.2989	-0.7356	1.1025
68	H	-7.1684	-1.3523	0.8420
69	H	-6.5119	0.2915	0.7901
70	H	-6.2139	-0.7479	2.1956
71	C	0.8335	-2.6611	2.7059
72	H	1.4499	-3.5237	2.4302
73	H	0.2153	-2.4081	1.8382
74	H	0.1625	-2.9561	3.5178
75	C	4.7142	0.5195	1.8568
76	H	4.4619	0.5507	0.7921
77	H	5.5657	-0.1635	1.9613
78	H	5.0464	1.5171	2.1596
79	C	1.6772	1.2088	5.8417
80	H	1.9930	2.2543	5.7617
81	H	2.1516	0.7923	6.7402
82	H	0.5947	1.1887	6.0076
83	C	-0.7939	2.3140	-4.3082
84	H	-0.9301	1.2488	-4.1008
85	H	-0.1961	2.3864	-5.2249
86	H	-1.7747	2.7527	-4.5154
87	C	2.7548	2.5060	-0.6602
88	H	3.5532	2.1969	-1.3443
89	H	2.4403	1.6084	-0.1190
90	H	3.1755	3.2104	0.0632
91	C	-0.4956	6.3366	-1.2792
92	H	-1.5518	6.2888	-0.9872
93	H	-0.4260	7.0501	-2.1095
94	H	0.0700	6.7460	-0.4369
95	H	0.6889	0.8933	1.7960
96	N	-2.1948	1.6875	1.5168
97	C	-1.7271	2.8552	0.7633
98	H	-0.8566	2.5438	0.1737
99	C	-1.3574	4.0411	1.6558
100	H	-0.9640	4.8573	1.0445
101	H	-2.2288	4.4160	2.2013
102	H	-0.5903	3.7528	2.3828

103	H	-2.5084	3.1379	0.0485

DCM_metallocarbene7
Energy (POTENTIAL) = -2198.98507083 Eh

	Atom	X	Y	Z
1	Cu	8.5894	10.4363	8.5323
2	N	8.3058	8.2092	6.5840
3	N	7.2207	13.0550	9.6987
4	N	6.9697	12.1058	8.7647
5	N	9.7361	13.0634	9.4692
6	N	9.9660	12.0554	8.5801
7	N	8.6411	11.6920	11.2806
8	N	8.6452	10.4882	10.6467
9	C	7.8271	9.1383	7.3757
10	H	6.7294	9.1168	7.3237
11	C	9.7581	8.0083	6.4404
12	H	9.9980	8.0146	5.3711
13	H	10.2290	8.8749	6.8995
14	C	10.2438	6.7193	7.0984
15	H	9.7893	5.8329	6.6432
16	H	10.0106	6.7227	8.1674
17	H	11.3298	6.6399	6.9804
18	C	7.4266	7.3093	5.7896
19	H	6.7149	7.9403	5.2496
20	H	8.0511	6.8000	5.0516
21	C	6.6861	6.2956	6.6596
22	H	6.0577	5.6570	6.0288
23	H	6.0400	6.8052	7.3809
24	H	7.3850	5.6609	7.2117
25	C	6.1716	13.9081	9.7979
26	H	6.1927	14.7371	10.4923
27	C	5.1974	13.4956	8.8951
28	H	4.2344	13.9505	8.7097
29	C	5.7520	12.3547	8.2656
30	C	5.2167	11.4631	7.2059
31	C	4.1504	10.5812	7.4927
32	C	3.4861	10.5797	8.8513
33	H	4.2274	10.5242	9.6556
34	H	2.8058	9.7287	8.9528
35	H	2.9065	11.4953	9.0167
36	C	3.7354	9.6726	6.5114
37	H	2.9250	8.9824	6.7392
38	C	4.3484	9.6155	5.2518
39	C	3.8987	8.6018	4.2258
40	H	4.0143	7.5790	4.6057
41	H	4.4760	8.6848	3.2998
42	H	2.8389	8.7316	3.9749
43	C	5.3981	10.5016	4.9872
44	H	5.8936	10.4650	4.0192
45	C	5.8488	11.4225	5.9446
46	C	7.0372	12.3030	5.6336
47	H	6.8655	13.3415	5.9366
48	H	7.2677	12.2864	4.5640
49	H	7.9198	11.9555	6.1819
50	C	10.6876	14.0175	9.3462
51	H	10.6720	14.8960	9.9764
52	C	11.5703	13.6254	8.3488
53	H	12.4506	14.1461	8.0002
54	C	11.0784	12.3786	7.9013
55	C	11.6792	11.4551	6.9064
56	C	11.6459	11.7646	5.5302
57	C	10.9598	13.0165	5.0316
58	H	9.9565	13.1205	5.4581
59	H	10.8691	13.0052	3.9412
60	H	11.5196	13.9168	5.3118
61	C	12.2525	10.8867	4.6206
62	H	12.2140	11.1188	3.5580
63	C	12.9031	9.7203	5.0425
64	C	13.5818	8.8043	4.0510
65	H	14.6481	9.0485	3.9570

66	H	13.1370	8.8925	3.0543
67	H	13.5162	7.7570	4.3656
68	C	12.9191	9.4306	6.4126
69	H	13.4006	8.5180	6.7581
70	C	12.3086	10.2709	7.3520
71	C	12.2767	9.8719	8.8089
72	H	11.2590	9.5912	9.1042
73	H	12.5762	10.6961	9.4654
74	H	12.9352	9.0194	8.9988
75	C	8.6510	11.5078	12.6216
76	H	8.6452	12.3533	13.2957
77	C	8.6642	10.1405	12.8729
78	H	8.6751	9.6443	13.8329
79	C	8.6554	9.5391	11.5946
80	C	8.6357	8.1071	11.2058
81	C	9.7979	7.3178	11.3433
82	C	11.0717	7.9018	11.9115
83	H	10.9943	8.0502	12.9954
84	H	11.2948	8.8794	11.4724
85	H	11.9228	7.2397	11.7251
86	C	9.7625	5.9801	10.9299
87	H	10.6628	5.3750	11.0211
88	C	8.6049	5.4067	10.3862
89	C	8.6118	3.9759	9.9022
90	H	7.6015	3.6268	9.6674
91	H	9.0410	3.3007	10.6516
92	H	9.2197	3.8754	8.9936
93	C	7.4629	6.2065	10.2668
94	H	6.5565	5.7796	9.8437
95	C	7.4571	7.5506	10.6642
96	C	6.2049	8.3832	10.5070
97	H	6.3779	9.2274	9.8335
98	H	5.8856	8.8073	11.4666
99	H	5.3812	7.7848	10.1074
100	B	8.5575	13.0147	10.4737
101	H	8.6255	13.9512	11.2241

DCM_TSDCO
Energy (POTENTIAL) = -2312.27070328 Eh

	Atom	X	Y	Z
1	Cu	0.5302	-0.4579	-0.3572
2	N	1.3169	-0.0081	-2.2254
3	C	1.4359	1.1327	-2.9226
4	C	2.4931	-0.4638	-4.0506
5	C	2.1749	0.8893	-4.1007
6	H	3.0513	-1.0877	-4.7349
7	H	2.4395	1.6021	-4.8686
8	N	-0.3101	-2.3044	-1.4061
9	C	-1.4678	-2.9750	-1.5039
10	C	-1.3103	-4.0885	-2.3636
11	C	0.0134	-4.0341	-2.7761
12	H	-2.0617	-4.8132	-2.6429
13	H	0.5790	-4.6728	-3.4406
14	N	2.2077	-1.6460	0.0132
15	C	2.9986	-1.7747	1.0891
16	C	4.0678	-2.6443	0.7819
17	C	3.8559	-3.0189	-0.5417
18	H	4.8735	-2.9506	1.4334
19	H	4.4189	-3.6724	-1.1938
20	N	1.9692	-0.9812	-2.9163
21	N	2.7335	-2.4040	-0.9804
22	N	0.5924	-2.9594	-2.1899
23	H	2.6465	-3.1324	-3.1216
24	C	-0.0121	0.5607	1.0831
25	C	-1.2992	0.7010	1.7126
26	O	-2.0659	-0.1276	2.1859
27	C	-2.8718	2.2502	0.6200
28	H	-3.0275	1.3521	0.0190
29	H	-2.6812	3.0794	-0.0705
30	B	2.0342	-2.4313	-2.3634

31	C	-4.1198	2.5327	1.4613
32	H	-4.0149	3.4570	2.0385
33	H	-4.3193	1.7066	2.1496
34	H	-4.9842	2.6488	0.7968
35	C	-2.7014	-2.5097	-0.8239
36	C	-3.3029	-1.2934	-1.2127
37	C	-3.2934	-3.2996	0.1885
38	C	-4.4993	-0.8982	-0.5960
39	C	-4.4823	-2.8630	0.7848
40	C	-5.1045	-1.6661	0.4040
41	H	-4.9645	0.0365	-0.9020
42	H	-4.9296	-3.4660	1.5729
43	C	2.6612	-1.0062	2.3122
44	C	3.4252	0.1302	2.6584
45	C	1.5071	-1.3433	3.0536
46	C	3.0155	0.9173	3.7415
47	C	1.1351	-0.5290	4.1346
48	C	1.8732	0.6046	4.4927
49	H	3.5956	1.8012	4.0001
50	H	0.2402	-0.7828	4.6981
51	C	0.8410	2.3843	-2.3913
52	C	1.4211	3.0068	-1.2654
53	C	-0.3303	2.9159	-2.9745
54	C	0.8123	4.1534	-0.7366
55	C	-0.9108	4.0581	-2.4098
56	C	-0.3567	4.6893	-1.2875
57	H	1.2568	4.6311	0.1337
58	H	-1.8231	4.4585	-2.8485
59	C	-2.6705	-0.4024	-2.2570
60	H	-2.3590	-0.9694	-3.1408
61	H	-1.7698	0.0799	-1.8625
62	H	-3.3634	0.3818	-2.5752
63	C	-2.6497	-4.5885	0.6493
64	H	-1.5906	-4.4388	0.8877
65	H	-2.6945	-5.3638	-0.1239
66	H	-3.1498	-4.9754	1.5423
67	C	-6.3723	-1.2082	1.0864
68	H	-6.8125	-0.3445	0.5782
69	H	-6.1725	-0.9172	2.1256
70	H	-7.1232	-2.0066	1.1140
71	C	0.6703	-2.5487	2.6890
72	H	1.2884	-3.4495	2.6011
73	H	0.1752	-2.4051	1.7234
74	H	-0.1024	-2.7312	3.4408
75	C	4.6553	0.5139	1.8671
76	H	4.4581	0.5112	0.7905
77	H	5.4760	-0.1920	2.0411
78	H	5.0062	1.5109	2.1496
79	C	1.4640	1.4726	5.6598
80	H	1.4377	2.5315	5.3782
81	H	2.1781	1.3787	6.4879
82	H	0.4758	1.1959	6.0387
83	C	-0.9620	2.2611	-4.1821
84	H	-1.0638	1.1803	-4.0440
85	H	-0.3506	2.4102	-5.0801
86	H	-1.9542	2.6776	-4.3799
87	C	2.6688	2.4498	-0.6194
88	H	3.4729	2.3117	-1.3511
89	H	2.4773	1.4686	-0.1751
90	H	3.0338	3.1144	0.1691
91	C	-1.0245	5.9015	-0.6820
92	H	-2.0288	5.6548	-0.3155
93	H	-1.1421	6.7032	-1.4209
94	H	-0.4475	6.2992	0.1585
95	H	0.7541	1.1946	1.5434
96	N	-1.6365	2.0381	1.3882
97	C	-1.0559	3.1728	2.1353
98	H	0.0288	3.1823	2.0059
99	C	-1.3989	3.1541	3.6277
100	H	-0.9598	4.0315	4.1169

101	H	-2.4796	3.1696	3.7946
102	H	-0.9918	2.2593	4.1095
103	H	-1.4362	4.0796	1.6585

DCM_INTDCO1
Energy (POTENTIAL) = -2312.28247342 Eh

	Atom	X	Y	Z
1	Cu	0.2168	-0.5072	-0.2964
2	N	1.1058	0.0765	-2.1938
3	C	1.2248	1.1957	-2.9244
4	C	2.1965	-0.4448	-4.0625
5	C	1.9101	0.9131	-4.1285
6	H	2.7147	-1.0957	-4.7533
7	H	2.1615	1.6040	-4.9206
8	N	-0.5005	-2.2338	-1.2796
9	C	-1.6420	-2.9399	-1.2820
10	C	-1.4825	-4.1091	-2.0585
11	C	-0.1764	-4.0465	-2.5288
12	H	-2.2212	-4.8730	-2.2548
13	H	0.3818	-4.7170	-3.1676
14	N	2.1123	-1.5973	0.0625
15	C	3.0154	-1.6760	1.0504
16	C	4.1162	-2.4604	0.6327
17	C	3.8061	-2.8411	-0.6677
18	H	5.0007	-2.7105	1.2012
19	H	4.3499	-3.4457	-1.3807
20	N	1.7043	-0.9248	-2.8958
21	N	2.6007	-2.3096	-0.9845
22	N	0.3917	-2.9148	-2.0518
23	H	2.3849	-3.0689	-3.0997
24	C	-0.1406	0.6670	1.2800
25	C	-1.2289	0.4462	2.1047
26	O	-1.9662	-0.0825	2.8955
27	C	-2.1174	1.8781	0.0796
28	H	-2.1847	0.8740	-0.3359
29	H	-1.5526	2.4995	-0.6197
30	B	1.8131	-2.3604	-2.3152
31	C	-3.5096	2.4441	0.3333
32	H	-3.4833	3.4836	0.6736
33	H	-4.0547	1.8435	1.0679
34	H	-4.0694	2.4201	-0.6069
35	C	-2.8478	-2.4056	-0.6045
36	C	-3.5168	-1.3039	-1.1794
37	C	-3.3138	-2.9732	0.6004
38	C	-4.6430	-0.7806	-0.5302
39	C	-4.4397	-2.4166	1.2203
40	C	-5.1140	-1.3162	0.6741
41	H	-5.1555	0.0719	-0.9696
42	H	-4.7886	-2.8403	2.1601
43	C	2.7565	-0.9730	2.3306
44	C	3.5462	0.1424	2.6940
45	C	1.6711	-1.3666	3.1427
46	C	3.2316	0.8465	3.8623
47	C	1.3941	-0.6361	4.3076
48	C	2.1586	0.4731	4.6835
49	H	3.8287	1.7163	4.1307
50	H	0.5476	-0.9325	4.9228
51	C	0.7031	2.4853	-2.4077
52	C	1.3015	3.0725	-1.2712
53	C	-0.3992	3.1122	-3.0322
54	C	0.7769	4.2735	-0.7718
55	C	-0.8890	4.3128	-2.5032
56	C	-0.3181	4.9073	-1.3692
57	H	1.2351	4.7217	0.1073
58	H	-1.7484	4.7857	-2.9747
59	C	-3.0254	-0.6846	-2.4692
60	H	-3.0049	-1.4213	-3.2812
61	H	-2.0019	-0.3104	-2.3632
62	H	-3.6680	0.1453	-2.7771
63	C	-2.6001	-4.1488	1.2280

64	H	-1.5284	-3.9498	1.3372
65	H	-2.6953	-5.0509	0.6123
66	H	-3.0087	-4.3720	2.2181
67	C	-6.2927	-0.7032	1.3933
68	H	-6.8318	0.0015	0.7523
69	H	-5.9617	-0.1571	2.2861
70	H	-7.0002	-1.4696	1.7299
71	C	0.7777	-2.5229	2.7557
72	H	1.3586	-3.3972	2.4429
73	H	0.1384	-2.2512	1.9083
74	H	0.1306	-2.8128	3.5885
75	C	4.6989	0.6000	1.8282
76	H	4.4070	0.6697	0.7755
77	H	5.5401	-0.1012	1.8782
78	H	5.0619	1.5808	2.1501
79	C	1.8393	1.2610	5.9323
80	H	0.8639	0.9823	6.3429
81	H	1.8291	2.3379	5.7282
82	H	2.5915	1.0880	6.7126
83	C	-1.0746	2.4850	-4.2310
84	H	-0.4141	2.4689	-5.1049
85	H	-1.9794	3.0369	-4.5033
86	H	-1.3561	1.4457	-4.0271
87	C	2.4875	2.4287	-0.5893
88	H	3.3005	2.2404	-1.3001
89	H	2.2181	1.4598	-0.1606
90	H	2.8745	3.0660	0.2112
91	C	-0.8967	6.1793	-0.7951
92	H	-0.9341	6.9758	-1.5476
93	H	-0.3062	6.5417	0.0521
94	H	-1.9247	6.0211	-0.4452
95	H	0.8107	0.9967	1.6907
96	N	-1.2880	1.7586	1.3099
97	C	-1.1473	3.0332	2.0843
98	H	-1.0036	3.8246	1.3434
99	C	-0.0111	3.0507	3.0977
100	H	-0.0483	4.0064	3.6304
101	H	-0.1000	2.2517	3.8388
102	H	0.9681	2.9717	2.6183
103	H	-2.1039	3.2034	2.5868

DCM_TSDCO2
Energy (POTENTIAL) = -2312.28176016 Eh

	Atom	X	Y	Z
1	Cu	0.2231	-0.4825	-0.3146
2	N	1.0944	0.0956	-2.2266
3	C	1.1919	1.1997	-2.9837
4	C	2.1656	-0.4537	-4.1001
5	C	1.8643	0.8989	-4.1904
6	H	2.6803	-1.1144	-4.7842
7	H	2.0974	1.5764	-4.9995
8	N	-0.4874	-2.2215	-1.2711
9	C	-1.6243	-2.9350	-1.2589
10	C	-1.4623	-4.1098	-2.0264
11	C	-0.1601	-4.0428	-2.5065
12	H	-2.1969	-4.8808	-2.2101
13	H	0.3980	-4.7150	-3.1435
14	N	2.1259	-1.5614	0.0502
15	C	3.0310	-1.6358	1.0364
16	C	4.1367	-2.4116	0.6160
17	C	3.8262	-2.7942	-0.6839
18	H	5.0234	-2.6569	1.1833
19	H	4.3720	-3.3958	-1.3978
20	N	1.6942	-0.9139	-2.9173
21	N	2.6166	-2.2709	-0.9977
22	N	0.4040	-2.9035	-2.0429
23	H	2.3910	-3.0529	-3.1028
24	C	-0.1083	0.7121	1.2749
25	C	-1.1295	0.3574	2.1209
26	O	-1.8719	-0.2035	2.8656

27	C	-2.0089	2.0108	0.1027
28	H	-2.0010	1.0611	-0.4318
29	H	-1.4912	2.7484	-0.5184
30	B	1.8194	-2.3410	-2.3212
31	C	-3.4412	2.4381	0.4044
32	H	-3.4901	3.4088	0.9083
33	H	-3.9477	1.6947	1.0269
34	H	-3.9928	2.5283	-0.5371
35	C	-2.8250	-2.4085	-0.5675
36	C	-3.4913	-1.2896	-1.1137
37	C	-3.2905	-3.0033	0.6241
38	C	-4.6140	-0.7792	-0.4498
39	C	-4.4156	-2.4602	1.2580
40	C	-5.0868	-1.3447	0.7408
41	H	-5.1249	0.0852	-0.8673
42	H	-4.7658	-2.9079	2.1861
43	C	2.7633	-0.9533	2.3257
44	C	3.5282	0.1722	2.7028
45	C	1.6906	-1.3863	3.1373
46	C	3.2079	0.8463	3.8897
47	C	1.4091	-0.6885	4.3187
48	C	2.1552	0.4287	4.7139
49	H	3.7870	1.7244	4.1691
50	H	0.5740	-1.0147	4.9346
51	C	0.6667	2.5028	-2.5059
52	C	1.2887	3.1490	-1.4123
53	C	-0.4430	3.0983	-3.1421
54	C	0.7897	4.3842	-0.9816
55	C	-0.9127	4.3356	-2.6768
56	C	-0.3115	4.9941	-1.5979
57	H	1.2706	4.8824	-0.1422
58	H	-1.7757	4.7871	-3.1619
59	C	-3.0094	-0.6472	-2.3956
60	H	-2.9958	-1.3695	-3.2206
61	H	-1.9855	-0.2751	-2.2920
62	H	-3.6542	0.1882	-2.6830
63	C	-2.5795	-4.1947	1.2251
64	H	-1.5046	-4.0075	1.3233
65	H	-2.6912	-5.0873	0.5984
66	H	-2.9777	-4.4283	2.2170
67	C	-6.2601	-0.7413	1.4764
68	H	-6.8617	-0.1043	0.8201
69	H	-5.9155	-0.1210	2.3145
70	H	-6.9127	-1.5150	1.8957
71	C	0.8187	-2.5516	2.7283
72	H	1.4170	-3.4138	2.4138
73	H	0.1856	-2.2824	1.8757
74	H	0.1674	-2.8600	3.5510
75	C	4.6594	0.6748	1.8339
76	H	4.3523	0.7622	0.7866
77	H	5.5157	-0.0092	1.8573
78	H	5.0060	1.6557	2.1733
79	C	1.8300	1.1609	5.9948
80	H	2.1610	2.2038	5.9546
81	H	2.3275	0.6911	6.8535
82	H	0.7537	1.1507	6.1969
83	C	-1.1434	2.4139	-4.2945
84	H	-0.5228	2.4081	-5.1979
85	H	-2.0821	2.9214	-4.5369
86	H	-1.3700	1.3685	-4.0583
87	C	2.4724	2.5249	-0.7090
88	H	3.2981	2.3400	-1.4063
89	H	2.2059	1.5562	-0.2781
90	H	2.8413	3.1718	0.0925
91	C	-0.8615	6.2991	-1.0724
92	H	-1.4834	6.8035	-1.8187
93	H	-0.0578	6.9825	-0.7768
94	H	-1.4841	6.1286	-0.1839
95	H	0.8735	1.0113	1.6424
96	N	-1.1980	1.8035	1.3258

97	C	-0.8487	3.0404	2.0765
98	H	0.0256	3.4768	1.5783
99	C	-0.5685	2.8182	3.5593
100	H	-0.3023	3.7777	4.0144
101	H	-1.4485	2.4265	4.0788
102	H	0.2663	2.1321	3.7208
103	H	-1.6799	3.7385	1.9604

DCM_INTDCO2

Energy (POTENTIAL) = -2312.29968795 Eh

	Atom	X	Y	Z
1	Cu	0.2641	-0.4550	-0.2838
2	N	1.0867	0.0967	-2.1578
3	C	1.1597	1.1730	-2.9614
4	C	2.0645	-0.5317	-4.0585
5	C	1.7731	0.8191	-4.1828
6	H	2.5386	-1.2224	-4.7418
7	H	1.9756	1.4670	-5.0234
8	N	-0.5423	-2.1761	-1.1979
9	C	-1.7186	-2.8232	-1.2000
10	C	-1.6100	-4.0010	-1.9732
11	C	-0.3013	-4.0039	-2.4399
12	H	-2.3837	-4.7294	-2.1689
13	H	0.2254	-4.7010	-3.0768
14	N	2.1283	-1.5852	0.1166
15	C	3.0691	-1.6689	1.0717
16	C	4.1327	-2.4836	0.6285
17	C	3.7673	-2.8774	-0.6541
18	H	5.0316	-2.7411	1.1707
19	H	4.2730	-3.5021	-1.3777
20	N	1.6457	-0.9463	-2.8419
21	N	2.5637	-2.3276	-0.9363
22	N	0.3189	-2.9006	-1.9626
23	H	2.2929	-3.0993	-3.0365
24	C	-0.2690	0.7287	1.3761
25	C	-0.5509	-0.5674	1.6965
26	O	-0.9726	-1.5617	2.1618
27	C	-1.6570	2.3224	0.1498
28	H	-1.5118	1.6055	-0.6600
29	H	-0.8987	3.1082	0.0192
30	B	1.7462	-2.3784	-2.2471
31	C	-3.0578	2.9237	0.0423
32	H	-3.2025	3.7757	0.7147
33	H	-3.8188	2.1706	0.2694
34	H	-3.2196	3.2841	-0.9796
35	C	-2.9029	-2.2455	-0.5194
36	C	-3.4837	-1.0620	-1.0316
37	C	-3.4426	-2.8626	0.6267
38	C	-4.6006	-0.5219	-0.3851
39	C	-4.5539	-2.2800	1.2529
40	C	-5.1455	-1.1098	0.7644
41	H	-5.0526	0.3841	-0.7827
42	H	-4.9593	-2.7476	2.1479
43	C	2.8679	-0.9413	2.3498
44	C	3.4305	0.3418	2.5293
45	C	2.0574	-1.5096	3.3543
46	C	3.1087	1.0659	3.6837
47	C	1.7583	-0.7509	4.4952
48	C	2.2598	0.5439	4.6701
49	H	3.5219	2.0648	3.8131
50	H	1.1093	-1.1784	5.2562
51	C	0.6791	2.5149	-2.5491
52	C	1.3714	3.2321	-1.5497
53	C	-0.4369	3.0945	-3.1912
54	C	0.9460	4.5254	-1.2198
55	C	-0.8419	4.3829	-2.8178
56	C	-0.1612	5.1180	-1.8395
57	H	1.4842	5.0764	-0.4512
58	H	-1.7111	4.8227	-3.3033
59	C	-2.9218	-0.3854	-2.2615

60	H	-2.9208	-1.0622	-3.1246
61	H	-1.8836	-0.0799	-2.1063
62	H	-3.5049	0.5019	-2.5230
63	C	-2.8298	-4.1215	1.1952
64	H	-1.7439	-4.0238	1.2893
65	H	-3.0215	-4.9876	0.5507
66	H	-3.2409	-4.3433	2.1848
67	C	-6.3220	-0.4726	1.4661
68	H	-7.1099	-0.1974	0.7554
69	H	-6.0166	0.4471	1.9815
70	H	-6.7562	-1.1441	2.2136
71	C	1.5413	-2.9238	3.2234
72	H	2.3659	-3.6422	3.3151
73	H	1.0723	-3.0978	2.2517
74	H	0.8086	-3.1495	4.0030
75	C	4.3961	0.9206	1.5203
76	H	4.1280	0.6566	0.4952
77	H	5.4085	0.5340	1.6963
78	H	4.4413	2.0108	1.5965
79	C	1.9035	1.3669	5.8857
80	H	1.1760	0.8517	6.5200
81	H	1.4746	2.3332	5.5948
82	H	2.7914	1.5792	6.4942
83	C	-1.1944	2.3548	-4.2712
84	H	-0.6471	2.3716	-5.2215
85	H	-2.1733	2.8124	-4.4436
86	H	-1.3472	1.3033	-4.0123
87	C	2.5346	2.6109	-0.8170
88	H	3.2709	2.1846	-1.5072
89	H	2.1920	1.7891	-0.1803
90	H	3.0397	3.3455	-0.1838
91	C	-0.6420	6.4896	-1.4284
92	H	-1.0700	7.0361	-2.2755
93	H	0.1706	7.0903	-1.0068
94	H	-1.4249	6.4133	-0.6619
95	H	0.7287	1.1154	1.6074
96	N	-1.4362	1.5825	1.4060
97	C	-1.4083	2.4432	2.6035
98	H	-0.4940	3.0676	2.6036
99	C	-1.4982	1.6558	3.9098
100	H	-1.5507	2.3490	4.7568
101	H	-2.3968	1.0286	3.9214
102	H	-0.6271	1.0114	4.0615
103	H	-2.2613	3.1239	2.5403

DCM_TSDCO3

Energy (POTENTIAL) = -2312.27972372 Eh

	Atom	X	Y	Z
1	Cu	0.2177	-0.5066	-0.2553
2	N	1.0815	0.0727	-2.1669
3	C	1.1460	1.1676	-2.9425
4	C	2.1273	-0.4859	-4.0530
5	C	1.8023	0.8597	-4.1562
6	H	2.6386	-1.1485	-4.7377
7	H	2.0083	1.5298	-4.9786
8	N	-0.4925	-2.2335	-1.2215
9	C	-1.6585	-2.8974	-1.2760
10	C	-1.5158	-4.0489	-2.0826
11	C	-0.1970	-4.0181	-2.5169
12	H	-2.2748	-4.7804	-2.3204
13	H	0.3540	-4.6861	-3.1645
14	N	2.1420	-1.5994	0.1047
15	C	3.0656	-1.6640	1.0747
16	C	4.1719	-2.4278	0.6363
17	C	3.8436	-2.8137	-0.6585
18	H	5.0704	-2.6632	1.1889
19	H	4.3844	-3.4077	-1.3826
20	N	1.6856	-0.9367	-2.8558
21	N	2.6230	-2.3044	-0.9508
22	N	0.3970	-2.9227	-1.9896

23	H	2.3798	-3.0744	-3.0561
24	C	-0.0652	0.7853	1.2135
25	C	-1.0443	-0.2169	2.2979
26	O	-2.1004	-0.6950	2.2916
27	C	-1.9045	2.1104	0.2760
28	H	-2.0165	1.1673	-0.2610
29	H	-1.7173	2.8925	-0.4676
30	B	1.8156	-2.3667	-2.2656
31	C	-3.1773	2.4305	1.0643
32	H	-3.1084	3.4020	1.5641
33	H	-3.3878	1.6656	1.8170
34	H	-4.0261	2.4698	0.3735
35	C	-2.8755	-2.3525	-0.6262
36	C	-3.4603	-1.1742	-1.1436
37	C	-3.4516	-3.0029	0.4839
38	C	-4.6135	-0.6666	-0.5327
39	C	-4.6112	-2.4673	1.0592
40	C	-5.2043	-1.2979	0.5698
41	H	-5.0635	0.2417	-0.9283
42	H	-5.0489	-2.9642	1.9228
43	C	2.8081	-0.9834	2.3676
44	C	3.5020	0.2023	2.6956
45	C	1.8037	-1.4798	3.2255
46	C	3.1661	0.8803	3.8745
47	C	1.5063	-0.7768	4.4014
48	C	2.1704	0.4075	4.7405
49	H	3.6832	1.8075	4.1146
50	H	0.7208	-1.1524	5.0536
51	C	0.6070	2.4762	-2.4957
52	C	1.2236	3.1613	-1.4250
53	C	-0.4963	3.0530	-3.1630
54	C	0.7279	4.4149	-1.0452
55	C	-0.9641	4.3073	-2.7476
56	C	-0.3656	5.0051	-1.6908
57	H	1.2034	4.9393	-0.2195
58	H	-1.8200	4.7460	-3.2572
59	C	-2.8713	-0.4725	-2.3473
60	H	-2.8493	-1.1347	-3.2215
61	H	-1.8377	-0.1648	-2.1657
62	H	-3.4533	0.4162	-2.6078
63	C	-2.8146	-4.2399	1.0759
64	H	-1.7651	-4.0563	1.3353
65	H	-2.8248	-5.0792	0.3716
66	H	-3.3395	-4.5539	1.9831
67	C	-6.4199	-0.7026	1.2409
68	H	-7.0193	-1.4700	1.7419
69	H	-7.0611	-0.1811	0.5221
70	H	-6.1225	0.0298	2.0035
71	C	1.0297	-2.7306	2.8730
72	H	1.7015	-3.5794	2.6988
73	H	0.4578	-2.5929	1.9492
74	H	0.3339	-3.0002	3.6729
75	C	4.5714	0.7580	1.7826
76	H	4.2372	0.7858	0.7408
77	H	5.4771	0.1403	1.8087
78	H	4.8512	1.7730	2.0810
79	C	1.8286	1.1597	6.0056
80	H	0.8413	0.8770	6.3847
81	H	1.8337	2.2424	5.8387
82	H	2.5588	0.9505	6.7984
83	C	-1.1791	2.3419	-4.3102
84	H	-0.5868	2.4087	-5.2307
85	H	-2.1597	2.7836	-4.5134
86	H	-1.3184	1.2776	-4.0991
87	C	2.4003	2.5610	-0.6919
88	H	3.2087	2.2963	-1.3832
89	H	2.1127	1.6378	-0.1821
90	H	2.8005	3.2573	0.0505
91	C	-0.9203	6.3314	-1.2269
92	H	-1.3151	6.9163	-2.0644

93	H	-0.1573	6.9304	-0.7191
94	H	-1.7465	6.1822	-0.5185
95	H	0.8447	0.8829	1.8107
96	N	-0.6989	1.9766	1.1019
97	C	-0.3115	3.1113	1.9583
98	H	0.7748	3.2274	1.8791
99	C	-0.7061	2.9315	3.4266
100	H	-0.3596	3.7940	4.0071
101	H	-1.7902	2.8481	3.5479
102	H	-0.2411	2.0348	3.8490
103	H	-0.7653	4.0136	1.5409

DCM_CO_BS2
Energy (POTENTIAL) = -113.310181110 Eh

	Atom	X	Y	Z
1	C	2.3468	-0.0678	0.0000
2	O	3.4840	-0.0678	0.0000

DCM_TS4
Energy (POTENTIAL) = -2312.26852936 Eh

	Atom	X	Y	Z
1	N	1.0885	0.6745	-2.2404
2	C	1.1125	1.9269	-2.7253
3	C	2.0551	0.6100	-4.2455
4	C	1.7157	1.9345	-4.0048
5	H	2.5442	0.1433	-5.0894
6	H	1.8820	2.7874	-4.6470
7	N	-0.3422	-1.8711	-1.7369
8	C	-1.4670	-2.5933	-1.8460
9	C	-1.3279	-3.5434	-2.8838
10	C	-0.0539	-3.3286	-3.3937
11	H	-2.0615	-4.2641	-3.2161
12	H	0.4787	-3.8092	-4.2027
13	N	2.3307	-1.3484	-0.4469
14	C	3.3219	-1.5586	0.4333
15	C	4.4241	-2.1554	-0.2202
16	C	4.0229	-2.2887	-1.5438
17	H	5.3664	-2.4508	0.2187
18	H	4.5348	-2.6946	-2.4055
19	N	1.6678	-0.1254	-3.1770
20	N	2.7660	-1.7988	-1.6536
21	N	0.5173	-2.3221	-2.6917
22	H	2.4381	-2.1351	-3.8604
23	C	-0.1753	0.3925	1.2904
24	C	-1.2107	-0.2040	2.1914
25	O	-1.1763	-1.2853	2.7767
26	C	-3.5429	0.5847	2.7994
27	H	-3.6505	-0.4740	3.0468
28	H	-4.2859	0.8158	2.0296
29	B	1.8864	-1.6389	-2.9152
30	C	-3.7448	1.4621	4.0352
31	H	-3.6200	2.5235	3.7922
32	H	-3.0260	1.2013	4.8196
33	H	-4.7564	1.3234	4.4329
34	C	-2.6571	-2.2799	-1.0174
35	C	-3.3671	-1.0846	-1.2637
36	C	-3.0847	-3.1655	-0.0043
37	C	-4.5101	-0.8051	-0.5005
38	C	-4.2286	-2.8470	0.7368
39	C	-4.9603	-1.6756	0.4982
40	H	-5.0584	0.1150	-0.6914
41	H	-4.5506	-3.5229	1.5268
42	C	3.1435	-1.1972	1.8599
43	C	2.2104	-1.9123	2.6432
44	C	3.8754	-0.1285	2.4215
45	C	2.0205	-1.5369	3.9791
46	C	3.6508	0.2177	3.7610
47	C	2.7285	-0.4756	4.5565
48	H	1.2901	-2.0769	4.5767
49	H	4.2050	1.0499	4.1911

50	C	0.5952	3.0737	-1.9389
51	C	1.2154	3.4273	-0.7168
52	C	-0.4860	3.8397	-2.4288
53	C	0.7310	4.5326	-0.0044
54	C	-0.9259	4.9491	-1.6929
55	C	-0.3345	5.3102	-0.4764
56	H	1.2094	4.8030	0.9350
57	H	-1.7623	5.5332	-2.0714
58	C	-2.9051	-0.1059	-2.3202
59	H	-2.7550	-0.5993	-3.2873
60	H	-1.9430	0.3406	-2.0476
61	H	-3.6332	0.6991	-2.4566
62	C	-2.3025	-4.4206	0.3081
63	H	-2.7285	-4.9413	1.1707
64	H	-1.2571	-4.1831	0.5364
65	H	-2.2951	-5.1162	-0.5385
66	C	-6.1918	-1.3635	1.3153
67	H	-6.6120	-0.3882	1.0513
68	H	-5.9626	-1.3553	2.3874
69	H	-6.9723	-2.1186	1.1598
70	C	4.8861	0.6439	1.6029
71	H	4.4956	0.8977	0.6130
72	H	5.7987	0.0568	1.4430
73	H	5.1726	1.5710	2.1088
74	C	1.4051	-3.0460	2.0532
75	H	2.0312	-3.7145	1.4521
76	H	0.6187	-2.6547	1.4007
77	H	0.9219	-3.6325	2.8394
78	C	2.5221	-0.1089	6.0080
79	H	3.1615	-0.7181	6.6607
80	H	1.4858	-0.2798	6.3191
81	H	2.7689	0.9413	6.1967
82	C	-1.1858	3.4763	-3.7197
83	H	-1.4186	2.4065	-3.7609
84	H	-0.5633	3.7002	-4.5933
85	H	-2.1203	4.0358	-3.8233
86	C	2.4083	2.6612	-0.1912
87	H	3.2117	2.6289	-0.9365
88	H	2.1546	1.6222	0.0341
89	H	2.8026	3.1247	0.7176
90	C	-0.8631	6.4719	0.3311
91	H	-1.6151	6.1315	1.0560
92	H	-1.3419	7.2212	-0.3078
93	H	-0.0647	6.9626	0.8978
94	H	0.6677	0.8227	1.8411
95	Cu	0.4097	-0.3584	-0.4234
96	N	-2.2120	0.7251	2.2219
97	C	-1.7025	1.8763	1.5505
98	H	-0.7566	1.2695	0.6916
99	H	-1.1262	2.5713	2.1629
100	C	-2.5673	2.5010	0.4944
101	H	-3.3916	3.0343	0.9883
102	H	-2.0025	3.2288	-0.0895
103	H	-2.9929	1.7430	-0.1675

DCM_TS5
Energy (POTENTIAL) = -2312.27130014 Eh

	Atom	X	Y	Z
1	Cu	4.0933	3.4200	2.7224
2	N	4.7206	5.3938	2.0840
3	C	4.1460	6.5919	1.8947
4	C	6.2417	6.7419	1.1769
5	C	5.0754	7.4839	1.3120
6	H	7.2156	7.0113	0.7917
7	H	4.9149	8.5191	1.0469
8	N	5.1973	2.6540	1.0259
9	C	5.0173	1.7408	0.0576
10	C	6.1954	1.6258	-0.7179
11	C	7.0813	2.5349	-0.1584
12	H	6.3588	0.9817	-1.5699

13	H	8.0942	2.8000	-0.4285
14	N	5.9464	3.3907	3.8774
15	C	6.2834	3.1921	5.1615
16	C	7.6400	3.5375	5.3604
17	C	8.0868	3.9546	4.1129
18	H	8.2038	3.4810	6.2806
19	H	9.0548	4.3084	3.7854
20	N	6.0044	5.4948	1.6449
21	N	7.0544	3.8563	3.2422
22	N	6.4630	3.1375	0.8839
23	H	8.0740	4.6320	1.3864
24	C	2.4439	2.8003	3.5683
25	C	1.7669	1.5097	3.2271
26	O	2.3344	0.4453	3.5184
27	C	-0.1671	0.3639	2.2185
28	B	6.9829	4.2970	1.7628
29	C	3.7190	1.0582	-0.1639
30	C	2.5802	1.8114	-0.5273
31	C	3.6363	-0.3516	-0.0853
32	C	1.3883	1.1381	-0.8324
33	C	2.4257	-0.9817	-0.3945
34	C	1.2926	-0.2560	-0.7839
35	H	0.5148	1.7209	-1.1173
36	H	2.3636	-2.0662	-0.3258
37	C	5.2805	2.6722	6.1215
38	C	4.7654	1.3677	5.9498
39	C	4.8164	3.4872	7.1780
40	C	3.7749	0.9098	6.8287
41	C	3.8203	2.9916	8.0304
42	C	3.2846	1.7066	7.8709
43	H	3.3720	-0.0910	6.6899
44	H	3.4505	3.6262	8.8337
45	C	2.7562	6.8665	2.3365
46	C	2.4552	6.8887	3.7179
47	C	1.7466	7.1428	1.3882
48	C	1.1571	7.2241	4.1262
49	C	0.4560	7.4546	1.8389
50	C	0.1448	7.5183	3.2038
51	H	0.9291	7.2441	5.1899
52	H	-0.3236	7.6546	1.1063
53	C	2.6190	3.3210	-0.6010
54	H	3.4809	3.6754	-1.1770
55	H	2.7153	3.7611	0.3967
56	H	1.7094	3.7133	-1.0651
57	C	4.8267	-1.1845	0.3354
58	H	5.2992	-0.7776	1.2356
59	H	5.5967	-1.2117	-0.4440
60	H	4.5246	-2.2153	0.5439
61	C	0.0039	-0.9702	-1.1161
62	H	0.1428	-1.6727	-1.9470
63	H	-0.7846	-0.2662	-1.3997
64	H	-0.3555	-1.5538	-0.2598
65	C	5.3726	4.8753	7.4049
66	H	5.4884	5.4268	6.4678
67	H	6.3647	4.8310	7.8708
68	H	4.7211	5.4536	8.0673
69	C	5.2528	0.4781	4.8308
70	H	6.3437	0.5129	4.7370
71	H	4.8246	0.7966	3.8761
72	H	4.9486	-0.5588	4.9992
73	C	2.2292	1.1823	8.8170
74	H	2.6882	0.6742	9.6755
75	H	1.5742	0.4571	8.3226
76	H	1.6076	1.9925	9.2130
77	C	2.0366	7.1106	-0.0957
78	H	2.6701	6.2605	-0.3663
79	H	2.5664	8.0163	-0.4147
80	H	1.1091	7.0484	-0.6731
81	C	3.5002	6.5352	4.7504
82	H	4.4321	7.0877	4.5880

83	H	3.7565	5.4720	4.6935
84	H	3.1425	6.7491	5.7614
85	C	-1.2420	7.9029	3.6629
86	H	-2.0101	7.5004	2.9933
87	H	-1.3621	8.9940	3.6743
88	H	-1.4437	7.5399	4.6759
89	H	2.4663	2.9329	4.6529
90	N	0.5487	1.5709	2.6254
91	C	-0.0000	2.8828	2.3186
92	C	0.6102	3.8741	3.2790
93	H	1.8622	3.8341	3.1163
94	H	0.2891	3.7882	4.3134
95	H	0.5892	4.9087	2.9325
96	H	-1.0888	2.8700	2.4409
97	C	-1.2059	-0.0702	3.2546
98	H	-1.9403	0.7229	3.4371
99	H	-1.7448	-0.9567	2.9010
100	H	-0.7228	-0.3180	4.2056
101	H	0.5741	-0.4213	2.0595
102	H	0.2220	3.1625	1.2797
103	H	-0.6433	0.5669	1.2550

DCM_pyrrolidinone3
Energy (POTENTIAL) = -365.312604545 Eh

	Atom	X	Y	Z
1	C	-4.4025	0.6992	0.6796
2	C	-2.6070	-0.7855	0.0357
3	H	-2.2167	-1.3656	-0.8042
4	H	-2.1798	-1.2027	0.9571
5	N	-3.2205	1.4578	0.2614
6	C	-2.1611	0.6688	-0.0607
7	C	-4.1413	-0.7032	0.0963
8	H	-4.5965	-1.4918	0.7003
9	H	-4.5579	-0.7628	-0.9146
10	H	-5.3106	1.1682	0.2871
11	C	-3.1380	2.9052	0.4033
12	H	-2.2586	3.2320	-0.1578
13	H	-4.0247	3.3437	-0.0704
14	C	-3.0385	3.3584	1.8634
15	H	-2.1461	2.9384	2.3406
16	H	-2.9723	4.4511	1.9121
17	H	-3.9173	3.0480	2.4390
18	O	-1.0279	1.0686	-0.3604
19	H	-4.4749	0.6780	1.7759

DCM_azetidinone4
Energy (POTENTIAL) = -365.288824689 Eh

	Atom	X	Y	Z
1	C	-3.8005	0.2334	0.7075
2	C	-2.5857	-0.7031	0.9916
3	H	-2.4648	-1.5266	0.2818
4	H	-2.4970	-1.0726	2.0166
5	N	-2.8065	1.2967	0.4261
6	C	-1.7176	0.5242	0.6816
7	C	-4.7026	-0.1209	-0.4634
8	H	-5.3183	-0.9927	-0.2143
9	H	-5.3762	0.7084	-0.7074
10	H	-4.1083	-0.3577	-1.3536
11	H	-4.3895	0.4598	1.6041
12	C	-2.9621	2.6925	0.0636
13	H	-1.9646	3.0673	-0.1859
14	H	-3.5749	2.7531	-0.8447
15	C	-3.5906	3.5233	1.1855
16	H	-2.9690	3.4937	2.0870
17	H	-3.6927	4.5674	0.8691
18	H	-4.5877	3.1485	1.4420
19	O	-0.5134	0.7702	0.6612

DCM_INTHOMO
Energy (POTENTIAL) = -2787.06035680 Eh

	Atom	X	Y	Z
1	Cu	0.3440	-0.1453	-0.9206
2	N	1.4211	-1.0693	-2.4103
3	C	2.6990	-1.4684	-2.5315
4	C	1.6905	-1.8398	-4.4761
5	C	2.9183	-1.9570	-3.8373
6	H	1.3821	-2.1021	-5.4786
7	H	3.8389	-2.3562	-4.2377
8	N	-0.5090	1.1684	-2.4924
9	C	-0.7888	2.4715	-2.6758
10	C	-1.3838	2.6591	-3.9459
11	C	-1.4351	1.3935	-4.5084
12	H	-1.7110	3.5909	-4.3831
13	H	-1.7972	1.0550	-5.4693
14	N	-1.3473	-1.4423	-1.4277
15	C	-2.2240	-2.1674	-0.7192
16	C	-2.9772	-2.9858	-1.5928
17	C	-2.4974	-2.6863	-2.8636
18	H	-3.7649	-3.6779	-1.3313
19	H	-2.7846	-3.0576	-3.8378
20	N	0.8079	-1.3087	-3.6024
21	N	-1.5203	-1.7580	-2.7351
22	N	-0.9102	0.5178	-3.6217
23	H	-1.0362	-1.3379	-4.9044
24	C	0.6213	0.1131	0.8778
25	C	1.3723	1.1525	1.5911
26	O	2.5795	0.8271	1.6798
27	C	1.7192	3.3320	2.6015
28	H	1.1913	4.2820	2.4875
29	H	2.6156	3.3781	1.9759
30	B	-0.6966	-1.0033	-3.8027
31	C	2.1148	3.1003	4.0612
32	H	2.6655	2.1621	4.1697
33	H	1.2386	3.0623	4.7144
34	H	2.7613	3.9192	4.3981
35	C	-0.4557	3.5236	-1.6860
36	C	0.8831	3.7336	-1.2854
37	C	-1.4789	4.3761	-1.2013
38	C	1.1745	4.8018	-0.4244
39	C	-1.1399	5.4312	-0.3468
40	C	0.1835	5.6643	0.0520
41	H	2.2059	4.9582	-0.1174
42	H	-1.9299	6.0771	0.0320
43	C	-2.3161	-1.9596	0.7464
44	C	-1.9006	-2.9611	1.6487
45	C	-2.7766	-0.7135	1.2295
46	C	-1.9424	-2.6968	3.0242
47	C	-2.8080	-0.4929	2.6126
48	C	-2.3929	-1.4689	3.5271
49	H	-1.6037	-3.4614	3.7181
50	H	-3.1596	0.4663	2.9841
51	C	3.6269	-1.4818	-1.3759
52	C	3.4056	-2.4131	-0.3387
53	C	4.7443	-0.6205	-1.3327
54	C	4.3131	-2.4699	0.7273
55	C	5.6232	-0.7052	-0.2457
56	C	5.4264	-1.6243	0.7937
57	H	4.1455	-3.1913	1.5225
58	H	6.4786	-0.0336	-0.2060
59	C	2.0123	2.8444	-1.7542
60	H	1.9153	2.5826	-2.8121
61	H	2.0261	1.9003	-1.1986
62	H	2.9783	3.3347	-1.6030
63	C	-2.9319	4.1586	-1.5646
64	H	-3.2015	3.0980	-1.5273
65	H	-3.1542	4.5088	-2.5788
66	H	-3.5857	4.7035	-0.8767
67	C	0.5126	6.7921	1.0010
68	H	0.0211	6.6458	1.9712
69	H	0.1657	7.7561	0.6098

70	H	1.5899	6.8666	1.1781
71	C	-3.2258	0.3761	0.2809
72	H	-3.9721	0.0034	-0.4302
73	H	-2.3883	0.7516	-0.3149
74	H	-3.6648	1.2147	0.8289
75	C	-1.3920	-4.2950	1.1508
76	H	-0.5984	-4.1687	0.4064
77	H	-2.1892	-4.8719	0.6679
78	H	-0.9973	-4.8938	1.9757
79	C	-2.4482	-1.2193	5.0157
80	H	-1.6206	-1.7167	5.5308
81	H	-3.3833	-1.6084	5.4404
82	H	-2.4071	-0.1488	5.2425
83	C	4.9886	0.3897	-2.4305
84	H	4.0964	0.9971	-2.6162
85	H	5.2440	-0.0997	-3.3776
86	H	5.8106	1.0621	-2.1674
87	C	2.1994	-3.3240	-0.3469
88	H	2.0010	-3.7336	-1.3425
89	H	1.2997	-2.7730	-0.0501
90	H	2.3365	-4.1582	0.3458
91	C	6.3643	-1.6651	1.9770
92	H	6.1062	-0.8812	2.7012
93	H	7.4032	-1.4962	1.6731
94	H	6.3104	-2.6257	2.4996
95	H	0.3059	-0.6676	1.5856
96	N	0.8411	2.2916	2.0472
97	C	-0.5996	2.5328	1.9224
98	H	-0.7509	3.3935	1.2668
99	C	-1.2953	2.7534	3.2643
100	H	-2.3669	2.9053	3.0977
101	H	-1.1621	1.8921	3.9247
102	H	-0.9083	3.6439	3.7701
103	H	-1.0304	1.6644	1.4140
104	N	1.1656	-1.0201	4.2773
105	N	0.8297	0.0201	4.5942
106	C	1.5041	-2.2375	3.9545
107	C	0.9956	-3.2927	4.8517
108	O	0.4399	-2.9888	5.9270
109	N	1.1576	-4.5857	4.4566
110	C	1.7188	-4.9941	3.1638
111	H	1.1889	-5.9007	2.8543
112	H	1.4812	-4.2391	2.4132
113	C	0.7279	-5.6566	5.3668
114	H	0.8876	-5.3201	6.3934
115	H	1.3836	-6.5151	5.1903
116	C	-0.7364	-6.0510	5.1598
117	H	-1.0015	-6.8798	5.8266
118	H	-1.3971	-5.2070	5.3799
119	H	-0.9166	-6.3742	4.1284
120	C	3.2243	-5.2598	3.2284
121	H	3.4470	-6.0528	3.9506
122	H	3.5991	-5.5774	2.2491
123	H	3.7674	-4.3594	3.5338
124	H	2.1309	-2.3386	3.0798

DCM_TSHOMO
Energy (POTENTIAL) = -2787.05254444 Eh

	Atom	X	Y	Z
1	Cu	1.2062	0.4889	0.6792
2	N	2.1235	2.3584	0.8200
3	C	1.7678	3.6428	0.6125
4	C	3.9020	3.6348	1.2299
5	C	2.8772	4.4851	0.8477
6	H	4.9199	3.8387	1.5313
7	H	2.9044	5.5618	0.7663
8	N	3.0976	-0.5512	0.2562
9	C	3.4771	-1.6606	-0.4028
10	C	4.8694	-1.8527	-0.2531
11	C	5.2932	-0.7962	0.5408

12	H	5.4671	-2.6500	-0.6710
13	H	6.2720	-0.5375	0.9206
14	N	1.9546	0.2544	2.8445
15	C	1.4590	-0.0475	4.0548
16	C	2.4426	0.1768	5.0484
17	C	3.5543	0.6292	4.3517
18	H	2.3457	0.0217	6.1133
19	H	4.5380	0.9226	4.6915
20	N	3.4332	2.3670	1.2056
21	N	3.2368	0.6668	3.0358
22	N	4.2148	-0.0324	0.8314
23	H	5.2047	1.4257	2.2277
24	C	-0.3358	-0.1554	-0.1198
25	C	-1.6393	-0.4192	0.5600
26	O	-2.2170	0.5284	1.1300
27	C	-3.4805	-1.9131	1.0977
28	H	-3.6725	-1.1027	1.8004
29	H	-3.4710	-2.8435	1.6687
30	B	4.1077	1.1249	1.8415
31	C	-4.5640	-1.9528	0.0172
32	H	-4.3978	-2.7731	-0.6896
33	H	-4.5793	-1.0153	-0.5436
34	H	-5.5481	-2.0971	0.4781
35	C	2.5085	-2.5054	-1.1441
36	C	1.8998	-2.0142	-2.3208
37	C	2.2202	-3.8110	-0.6917
38	C	0.9857	-2.8228	-3.0054
39	C	1.3122	-4.5949	-1.4172
40	C	0.6738	-4.1158	-2.5662
41	H	0.5038	-2.4328	-3.8993
42	H	1.0833	-5.5973	-1.0618
43	C	0.0844	-0.5751	4.2246
44	C	-0.8569	0.1281	5.0111
45	C	-0.2712	-1.8080	3.6339
46	C	-2.1397	-0.4076	5.1797
47	C	-1.5584	-2.3169	3.8469
48	C	-2.5105	-1.6310	4.6079
49	H	-2.8671	0.1444	5.7724
50	H	-1.8251	-3.2706	3.3994
51	C	0.3840	4.0759	0.3009
52	C	-0.6653	3.7976	1.2094
53	C	0.1261	4.8528	-0.8545
54	C	-1.9525	4.2807	0.9284
55	C	-1.1765	5.3040	-1.0985
56	C	-2.2296	5.0382	-0.2145
57	H	-2.7557	4.0602	1.6278
58	H	-1.3714	5.8831	-1.9992
59	C	2.2564	-0.6496	-2.8544
60	H	3.3002	-0.6247	-3.1934
61	H	2.1585	0.1154	-2.0825
62	H	1.6178	-0.3751	-3.6981
63	C	2.8697	-4.3818	0.5494
64	H	2.9189	-3.6462	1.3564
65	H	3.8987	-4.7032	0.3472
66	H	2.3156	-5.2534	0.9111
67	C	-0.3695	-4.9417	-3.2792
68	H	-1.3779	-4.6478	-2.9576
69	H	-0.2543	-6.0093	-3.0662
70	H	-0.3230	-4.7989	-4.3643
71	C	0.7062	-2.5885	2.7860
72	H	1.6980	-2.6271	3.2477
73	H	0.8376	-2.1216	1.8046
74	H	0.3572	-3.6129	2.6309
75	C	-0.5065	1.4346	5.6879
76	H	0.1287	2.0627	5.0590
77	H	0.0410	1.2609	6.6228
78	H	-1.4118	1.9971	5.9363
79	C	-3.9094	-2.1737	4.7828
80	H	-4.2767	-2.0144	5.8027
81	H	-3.9538	-3.2463	4.5677

82	H	-4.6110	-1.6720	4.1039
83	C	1.2209	5.2246	-1.8294
84	H	1.9767	4.4411	-1.9250
85	H	1.7424	6.1323	-1.5013
86	H	0.8038	5.4246	-2.8211
87	C	-0.4468	3.0052	2.4747
88	H	0.5427	3.1828	2.9029
89	H	-0.5368	1.9331	2.2758
90	H	-1.2038	3.2623	3.2214
91	C	-3.6131	5.5846	-0.4767
92	H	-4.3759	5.0293	0.0767
93	H	-3.8638	5.5461	-1.5424
94	H	-3.6806	6.6356	-0.1671
95	H	-0.3192	-0.5561	-1.1382
96	N	-2.1451	-1.6844	0.5365
97	C	-1.5110	-2.7455	-0.2712
98	H	-1.8431	-2.6610	-1.3141
99	C	-1.7889	-4.1554	0.2455
100	H	-1.2066	-4.8634	-0.3491
101	H	-1.4871	-4.2634	1.2913
102	H	-2.8434	-4.4328	0.1556
103	H	-0.4310	-2.5858	-0.2602
104	N	-0.1674	1.9789	-2.1207
105	N	0.8526	2.1709	-2.5745
106	C	-1.3405	1.6855	-1.6091
107	C	-2.2141	0.8498	-2.4756
108	O	-1.7045	0.0054	-3.2354
109	N	-3.5572	1.0266	-2.3647
110	C	-4.1987	2.2026	-1.7523
111	H	-3.4571	2.9946	-1.6470
112	H	-4.9433	2.5718	-2.4672
113	C	-4.4291	0.1255	-3.1347
114	H	-5.3969	0.1013	-2.6258
115	H	-4.0040	-0.8796	-3.0891
116	C	-4.6004	0.5654	-4.5909
117	H	-5.2835	-0.1162	-5.1107
118	H	-5.0186	1.5763	-4.6533
119	H	-3.6375	0.5530	-5.1097
120	C	-4.8584	1.9052	-0.4065
121	H	-4.1199	1.5403	0.3116
122	H	-5.3191	2.8180	-0.0129
123	H	-5.6470	1.1520	-0.5090
124	H	-1.6366	2.2695	-0.7492

DCM_olefin5
Energy (POTENTIAL) = -730.596966270 Eh

	Atom	X	Y	Z
1	C	0.6625	-0.2927	2.7815
2	C	1.4589	0.9590	2.5885
3	O	2.6399	1.0200	2.9808
4	C	1.6245	3.2248	1.6970
5	C	1.3492	4.3474	2.7014
6	H	1.5651	4.0172	3.7231
7	H	0.3074	4.6805	2.6573
8	H	1.9886	5.2090	2.4764
9	H	-0.3069	-0.4078	2.3113
10	N	0.8476	2.0027	1.9559
11	C	-0.5862	2.0336	1.6061
12	H	-0.7134	2.8702	0.9145
13	C	-1.5224	2.2113	2.8054
14	H	-2.5604	2.2635	2.4567
15	H	-1.4439	1.3733	3.5055
16	H	-1.2958	3.1317	3.3519
17	H	-0.8495	1.1317	1.0455
18	C	1.1340	-1.2932	3.5386
19	C	0.3291	-2.5374	3.7461
20	O	-0.8684	-2.5837	3.4040
21	N	0.9475	-3.5946	4.3412
22	C	2.3824	-3.6594	4.6663
23	H	2.9535	-3.0815	3.9375

24	C	0.1567	-4.7949	4.6484
25	H	-0.8637	-4.4820	4.8793
26	H	0.5850	-5.2428	5.5506
27	C	0.1550	-5.8054	3.4987
28	H	-0.4152	-6.6980	3.7806
29	H	-0.3042	-5.3709	2.6051
30	H	1.1747	-6.1187	3.2474
31	C	2.6876	-3.1975	6.0921
32	H	2.1246	-3.7878	6.8235
33	H	3.7558	-3.3146	6.3074
34	H	2.4219	-2.1435	6.2267
35	H	2.0980	-1.1761	4.0198
36	H	2.6805	2.9539	1.7197
37	H	1.3821	3.5596	0.6821
38	H	2.6894	-4.7015	4.5331

DCM_TSCC_BS2
Energy (POTENTIAL) = -4508.07407266 Eh

	Atom	X	Y	Z
1	Cu	6.7860	0.2516	-3.9586
2	N	5.0295	0.3943	-2.7712
3	C	3.8328	0.9561	-3.0440
4	C	3.5691	-0.3188	-1.2380
5	C	2.8753	0.5261	-2.0930
6	H	3.2442	-0.8582	-0.3613
7	H	1.8344	0.8047	-2.0490
8	N	6.6424	1.3886	-0.2811
9	C	7.3855	2.0134	0.6577
10	C	8.0181	1.0527	1.4945
11	C	7.5944	-0.1782	1.0066
12	H	8.6650	1.2392	2.3373
13	H	7.8114	-1.1815	1.3428
14	N	7.2092	-1.6173	-3.0966
15	C	7.9009	-2.6762	-3.5772
16	C	8.1130	-3.6148	-2.5392
17	C	7.4812	-3.0718	-1.4274
18	H	8.6200	-4.5635	-2.6167
19	H	7.3570	-3.4633	-0.4293
20	N	4.8596	-0.3889	-1.6679
21	N	6.9411	-1.8751	-1.7861
22	N	6.7750	0.0582	-0.0610
23	H	5.5290	-1.8021	-0.0787
24	C	7.2666	1.7223	-5.2486
25	C	6.8148	0.4957	-7.2981
26	H	7.3727	-0.2546	-6.7351
27	H	7.3288	0.6537	-8.2491
28	B	6.0073	-1.0259	-0.8714
29	C	5.3790	0.0160	-7.5200
30	H	4.8014	0.7137	-8.1349
31	H	4.8634	-0.1018	-6.5634
32	H	5.3943	-0.9541	-8.0283
33	C	7.4380	3.4951	0.7114
34	C	6.2320	4.2491	0.7207
35	C	8.6803	4.1771	0.7150
36	C	6.2963	5.6496	0.6793
37	C	8.6981	5.5817	0.6800
38	C	7.5199	6.3373	0.6459
39	H	5.3694	6.2183	0.6794
40	H	9.6571	6.0936	0.6631
41	C	8.1771	-2.8671	-5.0218
42	C	7.0666	-2.9841	-5.9078
43	C	9.4871	-3.0755	-5.5127
44	C	7.2925	-3.3216	-7.2474
45	C	9.6664	-3.4089	-6.8687
46	C	8.5864	-3.5605	-7.7443
47	H	6.4400	-3.4390	-7.9118
48	H	10.6759	-3.5837	-7.2331
49	C	3.6104	1.9242	-4.1461
50	C	2.7788	1.5667	-5.2368
51	C	4.1441	3.2337	-4.0658

52	C	2.4681	2.5304	-6.2082
53	C	3.7934	4.1723	-5.0499
54	C	2.9552	3.8445	-6.1237
55	H	1.8339	2.2508	-7.0460
56	H	4.2010	5.1781	-4.9816
57	C	4.8737	3.5826	0.8046
58	H	4.8531	2.8176	1.5900
59	H	4.6083	3.0772	-0.1274
60	H	4.0960	4.3207	1.0272
61	C	10.0009	3.4365	0.7073
62	H	9.9798	2.5906	0.0156
63	H	10.2512	3.0341	1.6972
64	H	10.8161	4.1028	0.4070
65	C	7.5558	7.8465	0.5616
66	H	7.0764	8.2015	-0.3598
67	H	8.5836	8.2239	0.5699
68	H	7.0194	8.3099	1.3998
69	C	10.7241	-2.9744	-4.6431
70	H	11.3123	-3.8991	-4.6969
71	H	10.4899	-2.7897	-3.5942
72	H	11.3729	-2.1602	-4.9873
73	C	5.6437	-2.8125	-5.4162
74	H	5.4357	-1.7786	-5.1224
75	H	5.4500	-3.4294	-4.5301
76	H	4.9284	-3.0943	-6.1949
77	C	8.7852	-4.0387	-9.1643
78	H	8.4207	-5.0685	-9.2799
79	H	9.8423	-4.0315	-9.4478
80	H	8.2337	-3.4225	-9.8822
81	C	5.0521	3.6705	-2.9370
82	H	4.4917	4.2379	-2.1828
83	H	5.8395	4.3283	-3.3140
84	H	5.5294	2.8315	-2.4311
85	C	2.2214	0.1646	-5.3650
86	H	1.3846	-0.0021	-4.6745
87	H	2.9777	-0.5942	-5.1344
88	H	1.8546	-0.0171	-6.3806
89	C	2.6560	4.8572	-7.2053
90	H	2.6216	5.8761	-6.8034
91	H	1.7009	4.6514	-7.7007
92	H	3.4361	4.8397	-7.9804
93	H	7.1935	2.7285	-4.8279
94	N	6.8732	1.7444	-6.5236
95	C	6.2394	2.9411	-7.1269
96	H	5.1660	2.8623	-6.9371
97	C	6.4775	3.1385	-8.6211
98	H	6.0003	4.0798	-8.9196
99	H	7.5405	3.1988	-8.8524
100	H	6.0288	2.3423	-9.2240
101	H	6.6117	3.8031	-6.5710
102	Cu	11.7366	2.8001	-6.1066
103	N	12.6639	2.8911	-8.1191
104	C	12.5966	2.1725	-9.2586
105	C	14.5304	3.2824	-9.2817
106	C	13.7692	2.3843	-10.0256
107	H	15.4860	3.7412	-9.4877
108	H	14.0022	1.9614	-10.9904
109	N	13.6919	2.8678	-5.2231
110	C	14.2798	2.4230	-4.0893
111	C	15.4655	3.1603	-3.8473
112	C	15.5585	4.0568	-4.9041
113	H	16.1554	3.0274	-3.0295
114	H	16.3071	4.7976	-5.1403
115	N	11.8729	5.1587	-6.0943
116	C	10.9935	6.1815	-6.1580
117	C	11.5208	7.2211	-6.9679
118	C	12.7697	6.7655	-7.3731
119	H	11.0578	8.1703	-7.1881
120	H	13.5288	7.2288	-7.9858
121	N	13.8403	3.5686	-8.1449

122	N	12.9566	5.5318	-6.8294
123	N	14.4859	3.8588	-5.7148
124	H	15.1276	5.2413	-7.3673
125	C	9.2352	1.3686	-5.1178
126	C	9.7967	2.3335	-6.2327
127	O	9.1460	2.7279	-7.1636
128	C	10.1656	0.2010	-3.1937
129	H	11.2537	0.1208	-3.2646
130	H	9.7295	-0.6247	-3.7537
131	B	14.1680	4.5937	-7.0353
132	C	9.7188	0.1098	-1.7405
133	H	8.6457	0.2736	-1.6523
134	H	10.2376	0.8336	-1.1077
135	H	9.9470	-0.8882	-1.3527
136	C	13.8084	1.2504	-3.3161
137	C	13.7798	-0.0287	-3.9263
138	C	13.4989	1.3807	-1.9390
139	C	13.4907	-1.1549	-3.1398
140	C	13.1963	0.2308	-1.1938
141	C	13.1986	-1.0475	-1.7722
142	H	13.4844	-2.1348	-3.6085
143	H	12.9515	0.3362	-0.1396
144	C	9.7394	6.2466	-5.3641
145	C	8.4792	6.2653	-6.0076
146	C	9.8127	6.4230	-3.9584
147	C	7.3211	6.4972	-5.2451
148	C	8.6370	6.6751	-3.2377
149	C	7.3826	6.7331	-3.8638
150	H	6.3564	6.5158	-5.7467
151	H	8.6974	6.8212	-2.1627
152	C	11.3709	1.4217	-9.6267
153	C	10.2487	2.1448	-10.0941
154	C	11.3177	0.0117	-9.5384
155	C	9.0965	1.4431	-10.4800
156	C	10.1416	-0.6526	-9.9171
157	C	9.0206	0.0463	-10.3933
158	H	8.2391	1.9999	-10.8475
159	H	10.1033	-1.7345	-9.8477
160	C	14.0226	-0.1968	-5.4096
161	H	14.9362	0.3116	-5.7398
162	H	13.1992	0.2441	-5.9846
163	H	14.0993	-1.2554	-5.6764
164	C	13.4838	2.7346	-1.2606
165	H	13.0366	3.5030	-1.9006
166	H	14.4973	3.0768	-1.0147
167	H	12.9150	2.6942	-0.3259
168	C	12.8372	-2.2659	-0.9542
169	H	11.7495	-2.3317	-0.8116
170	H	13.2898	-2.2298	0.0437
171	H	13.1617	-3.1901	-1.4440
172	C	11.1378	6.3545	-3.2299
173	H	11.8669	7.0603	-3.6475
174	H	11.5866	5.3577	-3.3123
175	H	11.0112	6.5844	-2.1672
176	C	8.3686	6.0491	-7.5008
177	H	8.8541	5.1150	-7.7993
178	H	8.8503	6.8576	-8.0638
179	H	7.3205	6.0019	-7.8124
180	C	6.1446	7.0719	-3.0638
181	H	6.1246	8.1411	-2.8111
182	H	6.1130	6.5146	-2.1213
183	H	5.2298	6.8496	-3.6224
184	C	12.5122	-0.7800	-9.0533
185	H	13.3435	-0.7321	-9.7685
186	H	12.2539	-1.8346	-8.9105
187	H	12.8900	-0.3907	-8.1021
188	C	10.2805	3.6543	-10.1697
189	H	11.1057	4.0094	-10.8005
190	H	10.4289	4.0865	-9.1749
191	H	9.3451	4.0483	-10.5797

192	C	7.7587	-0.6903	-10.7855
193	H	7.9622	-1.4703	-11.5295
194	H	7.0145	-0.0074	-11.2094
195	H	7.3014	-1.1839	-9.9185
196	H	9.2838	0.3641	-5.5438
197	N	9.7418	1.4299	-3.8696
198	C	10.0262	2.7129	-3.2372
199	H	10.8140	2.5421	-2.4990
200	C	8.8131	3.3711	-2.5814
201	H	9.1336	4.2220	-1.9776
202	H	8.1161	3.7477	-3.3310
203	H	8.2822	2.6704	-1.9345
204	H	10.4492	3.3816	-4.0013

DCM_INTCC2_BS2
Energy (POTENTIAL) = -4508.10094826 Eh

	Atom	X	Y	Z
1	Cu	-2.5247	-1.5305	1.7189
2	N	-3.8958	-1.3467	3.3217
3	C	-5.1795	-0.9372	3.2994
4	C	-4.6684	-1.4305	5.4097
5	C	-5.7118	-0.9730	4.6101
6	H	-4.6196	-1.6085	6.4735
7	H	-6.7124	-0.7109	4.9160
8	N	-1.5604	0.4412	4.6601
9	C	-0.6476	1.3163	5.1375
10	C	0.2199	0.6606	6.0552
11	C	-0.2383	-0.6494	6.1044
12	H	1.0376	1.0925	6.6102
13	H	0.1043	-1.4931	6.6852
14	N	-1.6207	-3.0913	2.7488
15	C	-0.9336	-4.1726	2.3141
16	C	-0.3382	-4.8292	3.4182
17	C	-0.7311	-4.0960	4.5303
18	H	0.2614	-5.7249	3.3948
19	H	-0.5322	-4.2511	5.5795
20	N	-3.5903	-1.6545	4.6077
21	N	-1.5052	-3.0592	4.1063
22	N	-1.3048	-0.7490	5.2558
23	H	-2.2444	-2.5698	6.1409
24	C	-2.6882	-0.3577	0.1977
25	C	-2.9382	-1.9851	-1.5703
26	H	-2.4478	-2.5866	-0.8058
27	H	-2.3140	-2.0110	-2.4648
28	B	-2.1718	-2.0249	5.0659
29	C	-4.3394	-2.5153	-1.8587
30	H	-4.8399	-1.9427	-2.6474
31	H	-4.9583	-2.4736	-0.9574
32	H	-4.2719	-3.5574	-2.1884
33	C	-0.6541	2.7412	4.7269
34	C	-1.8640	3.4890	4.7685
35	C	0.5376	3.3801	4.2976
36	C	-1.8637	4.8237	4.3370
37	C	0.4927	4.7218	3.8828
38	C	-0.6968	5.4609	3.8870
39	H	-2.7932	5.3868	4.3717
40	H	1.4110	5.1956	3.5447
41	C	-0.9326	-4.6239	0.9023
42	C	-2.1569	-4.9482	0.2603
43	C	0.2937	-4.8382	0.2207
44	C	-2.1273	-5.4940	-1.0336
45	C	0.2748	-5.3765	-1.0753
46	C	-0.9238	-5.7297	-1.7141
47	H	-3.0667	-5.7599	-1.5114
48	H	1.2190	-5.5424	-1.5887
49	C	-5.8183	-0.5604	2.0137
50	C	-6.6372	-1.4942	1.3362
51	C	-5.5678	0.7091	1.4423
52	C	-7.1978	-1.1410	0.0995
53	C	-6.1440	1.0238	0.2010

54	C	-6.9574	0.1117	-0.4872
55	H	-7.8233	-1.8615	-0.4219
56	H	-5.9475	1.9988	-0.2378
57	C	-3.1434	2.9045	5.3326
58	H	-2.9765	2.4935	6.3368
59	H	-3.5267	2.0833	4.7248
60	H	-3.9196	3.6734	5.4048
61	C	1.8746	2.6689	4.2694
62	H	1.7714	1.6124	4.0162
63	H	2.3739	2.7141	5.2468
64	H	2.5466	3.1346	3.5411
65	C	-0.7196	6.9114	3.4614
66	H	-1.6673	7.1723	2.9780
67	H	0.0927	7.1395	2.7633
68	H	-0.6025	7.5793	4.3264
69	C	1.6317	-4.5330	0.8613
70	H	2.0096	-5.3970	1.4244
71	H	1.5770	-3.6972	1.5604
72	H	2.3782	-4.2892	0.0993
73	C	-3.4960	-4.7356	0.9349
74	H	-3.7763	-3.6751	0.9382
75	H	-3.4812	-5.0548	1.9826
76	H	-4.2852	-5.2876	0.4146
77	C	-0.9074	-6.3807	-3.0788
78	H	-0.5086	-7.4024	-3.0188
79	H	-0.2723	-5.8299	-3.7822
80	H	-1.9124	-6.4426	-3.5074
81	C	-4.6988	1.7222	2.1532
82	H	-5.1929	2.1006	3.0569
83	H	-4.4816	2.5788	1.5069
84	H	-3.7482	1.2832	2.4719
85	C	-6.8814	-2.8697	1.9177
86	H	-7.3958	-2.8151	2.8847
87	H	-5.9361	-3.3989	2.0925
88	H	-7.4919	-3.4795	1.2442
89	C	-7.5161	0.4445	-1.8517
90	H	-7.5281	1.5251	-2.0298
91	H	-8.5379	0.0667	-1.9732
92	H	-6.9083	-0.0126	-2.6460
93	H	-2.7398	0.7208	0.3766
94	N	-2.9491	-0.6011	-1.0604
95	C	-3.3862	0.4624	-2.0074
96	H	-4.4812	0.4416	-2.0333
97	C	-2.8164	0.3265	-3.4142
98	H	-3.0821	1.2235	-3.9861
99	H	-1.7273	0.2541	-3.3998
100	H	-3.2243	-0.5392	-3.9442
101	H	-3.0827	1.4132	-1.5637
102	Cu	2.7811	1.0582	-2.2598
103	N	3.2647	0.3845	-4.3292
104	C	2.6390	-0.2762	-5.3247
105	C	4.5216	0.5758	-6.1659
106	C	3.4065	-0.1773	-6.5146
107	H	5.3750	0.8895	-6.7481
108	H	3.1761	-0.6074	-7.4764
109	N	4.8325	0.4930	-1.7622
110	C	5.4845	-0.2506	-0.8401
111	C	6.8387	-0.4120	-1.2244
112	C	6.9609	0.2814	-2.4211
113	H	7.6062	-0.9440	-0.6852
114	H	7.8135	0.4393	-3.0641
115	N	3.5940	3.0462	-2.8263
116	C	3.3172	4.3443	-2.5799
117	C	4.3497	5.1607	-3.1011
118	C	5.2553	4.2746	-3.6773
119	H	4.4089	6.2373	-3.0657
120	H	6.1848	4.4504	-4.1979
121	N	4.4067	0.8999	-4.8487
122	N	4.7753	3.0141	-3.4977
123	N	5.7470	0.8151	-2.7206

124	H	6.4034	1.9029	-4.5772
125	C	0.6188	0.0573	-1.0560
126	C	0.7937	1.1067	-2.0204
127	O	0.0262	1.8002	-2.6642
128	C	1.0718	-1.0276	1.0494
129	H	2.1348	-1.2494	0.9110
130	H	0.4932	-1.8447	0.6097
131	B	5.3974	1.6782	-3.9566
132	C	0.7333	-0.9129	2.5258
133	H	-0.3031	-0.6091	2.6732
134	H	1.3816	-0.2084	3.0461
135	H	0.8672	-1.8902	2.9954
136	C	4.8801	-0.7170	0.4296
137	C	4.8097	-2.1007	0.7215
138	C	4.4822	0.2310	1.4053
139	C	4.3674	-2.5105	1.9898
140	C	4.0648	-0.2201	2.6656
141	C	4.0058	-1.5861	2.9805
142	H	4.3030	-3.5735	2.2066
143	H	3.7703	0.5103	3.4138
144	C	2.0585	4.7403	-1.9027
145	C	0.8566	4.7624	-2.6459
146	C	2.0484	5.0617	-0.5252
147	C	-0.3442	5.0803	-1.9925
148	C	0.8277	5.3680	0.0937
149	C	-0.3808	5.3738	-0.6219
150	H	-1.2701	5.0786	-2.5622
151	H	0.8156	5.5844	1.1578
152	C	1.3751	-1.0180	-5.1124
153	C	0.2349	-0.7100	-5.8986
154	C	1.3078	-2.0646	-4.1586
155	C	-0.9252	-1.4842	-5.7572
156	C	0.1177	-2.8022	-4.0354
157	C	-0.9989	-2.5467	-4.8443
158	H	-1.7929	-1.2474	-6.3682
159	H	0.0716	-3.6004	-3.3008
160	C	5.1891	-3.1394	-0.3128
161	H	6.2696	-3.1518	-0.4998
162	H	4.7040	-2.9418	-1.2760
163	H	4.8980	-4.1416	0.0164
164	C	4.5022	1.7125	1.1025
165	H	3.7965	1.9596	0.3025
166	H	5.4897	2.0432	0.7571
167	H	4.2342	2.2974	1.9871
168	C	3.5562	-2.0481	4.3481
169	H	3.0358	-3.0110	4.2947
170	H	2.8785	-1.3229	4.8105
171	H	4.4120	-2.1767	5.0254
172	C	3.3293	5.0855	0.2809
173	H	3.9000	6.0052	0.0930
174	H	3.9854	4.2484	0.0268
175	H	3.1175	5.0433	1.3546
176	C	0.8615	4.4289	-4.1204
177	H	1.2276	3.4100	-4.2820
178	H	1.5141	5.1081	-4.6844
179	H	-0.1468	4.4948	-4.5411
180	C	-1.6849	5.6765	0.0821
181	H	-1.7365	6.7264	0.3990
182	H	-1.7955	5.0637	0.9849
183	H	-2.5447	5.4819	-0.5679
184	C	2.4889	-2.4244	-3.2813
185	H	3.4160	-2.5029	-3.8611
186	H	2.3156	-3.3784	-2.7734
187	H	2.6675	-1.6601	-2.5163
188	C	0.2404	0.4489	-6.8730
189	H	0.8149	0.2227	-7.7795
190	H	0.6900	1.3425	-6.4255
191	H	-0.7798	0.6986	-7.1823
192	C	-2.2428	-3.4032	-4.7569
193	H	-2.2667	-4.1420	-5.5698

194	H	-3.1536	-2.7993	-4.8444
195	H	-2.2869	-3.9534	-3.8133
196	H	0.6193	-0.9492	-1.4708
197	N	0.7354	0.1801	0.2631
198	C	0.8551	1.5282	0.8340
199	H	1.4430	1.4597	1.7477
200	C	-0.4932	2.1900	1.1092
201	H	-0.3356	3.1824	1.5387
202	H	-1.0608	2.3047	0.1803
203	H	-1.0807	1.5991	1.8174
204	H	1.4380	2.1301	0.1202

DCM_CuCO
Energy (POTENTIAL) = -2060.39474398 Eh

	Atom	X	Y	Z
1	Cu	16.0306	-4.2931	6.0507
2	N	17.0675	-4.4237	7.8894
3	C	16.9278	-3.9040	9.1181
4	C	18.8248	-5.0632	9.0864
5	C	18.0256	-4.2906	9.9203
6	H	19.7655	-5.5644	9.2684
7	H	18.2030	-4.0364	10.9552
8	N	17.8844	-3.7671	5.2189
9	C	18.3637	-2.7329	4.5115
10	C	19.7591	-2.8837	4.3436
11	C	20.0756	-4.0612	5.0090
12	H	20.4306	-2.2209	3.8173
13	H	21.0224	-4.5628	5.1543
14	N	16.4552	-6.3225	5.7250
15	C	15.7542	-7.3924	5.3176
16	C	16.5835	-8.5329	5.3288
17	C	17.8201	-8.0717	5.7709
18	H	16.3140	-9.5427	5.0541
19	H	18.7542	-8.5905	5.9369
20	N	18.2317	-5.1287	7.8719
21	N	17.7180	-6.7433	6.0012
22	N	18.9363	-4.5755	5.5277
23	H	19.8114	-6.2864	6.7285
24	C	14.4053	-3.5527	5.7652
25	O	13.3595	-3.1182	5.5801
26	B	18.7604	-5.7387	6.5427
27	C	17.4710	-1.6266	4.0845
28	C	16.4536	-1.8459	3.1329
29	C	17.6287	-0.3475	4.6710
30	C	15.6248	-0.7755	2.7623
31	C	16.7793	0.6910	4.2745
32	C	15.7728	0.4994	3.3166
33	H	14.8457	-0.9475	2.0225
34	H	16.8994	1.6727	4.7287
35	C	14.3265	-7.2099	4.9557
36	C	13.3256	-7.4113	5.9276
37	C	13.9882	-6.7475	3.6651
38	C	11.9962	-7.1025	5.6041
39	C	12.6493	-6.4548	3.3808
40	C	11.6405	-6.6112	4.3423
41	H	11.2246	-7.2406	6.3588
42	H	12.3873	-6.0887	2.3898
43	C	15.7633	-3.0403	9.4350
44	C	15.9650	-1.6697	9.7107
45	C	14.4532	-3.5724	9.4151
46	C	14.8521	-0.8554	9.9656
47	C	13.3704	-2.7228	9.6718
48	C	13.5471	-1.3611	9.9498
49	H	15.0110	0.2009	10.1722
50	H	12.3630	-3.1347	9.6549
51	C	16.2153	-3.2105	2.5322
52	H	15.6480	-3.8388	3.2264
53	H	17.1511	-3.7373	2.3219
54	H	15.6414	-3.1366	1.6035
55	C	18.6803	-0.0940	5.7282

56	H	19.6921	-0.1219	5.3083
57	H	18.6426	-0.8540	6.5162
58	H	18.5365	0.8861	6.1928
59	C	14.8838	1.6480	2.9007
60	H	15.4730	2.4635	2.4637
61	H	14.3471	2.0653	3.7614
62	H	14.1422	1.3333	2.1600
63	C	15.0466	-6.6025	2.5955
64	H	15.3578	-7.5875	2.2242
65	H	15.9445	-6.1102	2.9771
66	H	14.6722	-6.0275	1.7439
67	C	13.6640	-7.9755	7.2886
68	H	14.5780	-7.5368	7.6975
69	H	13.8300	-9.0587	7.2275
70	H	12.8506	-7.8044	7.9995
71	C	10.2137	-6.2289	4.0248
72	H	9.5129	-6.6478	4.7537
73	H	9.9195	-6.5751	3.0276
74	H	10.0919	-5.1380	4.0369
75	C	14.1956	-5.0273	9.1011
76	H	14.9410	-5.6837	9.5601
77	H	14.2404	-5.2066	8.0221
78	H	13.2039	-5.3325	9.4480
79	C	17.3505	-1.0629	9.7161
80	H	17.9089	-1.3311	8.8126
81	H	17.9410	-1.4131	10.5704
82	H	17.2961	0.0285	9.7709
83	C	12.3564	-0.4758	10.2354
84	H	11.8549	-0.7787	11.1631
85	H	11.6122	-0.5396	9.4325
86	H	12.6515	0.5729	10.3403

DCM_metallocarbene6_BS2
Energy (POTENTIAL) = -2197.37477054 Eh

	Atom	X	Y	Z
1	Cu	8.5163	10.4729	8.4728
2	N	8.2756	8.2037	6.5281
3	N	7.2133	13.0752	9.6931
4	N	6.9873	12.1742	8.7102
5	N	9.7308	13.0973	9.4326
6	N	9.9765	12.0751	8.5690
7	N	8.6603	11.7227	11.2560
8	N	8.5949	10.5215	10.6281
9	C	7.7679	9.1238	7.3070
10	H	6.6720	9.0708	7.2559
11	C	9.7346	8.0405	6.4011
12	H	9.9842	8.0248	5.3338
13	H	10.1787	8.9314	6.8413
14	C	10.2439	6.7823	7.0998
15	H	9.8162	5.8718	6.6639
16	H	9.9965	6.8076	8.1659
17	H	11.3340	6.7277	6.9980
18	C	7.4279	7.2695	5.7373
19	H	6.7162	7.8764	5.1696
20	H	8.0762	6.7534	5.0240
21	C	6.6897	6.2631	6.6175
22	H	6.0748	5.6062	5.9901
23	H	6.0292	6.7770	7.3239
24	H	7.3904	5.6452	7.1879
25	C	6.0955	13.8221	9.9219
26	H	6.0813	14.5991	10.6719
27	C	5.1066	13.3824	9.0449
28	H	4.0999	13.7575	8.9475
29	C	5.7158	12.3399	8.2989
30	C	5.1881	11.4592	7.2298
31	C	4.1371	10.5502	7.5112
32	C	3.4676	10.5246	8.8690
33	H	4.2058	10.5063	9.6785
34	H	2.8270	9.6429	8.9714
35	H	2.8424	11.4116	9.0299

36	C	3.7364	9.6386	6.5232
37	H	2.9371	8.9359	6.7469
38	C	4.3475	9.6030	5.2591
39	C	3.9116	8.5906	4.2237
40	H	4.0480	7.5642	4.5896
41	H	4.4825	8.6951	3.2952
42	H	2.8477	8.7012	3.9772
43	C	5.3771	10.5167	4.9954
44	H	5.8619	10.5042	4.0223
45	C	5.8132	11.4414	5.9595
46	C	6.9750	12.3565	5.6349
47	H	6.8098	13.3756	6.0018
48	H	7.1476	12.4005	4.5543
49	H	7.8934	11.9941	6.1133
50	C	10.6782	14.0658	9.2982
51	H	10.6576	14.9559	9.9087
52	C	11.5704	13.6583	8.3119
53	H	12.4463	14.1785	7.9576
54	C	11.0928	12.3942	7.8843
55	C	11.7038	11.4548	6.9125
56	C	11.7184	11.7589	5.5306
57	C	11.0701	13.0228	5.0076
58	H	10.0345	13.1149	5.3567
59	H	11.0624	13.0355	3.9131
60	H	11.6002	13.9201	5.3497
61	C	12.3342	10.8650	4.6396
62	H	12.3386	11.0978	3.5777
63	C	12.9486	9.6845	5.0856
64	C	13.6463	8.7562	4.1168
65	H	14.7177	8.9908	4.0455
66	H	13.2274	8.8395	3.1079
67	H	13.5662	7.7104	4.4353
68	C	12.9153	9.3960	6.4584
69	H	13.3727	8.4786	6.8206
70	C	12.2927	10.2533	7.3784
71	C	12.2123	9.8585	8.8368
72	H	11.1812	9.6026	9.1107
73	H	12.5170	10.6766	9.5000
74	H	12.8451	8.9902	9.0454
75	C	8.7950	11.5444	12.5992
76	H	8.8586	12.3866	13.2719
77	C	8.8203	10.1744	12.8485
78	H	8.9147	9.6794	13.8021
79	C	8.6885	9.5671	11.5749
80	C	8.6403	8.1361	11.1924
81	C	9.7905	7.3226	11.3358
82	C	11.0728	7.8823	11.9139
83	H	10.9932	8.0368	12.9976
84	H	11.3210	8.8539	11.4734
85	H	11.9119	7.2021	11.7361
86	C	9.7359	5.9828	10.9232
87	H	10.6227	5.3621	11.0279
88	C	8.5693	5.4269	10.3739
89	C	8.5501	3.9908	9.9009
90	H	7.5316	3.6551	9.6790
91	H	8.9748	3.3135	10.6519
92	H	9.1470	3.8685	8.9863
93	C	7.4382	6.2460	10.2497
94	H	6.5241	5.8303	9.8339
95	C	7.4527	7.5933	10.6455
96	C	6.2064	8.4378	10.4855
97	H	6.3521	9.2208	9.7341
98	H	5.9432	8.9469	11.4210
99	H	5.3533	7.8245	10.1784
100	B	8.5619	13.0442	10.4476
101	H	8.6339	13.9813	11.1992

DCM_INTCC3_BS2
Energy (POTENTIAL) = -4508.15154266 Eh

	Atom	X	Y	Z

1	Cu	-2.3856	-1.3243	1.6540
2	N	-3.7365	-0.8302	3.1425
3	C	-4.9063	-0.1574	3.1184
4	C	-4.6382	-0.9418	5.1827
5	C	-5.5119	-0.2038	4.3957
6	H	-4.6867	-1.2144	6.2258
7	H	-6.4523	0.2386	4.6837
8	N	-1.5163	0.5443	4.8733
9	C	-0.4706	1.2813	5.3077
10	C	0.5076	0.4299	5.8943
11	C	-0.0219	-0.8513	5.7854
12	H	1.4503	0.7122	6.3358
13	H	0.3734	-1.8050	6.1024
14	N	-1.7266	-3.0815	2.7270
15	C	-1.0982	-4.2229	2.3586
16	C	-0.6419	-4.9085	3.5103
17	C	-1.0485	-4.1261	4.5828
18	H	-0.1185	-5.8509	3.5388
19	H	-0.9384	-4.2759	5.6460
20	N	-3.5821	-1.3151	4.4096
21	N	-1.6983	-3.0367	4.0891
22	N	-1.2326	-0.7471	5.1600
23	H	-2.5146	-2.3841	6.0443
24	C	-2.2354	-0.2665	-0.4055
25	C	-3.1339	-2.1272	-1.6861
26	H	-2.8541	-2.7386	-0.8252
27	H	-2.2972	-2.1972	-2.4005
28	B	-2.2737	-1.8935	4.9658
29	C	-4.4031	-2.7082	-2.3034
30	H	-4.6731	-2.2164	-3.2445
31	H	-5.2494	-2.6199	-1.6124
32	H	-4.2461	-3.7706	-2.5190
33	C	-0.4669	2.7485	5.0924
34	C	-1.6261	3.5135	5.4010
35	C	0.6549	3.3994	4.5156
36	C	-1.6592	4.8782	5.0764
37	C	0.5783	4.7689	4.2113
38	C	-0.5747	5.5233	4.4636
39	H	-2.5509	5.4526	5.3163
40	H	1.4395	5.2528	3.7566
41	C	-1.0196	-4.7054	0.9587
42	C	-2.2153	-5.0053	0.2552
43	C	0.2350	-4.9799	0.3576
44	C	-2.1342	-5.5796	-1.0225
45	C	0.2697	-5.5439	-0.9286
46	C	-0.9013	-5.8709	-1.6271
47	H	-3.0532	-5.8249	-1.5481
48	H	1.2355	-5.7568	-1.3809
49	C	-5.4222	0.5138	1.9012
50	C	-6.1621	-0.2222	0.9479
51	C	-5.2117	1.9012	1.7239
52	C	-6.6887	0.4435	-0.1685
53	C	-5.7441	2.5290	0.5868
54	C	-6.4829	1.8168	-0.3699
55	H	-7.2568	-0.1234	-0.9019
56	H	-5.5759	3.5933	0.4458
57	C	-2.8242	2.9065	6.1027
58	H	-2.5161	2.2812	6.9490
59	H	-3.4024	2.2609	5.4370
60	H	-3.4866	3.6928	6.4802
61	C	1.9382	2.6661	4.1871
62	H	1.7480	1.7058	3.7014
63	H	2.5300	2.4565	5.0873
64	H	2.5617	3.2669	3.5177
65	C	-0.6578	6.9813	4.0720
66	H	-1.1918	7.1045	3.1192
67	H	0.3376	7.4217	3.9482
68	H	-1.1999	7.5692	4.8220
69	C	1.5480	-4.7327	1.0725
70	H	1.8897	-5.6398	1.5898

71	H	1.4733	-3.9455	1.8240
72	H	2.3307	-4.4527	0.3592
73	C	-3.5742	-4.7560	0.8753
74	H	-3.7500	-3.6890	1.0456
75	H	-3.6633	-5.2443	1.8539
76	H	-4.3732	-5.1339	0.2299
77	C	-0.8312	-6.5715	-2.9653
78	H	-0.5727	-7.6313	-2.8349
79	H	-0.0658	-6.1310	-3.6136
80	H	-1.7887	-6.5293	-3.4933
81	C	-4.4344	2.7012	2.7458
82	H	-4.9936	2.7930	3.6854
83	H	-4.2247	3.7109	2.3812
84	H	-3.4814	2.2227	2.9934
85	C	-6.3781	-1.7084	1.1269
86	H	-6.8825	-1.9305	2.0759
87	H	-5.4227	-2.2450	1.1454
88	H	-6.9841	-2.1214	0.3147
89	C	-6.9897	2.4986	-1.6201
90	H	-7.2128	3.5563	-1.4415
91	H	-7.8958	2.0167	-2.0039
92	H	-6.2342	2.4559	-2.4184
93	H	-2.3399	0.7898	-0.1867
94	N	-3.2919	-0.7626	-1.1931
95	C	-3.8824	0.2198	-2.1129
96	H	-4.8955	-0.1107	-2.3599
97	C	-3.0844	0.4699	-3.3990
98	H	-3.6032	1.2115	-4.0210
99	H	-2.0826	0.8460	-3.1771
100	H	-2.9747	-0.4471	-3.9893
101	H	-3.9982	1.1556	-1.5575
102	Cu	2.9106	0.7939	-2.3128
103	N	3.5080	0.2761	-4.4017
104	C	3.0027	-0.4321	-5.4332
105	C	4.7669	0.7024	-6.1943
106	C	3.7780	-0.1902	-6.5957
107	H	5.5775	1.1561	-6.7445
108	H	3.6315	-0.6127	-7.5769
109	N	4.9533	0.6334	-1.7739
110	C	5.6846	0.0071	-0.8290
111	C	7.0590	0.0858	-1.1613
112	C	7.1047	0.8027	-2.3508
113	H	7.8859	-0.3091	-0.5929
114	H	7.9411	1.1120	-2.9590
115	N	3.2674	2.8294	-2.8588
116	C	2.6465	4.0117	-2.6765
117	C	3.4517	5.0514	-3.2020
118	C	4.5789	4.4145	-3.7149
119	H	3.2291	6.1066	-3.2114
120	H	5.4476	4.8118	-4.2179
121	N	4.5764	0.9659	-4.8726
122	N	4.4420	3.0790	-3.4941
123	N	5.8299	1.1226	-2.6963
124	H	6.3185	2.3559	-4.5199
125	C	-1.0405	-0.9059	-0.1141
126	C	1.1490	0.1549	-2.2540
127	O	0.1167	-0.1077	-2.6720
128	C	1.1473	-1.0845	0.9626
129	H	2.1303	-0.6845	0.6876
130	H	1.0785	-2.0852	0.5257
131	B	5.3629	1.9149	-3.9395
132	C	1.0272	-1.1683	2.4876
133	H	-0.0059	-1.3402	2.7878
134	H	1.3749	-0.2526	2.9685
135	H	1.6328	-1.9903	2.8773
136	C	5.1088	-0.5417	0.4196
137	C	5.1716	-1.9288	0.6887
138	C	4.5988	0.3498	1.3962
139	C	4.7708	-2.3973	1.9501
140	C	4.2316	-0.1559	2.6517

141	C	4.3275	-1.5227	2.9535
142	H	4.8163	-3.4639	2.1556
143	H	3.8560	0.5292	3.4063
144	C	1.3016	4.0711	-2.0559
145	C	0.1842	3.5682	-2.7679
146	C	1.1313	4.6193	-0.7630
147	C	-1.0833	3.6205	-2.1690
148	C	-0.1554	4.6605	-0.2031
149	C	-1.2722	4.1582	-0.8861
150	H	-1.9401	3.2311	-2.7124
151	H	-0.2839	5.0764	0.7925
152	C	1.7946	-1.2729	-5.2657
153	C	0.6258	-0.9671	-6.0083
154	C	1.7790	-2.3415	-4.3359
155	C	-0.5267	-1.7420	-5.8188
156	C	0.6024	-3.0911	-4.1756
157	C	-0.5576	-2.8125	-4.9127
158	H	-1.4228	-1.4997	-6.3852
159	H	0.5896	-3.8998	-3.4499
160	C	5.6602	-2.9038	-0.3616
161	H	6.7311	-2.7805	-0.5646
162	H	5.1387	-2.7579	-1.3153
163	H	5.4979	-3.9376	-0.0405
164	C	4.4369	1.8251	1.0965
165	H	3.6540	1.9896	0.3455
166	H	5.3557	2.2630	0.6876
167	H	4.1620	2.3802	1.9980
168	C	3.9621	-2.0410	4.3256
169	H	3.5438	-3.0527	4.2733
170	H	3.2270	-1.3936	4.8149
171	H	4.8453	-2.0880	4.9781
172	C	2.3149	5.1429	0.0216
173	H	2.7388	6.0453	-0.4361
174	H	3.1220	4.4018	0.0666
175	H	2.0249	5.3912	1.0475
176	C	0.3377	3.0013	-4.1641
177	H	1.0704	2.1899	-4.1937
178	H	0.6909	3.7681	-4.8664
179	H	-0.6158	2.6134	-4.5360
180	C	-2.6359	4.1341	-0.2372
181	H	-3.4333	4.3181	-0.9659
182	H	-2.7170	4.8817	0.5591
183	H	-2.8334	3.1549	0.2170
184	C	2.9836	-2.6699	-3.4784
185	H	3.9226	-2.5813	-4.0353
186	H	2.9107	-3.6867	-3.0784
187	H	3.0580	-1.9807	-2.6269
188	C	0.5836	0.2021	-6.9702
189	H	1.1588	0.0039	-7.8830
190	H	1.0059	1.1080	-6.5201
191	H	-0.4469	0.4192	-7.2693
192	C	-1.8071	-3.6490	-4.7541
193	H	-1.8183	-4.4825	-5.4701
194	H	-2.7090	-3.0538	-4.9340
195	H	-1.8769	-4.0764	-3.7508
196	H	-0.8663	-1.9181	-0.4681
197	N	0.1304	-0.2674	0.3013
198	C	0.1480	1.1746	0.5334
199	H	1.2021	1.4658	0.6098
200	C	-0.6152	1.6755	1.7643
201	H	-0.5343	2.7640	1.8345
202	H	-1.6770	1.4196	1.7252
203	H	-0.2217	1.2456	2.6812
204	H	-0.2465	1.6620	-0.3650

DCM_INTCC1_BS2

Energy (POTENTIAL) = -4508.09059140 Eh

	Atom	X	Y	Z
1	Cu	-2.5006	-1.4567	1.6572
2	N	-3.8521	-1.1981	3.2725

3	C	-5.1090	-0.7133	3.2588
4	C	-4.6464	-1.3183	5.3514
5	C	-5.6551	-0.7720	4.5635
6	H	-4.6152	-1.5361	6.4084
7	H	-6.6410	-0.4625	4.8732
8	N	-1.5498	0.4897	4.6790
9	C	-0.5975	1.3336	5.1352
10	C	0.3286	0.6239	5.9496
11	C	-0.1367	-0.6853	5.9603
12	H	1.1907	1.0184	6.4639
13	H	0.2428	-1.5627	6.4629
14	N	-1.6430	-3.0587	2.6875
15	C	-0.9713	-4.1502	2.2525
16	C	-0.3912	-4.8204	3.3565
17	C	-0.7762	-4.0849	4.4695
18	H	0.1946	-5.7252	3.3310
19	H	-0.5833	-4.2456	5.5190
20	N	-3.5753	-1.5708	4.5480
21	N	-1.5309	-3.0341	4.0456
22	N	-1.2633	-0.7324	5.1889
23	H	-2.2606	-2.5296	6.0800
24	C	-2.6200	-0.2231	0.1787
25	C	-2.9491	-1.7421	-1.6653
26	H	-2.3177	-2.3643	-1.0284
27	H	-2.4964	-1.7096	-2.6557
28	B	-2.1700	-1.9850	5.0054
29	C	-4.3659	-2.3102	-1.7175
30	H	-5.0197	-1.7274	-2.3733
31	H	-4.8088	-2.3177	-0.7181
32	H	-4.3315	-3.3390	-2.0933
33	C	-0.6264	2.7765	4.7893
34	C	-1.8250	3.5267	4.9547
35	C	0.5263	3.4231	4.2781
36	C	-1.8600	4.8675	4.5458
37	C	0.4494	4.7724	3.8924
38	C	-0.7371	5.5068	3.9957
39	H	-2.7824	5.4305	4.6685
40	H	1.3360	5.2512	3.4844
41	C	-0.9718	-4.6005	0.8409
42	C	-2.2038	-4.8870	0.1940
43	C	0.2496	-4.8561	0.1657
44	C	-2.1874	-5.4296	-1.1005
45	C	0.2177	-5.3941	-1.1308
46	C	-0.9884	-5.7056	-1.7752
47	H	-3.1328	-5.6672	-1.5818
48	H	1.1581	-5.5931	-1.6395
49	C	-5.7039	-0.2099	1.9958
50	C	-6.5505	-1.0414	1.2295
51	C	-5.3838	1.0923	1.5398
52	C	-7.0694	-0.5583	0.0167
53	C	-5.9270	1.5408	0.3273
54	C	-6.7641	0.7271	-0.4533
55	H	-7.7130	-1.2034	-0.5764
56	H	-5.6857	2.5432	-0.0185
57	C	-3.0534	2.9292	5.6112
58	H	-2.8095	2.5214	6.6008
59	H	-3.4682	2.1039	5.0307
60	H	-3.8311	3.6888	5.7413
61	C	1.8431	2.6989	4.0953
62	H	1.6964	1.6963	3.6884
63	H	2.3822	2.5855	5.0446
64	H	2.4953	3.2525	3.4125
65	C	-0.8170	6.9416	3.5258
66	H	-1.4720	7.0367	2.6493
67	H	0.1689	7.3274	3.2457
68	H	-1.2285	7.5976	4.3033
69	C	1.5985	-4.6010	0.8074
70	H	1.9868	-5.5094	1.2880
71	H	1.5554	-3.8257	1.5726
72	H	2.3321	-4.2940	0.0544

73	C	-3.5348	-4.6567	0.8785
74	H	-3.7729	-3.5891	0.9432
75	H	-3.5320	-5.0350	1.9072
76	H	-4.3443	-5.1495	0.3307
77	C	-0.9896	-6.3686	-3.1340
78	H	-0.7307	-7.4329	-3.0482
79	H	-0.2551	-5.9131	-3.8076
80	H	-1.9721	-6.3071	-3.6120
81	C	-4.4744	1.9931	2.3441
82	H	-4.9604	2.3164	3.2730
83	H	-4.2053	2.8902	1.7779
84	H	-3.5509	1.4811	2.6324
85	C	-6.8694	-2.4471	1.6892
86	H	-7.4257	-2.4467	2.6347
87	H	-5.9529	-3.0243	1.8634
88	H	-7.4697	-2.9791	0.9443
89	C	-7.2720	1.2129	-1.7918
90	H	-7.6045	2.2565	-1.7429
91	H	-8.1090	0.6051	-2.1511
92	H	-6.4782	1.1636	-2.5512
93	H	-2.6103	0.8483	0.4179
94	N	-2.8961	-0.3843	-1.0894
95	C	-3.2299	0.8017	-1.9478
96	H	-4.1307	1.2465	-1.5180
97	C	-3.4552	0.5378	-3.4330
98	H	-3.6824	1.4998	-3.9088
99	H	-2.5728	0.1258	-3.9267
100	H	-4.3055	-0.1259	-3.6142
101	H	-2.4109	1.5138	-1.8187
102	Cu	2.8733	0.8282	-2.2205
103	N	3.4152	0.3488	-4.2798
104	C	2.9165	-0.3654	-5.3100
105	C	4.7936	0.6084	-6.0154
106	C	3.7641	-0.2283	-6.4369
107	H	5.6617	0.9810	-6.5383
108	H	3.6347	-0.6768	-7.4091
109	N	4.9578	0.5483	-1.6210
110	C	5.6729	-0.1099	-0.6838
111	C	7.0476	-0.0861	-1.0252
112	C	7.1127	0.6320	-2.2139
113	H	7.8627	-0.5173	-0.4658
114	H	7.9562	0.9088	-2.8283
115	N	3.3555	2.8866	-2.6854
116	C	2.8382	4.1185	-2.5009
117	C	3.7326	5.0920	-3.0115
118	C	4.8052	4.3668	-3.5199
119	H	3.5977	6.1620	-3.0150
120	H	5.7083	4.6927	-4.0136
121	N	4.5557	0.9407	-4.7173
122	N	4.5540	3.0451	-3.3107
123	N	5.8468	1.0023	-2.5473
124	H	6.3702	2.2246	-4.3621
125	C	1.3010	-0.3311	-1.2229
126	C	0.4187	-0.0222	-2.1987
127	O	-0.3759	0.3620	-2.9618
128	C	1.0697	-1.1797	0.9825
129	H	2.1117	-1.5296	0.8914
130	H	0.4230	-1.9762	0.5997
131	B	5.3981	1.8317	-3.7726
132	C	0.7573	-0.9579	2.4545
133	H	-0.2728	-0.6386	2.6101
134	H	1.4229	-0.2241	2.9098
135	H	0.8954	-1.9035	2.9855
136	C	5.0660	-0.6528	0.5558
137	C	4.9441	-2.0473	0.7527
138	C	4.6731	0.2452	1.5791
139	C	4.4461	-2.5234	1.9771
140	C	4.1821	-0.2701	2.7870
141	C	4.0675	-1.6516	3.0082
142	H	4.3470	-3.5957	2.1245

143	H	3.8779	0.4198	3.5698
144	C	1.4944	4.3118	-1.9060
145	C	0.3486	3.8822	-2.6171
146	C	1.3523	4.9357	-0.6423
147	C	-0.9203	4.0722	-2.0429
148	C	0.0681	5.1253	-0.1151
149	C	-1.0800	4.6878	-0.7934
150	H	-1.7995	3.7344	-2.5859
151	H	-0.0380	5.5844	0.8641
152	C	1.6506	-1.1206	-5.1645
153	C	0.5077	-0.7229	-5.8997
154	C	1.5672	-2.2122	-4.2660
155	C	-0.6891	-1.4366	-5.7431
156	C	0.3415	-2.8841	-4.1183
157	C	-0.7912	-2.5252	-4.8638
158	H	-1.5632	-1.1306	-6.3128
159	H	0.2764	-3.7058	-3.4122
160	C	5.3289	-3.0220	-0.3395
161	H	6.4001	-2.9706	-0.5695
162	H	4.7954	-2.8061	-1.2733
163	H	5.0971	-4.0509	-0.0470
164	C	4.7948	1.7416	1.3822
165	H	4.2193	2.0786	0.5134
166	H	5.8372	2.0362	1.2031
167	H	4.4340	2.2825	2.2620
168	C	3.5475	-2.1788	4.3264
169	H	3.1396	-3.1900	4.2212
170	H	2.7572	-1.5348	4.7274
171	H	4.3467	-2.2220	5.0797
172	C	2.5658	5.3497	0.1623
173	H	3.1366	6.1451	-0.3314
174	H	3.2538	4.5062	0.3019
175	H	2.2711	5.7118	1.1523
176	C	0.4733	3.2481	-3.9870
177	H	1.0855	2.3425	-3.9581
178	H	0.9555	3.9332	-4.6970
179	H	-0.5094	2.9788	-4.3848
180	C	-2.4417	4.8335	-0.1532
181	H	-2.6799	5.8840	0.0574
182	H	-2.4767	4.2985	0.8051
183	H	-3.2334	4.4301	-0.7937
184	C	2.7815	-2.7039	-3.5036
185	H	3.4945	-3.1956	-4.1802
186	H	2.4908	-3.4316	-2.7395
187	H	3.3239	-1.8883	-3.0180
188	C	0.5498	0.4773	-6.8214
189	H	1.1576	0.2867	-7.7143
190	H	0.9882	1.3481	-6.3199
191	H	-0.4573	0.7480	-7.1547
192	C	-2.0809	-3.3085	-4.7545
193	H	-2.1226	-4.0983	-5.5172
194	H	-2.9549	-2.6653	-4.9082
195	H	-2.1769	-3.7916	-3.7782
196	H	1.9431	-1.1995	-1.3868
197	N	0.8671	-0.0110	0.1092
198	C	1.2298	1.3173	0.6314
199	H	2.0424	1.2345	1.3635
200	C	0.0237	2.0349	1.2310
201	H	0.3094	3.0190	1.6077
202	H	-0.7406	2.1792	0.4619
203	H	-0.4193	1.4718	2.0550
204	H	1.6210	1.9085	-0.2013
